# Metagenomics Investigation of Agarlytic Genes and Genomes in Mangrove Sediments in China: A Potential Repertory for Carbohydrate-Active Enzymes

**DOI:** 10.3389/fmicb.2018.01864

**Published:** 2018-08-14

**Authors:** Wu Qu, Dan Lin, Zhouhao Zhang, Wenjie Di, Boliang Gao, Runying Zeng

**Affiliations:** ^1^School of Life Sciences, Xiamen University, Xiamen, China; ^2^Novogene Bioinformatics Technology Co. Ltd., Tianjin, China; ^3^Key Laboratory of Marine Genetic Resources, Third Institute of Oceanography, State Oceanic Administration, Xiamen, China; ^4^Key Laboratory of Marine Genetic Resources, Xiamen, China

**Keywords:** mangrove sediments, metagenomics, deep-sequencing, CAZyme, agarlytic gene

## Abstract

Monosaccharides and oligosaccharides produced by agarose degradation exhibit potential in the fields of bioenergy, medicine, and cosmetics. Mangrove sediments (MGSs) provide a special environment to enrich enzymes for agarose degradation. However, representative investigations of the agarlytic genes in MGSs have been rarely reported. In this study, agarlytic genes in MGSs were researched in detail from the aspects of diversity, abundance, activity, and location through deep metagenomics sequencing. Functional genes in MGSs were usually incomplete but were shown as results, which could cause virtually high number of results in previous studies because multiple fragmented sequences could originate from the same genes. In our work, only complete and nonredundant (CNR) genes were analyzed to avoid virtually high amount of the results. The number of CNR agarlytic genes in our datasets was significantly higher than that in the datasets of previous studies. Twenty-one recombinant agarases with agarose-degrading activity were detected using heterologous expression based on numerous complete open-reading frames, which are rarely obtained in metagenomics sequencing of samples with complex microbial communities, such as MGSs. Aga2, which had the highest crude enzyme activity among the 21 recombinant agarases, was further purified and subjected to enzymatic characterization. With its high agarose-degrading activity, resistance to temperature changes and chemical agents, Aga2 could be a suitable option for industrial production. The agarase ratio with signal peptides to that without signal peptides in our MGS datasets was lower than that of other reported agarases. Six draft genomes, namely, Clusters 1–6, were recovered from the datasets. The taxonomic annotation of these genomes revealed that Clusters 1, 3, 5, and 6 were annotated as *Desulfuromonas* sp., *Treponema* sp., Ignavibacteriales spp., and Polyangiaceae spp., respectively. Meanwhile, Clusters 2 and 4 were potential new species. All these genomes were first reported and found to have abilities of degrading various important polysaccharides. The metabolic pathway of agarose in Cluster 4 was also speculated. Our results showed the capacity and activity of agarases in the MGS microbiome, and MGSs exert potential as a repertory for mining not only agarlytic genes but also almost all genes of the carbohydrate-active enzyme family.

## Introduction

Given the demand for bioenergy, researchers have isolated numerous genes and genomes with biomass-degrading functions (Harris et al., 2010; Langston et al., 2011; Sheydina et al., 2014; Yang et al., 2016) to produce fermentable monosaccharides. To date, most of these works have focused on screening cellulase genes, and cellulose is the usual material for producing fermentable monosaccharides. However, cellulose application is limited by two reasons. First, cellulose is extremely difficult to dissolve in water. Therefore, cellulases should be adsorbed on the cellulose surface to be hydrolyzed and thus have low catalytic efficiency (Lynd et al., 2002; Zhang and Lynd, 2003). Second, cellulose has high molecular weight (MW) and exists in natural plants; lignin is tightly intertwined with cellulose and has also high MW. Therefore, natural cellulose fibrils not only exclude water but also cellulases (Lynd et al., 2002; Zhang and Lynd, 2003). Therefore, pretreatment using molten salts (i.e., LiCl_3_ H_2_O), nonaqueous solvent systems (i.e., *N*,*N*-dimethylacetamide/LiCl, DMSO/SO_2_, and *N*-methylmorpholine-*N*-oxide) (Heinze and Liebert, 2001), supercritical carbon dioxide (SC–CO_2_) (Kim and Hong, 2001), and ionic liquids (Kuo and Lee, 2009) is needed to separate lignin from cellulose and increase cellulose solubility. According to a previous study, the requirement for pretreatment restricts the application of cellulose hydrolysis (Jeoh et al., 2007). Meanwhile, the cost of above-mentioned cellulose pre-treatment is very expensive. Therefore, producing fermentable monosaccharides through cellulose hydrolysis by cellulases still has limited application. Agarose extracted from red algae has attracted increasing attention in recent years because of its high yield similar to cellulose (Roesijadi et al., 2010; Wargacki et al., 2012; Yanagisawa et al., 2013). Agarose exhibits properties that address the limitations of cellulose. Individual molecular agarose shows no complex coexistence with other molecular particles. Moreover, the solubility of agarose is higher than that of celluloses, and agarases could directly function on agarose dissolved in water. Accordingly, the enzymatic hydrolysis efficiency of agarose is significantly increased compared with that of celluloses. Therefore, agarose can be simply extracted and dissolved by heating, which is easier and cost saving than the current pre-treatment methods for cellulose. Hence, agarose is a potential alternative to cellulose for fermentable monosaccharide production. Ethanol and hydrogen (H_2_) (Kim et al., 2012; Wu et al., 2017) are fermentation products obtained after agarose saccharification using three types of agarlytic enzymes, including α-agarase, β-agarase, and α-1,3-neoagarobiose hydrolase (NABH) (Suzuki et al., 2003; Ohta et al., 2005; Watanabe et al., 2017). Neoagarooligosaccharides (NAs) produced by β-agarase degradation also exert many biological activities (Hong et al., 2017a,b), including anti-oxidation, anti-inflammation (Wang et al., 2004; Enoki et al., 2010), probiotic, and whitening properties (Hu et al., 2006). Therefore, the demand for enzymes with outstanding agarose-degrading activity is high.

Appropriate environmental samples containing considerable amount of these genes are required to isolate agarlytic genes. For example, cattle rumen and termite intestines have been reported as excellent sources of cellulase genes (Warnecke et al., 2007; Hess et al., 2011; Liu et al., 2011). However, a repertory of the agarlytic genes has never been proved to date. Sediments of mangrove ecosystems have extremely complex microbial communities (Bharathkumar et al., 2008; Dias et al., 2009; Jiang et al., 2013; Wu et al., 2016). Leaves, crustacean carcasses, and algal debris are abundant in mangrove sediments (MGSs), which are potential sources of agarlytic genes. A few agarase genes have been isolated from MGSs by using pure culture and metagenomics library methods (Shome et al., 2000; Mai et al., 2016). However, these methods are biased and time consuming in making a general survey of the capacity of agarlytic genes in MGSs. High-throughput sequencing provides a fresh perspective to investigate sequences in environmental samples integrally and without bias through simple operations. This method can detect genes, determine the abundance and diversity of certain genes (Anantharaman et al., 2016; Slaby et al., 2017), and draft genomics information in environmental samples (Leung et al., 2011; Girotto et al., 2016). However, only few studies have investigated genes in MGSs (Andreote et al., 2012; Thompson et al., 2013; Alzubaidy et al., 2016) by metagenomics sequencing because of the extremely complex microbial community. Only one study reported on CAZyme genes in MGSs (Thompson et al., 2013). Furthermore, these reports have some drawbacks as follows. (i) The genes obtained in these datasets are usually incomplete, but all the open-reading frames (ORFs) are shown as the results, regardless of completeness. Therefore, the true number of genes in these studies will be lower than those in the results because the datasets may contain nonoverlapping fragments from the same ORF. (ii) Furthermore, the massive incomplete genes complicated the application of heterologous expressions to verify the catalytic activities of the corresponding proteins. (iii) Lastly, the draft genomes are important to understand the metabolic pathways of agarose in nonculturable microorganisms, which make up the majority of the microbial community in nature (Zengler et al., 2005; Lengeler et al., 2009; D’Onofrio et al., 2010) and cannot be assembled from these datasets because of the low coverage. From these studies, the actual capacity, diversity, and activity of the agarlytic genes in MGSs remain unexplored and unconfirmed.

To address the above mentioned concerns and conduct a comprehensive study on the agarlytic genes and related genomes in MGSs, we deeply sequenced the environmental DNA (eDNA) of MGSs in China by using the Illumina platform. We obtained ∼300 Gb of data in total, which is the maximum data size among the metagenomics projects of MGSs. The amount, diversity, and completeness of the agarlytic genes were significantly increased compared with the previous works. Moreover, we focused on the CNR agarlytic genes. Most of these CNR agarlytic genes were unreported in the NCBI nr database. Twenty-one recombinant agarases with agarose-degrading activity were discovered using heterologous expression in *Escherichia coli* cells. Aga2 showed potential for practical applications due to its high activity and stability against thermo and many chemical agents. The signal peptide analysis showed that the agarase ratio of with signal peptides to that without signal peptides in our MGS datasets was obviously lower than that of the agarases reported in NCBI nr database. Moreover, we assembled six draft genomes from our datasets with unsupervised machine learning. We also analyzed the abilities for polysaccharide degradation in our study. The results proved that MGSs are potential repertories of agarlytic genes and almost all families of CAZyme genes.

## Materials and Methods

### Sampling and Physicochemical Analysis

Sediment samples were collected from a mangrove forest located in Longhai City, Fujian, China (Zini Mangrove Nature Reserve; 24°20’ N, 117°45′ E). To increase the abundance of the agarase genes, we buried 1% agar with the sediments in five sites of the middle of mangrove with 2-m interval for 20 days. Sediments from the other three sites without any treatment served as control groups were also collected. Approximately 500 g each of the agar-enriched (enrichment groups) and unenriched sediments (control groups) was collected in several 50 mL sterile centrifugal tubes. The sediment samples were transported to the laboratory and stored in an ultra-low temperature freezer at −80°C.

Sediment temperature was measured with an alcohol thermometer *in situ*. Sediments salinity was determined by using a handheld salinity meter (ATAGO, Japan). The humidity of sediments was defined as the percentage of the weights of dry and wet samples and was measured accordingly. pH, total nitrogen, total phosphorus, and total carbon were measured by Qingdao Hengli Testing Co., Ltd. (China) in accordance with the national standards of China.

### eDNA Extraction

The Powersoil DNA Isolation Kit (MO BIO, United States) was used to extract the eDNA of MGSs in accordance with the manufacturer’s instructions except that the DNA was eluted by sterile water instead of Solution C6 provided in the kit. Approximately 0.5 g of each sediment sample was used in the DNA extraction. Three replications of extraction and purification were performed and pooled together to avoid extraction bias. The DNA purity and integrity were determined by 1% agarose electrophoresis and NanoDrop 2000 (Thermo Scientific, United States).

### Sequencing of 16S rRNA Genes

Primer pairs 338F and 806R with barcodes (Fan et al., 2014) were chosen to amplify the 16S rRNA genes from MGSs. According to former studies (Xiao et al., 2015; Zhang et al., 2015; Zheng et al., 2015), the polymerase chain reaction (PCR) system and programs are listed in **Supplementary Tables S1**, **S2**, respectively.

Polymerase chain reaction products were used to construct a paired-end library of Illumina Miseq (Illumina Inc., United States). The low-quality bases (<20) were removed. The paired reads were assembled based on the overlapping sequences. The identification and removal of chimeras, operational taxonomic units (OTUs) clustering, and taxonomic assignment were performed using Qiime (Caporaso et al., 2010). The beta-diversity were analyzed using the R software (R Core Team, 2016).

### Metagenomics Sequencing

Environmental DNA was used to construct the 400 bp and 6 kb library, which were sequenced using an Illumina HiSeq (Illumina Inc., United States) by Novogene Technology Co., Ltd. (China). Approximately 300 Gb of data was generated. The reads with more than 40 nt low-quality bases (quality value ≤ 38) were removed. Meanwhile, the reads with more than 10 nt “N” bases were filtered out of the datasets. Lastly, the reads overlapping more than 15 nt bases with the adapters were also removed. Accordingly, the clean data were obtained. All clean reads were assembled using MEGAHIT (Li et al., 2015) with the parameter of “–presets meta-large,” and the scaffolds were broken at N into the scaftigs (continuous sequences within scaffolds) (Mende et al., 2012; Nielsen et al., 2014). The scaftigs with the length of ≥500 nt were used for further analysis (Li et al., 2014; Sunagawa et al., 2015). The ORFs in the scaftigs (≥500 bp) were predicted by MetaGeneMark. A nonredundant gene catalog was obtained after processing by using the CD-HIT software. Gene abundance was calculated based on the number of reads mapped to the genes and the length of the genes. The databases, including the CAZy Database^1^ , Kyoto Encyclopedia of Genes and Genomes (KEGG^2^), and Evolutionary Genealogy of Genes: Nonsupervised Orthologous Groups^3^, were used for the functional annotation of genes. Unigenes were aligned with the sequences in these databases by using the DIAMOND software (blastp, cut-off *E*-value of 1*e*-5), and the best hits were chosen as the functional annotations. The amino acid sequences of the proteins annotated as agarlytic genes were realigned in the NCBI nr database by using the BLAST software (version 2.2.26) to determine the novelty of these sequences.

To examine the sequencing accuracy, we randomly picked and amplified 100 sequences from our datasets (**Supplementary Table S3**). The sequences of the PCR products were determined by Shanghai Majorbio Bio-Pharm Technology Co. (China) by using an ABI 3730xl DNA Analyzer (Thermo Scientific, United States). The results were compared with the original sequences through DNAman software.

### Prediction of the Optimum Temperature and Signal Peptide

The optimum temperatures of the agarlytic genes with complete ORFs were predicted using the method described in a previous report (Chu et al., 2016). Meanwhile, the signal peptides were predicted using the SignalP script (SignalP 4.1 for Linux) (Petersen et al., 2011). All the complete agarase genes in the enrichment and control groups were selected for the signal peptide prediction. For further study, 424 agarases (**Supplementary File**), which were found in the culturable microorganisms, metagenomic libraries, and genome sequencing, were selected for signal peptide prediction by using the same script.

### Chimerism Correction

The contig coverage was calculated using the bedtools based on the read and insert mapping performed by Bowtie. Coverage difference (CD) was calculated as follows: CD = abs(m1–m2)/min(m1, m2), where m1 and m2 are the coverage of the two adjacent windows. Contig would be broken at where CD was over 0.75 regardless it was based on read or insert mapping. After the correction, all datasets were pooled together, and deredundancy was performed for further genome assembly.

### Genome Assembly

CONCOCT (Alneberg et al., 2014) was employed to bin the contigs (>2 kb) into clusters on the basis of the sequence composition and coverage. The gene integrity and heterozygosity of each cluster were evaluated by Prodigal (Hyatt et al., 2010), and the alignment was conducted using RPS-BLAST (*E*-value: 1.0*e-*3; coverage: 50%) in the NCBI-COG database. On the basis of the reference, the clusters with coverage of single-copy genes above 75% were selected for further genome assembly. The reads from the 400 bp and 6 kb libraries that were mapped to the selected clusters were isolated with SOAP (version 2.21) (Li et al., 2008). Genome assembly was performed using SPAdes (version 3.10) (Bankevich et al., 2012) with different K-mer values (17, 27, 37, 47, 57, 67, and 77). The DNA fragments shorter than 500 bp were removed. Six draft genomes were obtained for further study through these methods.

For the taxonomic annotation of these draft genomes, BLASTn was first used to align the genomic sequences to NCBI nr database with the parameters of “-a 4 -e 1e-5 -b 50.” Sequences with *E*-value ≧10× minimum *E*-value were picked up (Qin et al., 2010). To further study the taxonomy of these sequences, least common ancestors (LCAs) method was performed using MEGAN software (Huson et al., 2011), and the taxonomy of the sequences was confirmed based on the taxonomic result before the first branch. Subsequently, the taxonomic annotation results of the six draft genomes were obtained according to the percentage of annotated results of the sequences within each draft genome.

### Agarase Gene Expression

Thirty agarase genes (*aga*–*aga30*) with random selection were expressed in *E*. *coli*. The primers designed based on ORFs are listed in **Supplementary Table S4**. All PCR products were ligated into the pEASY-Blunt E2 Expression Vector (TransGen Biotech, China) and transformed into *E*. *coli* BL21 (DE3) cells. The recombinant proteins were induced by 0.05 mM isopropyl β-D-1-thiogalactopyranoside at 16°C for 24 h.

### Agarase Activity Assay

Crude agarase was produced after ultrasonicating the host cells. The protein concentration was measured using a Pierce BCA Protein Assay Kit (Thermo Scientific, United States), following the manufacturer’s instructions. Agarase activity (U) was defined as the amount of enzymes that produced 1 μM of reducing sugar per minute under the assay conditions. The concentration of reducing sugar was measured using the 3,5-dinitrosalicylic acid (DNS) method (Miller, 1959) by using D-galatose as a standard. Different buffer solutions (acetate–NaOH, pH 3.0–6.0; phosphate–NaOH, pH 7.0–8.0; Tris–HCl, and pH 9.0–10.0) and temperatures (20–80°C) were used to determine the effects of pH and temperature on the agarase activity. The reaction mixture was composed of 5 μL of crude agarose and 245 μL of reaction buffer solution with 0.2% dissolved agarose. After incubating at a certain temperature (20–80°C) for 30 min, 750 μL of DNS was added and heated at 100°C for 10 min. The absorbance was measured at a wavelength of 550 nm. The heat-inactivated crude agarase served as the negative control. Each experiment was performed in triplicate.

### Purification and Activity Staining of Recombinant Agarase

Aga2 with the crude highest agarose-degrading activity was further studied after purification with HisPur Ni-NTA spin columns (Thermo, United States). Purified proteins were eluted by using 200 mM imidazole solution. Furthermore, purified proteins were dialyzed against 20 mM PBS (pH 7.4) to replace midazole and stored at −20°C with 30% glycerol (final concentration). The protein concentration was detected using Pierce BCA Protein Assay Kit (Thermo Fisher Scientific, United States) by using bovine serum protein as a standard. All the experiments followed the manufacturer’s instructions. The purified Aga2 was analyzed by 10% sodium dodecyl sulfate–polyacrylamide gel electrophoresis (SDS-PAGE) and stained with Coomassie Brilliant blue.

After SDS–PAGE, activity staining of Aga2 was conducted. SDS in the gel was removed by rinsing the gel in PBS (pH 7.4) for three times at about 30 min each time. Then, the gel was placed on 1% agar plate, and the plate was incubated at 30°C for 12 h. Iodine solution was used for staining the agar plate to show a clean zone at the Aga2 position.

### Determination of Thermal and pH Stability of Aga2

To determine the Aga2 stability against the temperature, the enzyme solution was preincubated at 20–80°C for 0.5 h at pH 7.0. In addition, the enzyme solution was preincubated at 50°C in pH 4.0–9.0 for 0.5 h to determine the stability of Aga2 against the pH. After preincubation, the residual activity of the enzyme solution was measured as mentioned above, and the activity in the absence of any treatment was defined as 100%. All experiments were performed in triplicates.

### Effects of Reagents on Aga2 Activity

The effects of metal ions and chelating agent were measured by determining the Aga2 activity with Ca^2+^, Ni^2+^, Fe^3+^, Zn^2+^, Ag^+^, Mn^2+^, Cu^2+^, and Al^3+^ at a final concentration of 1 mmol/L. The effect of denaturants was determined by mixing the reaction mixture with different concentrations of SDS, dithiothreitol (DTT), and beta-mercaptoethanol (beta-Me). The effects of salt concentration and metal chelator were measured with 10 and 100 mM NaCl and EDTA-2Na (pH 8.0), respectively. Agarase activity was measured. The Aga2 activity in the absence of any treatment was 100%. All experiments were performed in triplicates.

### Determination of Agarase Activity in MGSs

The extracellular agarase activity in the sediments was determined as follows. Initially, 10 g of fresh sediments and 10 mL of 2% agarose dissolved in phosphate-buffered saline (PBS) (10 mM, pH 7.4) were diluted to 100 mL with PBS. The mixture was incubated at 35°C for 72 h. The reaction mixture (2 mL) was added with 6 mL of DNS and heated at 100°C for 10 min. Absorbance was recorded at a wavelength of 550 nm.

Total agarase activity was determined as follows. A total of 10 g of fresh sediments were adequately suspended in 50 mL of PBS. The intracellular agarases were released after ultrasonication. The lysate and 10 mL of 2% agarose were diluted to 100 mL with PBS, and the remaining processes were performed as described above. The intracellular activity was obtained by subtracting the extracellular activity from the total activity. Sterilized sediments served as the negative controls. The agarase activity was defined as the mass (mg) of reducing sugar produced by per gram of sediment after incubation for 72 h.

### Nucleotide Sequence Accession Numbers

The Illumina HiSeq sequence data from this study were submitted to the EMBL-EBI European Nucleotide Archive under the Accession Nos. ERR2179512 (Mgv-CK), ERR2179511 (Mgv-B-L6000), and ERR2179510 (Mgv-B). The nucleotide sequences of the 21 agarase genes that were conformed with agar-degrading activity were submitted to GenBank under the Accession Nos. MG280837 (*aga1*), MG383539 (*aga2*), MG383540 (*aga4*), MG383541 (*aga5*), MG383542 (*aga6*), MG383543 (*aga7*), MG383544 (*aga9*), MG383545 (*aga10*), MG383546 (*aga12*), MG383547 (*aga13*), MG383548 (*aga14*), MG383549 (*aga15*), MG383550 (*aga16*), MG383551 (*aga18*), MG383552 (*aga19*), MG383553 (*aga21*), MG383554 (*aga22*), MG383555 (*aga23*), MG383556 (*aga27*), MG383557 (*aga28*), and MG383558 (*aga29*).

## Results

### Sample Description

For all the enrichment groups, the agar in the sediments changed dramatically, showing liquefaction and numerous holes. This phenomenon suggested agar degradation. However, the enrichment groups were changed into two colors, namely, yellow and black, respectively. In detail, three sites were changed into black and two sites were changed into yellow. Physicochemical parameters of the enrichment groups were measured along with the control groups (without any treatment). Results showed that there was no obvious difference among the samples from enrichment groups (**Table 1**) except the colors. Therefore, the enrichment groups were classified into two groups according to the color, namely, Mgv-Y (yellow) and Mgv-B (black). Meanwhile, the control groups were named Mgv-CK. The eDNA from same group was pooled together for further analyses.

**Table 1:** Physicochemical parameters of the sediment samples^∗^.

Group	Sample No.	pH	Temperature (°C)	Salinity ‰	Humidity (%)	TC (g/kg)	TP (g/kg)	TN (g/kg)
Mgv-B	1	6.41	25.4	15.1	20.9	5.9	0.127	0.325
	2	6.33	24.5	16.0	16.8	5.3	0.109	0.344
	3	6.94	25.5	15.4	15.2	5.1	0.174	0.239
Mgv-Y	4	6.24	26.2	15.0	23.5	4.9	0.146	0.368
	5	6.78	24.9	15.3	21.1	5.4	0.109	0.308
Mgv-CK	6	6.51	26.7	16.7	18.4	3.8	0.140	0.408
	7	6.15	25.8	16.4	23.1	4.2	0.135	0.586
	8	6.43	25.1	15.6	22.9	4.6	0.156	0.301

*^∗^TC, TP, and TN represented total carbon, total phosphorus, and total nitrogen, respectively.*

### Change in the Microbial Diversity in MGSs After Enrichment

We analyzed the diversity of the 16S rRNA genes to confirm whether the agar enrichment was effective and to identify which of the two enrichment samples (Mgv-Y and Mgv-B) was more effectively agar-enriched. The sequencing depth was sufficient according to rarefaction curve (**Supplementary Figure S1**).

The alpha-diversity of microbes in MGSs varied after enrichment (**Table 2**). The abundance represented by abundance-based coverage estimator (ACE) and chao indices increased in Mgv-Y and Mgv-B groups; however, the microbial diversities represented by Shannon and Simpson indices of Mgv-Y and Mgv-B groups were lower than Mgv-CK, indicating that agar enrichment could increase the microbial abundance but reduce the diversity in the sediments. Moreover, compared with Mgv-Y, the variation of microbial alpha-diversity was more obvious in Mgv-B.

**Table 2:** Alpha-diversity indices of microbial 16S rRNA genes in the sediment samples after subsampling.

Group	Reads No.	ACE	Chao	Shannon	Simpson
Mgv-B	59755	1991	2025	5.94	0.0118
Mgv-Y	59755	1962	1986	6.25	0.0052
Mgv-CK	59755	1882	1921	6.56	0.0033

**FIGURE 1:**
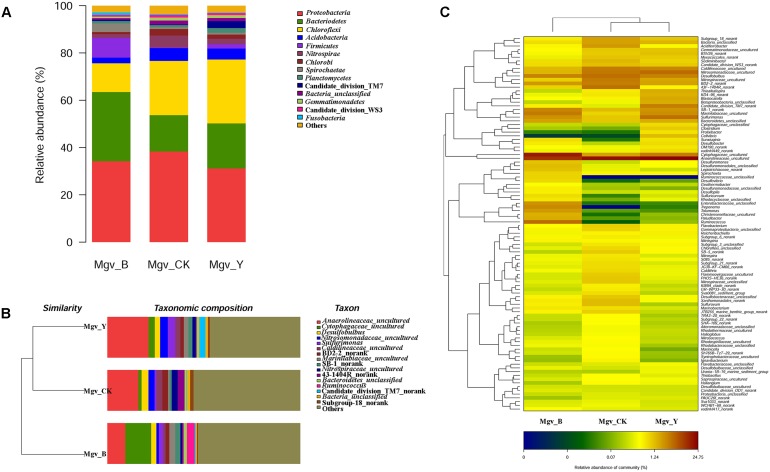
Diversity of 16S rRNA genes in the enrichment and control groups. **(A)** Community composition (phylum level). **(B)** Sample clustering (genus level). **(C)** Heatmap of community composition (genus level).

The beta-diversity results showed that the community of the two enrichment groups was significantly changed relative to that of Mgv-CK (**Figure 1**). Proteobacteria was the dominant phylum in MGSs, followed by Bacteroidetes, Chloroflexi, Acidobacteria, and Firmicutes. After enrichment, the abundance of Bacteroidetes and Firmicutes increased in Mgv-B and Mgv-Y, and most obvious increasing occurred in Mgv-B (**Figure 1A**). The microbial community in Mgv-Y was still similar to the control group (Mgv-CK). However, the microbial Mgv-B structure was significantly shifted and phylogenetically distanced from the others (**Figure 1B**). On the genus level, several genera, such as *Acidaminococcus*, *Aeromonas*, *Arcobacter*, *Bacteroides*, and *Ruminococcus* were enriched in Mgv-B and Mgv-Y. Among these genera, 10/16 was reported with the ability of glycoside hydrolysis (**Figure 1C** and **Supplementary Table S5**). Similarly, the most significant change occurred in Mgv-B (**Figure 1C** and **Supplementary Table S5**).

Therefore, eDNA of Mgv-B with the most obvious changes after enrichment was further sequenced along with the control group (Mgv-CK).

### Metagenomics Data Overview

In total, ∼300 Gb of the data was generated from the enrichment and control groups (**Supplementary Table S6**). Moreover, 25,172,487 ORFs (13,049,096 from the control group and 12,123,391 from the enrichment group) were found in our datasets. Among these sequences, nearly 1/5–1/4 had complete ORFs (**Supplementary Table S7**). To examine the accuracy of sequencing and assembly, we randomly sequenced and amplified 100 sequences by using PCR, and 97 out of the 100 amplified and sequenced fragments were found with more than 97% identity of the original sequences. This result suggested the high accuracy of our datasets (**Supplementary Figure S2**).

Except for the microbial community, the number of the function genes was varied (**Supplementary Figure S3**). The gene abundance related to “Environmental Information Processing,” “Genetic Information Processing,” and “Cellular Processes” increased after enrichment. For example, the abundance of ABC transporter genes increased from 1.1% to 1.5%. The abundance of ribosome genes increased from 0.5% to 0.9%. The gene abundance of “Cellular Processes” evidently increased. Moreover, after agar enrichment, the abundance of bacterial chemotaxis and flagellar assembly genes increased from 0.1% to 0.4% and from 0.08% to 0.3%, respectively.

### CAZyme Genes in MGSs

To investigate the capacity of CAZymes in MGSs, the CNR CAZyme genes of the control group were emphatically analyzed. These results included the auxiliary activities, carbohydrate-binding modules (CBMs), carbohydrate esterases, glycoside hydrolases (GHs), glycosyl transferases, and polysaccharide lyases (PLs) (**Supplementary Figure S4** and **Supplementary Table S8**).

In the Mgv-CK datasets (control group), 12,195 CNR GH genes were found (**Supplementary Table S9**). This value was significantly higher than the number in the previous mangrove studies. GH 3 and GH 13, which are known as the important families related to polysaccharide hydrolysis, were abundant in the MGS datasets. In addition to GH 3 and 13, other families associated with cellulose hydrolysis, such as GH 6, GH 12, GH 17, CBM 2, and CBM 3, were also detected with high abundance in the datasets. The lysozyme family GH 23 was as well abundant. The pectin lyase families, namely, PL 1 and PL 9, achieved the highest numbers in the PL family.

The number of the CNR genes from each GH family from our datasets was compared with those from other datasets, including the previous metagenomic projects on MGSs, termite gut, and cow rumen microbiome (**Supplementary Table S9**). Similar or even higher amounts of CNR genes from the GH family were found in our datasets. Some GH families, such GH 15 and GH 23, were not detected in the datasets of termite gut and cow rumen microbiome but were abundant in the datasets of this study. Therefore, although only CNR genes were involved in analysis, the diversity and abundance of GH genes from this study had clear advantages compared with that of other datasets.

### Abundance and Diversity of the Agarlytic Genes in MGSs

**FIGURE 2:**
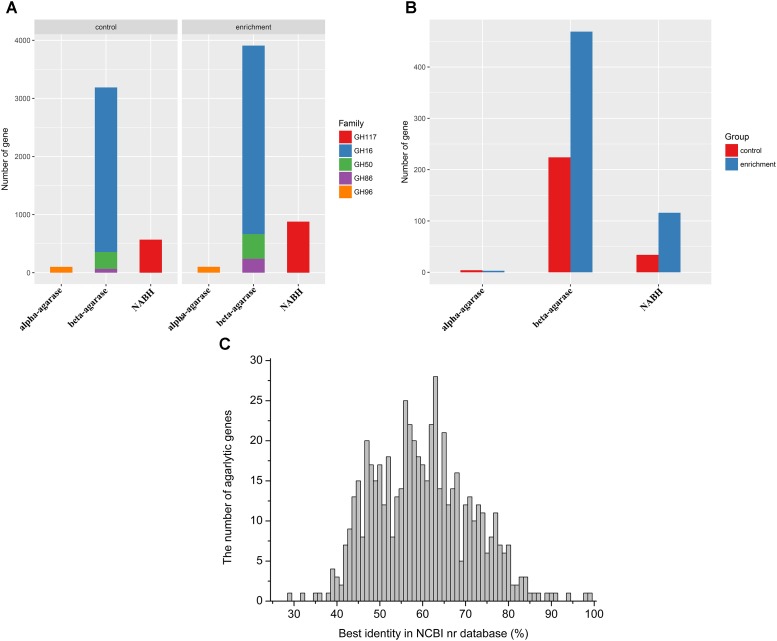
Abundance and novelty of agarlytic genes in the MGS datasets. **(A)** The number of all agarlytic genes found in the datasets (NABH, α-1,3-neoagarobiose hydrolase). **(B)** The number of complete and nonredundant (CNR) agarlytic genes in the datasets. **(C)** The distribution of the best identity of the corresponding amino acid sequences of the agarlytic genes in the NCBI nr database.

Agarlytic genes belonging to GH 16, GH 50, GH 86, GH 96, and GH 117 were found in the datasets (**Figure 2A**). Furthermore, only the CNR agarlytic genes were involved in the follow-up analysis. GH 16 was the most abundant among these families. A total of 262 CNR agarlytic genes were observed in the control group, and the number of agarlytic genes was significantly increased to 588 in the enrichment group (**Figure 2B**). The number of beta-agarase and NABH genes rose from 224 and 34 to 469 and 116, respectively. In addition, the number of CNR NABH genes originating only from the datasets of enrichment group was 70% higher than those from all the previous reports according to the CAZy database (**Supplementary Table S9**).

The corresponding amino acid sequences of the CNR agarlytic genes were aligned in the NCBI nr database, and the best identities are shown in **Figure 2C**. The main distribution range was from 45% to 80%. This result suggested that most of these agarlytic genes in MGSs are unreported.

### Recombinant Agarase Activity

**FIGURE 3:**
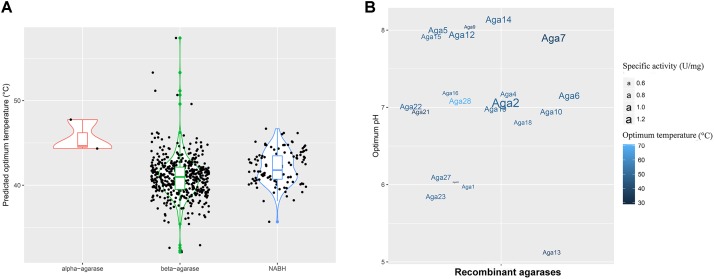
Enzyme properties of agarases in the MGS datasets. **(A)** Density curve of the predicted optimum temperatures of the agarase in the MGS datasets. **(B)** The effects of pH and temperature on the activities of crude Aga1–Aga30. The color shades correspond to the optimum temperatures, and the text sizes stand for the specific activities of agarases.

The optimum temperature of these agarase genes was predicted (**Figure 3A**). The values were mainly distributed between 40 and 50°C. This finding indicated that most of the agarases from MGSs were mesophilic.

To utilize the agarase activity authentically in practical production, 30 genes (*aga1*–*aga30*) were expressed in the *E*. *coli* cells. Moreover, the effects of temperature and pH were determined (**Supplementary Figures S5**, **S6**). A total of 21 genes out of 30 genes were detected with activity (**Figure 3B**). Moreover, as predicted, the optimum temperatures were mainly 40 and 50°C. Crude Aga2 was detected with the highest specific activity (1.2 U/mg) among these agarases. Furthermore, a few extremozymes were found. The optimum temperatures for Aga28 and Aga29 were 30 and 70°C, respectively. As another example, Aga13 possessed the optimum pH (pH 5.0). This pH was lower than those of the others.

### Agarose-Degrading Characteristics of Aga2

**FIGURE 4:**
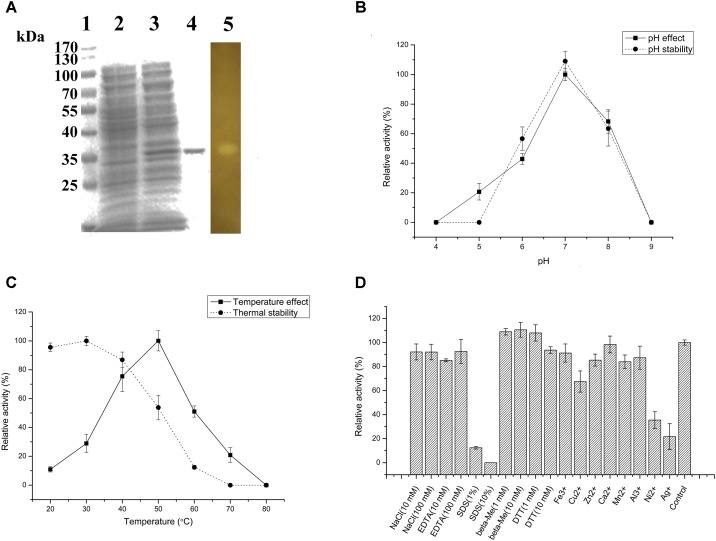
Agarose-degrading characteristics of purified Aga2. **(A)** SDS–PAGE result. Lanes 1–5 stand for the standard MWs of proteins, supernatant of the cell disruption liquid with and without isopropyl β-D-thiogalactoside (IPTG) induction, purified Aga2, and the result of Aga2 activity staining. **(B)** The effects of pH on the activity and pH stability of purified Aga2. **(C)** The temperature effects on the activity and thermal stability of purified Aga2. **(D)** The effects of chemical agents on the activity of purified Aga2 (SDS, sodium dodecyl sulfate; DTT, dithiothreitol; beta-Me, beta-mercaptoethanol).

The agarose degradation characteristics of Aga2 were further studied after purification because of the highest crude enzyme activity of Aga2. The amino acid sequence of Aga2, which belonged to GH 16 family, showed 61.8% identity with the β-agarase from *Bacteroides sartorii* (WP_025018818.1). The MW of Aga2 was ∼37 kDa according to SDS–PAGE. A clean zone was observed at the position of Aga2 after activity staining (**Figure 4A**). The optimum pH (7.0) and temperature (50°C) of the purified Aga2 (**Figures 4B,C**) were in agreement with the previous results of the crude Aga2 (**Figure 3B**). The specific activity of Aga2 was 234.05 U/mg in the optimum conditions mentioned above. Aga2 was sensitive to pH and was only stable at a pH level of 7.0 (**Figure 4B**). Moreover, the Aga2 activity retained more than 85% after the preincubation at 20–40°C (**Figure 4C**). Aga2 resisted most chemical agents, including NaCl, EDTA-2Na, and metal ions (except Cu^2+^, Ni^2+^, and Ag^+^). DTT and beta-Me increased the Aga2 activity moderately. However, most of the activity was lost with 1% and 10% SDS (**Figure 4D**).

### Analysis of the Signal Peptides of the Agarlytic Enzymes in MGSs

**FIGURE 5:**
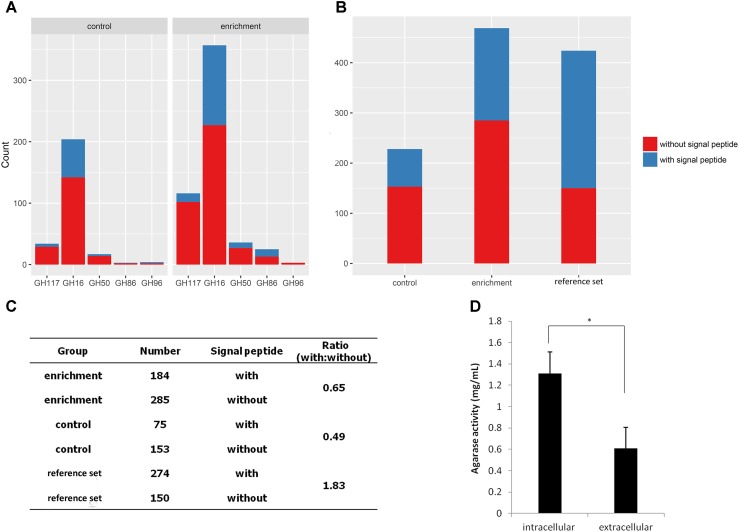
Analysis of agarase signal peptides in the MGS datasets. **(A)** Signal peptide statistics of agarase in each GH family. **(B)** The signal peptide statistics of agarases in different datasets. **(C)** The ratio of agarases with signal peptides to those without signal peptides. **(D)** Extracellular and intracellular agarase activities in MGSs.

Subsequently, the signal peptides of CNR agarases were analyzed using the SignalP script (**Figure 5A**). A total of 60.8% of the agarases were predicted without a signal peptide (**Figure 5C**). This result indicated that most of the agarases from MGSs cannot be transported out of the cell. Furthermore, the intracellular agarase activity (1.31 mg/mL) was significantly higher than the extracellular agarase activity (0.61 mg/mL, *p* = 0.014) in MGSs. This finding verified the possibility that more agarases remained in the cells of the microorganisms (**Figure 5D**).

To investigate whether this result is exclusive to our datasets or a general phenomenon, 424 agarases were selected as the reference set from the NCBI nr database to predict the signal peptide. The agarase ratio with signal peptides to that without signal peptides in the reference set (1.83) was significantly higher than those in the enrichment and control groups (0.65 and 0.49, respectively) (**Figures 5B,C**).

### Recovery of the Draft Genomes From the MGS Datasets

Sequences from our datasets were binned and assembled into six draft genomes, which were named as Clusters 1–6 (**Supplementary Tables S10**, **S11**). According to the taxonomic annotation results (**Supplementary Table S11**), Clusters 1, 3, 5, and 6 were annotated as *Desulfuromonas* sp., *Treponema* sp., Ignavibacteriales spp., and Polyangiaceae spp., respectively. Meanwhile, Clusters 2 and 4 were potential new species. The accuracy of binning and assembly was verified by a 15-mer frequency distribution and the relationship between the GC content and sequence depth (**Supplementary Figure S7**). The result showed that the relationship between the GC content and sequence depth basically fitted the normal distribution, and the distribution 15-mer frequency accorded with the poisson distribution, indicating a relatively high accuracy of these clusters. All these clusters lacked the reported reference genomes and were firstly reported. Clusters 2 and 4 were potential new species because the proteins in these genomes lack the concentrated BLAST hits in the NCBI nr database (**Supplementary Table S11**).

### Polysaccharide Degradation in the Draft Genomes

**FIGURE 6:**
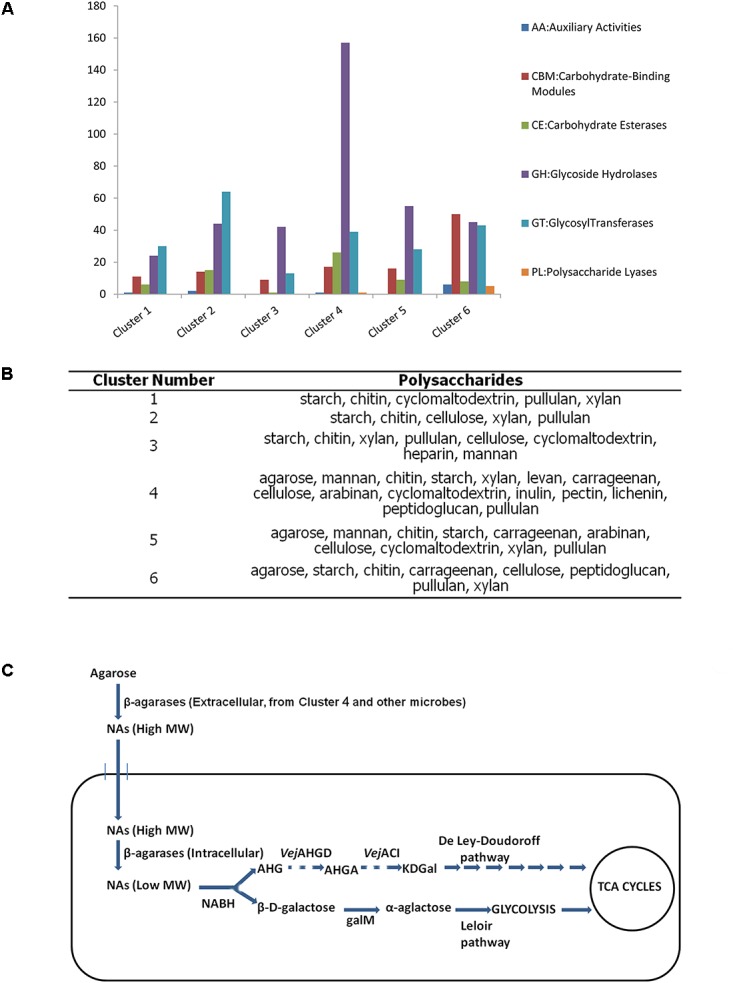
Analysis of the six draft genomes. **(A)** The number of CAZyme genes in each draft genome. **(B)** Polysaccharides that can be degraded by each draft genome. **(C)** Putative pathway of agarose utilization in cluster 4 based on the functional annotation and location prediction of the agarlytic genes. The pathway shown with dashes suggests the related genes absent in the genome of cluster 4 (NAs, neoagarooligosaccharides; MW, molecular weight; NABH, α-1,3-neoagarobiose hydrolase; AHG, 1,6-anhydro-β-D-galactose; *Vej*AHGD, AHG dehydrogenase; AHGA, 3,6-anhydrogalactonate; *Vej*ACI, 3,6-anhydrogalactonate cycloisomerase; KDGal, 2-keto-3-deoxygalactonate; galM, aldose 1-epimerase).

All the six clusters were found with many genes related to glycometabolism (**Figure 6A**). Cluster 4 had the most genes for carbohydrate metabolism among these draft genomes according to the annotation results of KEGG database (**Supplementary Figure S8**). Meanwhile, most GH genes were found in Cluster 4 (**Figure 6A**). All these clusters possessed the degrading abilities of important polysaccharides, including starch, chitin, xylan, and cellulose (**Figure 6B**). Cluster 4 also had the most wide-ranging degrading ability for polysaccharides (**Figure 6B**).

Three clusters, namely, Clusters 4, 5, and 6, were found to harbor agarlytic genes. The locations of these enzymes were predicted (**Supplementary Table S12**). Most of the agarases in these genomes were located in the cytoplasm and had no signal peptides. This finding was in line with the analysis of the signal peptides in the datasets. Cluster 4 had the most CNR genes for utilizing agarose (**Supplementary Table S12**). Therefore, the agarose metabolism pathways of this cluster were speculated based on the annotation results from the CAZy and KEGG databases (**Figure 6**). The only agarase in Cluster 4 with signal peptide was transported outside the cell to cut the agarose into NAs that were subsequently transported into cells with the other NAs produced by the agarases in the environment. Agarases in the cytoplasm acted on the NAs to produce oligosaccharides with low MW. β-D-Galactose and 1,6-anhydro-β-D-galactose (AHG) were produced by NABH. β-D-Galactose was isomerized to α-galactose by galM, an aldose 1-epimerase. α-Galactose is further metabolized into glycolysis through the Leloir pathway. The metabolic pathway of AHG was absent in these clusters.

## Discussion

The organic carbon content in MGSs was studied in former studies. Bouillon et al. (2003) proved that the organic carbon content in India’s MGSs was highly variable with a content of 0.6–31.7% dry weight. Their finding showed that this condition could affect the carbon dynamics in mangrove ecosystems (Bouillon et al., 2003). Donato et al. (2011) investigated 25 mangroves across of the Indo-Pacific region, and their results showed that an average 1,023 mg carbon per hectare was contained in mangroves, and these mangroves are among the most carbon-rich forests in the tropics. Kenyan mangroves were investigated with an estimated belowground carbon of 69.41 t (Gress et al., 2017). Kelleway et al. (2016) found that the 70-year mangroves contained approximately 12–15 kg/m^2^ aboveground biomass and 20–30 kg/m^2^ underground carbon. Therefore, mangroves serve as enormous warehouses for both aboveground and underground carbon. The genes for carbon cycling have been investigated by metagenomics sequencing (Andreote et al., 2012). Polysaccharides, such as cellulose, agarose, starch, and xylan, were important carbon forms in MGSs. Thus, MGSs are ideal environment for the enrichment of bacteria carrying polysaccharide-degrading genes. However, the abundance of genes responsible for polysaccharide degradation, which is the first step for carbon cycling and biofuel production, has rarely been comprehensively studied in early works. In former studies on MGSs by metagenomics sequencing, 454 pyrosequencing was the most frequently used method (Andreote et al., 2012; Thompson et al., 2013; Alzubaidy et al., 2016), and the relatively long reads from this strategy can improve the assembly effect. Thompson et al. (2013) investigated polysaccharide-degrading genes in Brazilian mangroves. Many cellulolytic genes and sequences, including cellulases, hemicellulases, carbohydrate-binding domains, dockerins, and cohesins were detected, indicating that the microbiome in MGSs harbor all of the molecular tools for cellulose degradation and is an ideal source for cellulolytic genes. However, only 1,269,282 raw reads (∼1.008 Gb) for two samples were obtained in that study. For extremely complex microbial community in MGSs, this data size does not cover the majority of genetic information. Therefore, the real capacity of polysaccharide-degrading genes in MGSs can be more abundant than the data in that work. In the current study, we considerably increased the sequencing data to ∼300 Gb, which is approximately a 100- to 1000-fold larger than those in previous reports. The abundance and completeness of the genes sharply increased with data size in our work.

Few studies have reported on agarases from MGSs. Shome et al. (2000) isolated the agar and agarose-degrading bacterium *Alteromonas* spp. from 1000 soil samples collected from various mangroves located in South Andaman. This bacterium can produce an extra-cellular exo-acting agarase whose optimum pH and temperature are 6.0–9.0 and 25–37°C, respectively (Shome et al., 2000). Mai et al. (2016) constructed a fosmid metagenomic library containing ∼3.0 Gb of genetic sequences using eDNA from MGSs, and a β-agarase belonging to GH 16 has been isolated from the library by functional screening. The optimum pH and temperature of the agarase were 7.0 and 50°C, respectively, and this agarase was the first to be isolated from a metagenomics library of MGSs in 2016 (Mai et al., 2016). Agarases from other GH families have not been reported. In the current study, numerous agarlytic genes belonging to GH 16, GH 50, GH 86, GH 96, and GH 117 were discovered by deep sequencing. To improve the accuracy of our investigation, we only analyzed the CNR ORFs to discover the abundance and diversity of the agarlytic genes in MGSs. The results (**Supplementary Table S9**) showed that 204, 17, 3, 4, and 34 CNR agarase genes belonged to GH 16, GH 50, GH 86, GH 96, and GH 117 were found in control group, respectively. Furthermore, 409, 36, 25, 3, and 116 CNR agarase genes belonged to GH 16, GH 50, GH 86, GH 96, and GH 117 were found in enrichment group, respectively. Obvious differences in the capacity and diversity of agarlytic genes were observed between a former study on MGSs (Thompson et al., 2013) and our work because no genes belonged to GH 50, GH 86, GH 96, and GH 117 in that study (**Supplementary Table S9**). Therefore, the capacity and diversity of agarlytic genes in MGSs microbiome is very abundant and seriously underestimated in former mangrove investigation (Thompson et al., 2013).

Agarlytic genes are not the only gene class that is abundant and diverse in our MGS datasets. Starch, cellulose, and xylan are also important and abundant polysaccharides in MGSs, and the enzymes that degrade these polysaccharides were detected with great amount in our MGS datasets. Amylases mainly belong to GH 13 (Andrej et al., 2010; Majzlová et al., 2013; Peng et al., 2013; Štefan et al., 2015), whereas cellulases are mostly grouped into GH 3, GH 6, GH 12, and GH 17 (Sandgren et al., 2005; Van et al., 2012; Lima et al., 2013; Tishkov et al., 2013; Zhao et al., 2013; Jennifer et al., 2016; Zhang et al., 2016; Uchiyama et al., 2017). Xylanases are classified into GH 10 and GH 11 (Beaugrand et al., 2004; Dumon et al., 2008; Zhang et al., 2011; Paës et al., 2012; Wang et al., 2012). Therefore, these GH families are crucial for polysaccharide degradation in nature. The number of each GH in termite gut and rumen microbiome, two proven warehouses for cellulolytic genes, is listed in **Supplementary Table S9**. In termite gut microbiome datasets, 69, 46, 14, and 48 genes belonging to GH 3, GH 10, GH 11, and GH 13 were found. In rumen microbiome datasets, 2844, 1025, 165, and 3442 genes belonging to GH 3, GH 10, GH 11, and GH 13 were identified (**Supplementary Table S9**). However, few GH 6, GH 12, and GH 17 genes were annotated in both datasets. Although the number of genes belonging to GH 3, GH 10, GH 11, and GH 13 were less in our MGSs datasets (both control and enrichment group) than that in rumen microbiome, the genes from these families in our MGS datasets were more abundant than those in termite gut datasets (**Supplementary Table S9**). The GH families that are not or rarely detected in termite gut and rumen microbiome, such as GH 6, GH 12, GH 17, GH 46, GH 47, GH 102, and GH 103, were abundant in our MGS datasets. Compared with former datasets, our results showed high diversity and capacity of not only agarlytic CNR genes but also that of nearly all CNR GH family genes in MGS microbiome.

Interestingly, few agarlytic bacteria and genes were isolated from the MGS microbiome but with high capacity of agarases proven by our data. Functional screening strategy is usually employed for the isolation of functional genes from environmental samples for both pure culturing and metagenomics library construction. Signal peptides are contiguous amino acid sequences that are mostly present in proteins and directs protein localization (von Heijne, 1990). Given the lack of membranous organelles in bacteria, bacterial proteins containing signal peptides must be transported extracellularly or to the cell membrane. Thus, functional screening-based strategy heavily depends on the signal peptide in enzymes because no significant phenomenon can be observed during functional screening if the protein cannot transport extracellularly. Therefore, we analyzed signal peptide within agarases from MGS datasets. Normally, agarases must be transported to or outside the cell membrane to play a role because high MW agarose can barely be transported into cells. Therefore, 64.6% of the agarases from the reference set were predicted to possess signal peptides (**Figure 5C**). However, the proportion of agarase with signal peptides was significantly lower in the mangrove datasets (39.4 and 32.9% for enrichment and control group, respectively) than that in the reference set. This result may explain the reason of rare agarases isolated from MGSs. The deficiency of signal peptide in most of the agarases from MGSs caused difficulty in discovering agarlytic genes through functional screening. Moreover, this observation suggested that only a small portion of agarases are carried externally from the cell to degrade agarose into low-MW oligosaccharides that can be transported into cells. The rest of the agarases are retained in the cells to degrade entering oligosaccharides, such as the pathway of agarose degradation in Cluster 4. Using this strategy, a bacterium can highly utilize the oligosaccharides produced by other agarlytic microbes, and the resources and energy for signal peptide recognition, translocation, and resection of most agarases can be conserved. Therefore, utilizing agarose in MGSs may be collaborative and economical.

Full-length of ORFs is difficult to obtain using sequencing-based metagenomics, Especially in samples containing complex microbial community. Brulc et al. (2009) investigated the cellulolytic genes in rumen microbiome and found that CBM domains that are under-represented in cellulolytic genes are often absent, which caused incomplete ORFs in the datasets. Similar results were obtained in the microbiome of *Tammar wallaby* by Pope and Moran (2010). Hess et al. (2011) stated that sequence-based metagenomic discovery of complete genes from environmental samples is limited by the microbial species complexity of most environments and the consequent rarity of full-length genes in low-coverage metagenomic assemblies. Therefore, increasing the environmental samples with complex microbial community is important to improve the completeness of ORFs. Unfortunately, MGSs are among complex environmental samples. Hence, full-length ORFs were rarely obtained in former metagenomics sequencing studies on MGSs. The incomplete ORFs will weaken the practical value of metagenomics studies because of problems in protein expression. Unlike the case for these metagenomics studies, we aimed to obtain complete agarase genes for heterologous expressions to identify the real activities of the enzymes for possible production applications by substantially increasing the depth of sequencing. Consequently, 469 and 228 CNR agarase genes were obtained in the enrichment and control groups, respectively. A total of 21 expressed CNR agarase genes were detected with the ability to degrade agarose. Some enzymes (9 out of the 30 enzymes) lacked this activity because of possibly low expression levels, inclusion body formation, and inappropriate conditions for protein folding and functioning. Among these agarases, Aga28 and Aga29 could work optimally at extreme temperatures (30 and 70°C, respectively). Aga2, which exhibits the highest crude enzyme activity of agarose degradation among the agarases expressed in this study, was purified. We found that Aga2 demonstrates a MW of 37 kDa and a high specific activity of 234.05 U/mg. Former review (Ting and Moo, 2010) summarized the properties of existing recombinant agarases and showed that except for two recombinant agarase (agaA and agaB) with extremely high activity, the MWs and specific activities of these agarase mainly ranged from 30 to 146 kDa and from 16.4 to 398 U/mg, respectively, which demonstrated that Aga2 possessed a lower MW and a higher specific activity among most recombinant agarases, indicating Aga2 could efficiently degrade agarose with less consumption of amino acids. In addition, Aga2 is resistant to temperature change from 20 to 40°C and many chemical agents. In our parallel study (Di et al., 2018), Aga1 was renamed AgaM1 to avoid duplication and was further purified for the analysis of thermodynamic and kinetic properties, and the results showed the purified Aga1 possessed extraordinary thermo and pH stabilities, even better than Aga2. In fact, the weak stability against temperatures and pHs limited the application of many agarases (Shome et al., 2000; Fu et al., 2009; Dong et al., 2016; Zhu et al., 2016; Jung et al., 2017), and thus, these mangrove-derived enzymes with excellent stabilities were good options for industrial production with complex and varying conditions. Given that most of these CNR agarase genes are unreported, the agarases in MGSs are not only abundant in number, but also possess sequence novelty and outstanding enzymatic characteristics.

Aga2 was grouped in the GH 16 family, and the degradation end-products of agarose by the agarase of GH 16 family are neoagarotetraose (NA4) and neoagarohexaose (NA6) according to CAZy database and former studies (Ohta et al., 2004; Dong et al., 2016). The biological activities of NA4 and NA6 have been confirmed by many works. Wang et al. (2017) demonstrated that NAs with different degrees of polymerization, including NA4 and NA6, possess anti-inflammatory activity in lipopolysaccharides-stimulated macrophages. NA4 and NA6 exhibit antibacterial activity against several Gram-negative and -positive bacteria (Hong et al., 2017a). NA4 and NA6 can also induce adiponectin production and effectively suppress obesity and obesity-related metabolic syndromes in obese mice (Hong et al., 2017b). Hou et al. (2014) demonstrated that treatment with neoagaro-oligosaccharide with 4–8 degrees of polymerization can reduce the consumption of fruit weight, titratable acid, and vitamin C contents of cherry tomatoes. Moreover, the safety of NA4 and NA6 has been confirmed by previous genotoxicity test (Hong et al., 2017c). Therefore, Aga2 expressed in this study possesses great potential in food and pharmaceutical industries. Furthermore, a great number of NABH genes belonging to GH 117 family were found in our datasets. NABH is key to hydrolyze NA2 into L-AHG and D-galactose, two valuable fermentable monosaccharides, by cleaving α-1,3 linkage (Watanabe et al., 2017). D-Galactose is an important precursor of bio-fuel, such as ethanol and butanol (Chen and Dixon, 2007; Ha et al., 2015; Hyungyu et al., 2015), as well as L-AHG (Yun et al., 2015b). Besides, a recent Japanese potent study showed that L-AHG could inhibit melanin production, indicating the whitening ability of L-AHG (K. Kirimura and T. Koide, Japan patent JP2013-247884). The substantial NABH genes obtained in this study can provide more choices to hydrolyze NA2 for the production of L-AHG and D-galactose. Therefore, above enzymes possess great potential in food, pharmaceutical, cosmetic, and bio-fuel industries.

Genome sequences are crucial to study metabolic mechanism and pathway in nonculturable bacteria, which comprise more than 90% of microorganisms in the world. Unfortunately, the data sizes of previous studies are insufficient to assemble genomes. Our study was the first to attempt to assemble the genomes from MGS datasets. All of these draft genomes remained unreported and exhibited many CAZyme genes and degrading ability for several polysaccharides. Among these draft genomes, clusters 1, 3, 5, and 6 were annotated as *Desulfuromonas* genus, *Treponema* genus, Ignavibacteriales order, and Polyangiaceae family. *Desulfuromonas* is closely related to sulfur reduction, acetate oxidization, and Fe(III) reduction (Pfennig and Biebl, 1976; Roden and Lovley, 1993; Coates et al., 1995). Bacteria in the *Treponema* genus are often pathogens of human and animals (Harris et al., 1972; Fraser et al., 1998; Handsfield, 2004). Ignavibacteriales order is reported with the function of CO_2_ fixation (Iino et al., 2010). Polyangiaceae family is the only family of myxobacteria that has only included cellulose-degrading strains (Garcia and Müller, 2014). Clusters 2 and 4 are potential new species, and Cluster 4 harbored complex degrading-ability for most polysaccharides, including starch, chitin, cellulose, pectin, agarose, and so on. This result indicated that several new and unknown microbial resources for the degradation of complex polysaccharides may remain unexplored in MGS microbiome. The metabolic pathway of agarose was speculated in Cluster 4. However, the enzymes for AHG metabolism that possibly include AHG dehydrogenase (*Vej*AHGD) and 3,6-anhydrogalactonate (AHGA) cycloisomerase (*Vej*ACI) (Choi et al., 2011, 2013; Yun et al., 2015a) were not detected probably because of incomplete assembly. Iverson et al. (2012) assembled the genome of an uncultured marine group II *Euryarchaeote* through an ocean water metagenomics dataset with about threefold mate-pair reads relative to that in the Global Ocean Sampling database (Rusch et al., 2007). The high coverage of database resulted in a nearly closed genome of marine group II *Euryarchaeote*. Luo et al. (2012) performed the genomic assemblies using the datasets derived from the microbial community in freshwater, soil, and *in silico* simulations. The results revealed that the genome of a single genotype or species can be accurately recovered and assembled using a 20× coverage metagenomics database from environmental samples with complex microbial community (Luo et al., 2012), suggesting that the integrity of a genome assembly relies heavily on sequencing coverage. However, even the large data size in our study only reluctantly supported the genome assembly and could not extract sufficient reads, which caused low-coverage and fragmented genomes. This result also indicates that enormous genetic resources, including polysaccharide-degrading genes, were uncovered in the MGS microbiome. We believe that with further studies, massive datasets that are similar in size to that of human gut microbiota will be generated. Using these terabase-level data, complete and valuable genomes and several undiscovered genomes and pathways in MGSs will be obtained.

In summary, this study demonstrated the advantages of MGS microbiome in agarlytic gene isolation from the aspects of the capacity, diversity, activity, novelty, signal peptide and related unculturable genomes, enlarged the routine roles of mangroves, and provided insight into the potential of MGSs as potential repertories not only of the agarlytic genes but almost all of the CAZyme family genes.

## Author Contributions

WQ and RZ designed this study. WQ and WD performed the experiences. WQ, DL, ZZ, and RZ analyzed the sequencing data. WQ, BG, and RZ wrote the paper. All the authors have read and approved the final manuscript.

## Conflict of Interest Statement

The authors declare that the research was conducted in the absence of any commercial or financial relationships that could be construed as a potential conflict of interest.

## Footnotes

1 http://www.cazy.org/

2 http://www.kegg.jp/

3 http://eggnog.embl.de/
