# Supplementary material for: Metagenomics Investigation of Agarlytic Genes and Genomes in Mangrove Sediments in China: A Potential Repertory for Carbohydrate-Active Enzymes
Source: Front Microbiol. 2018 Aug 14;9:1864. doi: 10.3389/fmicb.2018.01864 (PMC6109693; doi:10.3389/fmicb.2018.01864)
Supplement: Supplementary file 1 [file Data_Sheet_1.PDF]

Supplementary Material:

Metagenomics investigation of agarlytic genes and genomes in mangrove sediments in China: a potential repertory for carbohydrate-active enzymes

Wu Qu *, School of life sciences, Xiamen university, Xiamen, China

Dan Lin *, Novogene Bioinformatics Technology Co., Ltd., Tianjin, China

Zhouhao Zhang, Novogene Bioinformatics Technology Co., Ltd., Tianjin, China

Wenjie Di, Key Laboratory of Marine Genetic Resources, Third Institute of Oceanography, State Oceanic Administration (SOA), Xiamen, China

Boliang Gao, School of life sciences, Xiamen university, Xiamen, China

Runying Zeng **, Key Laboratory of Marine Genetic Resources, Third Institute of Oceanography, State Oceanic Administration (SOA), Xiamen, China; Key Laboratory of Marine Genetic Resources, Fujian Province, Xiamen, China

*** These authors contributed equally to this work**

****Correspondence:**

Runying Zeng

E-mail address: zeng@tio.org.cn

1 Supplementary Figures and Tables

1.1 Supplementary Figures

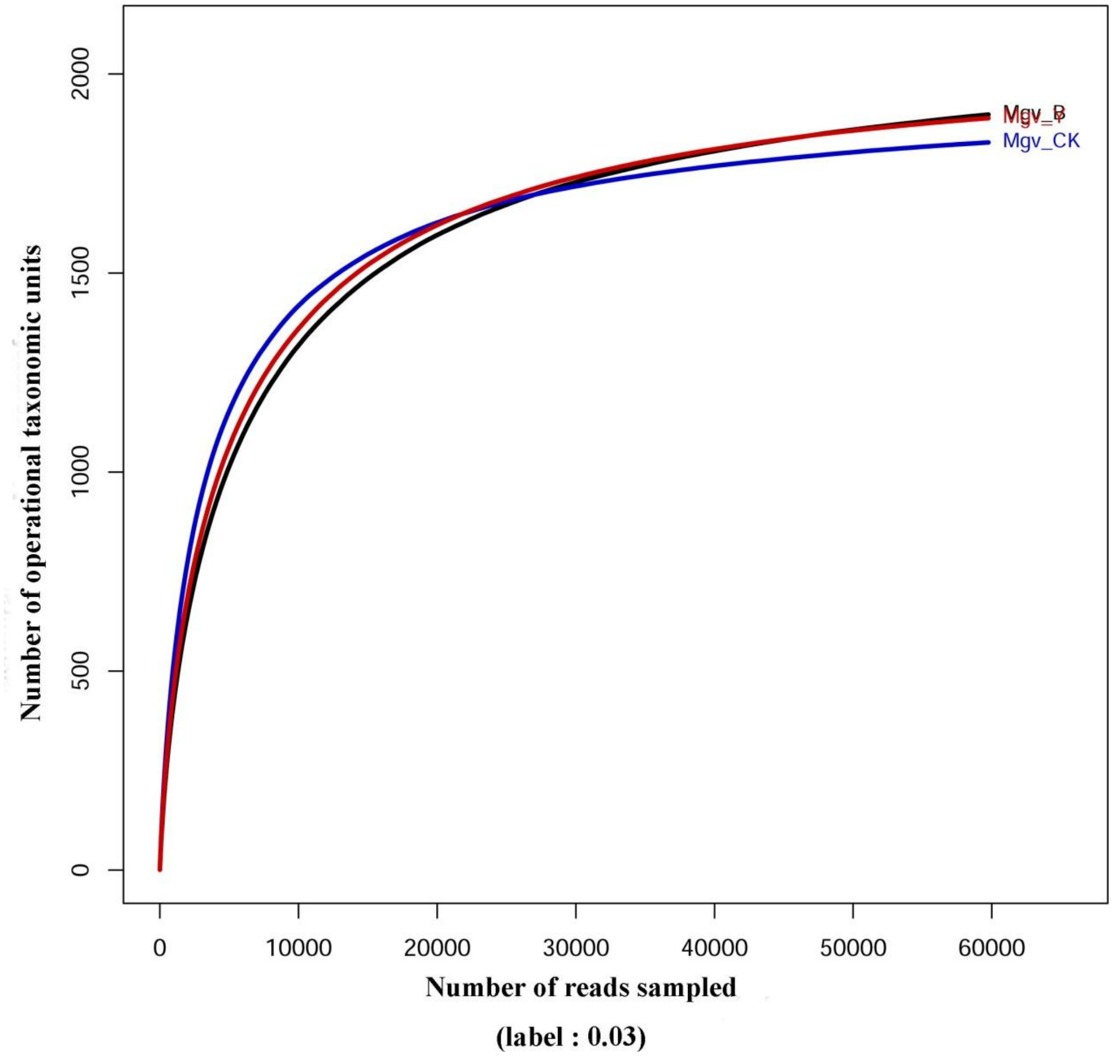

Figure S1 Rarefaction curve of 16S rRNA genes in the control and enrichment groups. The sequences that have 97 % similarity are defined as one operational taxonomic unit (OTU). The slightly increasing trend of the curve of the three samples showed that sequencing depth was sufficient to cover the most prokaryotes in the samples of mangrove sediments.

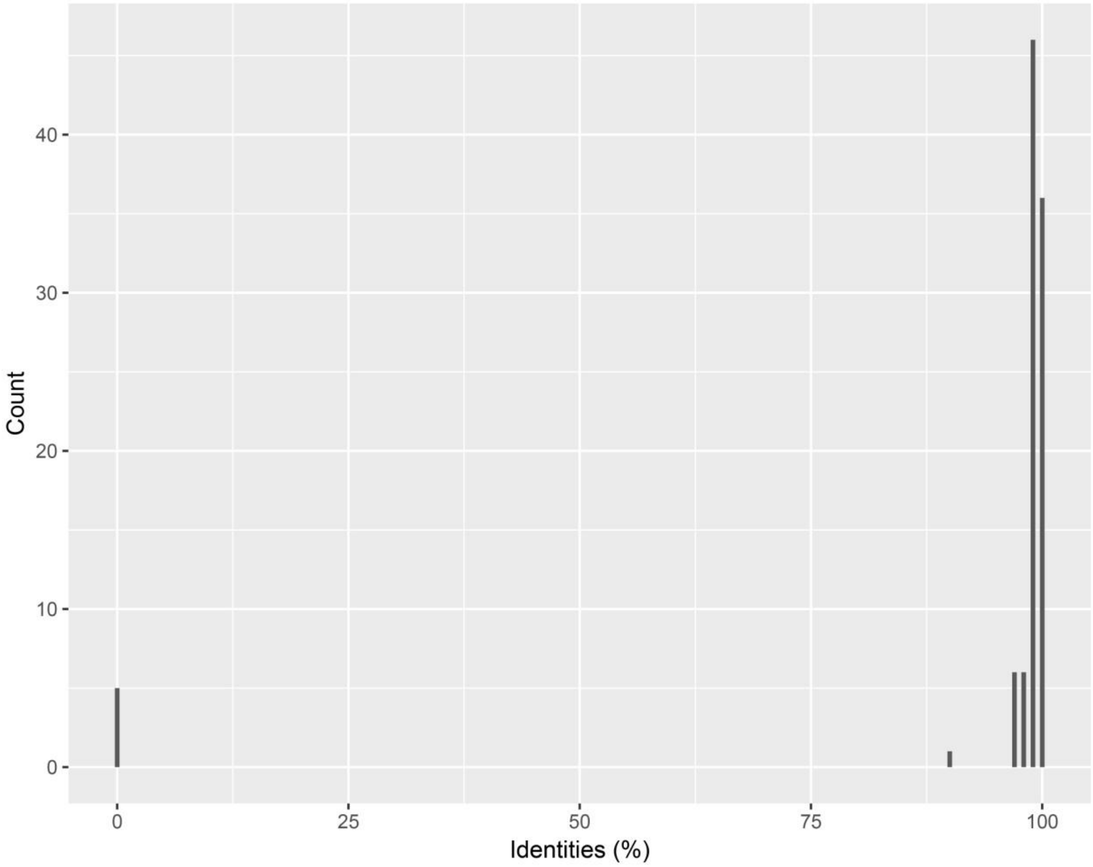

Figure S2 Accuracy examination for metagenomics sequencing and assembly. One hundred sequences were randomly selected to amplify using PCR. The sequences of the metagenomics datasets were compared with those of the PCR and sequencing. The results showed that 97 out of the 100 amplified DNA fragments were found with more than 97% identity of the original sequences, which suggested the high accuracy of the datasets in this study.

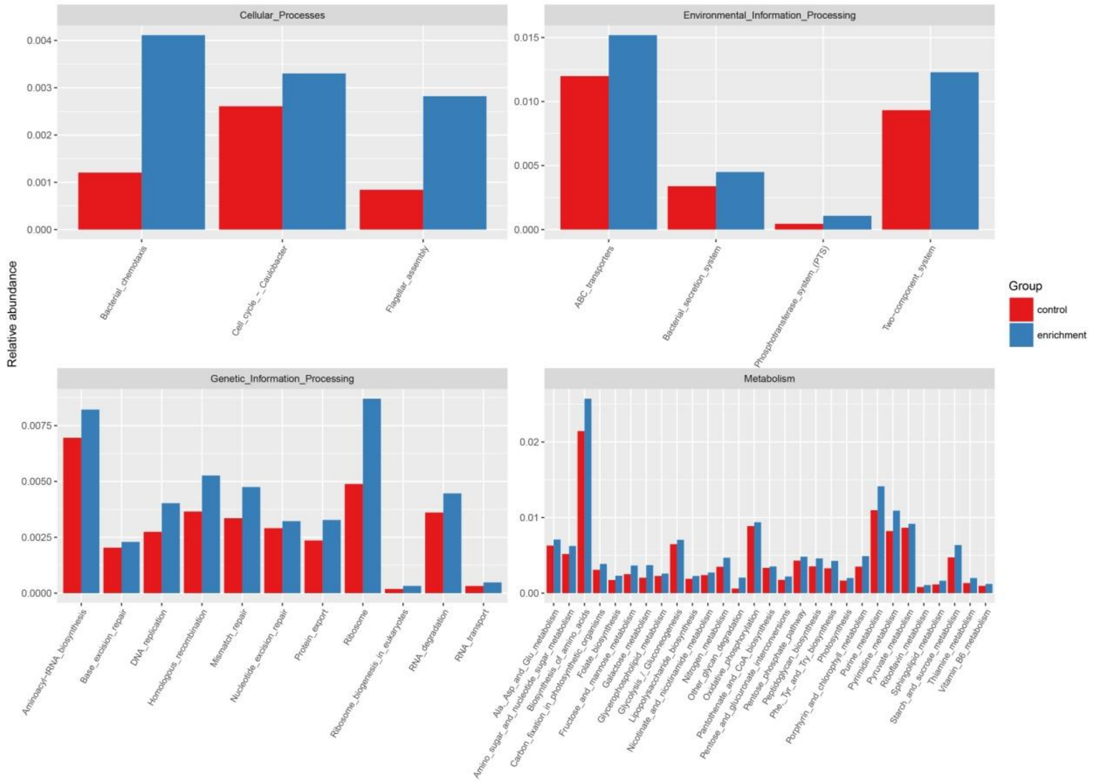

Figure S3 Abundance variation of the functional genes after agar enrichment. Subsampling was performed to the datasets for comparing at the same level. The abundance of genes, including bacterial chemotaxis, flagellar assembly, ribosome, protein export, etc, multiplied after agar enrichment.

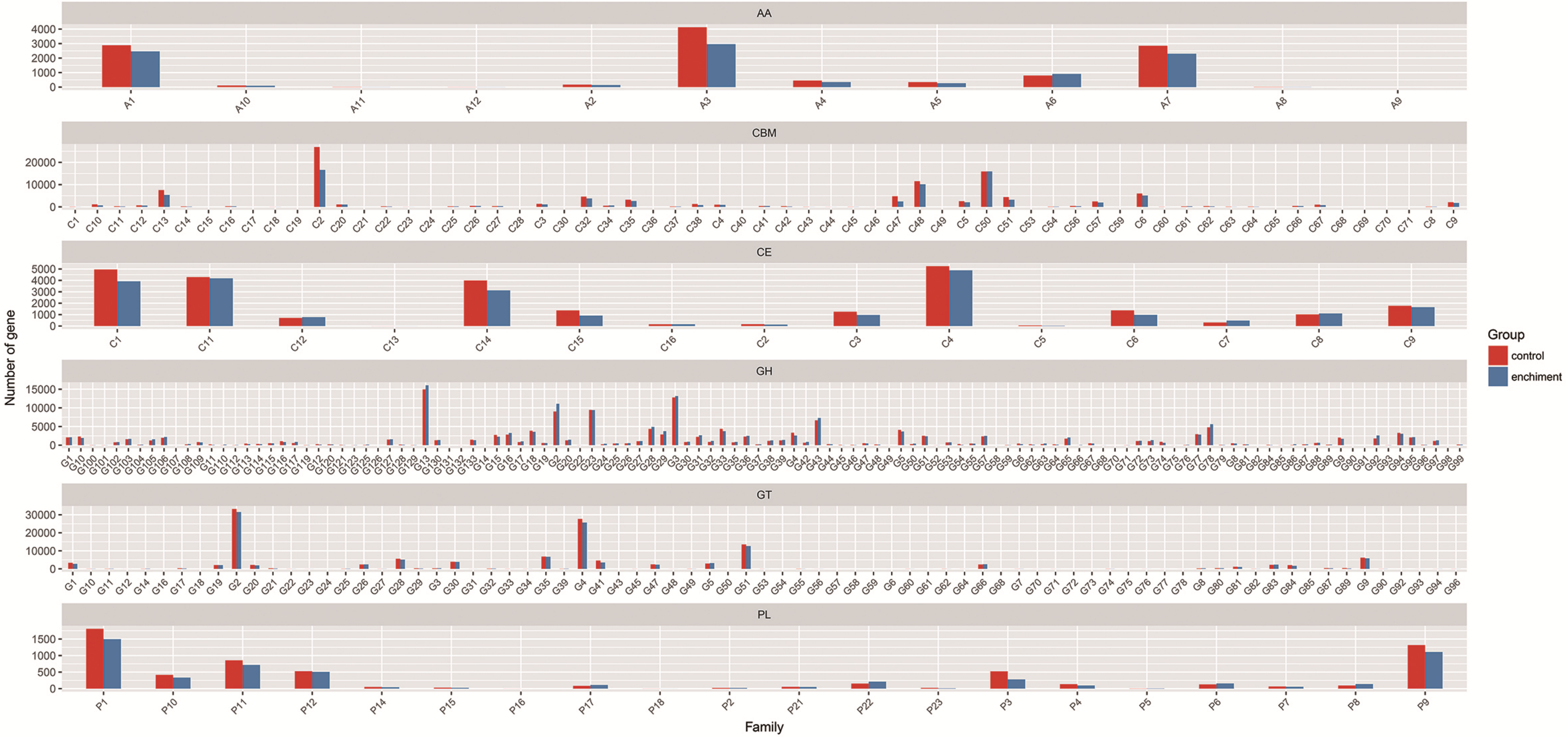

Figure S4 Number of CAZyme genes in the MGS datasets. Nearly all the CAZyme families were found in both of the datasets. After subsampling of the datasets, there was no significantly change after agar enrichment except CBM 2. The number of CAZyme gene regardless the gene completeness was shown in this figure, and the number of CNR CAZyme genes compared with other datasets were shown in Table S9.

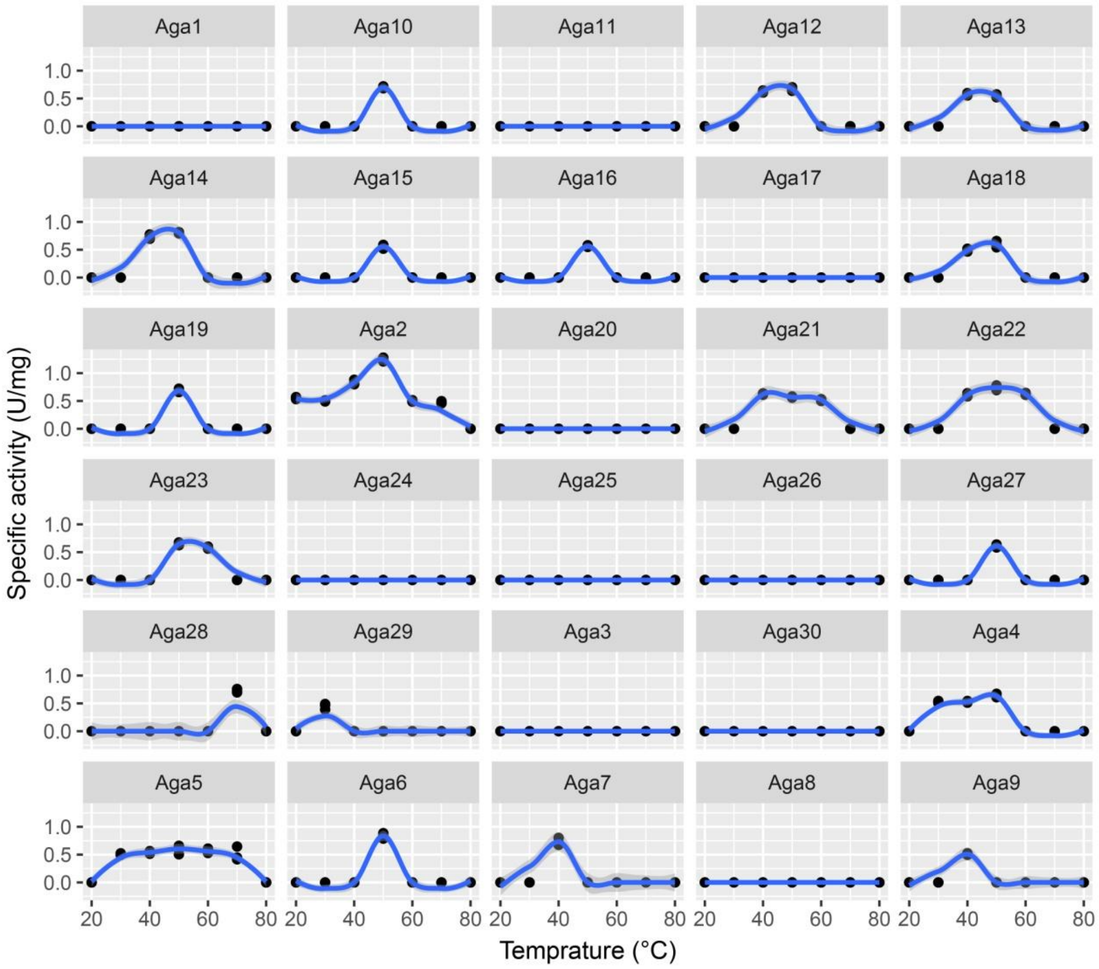

Figure S5 Temperature effect on the specific activities of selected agarases.

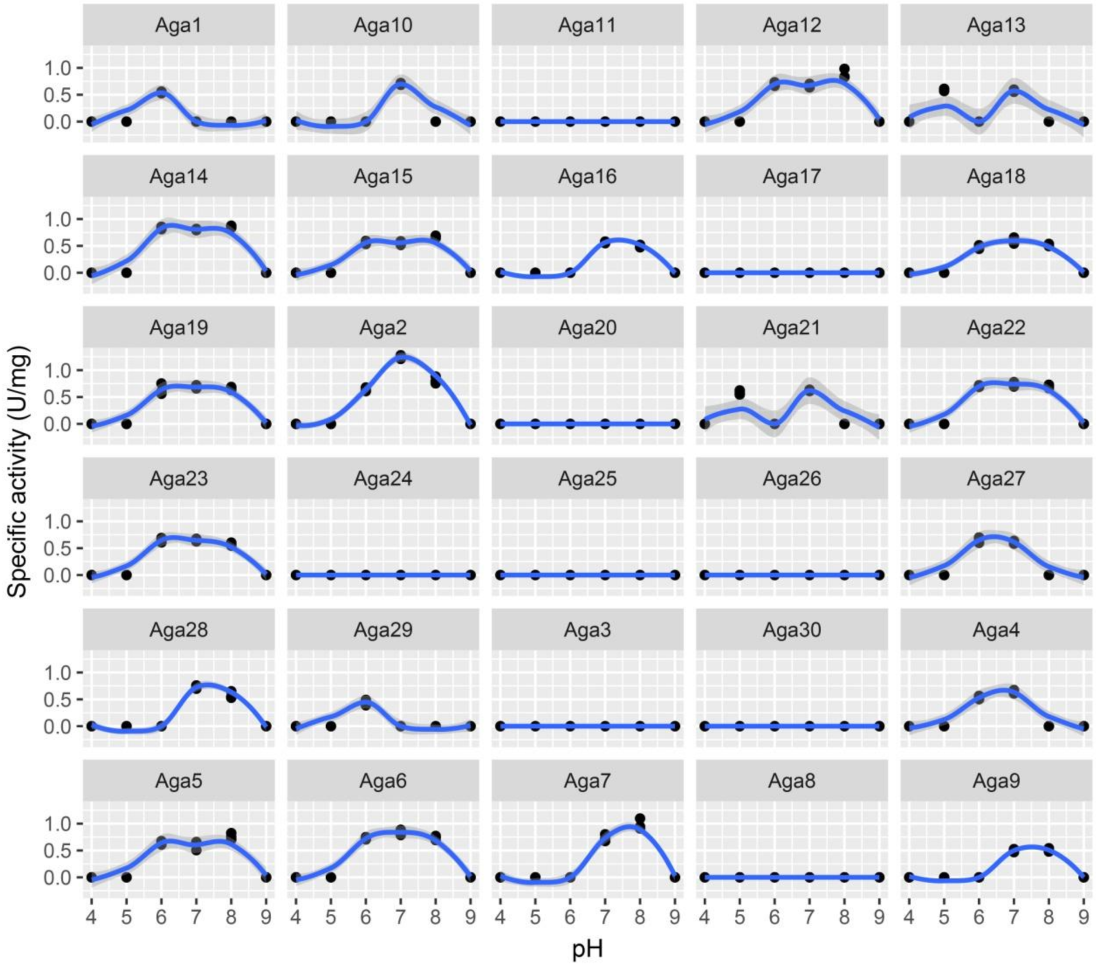

Figure S6 pH effect on the specific activities of selected agarases.

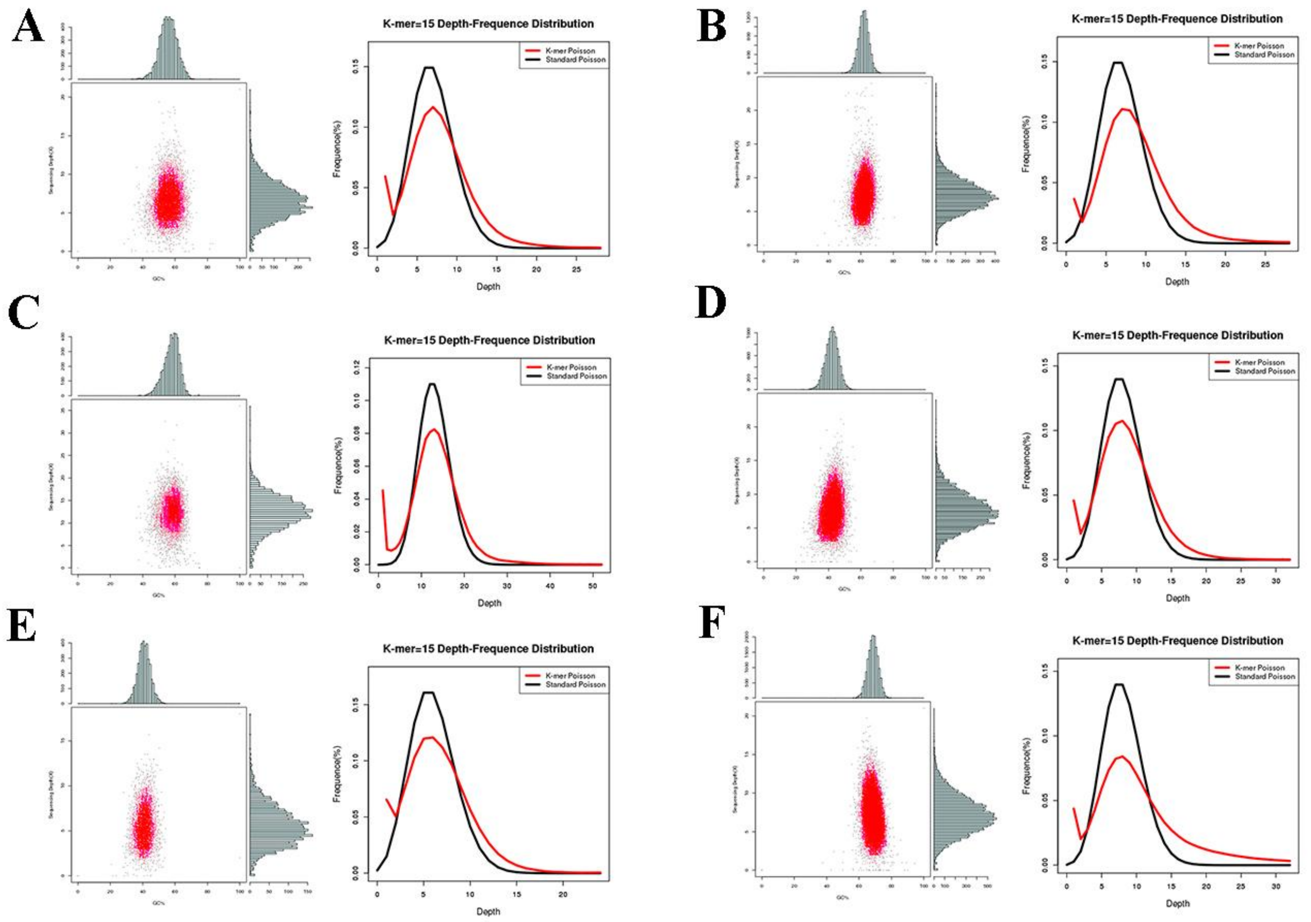

Figure S7 Accuracy assessment of the six genomes by 15-mer frequency distribution and the relationship between GC content and sequence depth. A - F represent the accuracy assessment of Cluster 1 - Cluster 6, respectively.

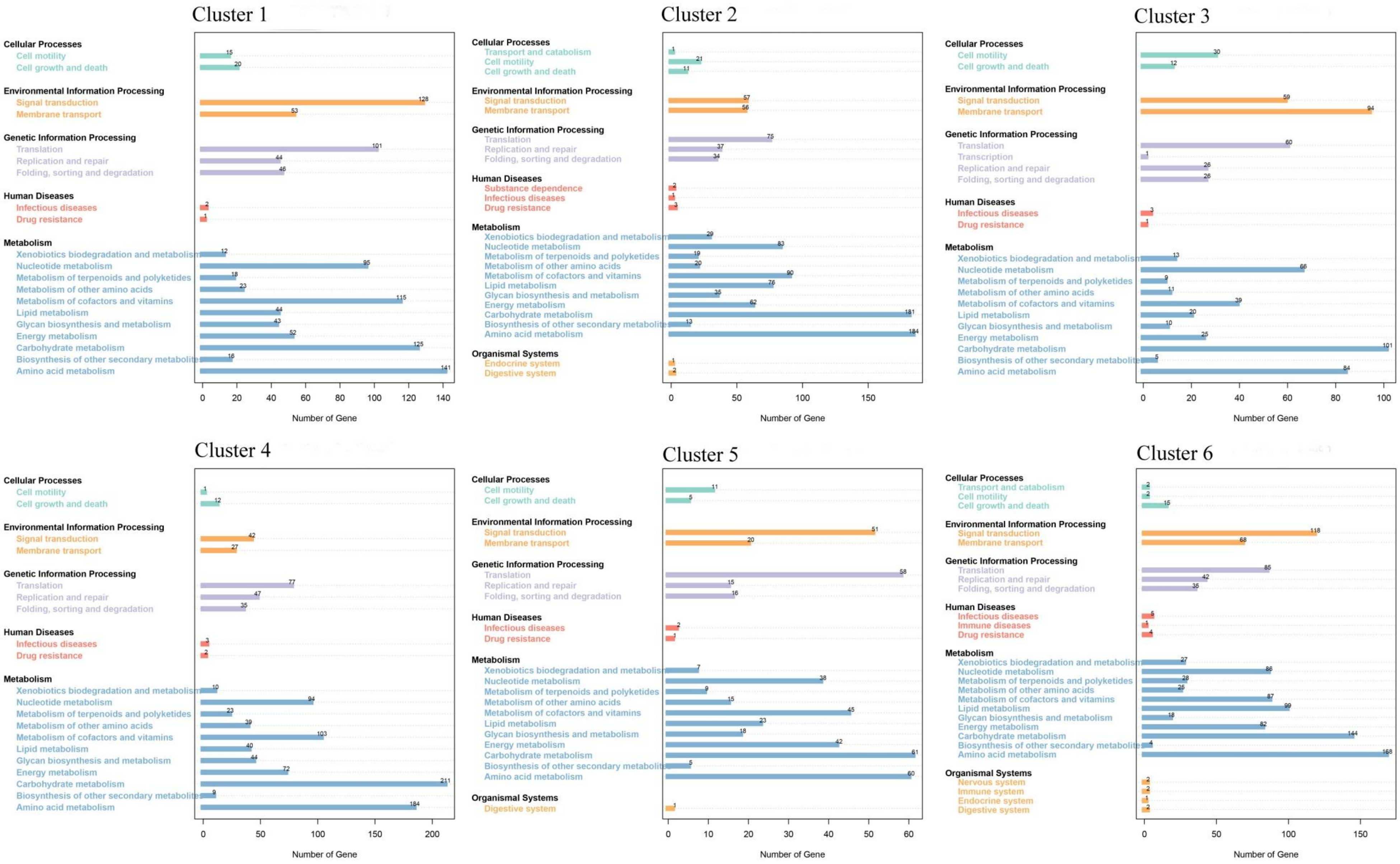

Figure S8 KEGG analysis of the 6 draft genomes. The proteins in each draft genome were annotated in KEGG database. The result showed that Cluster 4 had the most genes related to carbohydrate metabolism among these draft genomes.

1.2 Supplementary Tables

Table S1 Best dosage for 16S rRNA gene amplification

5×FastPfu Buffer	4 μL
dNTPs	2 μL
Forward Primer(5 μM)	0.8 μL
Reverse Primer(5 μM)	0.8 μL
FastPfu Polymerase	0.4 μL
BSA	0.2 μL
Template DNA	10 ng
Double distilled water	Up to 20 μL

Table S2 Best procedures for 16S rRNA gene amplification

Temperature (℃)	Time
95	3 minutes
95	30 seconds
55	30 seconds
72	45 seconds
Go to Step 2 for 27 cycles	
72	10 minutes
10	Forever

Table S3 Primers of sequence amplification for accuracy examination

enrichment group		control group	
**Number**	**Primers (5' - 3')**	**Number**	**Primers (5' - 3')**
**1**	CAATTAGCCAGCATCAGCGTAAA<br>GGTCATCCAGTCGAAACCAGTTGTA	**51**	ACCTGACCGTCGCCTTCTC<br>TCACGTCATCCGCCGAAC
**2**	GAGCGGTCAGTCCACCGAAGACGT<br>GGATCTCGGCGATTTCCTTGTAGTT	**52**	GCATACGATGTCGGTGAATTGTCG<br>GCTGCCATGTTGTGCCGTTACTC
**3**	CAAACGGGAAAACGAGCGTCTGA<br>AGCGGGAAATCACTTCGCCAATA	**53**	CCCCTTCCCGTCAAAGCCACA<br>TCCGTCGAGAACCGCATCCC
**4**	CTCCGACCGCCTGTTATTGGTAT<br>TTCGAGCAGCTTAATGGTGACTT	**54**	CGGCAATTCGGGCAGCAA<br>TCAGGACCAGGTTCTCGACATT
**5**	GTTCAGGGAAGACGCAGTTTGTGA<br>CGCCTCCTCCAAGTTCAATGCTC	**55**	ACAGGCTGCTGCGTAATGG<br>GGGTCTACCTCGTGAATGAACT
**6**	ACGTACTGGACCACGAGGTGACAAT<br>TGCTGGTAGGCTATTCCTCCTTTGC	**56**	CCCGACATTGATGGGTTGC<br>TCCAAGTCCCGCCAGAAG
**7**	TGCTGGGACCGGCTGAAGAAACT<br>ACCGGCTTGGTGACGATGTAGGTG	**57**	AAGCGAGGAAGGACGAGAA<br>GTTGAGCAGCACCATGAGC
**8**	GGCATGTTCGTCAGCGGCGATAT<br>CACCGGCAAGAAACTCACCAGGAT	**58**	GGGCTGCTGACCATCACTG<br>ACGCCGTCCACGTACCAA
**9**	ACTGAAGAAAGCAAGGACCCTGTG<br>AAAGAGCGGATCATAACCAAAGC	**59**	CCGATAGGGTAGACGCTGAC<br>CGACTTCCCAACGGATTTG
**10**	GGAGCGTCGTGACATAGTCCGTGAG<br>CTTGACAGCCTGCCAATCATCCC	**60**	CGCATCAGCTACGCCCTGTAC<br>TCGGATCTCGCCGGTTCCTC
**11**	AAGTAACGGAAGCAGACCGAATA<br>GGAATAATGAAAGCGTCCACATC	**61**	GCAATTTACGGTTCTTTCTCG<br>GCTCGGTCATGTGGTCCTG
**12**	ACACGGTCCGGCGATTCAAGCTG<br>ACGAAAGCGGCGAAAGCCAACAA	**62**	CCATACACCGCCCATCGGCACTT<br>GACGACCACTTTCCTGATTTGTCTTTC
**13**	CCCTATGACACGCCCAAGATGAAG<br>CCGGAGTTGCGGTCGTAGAACAT	**63**	CGTCGATCACCTATACCCTTCC<br>CCTGCGTGCTTGCCAGAT

Table S3 (continued) Primers of sequence amplification for accuracy examination

enrichment group		control group	
**Number**	**Primers (5' - 3')**	**Number**	**Primers (5' - 3')**
**14**	TGCCGGTGAATCTGACCTAGTCG<br>GTCCCATCAGCCAAAGTCCCAGT	**64**	GAGCCGCCTTACAACCTCA<br>CCAACCTTGGCAAGACCC
**15**	TTTTAATCTTCCTGCAACACTTTGG<br>CAATTATTACACGACTAACCACGACAT	**65**	TACACCTCCGGGCGCATCCA<br>AGCAGGGACTGCGGGAACAC
**16**	AGCAGGACAGTTCCTCAATCCAA<br>GCTGTAATCGGTCAACTCCCAAG	**66**	CTGAGCCCTCTATCTGGAAACTG<br>CATAGCTCTGACCGCCTGAGTA
**17**	CCGCTGGTTACCTACGTTTTCAGTT<br>TCGTGTCCCGGAAGAGTTTATCG	**67**	CCGTCTTCCCGCAAACCA<br>ACGCTGCCGGAGCTGTTG
**18**	ATGCTGTCTTCTTAGCCCGTGAG<br>GATTTGCAGTTTGGCTTGTTTCA	**68**	GCTACTACGATGTAGGCGAACC<br>TTGAATCCGAGGGACTGAATG
**19**	ATCGAACTGTTCCATACCCCGAATACC<br>GCTGTGCAGTACGATCTCGAAACCC	**69**	CCTCGGAGCTTCCATCTTACC<br>GAGAATGTCATAGCCGTTGTCG
**20**	TCTACCACGCCTTCGGCAACAAG<br>TCGAGATCCTGCGGCAATTCACC	**70**	ACCGGGAAGAGTACGAGGGC<br>AGAATCAGGTGGAACGGATGG
**21**	ATTATCATCATCGACGACGAACCC<br>CAAACTGCCTTTGAATCGACTGC	**71**	GGCAAGCCCTACGTGGTCAG<br>ATCGGTGTCGTCACAGGATGGT
**22**	GACTTGGAGGTTGTCGGCGAGGCG<br>CGGTCTTCAGAATCTGAGGCTTTTCC	**72**	GATGGGAGCGGCAATCTGGT<br>CGGATGGAGTCTTGGTGGAGTAG
**23**	CGACCCGCCAGGTGCTAGAGGAGAA<br>ATTCCAGCGGACGCAGGAAGACA	**73**	CGGCAATACATCGAATACACCTC<br>GTAATGCGGCTCATGGAACG
**24**	CCCTGGACCAGATGACCCAGCAA<br>GAACCTCAGGTCGAGCTTCTCCTCCTT	**74**	TATTACACCTCTGCGAAATTATGG<br>TCAGCGTATAGTATGGTTCTCCA
**25**	CCGATTCCTACCGCATCATCTGGG<br>GCATCATCCCGTCGCCCTTGA	**75**	GATCTCAACCTCACGGGCAATCTC<br>GCCGATCAGCGTGTCGTTCC
**26**	CTGGGCCTGTTCGTCAGCCTG<br>CGAGACCAGCAGCGTGGAGGC	**76**	GATCTCAACCTCACGGGCAATCTC<br>CGATCAGCGTGTCGTTCCCAC
**27**	TGGCAGGACATTCCAGCTTTATG<br>GACGCAGCAGCATGTGGTACTCT	**77**	GCTGTTCGTGGTCGGCAATG<br>GCGGAGCGCACCATGTAGGT

Table S3 (continued) Primers of sequence amplification for accuracy examination

enrichment group		control group	
**Number**	**Primers (5' - 3')**	**Number**	**Primers (5' - 3')**
**28**	GAAACCTTCGCCCGCCTCAAC<br>CGACCCGACGATATTCCGCACC	**78**	GCTTATTATGCAGATAACCGGAGTA<br>AACCAGAAGGCACTTGAACACC
**29**	AAGGCACGCGGTGAAGAGGAG<br>GATGTAGTCGAGCAGACGCTGGTAA	**79**	AGGTATGCGCCATTTCGATGTTT<br>CCAGGAAGCCGATATTGTAGTTCTCA
**30**	CAGTGGTTTGATGGAGGAGATGC<br>AAGCGATTGTCTTTGGTCTTGTAGG	**80**	AAGCGTCACCATTCAGCC<br>GTTCCAGCCAGGTCTCGTC
**31**	GATGCCGCAGGACAGAATAAGTT<br>TTCAACAGCTCCATCTCCCAATC	**81**	TGGGCAGACCAGGACATAGGA<br>GCATGGGAAGTCACGCACAAT
**32**	AGGACGAGCAGGGCATCAAAGC<br>GCCGAAAGGGACTGGGAGAAAA	**82**	CTGGTTTCTTTCGTCTTGATGTTG<br>TTGCCCTCGCGTACTATCTTTAC
**33**	CATCGTCCGTCTGCCCGTCTT<br>CGTTGGTCTTGAGGCTCGTGGT	**83**	GGTTTCTTTCGTCTTGATGTTGTCT<br>TTGCCCTCGCGTACTATCTTTAC
**34**	CGGAATCGTCGAAAGGGAGGC<br>CGTGAGCAGCAGCAGGTTCAGGTA	**84**	GCACCTACGACGTGCTCTTGT<br>CAGTCGCAGATCGGCTAATGT
**35**	GCCTATCGGGCACGACTTCAA<br>GAGCACTTCCGCTTCGTCCAT	**85**	GGTCTACAGCCGCGACATG<br>AGCCGACCGTTCGCATCAC
**36**	CCCCAGTACGGCAACATCTTCGA<br>GAGGCGTTCATCACCCAGTCCC	**86**	AAGCCGTATCGCGGAGTC<br>TGGTCGGAGCGGTAGTGTC
**37**	GGGTGGACATGAACAAGGGAACG<br>AGCCGATGAACGGAGGGTTGC	**87**	CGCCTCCAGAAACCTCACC<br>ATCACCAGCGGGAACACC
**38**	GGAGTTGAATACACGGAGACTTTGC<br>TGATGCCGCTGCCATACCAAT	**88**	TACCACCGCCTCCAGAAAC<br>ATCACCAGCGGGAACACC
**39**	GCACCACCTACGGCTACCACA<br>CAGCAACACCGAGTCGGACAG	**89**	CGCCTCCAGAAACCTCACC<br>TCACCAGCGGGAACACCAG
**40**	CGAAGCGTCGGGCAATATGAT<br>ACCGCCTCGACAATGGTGTTC	**90**	ACCGCCTCCAGAAACCTCA<br>ATCACCAGCGGGAACACC
**41**	GGGTGCTGCTGATTACCGATGC<br>CCGGAACCTGCTCCTCCTGAT	**91**	TCTGGAATGCTCATCGTCCTC<br>CAGTTGCCGCACCTGAAATAA

Table S3 (continued) Primers of sequence amplification for accuracy examination

enrichment group		control group	
**Number**	**Primers (5' - 3')**	**Number**	**Primers (5' - 3')**
**42**	GAAGCCTGACGCGGTGGTGAT<br>GGCGACAACGAGGTTGGAGAT	**92**	CAAGAAGCACCTTGAGACGGAATA<br>TGCCAGAGGCGGATGTAGTCG
**43**	GGAACTGGGTGGTGCATTGGC<br>CGCTTCGTCACGACACGGTAGGT	**93**	TGTTACTCACAAATCCCTGGTTGA<br>TGCCATCGGTATCCGGCTTAT
**44**	AAGTTTGAGACCATTGGCTTTGA<br>TGGGTTTATTCTTTCTGGGCTGT	**94**	GTCGAGGTCTTCTTCGTCATCAGTG<br>CCGTAATAGAGGCGGGAGGTGT
**45**	CCCCGTTAAGAAAGGAGACCTGA<br>ATCAACAGACATACCGCCAGAGG	**95**	GCAACGGATTCACGCAACA<br>TGACCAGCGGGAACAAGG
**46**	GGGATCGCTTCGGAATATGTCGG<br>CGCAGGCGCTCATCATGTTGTAT	**96**	CGGAAGGGCGGTATGCTA<br>CGGAAGGGCGGTATGCTA
**47**	TCAACAACACCACCTACGGTATGAC<br>TAGACGCCCAACGGGAAGTAA	**97**	GCCCGCATCAACACGAAG<br>CACCGCGAGGTTCAGGATC
**48**	GCTTATGGCGTCGAGAAACGG<br>CCGGCAGAACACCGGAAAGTG	**98**	GAGCTGGAATGTCGAGGTGATG<br>GGCGGTGATCCGGTTGTT
**49**	ATACGCTGAAACACTCAAGCAAGGA<br>GTTGCCCGTAACCTACATCCACA	**99**	CCTGGCTTGGACTTGGGATG<br>GGCTGTAGTGAGGGTGCGAAT
**50**	GTTCAGGGAAGACGCAGTTTGTGA<br>CGCCTCCTCCAAGTTCAATGCTC	**100**	TTGCATTGGAATGGAAGGAGGAA<br>TATTATAGATGCCCGCCGAAACG

Table S4 Primers for agarase gene amplification

Name	Forward Primer (5'-3')	Reverse Primer (5'-3')
Aga1	CAATACGACTGGGATAACATTGCAATTCC	TAGCTTTGTTAGTTTGCGAGTGACCC
Aga2	CAAAAACATGAATGGGATAAATTTCCTCTTCC	TTTCTTCTTATCAACTAGGTCATAAACCCTTATCC
Aga3	CAAAAACTTATTTGGGAAGAACAGTTTAATGGAG	TTGATATACCTTAATGTAATCTATAACGTATTCTTGTGG
Aga4	TTGTTTTCCAGTGAAGAAGCTGGG	CCCCCTTGATTTTTTGAAAACCCG
Aga5	AATGCCAGTTTCGATGGAAGTCC	TTTTACGAATTTGAAGGACTTTGATATATTATTTTGTTTAATCAG
Aga6	GCCAATGACTGGGACGGAATTGCAATTC	TTGGATGATTAATTTATGAGTTTCAATGCCTTGC
Aga7	TCATGTCAAGAAGAGGAACTAATTACGGAGC	TATACGTGTGTATGTTTTAGTAGATTTATTGATTTGACC
Aga8	GCTCCCCTGCTGCCCG	TTTCCTCTTCTGCCAGACTCTGACATATTC
Aga9	CATGAATGGGATAATTATCCGGTGCCTG	TTTTACCAACTTCTTAATTGTCCAATTTCCATCC
Aga10	AAACTTATTGTTAAAATAATTTTATATGTAATAGCATCAAGCGG	TCTCATTAAAACCATCTTTTTAGTTTTTACAAATGATTGATTTTC
Aga11	GGCACTCAACCCGAAGATATGAGTCTTG	GTAAATCTCATCGGAATACCAGGCAACG
Aga12	AATTATACAGGAAGAGCGGTAGGGCG	GCGCGCTTTACCCCGGC
Aga13	AACGCGAGTATCGATCTTTCGTCCCTC	GTAGGGGCTTCCGGCGGTG
Aga14	GTTTGTGATTTAAGGAGCACCATTTTTCAC	TTTAACTTTAGCGGTATTACGGATTTCGTAC
Aga15	GAGGATATGGCAAATGCGGAGGCTG	CTTCATCCTGTTTTCAAACGTGCCTCTG
Aga16	GCAGATGTTGCAAATGAAGTGATGCTTTTTG	ATGTGTATCTCGATACTCATACATATCATAACAGG
Aga17	AATTTATTGAAACATTATGATTTTACGCGCATGAC	TTGATATACTTTTACTTCCTTAATGATAAATTCTTGTGGAAAC
Aga18	AAGGAAATCTTACAGATAAAGGAACAACAAATGATTAATGGTTC	ATAATGAATCGGTGGAACATCCTCTCCTTTGTCATTTG
Aga19	GACCCCTTGCTCCTCTGGCC	TCGTTTACCGAAACGCCGCTC
Aga20	TCACCAAAAACATTTAAAATTCAATTTGATAGCAGTAAAG	TTCATTCCACAGATTTGGAATTCCTCCGTG
Aga21	GGCAACGATGCCTGCCTGTTTG	GTGGACCGTCCGGATCTGATAGAG
Aga22	CAACCTTGGACTAACAAATCAGCGAAG	CTCATCTGAGACAATCGTGATGGGAGATG
Aga23	CAACCGGTAGTTGTGGAAGTTAATCTTAACG	ATGCTGAATTAAAACTCTCACTCTTACAATTTTATTATTGC
Aga24	AATATTACAAATATAAAACACCATATTATGAAACACCTCTTTTTGC	ATCTTCGAATGACCTTTTAATATCTGTACTAAAGTTAAATACTCGC
Aga25	CAAACGAATGTAGATGTTAATTTAAACATCAAACATTCGGTGG	TTGTTTAATAAATACTTCGGTATGAATAGTTGTACCATTTGTAATTTG

Table S4 (continued) Primers for agarase gene amplification

Name	Forward Primer (5'-3')	Reverse Primer (5'-3')
Aga26	AAATATATTTTATTTATAGTTCTTAATTTTTATACCATAAACATAGTTCGTTCGC	ATTTTCCTGAATATCAAATACTTGTAAAGCAACACTACTAATAAAACCACC
Aga27	CAAAAGCGAACTGCCGTAATATTTTTTGTGGG	TTTCTTAAGCTTCACAAGCTTTCGGCTGC
Aga28	AGAAGTTTTTTGACTTTCCTGCTGATGATTACTG	TTCCATTACATTTATTTTAAAAGATTTATTCGAGTTTTCGCCC
Aga29	ATGACTGATTCCAACCCAACGGC	TTTCTCTATTCGGTTTACCTGCAGCAC
Aga30	CAGGATATTTACATTGATGTAAATTTGAATGTAGAACATAAAATTGATAC	ATGTTGTATAAAAAGTTTGGTAACGGCTTTGTTCGAATTATTG

Table S5 Shifting of 16S rRNA gene diversity in MDSs after agar enrichment

Genus Name	Abundance (Mgv-B/CK/Y)	GH Family
Acidaminococcus	178/3/61	3, 13, 18, 23, 73, 77
Aeromonas	244/20/1	1, 2, 3, 5, 8, 9, 13, 18, 19, 20, 23, 32, 36, 63, 73, 77, 84, 94, 102, 103
Arcobacter	267/20/7	3, 17, 23, 102
Bacteroides	1015/58/901	2, 3, 10, 13, 15, 16, 18, 20, 23, 24, 25, 27, 28, 29, 30, 31, 32, 33, 35, 36, 39, 42, 43, 51, 53, 57, 63, 67, 73, 77, 78, 84, 88, 89, 92, 95, 97, 105, 106, 108, 109, 110, 115, 117, 123, 127, 130, 133, NC
Cytophagaceae_uncultured	8021/1006/1935	1, 3, 5, 8, 9, 10, 11, 13, 15, 16, 23, 26, 30, 31, 43, 57, 73, 74, 77, NC
Enterobacteriaceae_unclassified	1314/8/13	1, 2, 3, 4, 5, 8, 13, 20, 23, 24, 28, 31, 36, 38, 43, 65, 73, 77, 88, 102, 103, 104, 105, 127
Leptospiraceae_uncultured	630/180/115	2, 3, 5, 13, 15, 18, 23, 31, 36, 42, 57, 77, 94, 102
Rhodocyclaceae_uncultured	365/30/187	13, 23, 24, 57, 94, 102, 103, 105
Ruminococcus	2383/4/51	2, 3, 4, 5, 8, 9, 10, 11, 13, 16, 23, 25, 26, 27, 28, 30, 31, 36, 39, 43, 44, 48, 51, 53, 67, 73, 74, 77, 94, 98, 105, 113, 124, 130, NC
Treponema	1430/0/10	1, 2, 3, 4, 5, 10, 13, 16, 18, 23, 29, 30, 31, 36, 38, 39, 42, 43, 51, 57, 63, 73, 77, 78, 109, 116, 127, NC

Table S6 Overview of sequencing

Sample	InsertSize(bp)	SeqStrategy	RawData	CleanData	Effective(%)
Mgv-B	350	(150:150)	135,810.50	135,490.93	99.765
Mgv-CK	350	(150:150)	129,764.80	129,337.26	99.67
Mgv-B-L6000	6000	(150:150)	40,865.32	40,665.56	99.49

Table S7 Statistics of gene catalogue

	Mgv-CK	Mgv-B
**ORFs NO.**	13,049,096	12,123,391
**integrity:end**	3,313,299(25.39%)	4,060,572(33.49%)
**integrity:all**	2,963,924(22.71%)	3,033,802(25.02%)
**integrity:none**	2,337,956(17.92%)	3,123,477(25.76%)
**integrity:start**	4,433,917(33.98%)	1,905,540(15.72%)
**Total Len.(Mbp)**	7,494.8	7,019.53
**Average Len.(bp)**	574.35	579.01
**GC percent**	62.63	58.92

Table S8 Number of gene annotated in MGSs datasets from different CAZy families

CAZy family	Description	Number	Group
AA	Auxiliary Activities	9505	enrichment
		11776	control
CBM	Carbohydrate-Binding Modules	78710	enrichment
		105142	control
CE	Carbohydrate Esterases	23238	enrichment
		26676	control
GH	Glycoside Hydrolases	156934	enrichment
		150993	control
GT	GlycosylTransferases	119819	enrichment
		128994	control
PL	Polysaccharide Lyases	5370	enrichment
		6301	control

Table S9 Comparison on the number of GH genes from different datasets. Only the number of CNR genes in the datasets of Mgv-CK and Mgv-B were shown. The gene completeness of other datasets was not especially emphasized in paper. The amount of GH genes in this study was several times higher than that from former MGSs metagenomics studies. The GH genes that failed to detect in the datasets of termite gut and rumen microbiome were found abundant in MGS datasets of this study. In summary, although only CNR genes were involved in analysis, the diversity and abundance of GH genes from this study had clear advantages.

CAZy Family	Number of gene						
	MGSs datasets in this study		MGSs datasets in previous studies		Termite Gut	Rumen Microbiome	CAZy Database
	Mgv-CK	Mgv-B	BA	RJ			
GH1	176	230	54	60	22	253	11885
GH2	380	673	36	35	23	1436	7552
GH3	1121	1358	151	127	69	2844	12001
GH4	128	154	39	52	14	215	4935
GH5	305	400	35	29	56	1451	8093
GH6	34	12	0	3	0	0	871
GH7	0	0	0	0	0	15	5081
GH8	31	35	11	4	5	329	2126
GH9	111	132	12	9	9	795	2146
GH10	202	166	31	21	46	1025	2534
GH11	21	12	1	0	14	165	1227
GH12	26	10	3	1	0	0	638
GH13	1184	1606	288	322	48	3442	37142
GH14	0	3	0	0	0	0	469
GH15	219	185	57	20	0	2	3064
GH16	204	409	10	30	1	483	4847
GH17	71	98	5	6	0	1	2109
GH18	265	400	12	6	17	364	10431
GH19	52	85	3	1	0	7	3635
GH20	81	133	35	28	15	765	3050
GH21	0	0	0	0	0	0	0

GH22	2	3	0	0	0	8	665
GH23	1330	1563	0	0	52	0	28259
GH24	40	100	1	8	0	67	4908
GH25	60	91	9	11	1	508	3694
GH26	39	72	9	10	15	369	1130
GH27	96	124	40	9	4	1114	934
GH28	308	461	17	17	6	472	4103
GH29	213	370	71	77	0	939	1649
GH30	78	125	6	19	0	209	1216
GH31	114	185	60	80	26	1382	5237
GH32	50	117	21	10	0	639	4803
GH33	367	340	53	86	0	1217	2491
GH34	0	0	0	0	0	0	69055
GH35	44	85	6	8	3	158	1499
GH36	791	811	0	0	5 to 7	0	2391
GH37	29	33	11	8	0	127	2834
GH38	32	61	41	54	11	272	2501
GH39	80	100	8	5	3	315	1237
GH40	0	0	0	0	0	0	0
GH41	0	0	0	0	0	0	0
GH42	29	77	47	50	24	374	1636
GH43	528	867	49	34	16	0	6317
GH44	11	12	2	1	6	0	120
GH45	10	6	0	1	4	115	360
GH46	11	13	1	0	0	0	338
GH47	115	122	2	1	0	0	891
GH48	4	9	0	0	0	3	906
GH49	0	0	0	0	0	5	23
GH50	17	36	0	0	0	0	341
GH51	163	195	18	18	18 to 19	0	1748

GH52	0	2	0	0	3	0	80
GH53	82	104	9	11	12	0	837
GH54	19	6	0	0	0	0	140
GH55	15	21	0	0	0	0	395
GH56	0	0	0	0	0	0	213
GH57	157	205	59	92	17	374	1607
GH58	0	0	0	0	1	0	41
GH59	2	2	0	0	0	9	85
GH60	0	0	0	0	0	0	0
GH61	0	0	0	0	0	0	0
GH62	19	10	1	2	0	1	210
GH63	21	61	9	14	0	28	1354
GH64	6	6	0	0	0	0	259
GH65	174	232	55	36	6	125	2474
GH66	0	13	0	0	0	0	113
GH67	41	36	4	4	10	120	339
GH68	0	2	0	0	0	0	530
GH69	0	0	0	0	0	0	0
GH70	0	0	0	1	0	7	321
GH71	2	4	0	1	0	2	119
GH72	171	216	0	1	0	5	798
GH73	171	293	16	16	0	393	7944
GH74	21	28	0	0	7	0	281
GH75	0	5	0	0	0	0	122
GH76	1	228	2	3	0	71	1104
GH77	214	382	70	92	14	943	3794
GH78	313	12	75	46	0	1260	1162
GH79	5	0	1	0	0	5	253
GH80	0	0	0	0	0	0	16
GH81	12	10	1	0	0	12	576

GH82	0	0	0	0	0	0	28
GH83	0	0	0	0	0	0	1409
GH84	6	6	0	0	0	0	436
GH85	1	2	0	0	0	2	346
GH86	3	25	0	0	0	0	73
GH87	6	11	0	0	0	0	259
GH88	70	83	20	21	9	527	943
GH89	2	11	3	1	0	251	232
GH90	0	1	0	0	0	0	141
GH91	0	2	0	0	1	0	103
GH92	59	135	0	0	2	0	2006
GH93	0	1	0	0	0	0	113
GH94	93	115	0	0	68 to 132	0	1353
GH95	101	123	0	0	12 to 31	0	903
GH96	4	3	0	0	0	0	4
GH97	76	117	0	0	0	0	654
GH98	0	1	0	0	1	0	97
GH99	10	20	0	0	0	0	91
GH100	6	4	0	0	0	0	259
GH101	1	1	0	0	0	0	198
GH102	106	133	11	8	0	13	2592
GH103	243	260	0	0	3	0	4655
GH104	23	43	1	8	see GH24	see GH24	716
GH105	107	219	0	0	see GH88	0	1717
GH106	64	80	0	0	2	0	223
GH107	0	1	0	0	0	0	8
GH108	38	81	0	0	0	0	1702
GH109	57	65	0	0	3 to 5	0	312
GH110	2	16	0	0	0	0	114
GH111	0	0	0	0	0	0	8

GH112	0	11	0	0	0	0	155
GH113	35	18	0	0	0	0	236
GH114	34	52	0	0	0	0	300
GH115	22	26	0	0	0	0	422
GH116	24	32	0	0	0	0	300
GH117	34	116	0	0	0	0	106
GH118	0	0	0	0	0	0	8
GH119	1	2	0	0	0	0	12
GH120	6	7	0	0	0	0	90
GH121	1	1	0	0	0	0	99
GH122	0	0	0	0	0	0	56
GH123	2	6	0	0	0	0	93
GH124	0	0	0	0	0	0	5
GH125	8	18	0	0	0	0	804
GH126	0	3	0	0	0	0	388
GH127	80	104	0	0	0	0	1066
GH128	15	15	0	0	0	0	209
GH129	2	3	0	0	0	0	54
GH130	146	238	0	0	0	0	1071
GH131	0	0	0	0	0	0	36
GH132	1	0	0	0	0	0	620
GH133	88	90	0	0	0	0	400
GH134	0	0	0	0	0	0	136
GH135	0	0	0	0	0	0	225

Table S10 Genome assembly from mangrove dataset

	Cluster 1		Cluster 2		Cluster 3		Cluster 4		Cluster 5		Cluster 6	
	Scaffold	Contig	Scaffold	Contig	Scaffold	Contig	Scaffold	Contig	Scaffold	Contig	Scaffold	Contig
Total Num(>500bp)	528	757	640	849	181	299	814	1,099	489	634	959	1,430
Total Length(bp)	3,470,006	3,320,530	5,472,370	5,372,039	2,477,813	2,378,516	5,436,698	5,260,611	2,112,993	1,991,077	8,552,982	8,305,383
N50 Length(bp)	13,337	5,545	21,058	10,586	27,318	12,346	16,867	6,707	7,856	3,747	18,716	8,528
N90 Length(bp)	2,644	2,444	2,696	2,607	5,972	3,423	2,435	2,387	2,000	1,887	3,428	2,823
Max Length(bp)	65,173	27,421	165,069	61,318	79,231	49,842	120,513	44,275	31,554	19,931	99,422	47,780
Min Length(bp)	501	501	503	503	503	503	501	288	502	371	501	501
Sequence GC(%)	55.89	55.89	61.69	61.69	57.72	57.72	42.23	42.23	40.89	40.89	68.64	68.64

Table S11 Genomic information of the six clusters

Cluster	Genome size(bp)	Gene number(#)	Gene total length(bp)	Gene average length(bp)	Gene length/Genome(%)	Species annotation
1	3,470,006	2,584	2,301,297	891	66.32	*Desulfuromonas* sp.
2	5,472,370	3,931	3,935,349	1,001	71.91	NA
3	2,477,813	2,015	1,916,280	951	77.34	*Treponema* sp.
4	5,436,698	3,670	3,646,575	994	67.07	NA
5	2,112,993	1,387	1,152,795	831	54.56	*Ignavibacteriales* spp.
6	8,552,982	5,859	6,211,239	1,060	72.62	*Polyangiaceae* spp.

Table S12 Agarases in the six clusters derived from mangrove datasets

Cluster	Gene name	GH family	Location	Completeness
4	4_177	GH 16	Cytoplasm	Complete
	4_992	GH 16	Cytoplasm	Complete
	4_2270	GH 50	Cytoplasm	Complete
	4_4386	GH 16	Cytoplasm	Complete
	4_4450	GH 16	Cytoplasm	Complete
	4_4608	GH 50	Extracellular	Incomplete
	4_4215	GH 117	Cytoplasm	Complete
	4_4843	GH 117	Cytoplasm	Complete
	4_3402	GH 117	Cytoplasm	Incomplete
5	5_74	GH 16	Extracellular	Complete
6	6_6833	GH 16	Cytoplasm	Complete

Supplementary file: The amino acid sequences of the agarases in reference set for the analysis of signal peptide.

>WP_010521997.1
MKCLKFVVAALCFTSIGALAQDWDGIPIPASAPSGMTWELNPVSDSFNYESNSRALHPEFEKRWNELYIN
TFSGPSATSYHKDHTWITGGKLNIHAAWDASLPIIYTGCISSKEPLTYPMYMEASVLQAGCMLANNIWMI
SADETEELDMLESYPNIRENQEYFDKRIHLSHHTFIREPFTDYQPRDEEGVFGTWYMEDGRDSWRGEYFT
IGVYWVNPHHAQYYINGKLVRTIKSNEHNFIDPEGNLSEHTTTFDAIDKFGYTGGTGLSKPQHIIINMEQ
QSWITEMNVFPTPEDLDDANGRNLFMVDWIRVYDAVPTGGKVPVTGIDVVGDRAITIRPGETVDLGFAIT
PANATKKIVTWEISNKQIAKVNGLGQVTGLSNGTVGATVTTFDGGFSDVVTVTVAGEPIGPIDQTISVDA
VSISPSVLTLEAGETSKVTAGIIPFNATVQSVVWSSSNSDVVTISNIGDIIAVAEGTATITATTLDGGKT
DSITVTITAGTSTGGGNGGTPTVQVDGGTLTGGPFTFTVGDGVADNVSGVSLSGNVGANSQYVVTDDSGN
ILGLPPTPEAVNFDDAGEGTCFIYHVSFEGTLNGLSAGENISGFSGDFDLSNRIRVDRNQATAPSGINGG
TLSGGPFTFTVGDGIADNVSGVTLTGNTGANSQYVVTDDAGNILGLPPTPEAVNFDEAGAGTCFIYHLSY
DGTIGGLAAGSNISGLSGDFDLSNRVRVDRNAVVIPGTPSGDVIIIEAESLTSTDGTYDDASAGGSGQGV
NVIAIGVNFVNSGDYADYTINVGTAGDYAIEYLISTPSNDAQIQIAVQGTSETVTDNVPNNGDWEDYTAL
TSSSVLSLSAGVQTIRISATGTNLWQWNLDKVTLTRQNGGTSTPIVSGGTLSGGPFTFTVDGIADNVSGV
TVSENQGANANWVVTDEDLNILGLPPTPQAVNFDEAGVGVCLIWHISYEDGLTGLVAGQNVSGLQGTYEL
SSNSVRVNRIAAPSGGNATLVIEAEDFDSTDGTIDDAQFGGQGLGVNAEANNINYVNSGDYAEYSIDVAV
AGTYNIDYLISSPSNNAQIELEVVGSGASLTTNVPNNGQWDSFEPLSGGTVALTAGTNVIRVTATGSNVW
QWNLDKITLSTGTSGAKDLDDVASLELIVYPNPSTDIVFLDGLNSNNSATIHLYDISGAEFKAELNADNS
INVESLAQGVYFITVINGVAKQTARFIKH

>WP_010182069.1
MKRLKFMLAALCLSSIGASAQDWNGIPIPANAPSGMVWELNPVSDSFNYESNTRSIHPEFEKRWNELYIN
GFSGPSATSYHKDHTWITNGMLNIHAAWDATLPIVYTGCISSKSTLSYPMYMESRVKQAGCMLANNIWMI
SDDETEELDMLESYPNTQPGREFLDERIHLSHHTFIREPFTDYQPRDEEGVFGTWYFEEDRKTWRKDFFT
IGVYWKNPHHAEYYINGKLVRTIKKNEHSFVDPQGNLSEHTTSFDAIDKYGYTGGTGLSKPQHIIINMEQ
QSWLAALNIFPTREDLDDKNQRNLFLVDWIRVYDAVPTGGRVPVTDIQVTPVDVNIRPGETFNLNASIIP
SNATTQAITWTTSNSTIAIVNGAGVVTGISEGTVTAEVTTQDGGLVGISTVRVSGTPVAPTDTSIDVEAV
SISPREATVGVGETVELNGAIIPFNATVQSIGWATSNSSVAQVDNLGVVTAVSTGTTTITATTLEGEKTD
AVVITVTDNSGGSADPAVSVTGLNVSPATITIGIGEVECLKGTVAPANASNGAVNWSSNNTDVVLVNNSG
VITGVANGTATITATSRDGSFTDTSVVTVGEGGGSGPIDPVDPVVNGGTLTGGPFTFTVGDGIADNVSGV
SLSGNVGANSQYVVTDDQGVILGLPGVPTAVNFDTAGTGTCFIYHLSYEGTIGGLTAGANISGFTGNFDL
SNSIRVNRNPVVVVVDPAGDKIVIEAETFISTNGTFDDASAGGPGLGVNQTAVGINYVNRGDYAEYTINV
GSAGEYAIEYLISTPSDNAQIQLAINGTALVTDNVPNNGDWDAYTTLTSGTVINLSTGVQTLRVTGSGTN
DWQWNLDKITLTKQGGTTAPVVNGGTLTGGPFTFVVDGIADNVSGVSVSGNQGANSGWVVTDESLNILGL
PPTPQAVNFDDAGTGVCLIWHISYESGLTGLSVGENVSGLQGTYDLSSNSVRVERNAAPTTGQNATLVIE
AEDFDTTGGTFDDAFAGGPGSGVNNAGANINYVNSGDYVEYTVDVAVAGTYTIDYSISSPSDNAQIEFDV
VGSSASTTTNVPNNGQWDSFGSLDGGTVALAAGSNTIRITASGSNVWQWNLDKITLSTGGSGAKNIEGST
SLGLSVYPNPATDAVFVEGLDLTQVNTIHMYDITGAEYPININADASINVEGLASGVYFITVMSLEKGKQ
TARLLKN

>AEK80424.1
MKNFYLILLLSFNLGHLVNAQEWSNIPVPAYAGPGNTWELQSNLSDDFNYNFNAVNYKSNFGNGKWYNFY
HNGWDGPGTTYWTYNKVKVDGDNLVITVAKSNNTSKMGIPGVFSGCVTSNNRVVYPVYVESAISVANISL
ASCFWLLSPDDTQEIDIIENYGNVPWFKQFTHISHHSFIRTPFTDYQPKDWNSWYNDNRVTANYGWGDWC
WNNGNRRYMRMGVYWVGPKHFEYYIDGQLVRVMYHNATATKVNGTWEYQYFNAMNGQFPANNANGYTAVT
TYATGSVYNFSTIQAASNNSNGISVIDPGNFQGGAGFTKAMDIIINVESQQWLALNHTPSDADLASSARN
QMKVDWVRVYKPKSSSGDGSNGTTCADAPDYNGNSNSYSAGQYVINGGILYRKRSDGQWDWIANCNSSSR
VSELVIEDIPEAQIALSPNPAKGFVKISGLGEGSFQAEIVTMQGGQILSTQTVSRATNTLSTADLSPGVYI
IKVAGEAQKLIVR

>SFW66912.1
MMKFNVAILFLFFYAVSCSNNDSTNKLEEKEEIVETPDTPTEEPEVTWKTIPVPPNAGEGNVWEFQDMSD
SFDYEAEAANKGAEFNAKWTDFYHNQWSGPGLTEWKRDHSLVKDGYLQMVASRVPNSNKIHLGCITSKEQ
VIYPVYIEANVKIANTTLASDVWLLSSDDTQEIDIVEAYGASYSELAEADQTWYAERIHLSHHMFIRDPF

QDYQPTDSGSWYKDEVGTLWREEFHRVGVYWRDPFHLEYYINGKLVRTTSGEDMIDPNDFAEGKGLSKPM
DIIINAEDQTWRSDKNITPSDAELANEENNTFKVDWIRIFKPVKAD

>SER23339.1
MQSLTPQILNRCTFLLFIAFLSFSLAGQDWKKLPVPVPAGPGQSWKLLKELSDDFNYRGKKKRFRRRWQD
TYFNRWNGPGLTQWQGDHSSVEGGNLVIRASRREGTEQVNCGIVTAKAALMYPAYTEARIKVSNLELSSN
FWFLSRDSRREIDVLEVYGGAEKGDFARRMSTNFHVFIRSEEKGITSDFNASNHVELPDEAYWREEYHTF
GVFWKSPAEVYFYIDGKPLPTGSWAQAEMFDKDYTKTFMDKSAFVMDRPMVMILDTEDHHWRSRQGIIAK
DEDLAAPSKNKMLVDWVRTYRLVEE

>WP_070726687.1
MDPEGYYFLHNAPNAVSHGTSERTKKALQEKYGNAAGWMTATRKFLFANGFNGAGAWSATKEIQAENARA
DRPLAYTINLDFMSKYGDKRGGTYVQPGHKGYPNDVIFAFDPGFEAFAEQHAKQLAKNRKDKNLFGYFSD
NEMPLLRKNLDGYLTLPVTEPGYVAAKKWADERGITLATITDQNRQDFLAFAAEKYFSIVAKAIKKYDPN
HLYLGCRFHGAQRYYPELMKVAGKYLDAVSINYYNNWTPEKRWVAEWEQSAGKPFLITEWYVKGEDAPGL
ANTAGAGWVVKTQADRGLFYQNFTLGLLESKNCVGWHWFKYQDNDPTIVGAEPSNTNANKGIVNNYLEPY
TPLLDKMRELNLQMYPLADFFDQRTPQP

>SEC64965.1
MKFKSLVLSALFLFLLTASNCSSSSEVNEAPEVDEVDTTDPVEEGEEVEEEEAIEENYSTMAVPADPGSG
LEWEFQDFSDEFDYEAPAGNKGDAFYNKWDDFYHNNWSGPGLTEWSRDIPFVSDGLLQIPAKRKAGTDKI
STGCITNKTRVQYPVYVEARAKIMNSVLANGIWMLSPDDTQEIDIMEAYGAAYSESAQASHSWYEVRMHI
SHHVFIRDPFQDYQPKDAGSWFMKDNIKWREDYHTYGVYWRDPWHLEYYIDGELIRTVSGKDQIDPLFYT
NATNPGDTSTDIRTGLNKEMDIIIDVEDQDWRSSPASGNQSDTYTPTDNELSDTEGHTLKVDWIRIYKPV
EK

>SDX72292.1
MTRRILTFILFLNLFTFYAQTNVTVNLNVKHSVGEASTFDRSKFMVIHANSTENEWNGDNFTSDLKDHFL
NGYDVYLGRDTGGITWNINQIQEDPARTGFANPTEIASRGLTTRNNFANNTDIHQYEVRKVHNVVGAQLH
PFWTGESQKETGQGWKFASPTATGEYMGRYFNEFHGDNGQKVPTYIEVINEPAYEALGGKMDYSNSIQEI
ADFHVEVADAIRAQNSNLKIGGYTTAFPDFEVGDFQRWNNRWKLFMDVAGEKMDFWSIHLYDFGSIRGGE
KELRSGSNVEATFDMMEHYSKLSFNEVKPFVISEYGAQTHDYNNETWSSYRDWLHVKASNSQLMSFLERP
ESIASAINFVIVKAEWGYNTDKDIPYPSRLMRKTNEPDSYTGQWVYTDMVKFFQLWQNVNGTRVDSITDD
LDIQVDAYVDGDKGYVILNNLNFEATTVNLDVFDASNINVSSILKRHLTLSGGNTTVLEEETFNAAISSV
EIGAESTVILEYTFDSAITINKTSKETKYYATSYLKPIVANQPNTFNINNVVKGSFGEAVLRVGVGREHG
KSLHPSVKINGQTIEVPNDWRGYDQAQRERFFGVLEIPVSFGELQENNTVEVEFGDAGGHISSVSLQVFN
FSDNIRVFDPLSLPSNNFEIKTIGSTCTGENNGKLEIKSVKSLNYAVTIIGDNYNKNFTFTNNLNVEGLK
PGTYTVEVKIDEFPDYNARFIIEITEPEAISVSSKIDISSKSVVLKLTGNTNYIVNINGKEVETTFNEIS
LPLNFGINSISVKTDKECQGVFNEKLMLENSLILYPNPTEKQFSAYMPKEMIGGEISIVSMNGLLVHSQK
IVDENQQVDASNLSKGVYIVKISNKSKIQLQSKLIIN

>SDX90069.1
MTKKLLYIFLIISIYHFSTFAQDIVDINLNVKHSVGGVSELDRSKYLMLHAGVDDNEWPNEEFKREFLED
FDTYLGRNNGSLPWNLTICEEDPSKPGWPSVSYLQTKGAQEVNKYKNNTSEHKYENRISRYMMGGQEGMY
PNGSHEIGPNGAKWTMIKDDYKPIAEFYANYFKYFFGTGGINGQPLPTYIEVMNEPFVKANSLGTTRTNL
SQMHKVVAQRIKELNPNAKVGGYSAAHPAYEASNFQHWEKNWKTFIDIAGEEMDFFSLHLYDNVQKNDEG
QYRAGSNVEAILDMVEHYEYLKLGEVKPFCISEYGCLNTEGELYTKERDWHNLRSFSTMMMQLLERPDVI
DQALPFIILKANWWKPSGSQDPNAKYAHRLFRQKKELEGETGDEWVYTEIVKFYQLWKNVNGTRVDTKSS
NLDTQIDTYVNGNKAYVIINNMHHEARTVDLSLKGFGSAKIQNIVKKHLHAENSGIPALDEETFTSNITK
VNIGREATIILEFTFDKEITISDTSNETKYFADTYYKPITANQAINFNINNVALDTKGEAILRLGIGRDH
GKSLKPTLKINGTTVEVPTNWRGNDQSGRDRFFGVLEIPVPYNILQANNTVSVTFSDTNGHISSLTLQVF
NFSKEIIRSEEKIASNNFTIKTTSETCDNKNNGKIEITALESFDYVAIITETSSTKNFTETTSFENLAVG
TYELCITIPSKPTYKQCFSINIKEVETLGVSSKVNSSAKTATYNLKGGTNYTILLNNKTYKTSNSTIELP
LINGLNSVEIKADKECQGVYKNSLFIGSEIIAYPNPFKNNFTIDLGIDTSKTASIAVYSSIGNLVYSKKY
TVNNGKINLDLNSLNSNVYMLEVKTEKSSKKIKIIKQ

>OGX87058.1

MDPEGYYFLHNAPNAVSHGTSERTKKALQEKYGNAAGWMTATRKFLFANGFNGAGAWSATKEIQAENARA
DRPLAYTINLDFMSKYGDKRGGTYVQPGHKGYPNDVIFAFDPGFEAFAEQHAKQLAKNRKDKNLFGYFSD
NEMPLLRKNLDGYLTLPVTEPGYVAAKKWADERGITLATITDQNRQDFLAFAAEKYFSIVAKAIKKYDPN
HLYLGCRFHGAQRYYPELMKVAGKYLDAVSINYYNNWTPEKRWVAEWEQSAGKPFLITEWYVKGEDAPGL
ANTAGAGWVVKTQADRGLFYQNFTLGLLESKNCVGWHWFKYQDNDPTIVGAEPSNTNANKGIVNNYLEPY
TPLLDKMRELNLQMYPLADFFDQRTPQP

>SDS10186.1
MKFKFLILSSLFLFLLTASNCNSSSDVNETPDVEVDNTDPKEGEEKEEETEEDYSTMAVPADPGSGLEWE
FQDFSDDFEYEAPAGNKGDAFYNKWDDFYHNNWSGPGLTEWSRDIPFVSDGLLQIPAKRKAGTDKISTGC
ITNKTRVQYPVYVEARAKIMNSVLANGIWMLSPDDTQEIDIMEAYGAAYSESAQASHSWYEVRMHISHHV
FIRDPFQDYQPKDAGSWFMKDNIKWREDYHTYGVYWRDPWHLEYYIDGELIRTVSGKEQIDPLFYTNATN
AGDTSTDTRTGLNKEMDIIIDVEDQDWRSNPASGNQSDTYTPTDNELTDTEGHTLKVDWIRIYKPVEK

>OFO89203.1
MKNSIVILFLLLLSQFGYAQGHTFKVTARPWVKGQKNLPWKEYDTRTIAQLDGFKPTGKVRVNKYGSDLD
ASRHRATGFFRVERIGDRWWMIDPDGYRHLQKVVVGVRLGTSERNKQAMLDKFGTEEKWIEGTARMIHSL
GFSGAGSWSNEEAIASYNASHKEVLTRSIILNLMSGYGKKRGGTYQLPGNTGYPNQCIFVFDPEFETYCD
EMAQKLVANKTDKNIIGYFSDNELPFGPKNLEGYLTLKNPNDPGRLYAESWLKQQGITLQQITDEHREEF
AGVVAERYYKVVSEAIRKYDPNHLYLGSRLHGKPKFVRQIVEAAGRYCDVVAINYYGAWTPNEKTMKHWG
EWAQKPFIITEFYTKGMDSGLANTTGAGFTVQTQQERGYAYQHFVLGLLESGNCVGWHWFRYQDNDPTAK
GVDPSNLDSNKGLVDNEYNLYKPLADAMKELNINAYRLADWFDQQSTNNK

>WP_068828862.1
MKKSIYLFIILVGWSSAKTYAQAKTEQDYILVDARVRVYHKDGTRTYKEYQPFKTQTLELLPNFNKEKSL
SKLSKYGGLMADKTKFATGFFHVKKIDGRWWGIDPLGYKYFNISLNSINTGKSKGSKVAFKEKFGNKENW
IHQTVEMLQNHGFNCAGSWSDVEAIIEANKTLEKPLAYTVNWNFMSSYGRKRGGTHQQAGHTGYPKDAIF
VFDPEFKTFCDEHAKQLLKYKDDPNLFGHFSDNEMPFKQKALDNYLKLPKNEHGYKAAIDWLKENGITKA
EITDTHRDLFMAYVGDTYFSIVSKAIKKYDPNHMYIGARFYSEEKNHAAFMKAAGKHLDIISNNYYNHWT
PDKTDLVNWTKWSGRPFIITEYYVKGEDSGMGNTSGAGWIVRTQKDRGLFYQNYNLALLASKNCVGWHYF
KYQDNDPTAKGVDPSNIDANKGIVTSDYKPWTAMLEKMKALNNRVYDLINYFDHHEN

>WP_066838698.1
MDPEGYYFLHTAVNTVTMGASARNKEALKETFKDSRGWASAANQLLRENGFNGTGSWSNVKELIASPRQE
KTPLAYTVNWDFMSAYGKKRGGTYQKPGHRGYPNDVIFVFDPEFEAFCDNHAKQLAAYKDDKNVFGYFSD
NEMPFGRKNLEGYLTLDPKEPGYAAAHQWIKERGITPEQITDAHRKEFLAFAAERYFSIVAKAIKKYDPN
HLYLGCRFYGNQREYPEMLEAAGKYVDVVSINYYNVWTPEQDKMRNWGAWSKKPFIITEWYVKGEDSGLP
NRTGAGWMVKTQQDRGLFYQNFTLGLLENPNCVGWHWFQYQDNDPTLEGAEPSNIDANKGIVDNYYQVYQ
PLMDKMREINQHMYQLADYFQKKK

>WP_066622260.1
MLRLFFLSAMVSPWACKAQQKSPEPLIVQAKRGLTTSDATLGYGEWKDYETRSLAHLTDFKPKNSVKLSK
YGGRKDKRTQATGFFHVKQLDGRWWAIDPEGYYFLHTAVNTVSMGGSTRNKEALKEKFTDTKGWAQGTHQ
MLLDHGYNGLGSWSTVKEFRETPLQEKSPLAYTINLDFMSAYGKKRGGTYQKPGHRGYPNDCIFVFDPDF
ETFSDEHVKQLVAYKDDKNLFGYFSDNEMPLLRRNLEGYLKLDPKEPGYQAAKKWIDEKGISPEQITEAH
KKEFLQLVTERYFSIVSKAIRKYDPNHMYLGCRYYGPQRDYPEMMEAAGKYVDIVSINYYNVWTPEAKKM
EEWGKLANKPFMITEWYVKGEDSGLPNKTGAGWMVKTQEDRGLFYQNFTLSLLESKNCVGWHWFQYQDND
PTKEGAEPSNIDANKGIVDNYYNVYAPLAEKMKEINRNKYQLADYFIKKKN

>ANW96656.1
MIINGSNNFLNNMKKSIYLFIILVGWSSAKTYAQAKTEQDYILVDARVRVYHKDGTRTYKEYQPFKTQTL
ELLPNFNKEKSLSKLSKYGGLMADKTKFATGFFHVKKIDGRWWGIDPLGYKYFNISLNSINTGKSKGSKV
AFKEKFGNKENWIHQTVEMLQNHGFNCAGSWSDVEAIIEANKTLEKPLAYTVNWNFMSSYGRKRGGTHQQ
AGHTGYPKDAIFVFDPEFKTFCDEHAKQLLKYKDDPNLFGHFSDNEMPFKQKALDNYLKLPKNEHGYKAA
IDWLKENGITKAEITDTHRDLFMAYVGDTYFSIVSKAIKKYDPNHMYIGARFYSEEKNHAAFMKAAGKHL
DIISNNYYNHWTPDKTDLVNWTKWSGRPFIITEYYVKGEDSGMGNTSGAGWIVRTQKDRGLFYQNYNLAL
LASKNCVGWHYFKYQDNDPTAKGVDPSNIDANKGIVTSDYKPWTAMLEKMKALNNRVYDLINYFDHHEN

>ALJ05413.1
MNNMKRILFVIVILFIWNDIKVFAQNNAGQAFTLVDAKTKIYHKDGTRSYKDYKPFKTRTIELLKGFEVS
TNLPKVSKYGGSLEHKTSHATGFFHVKKMGDRWWGIDPLGYKYFNIAINSINTGKSIRTKKAFTEKFGSK
KKWINETIELLQNNGFNSAGSWSDVEAIVEANKTLEKPLQYTVNWNFMSSYGRKRGGTHQQSGHIGYTND
AIFVFDPDFKTFCDEHAKKLLKYKDDPNLFGHFSDNEMPFKFKAIDNYLALPKDDYGYLAAKKWLKDNNI
NQEEIIDKHRETFMAYVADTYFSIVSAAIKKYDPNHMFIGSRFYSTERNYPQFMKTVGKYLDVISINYYN
HWTPDNEDVVNWEKWSNTPFIISEYYVKGEDSGMGNTSGAGWIVRTQKDRGLFYQNYNLALLESKNCVGW
HYFKYQDNDPTAKGVDPSNVDANKGIVSNEYEIWTPMMEKMKALNINVYSLIEYFDKNEK

>EFI11824.1
MMKNSIVILFLLLLSQFGYAQGRTFKVTARPWVKGQKNLPWKEYDTRTIAQLDGFKPTGKVRVNKYGSDL
DASRHRATGFFRVERIGDRWWMIDPDGYRHLQKVVVGVRLGTSERNKQAMLDKFGTEEKWIEGTARMIHS
LGFSGTGSWSNEEAIASYNASHKEVLTRSIILNLMSGYGKKRGGTYQLPGNTGYPNQCIFVFDPEFETYC
DEMAQKLVANKTDKNIIGYFSDNELPFGPKNLEGYLTLKNPNDPGRLYAESWLKQQGITLQQITDEHREE
FAGVVAERYYKVVSEAIRKYDPNHLYLGSRLHGKPKFVRQIVEAAGRYCDVVAINYYGAWTPNEKTMKHW
GEWAQKPFIITEFYTKGMDSGLANTTGAGFTVQTQQERGYAYQHFVLGLLESGNCVGWHWFRYQDNDPTA
KGVDPSNLDSNKGLVDNEYNFYKPLADAMKELNINAYRLADWFDQQSTNNK

>EFI11823.1
MKKLFILGTFLFISSIPMVSCTDDDDKDPNFMPPDIVMGGGDVESEYPEDLPAPGASVMYTPSLNANMYR
PISVKYSSAYPPISSWKTENTRIIAYMDGYKPAIKTLKAYQESVNKYGSSTTLPKQAATGRFYTKKIDGR
WWLVDPEGCLHLERSATSLRKGTSSRNKTAWNSRFGTDEKWLSTTQRELSEIGFHGTGAFCTGTYSLIQT
HNASNPSSPLTLAPSFAFLSQFKSAKSYNYPGGSDDNAAGLVFYNGWTEWCESYLAGSAFADYLRDPNVL
GFFSDNEINFSSNSSRILDRFLAISNSSDPAYVAAKAFMDSKGTQSVTDDLNNEFAGIVAEKYYKAVKEA
VKKVDDKLLYLGTRLHGTPKYMEGVVRAAGKYCDVISINYYSRWSPELTTAIADWANWADKPFLVSEFYT
KGVEDSDLNNQSGAGYSVPTQYERAYAYQHFTLGLLEAKNCIGWHWFKYQDDDGTDNSSKPANKGLYDNS
YQLFPYLSFFARELNFNAYDLIQYFDK

>GAL80684.1
MFQKIEKYTFLALALFVITACSNNDSDEKEEVIPIDTTNPTITCESSISVFIDAGKNDAIVNYTAPVGTD
NVLGAVTAQTAGLASGSSFPIGTTTNTFVTTDKAGNTASCSFDVVVMEPSGDKPYAVADGVEVPSGKKWV
KVEDMSDEFDGTTFDDNKWHRYPSSDGFSWIGRPPALFEADNVTVANGNLNITVEKFDAPKRVNGIDWTH
GGAIVRSKITAKPGYYYECKMQANKTIMSSTFWIAFKQNCNTGPVRKLELDIQECVGRLTNTAAWADNFD
QIYASNTWRHERDCDTEVTGSKQKSCQNYFNRKK

>GAL80600.1
MKKYIGLLLIFASICHVSAQSGPPEPPVGKRWVINPSFSDEFNGETLDSDKWYDYHPSWKGREPGIFLPS
QVSVKNGFLQIKGEKLEKDTIVKAYGRELKFNIAGGAVVSKKTAFLGYYECRAKAAATTMSTTFWFSTTG
AEDGPNGCDKYGQEWDIQECIGRSGDFAGSFFSNGMNSNGHFWYTDCDNKRHDLRAPAVKFVNKELASKD
FHVYGGWWRDEKTATLYYDDRAPKHMKFYDEIVDKPFNRPMYMRLVSETYPFPWIELPTDEELSDPGKNT
VYYDWVRGYDMVDVDAKDIDQSYEKGLNLYNESIIFSEVETLMEVTDGLKIPLSFKVNEHRKIYIKISET
TDKLKEKWNKKVFEKTIDVYPGYGHMEVVCNVDKKMSKSATYVVEALIRDINEENKSKGALDTSTLFFTI
R

>GAL80590.1
MKKLYIVLFTCTLFNSLLYAQTPAPEGKKWVKIDNLSDEFDGKKLDNKKWIGDPEKHPDFGWIGRPPALF
KESSIKVNKGFMEIEVGKLDKTITSLKYKTPSIYNYYGGIIRAVQPISYGHYFESKFKMSKTEMGGGFWL
MSRNNCGKKHEIDITESVGSVSPLAKDWGEKIWDKIMHSNTIFRKTECNEATRSQDMIIPDVKNSDKFYT
YGCWWKSPTELLFYLDGKHVYTIKPPVDFDQQLFIHFSIESYDWNPIPEDGGKVASANKEDRTAFIDYIR
TYKLVDSKD

>GAL62734.1
MNVSVDTFSNGAVVTFTSPVGTDNVAGSVTTQTGGLASGKVFPIGTTTNTFTTRDAAGNTSICSFNVIVT
RKAPSEDLPYFVIENPTPAGKKWAKIENLSDEFNQTDGIDRTKWYTKPDIASAGWFWTGRPPGLFIEESI
TVADGKLKIEANKLPTTKIINGKSYDYSGGIVRSINQCKVGYYYESKMKANKTFMSSTFWMMTEENACPK
RLELDIQECVGELTPGADAWAVSGKFDQIFHSNAFHRTSCANTVETRKQGSVITDVKNWSEYMVYGFWWK

SETELWFYLNGKLAYKITNPTTTFDLPMYYNLAVETYDWNPPHADGKGMEKFTKEERSTQYEWIRTWKLD
DK

>GAL62680.1
MTSAATNSGVTDGNLWIKATLNESNPEDRWFQTARIHSKAETSFPMYTEARIKAAHISAYNTYWLNNGDI
NNRDEIDIIENNSKPSCVGCSQAADFPNQMNSQYFHADSSKSPVTIRDEDNFKRSGLSDTNPLKNVKWNE
DYHTFGVWWKDAKHIQFYLNGEPAGSVEVGEDRSGTTYTGREFSRDLEIIFDLWTNEAVWVGGLPPKSDL
ADNTINTMKIDWVRTWKLEDE

>GAL64777.1
MKKLYIVLFTCTLFNSLLYAQTPAPEGKKWVKIDNLSDEFDGKKLDNKKWIGDPEKHPDFGWIGRPPALF
KESSIKVNKGFMEIEVGKLDKTITSLKYKTPSIYNYYGGIIRAVQPISYGHYFESKFKMSKTEMGGGFWL
MSRNNCGKKHEIDITESVGSVSPLAKDWGKIWDKIMHSNTIFRKTECNEATRSQDMILPEVKNSDKFYTY
GCWWKSPTELLFYLDGKHVYTIKPPVDFDQQLFIHFSIESYDWNPIPEDGGKVASANKEDRTAFIDYIRT
YKLVDSKD

>WP_027473776.1
MKSFTKYFNPTVSFLMVLLLSCSIHNRQKSKASEYLMVDAKTKAYHKDGTRSYNDYQPFKTRTINLLADY
QQPKQAPDLSIYGGDMSQQVEATGYFHVKKVGSRWWAIDPLGYKYVNIGLNSVKTGKTKVSKDALQQKFG
SKENWTQETMQLLHNHGFNCAGSWSDTEAIIAYNKTAERPIAYTVNWSFMSSYGKKRGGTYMQPGHTGYP
QDAIFVFDPEFESFCDTHAQQLAKLKDDPHLFGHFSDNEMPFKFKALDNYLSLPTDDYGYMAAIKWLKDN
GISKDSISNKNRAAFMALVADKYFSTVAKAIKKYDPNHMYIGARFYSNEKNHAAFMKAAGKHLDLISTNY
YNYWTPDSAQMDNWTKWTNKPFIITEYYTKGEDSGMGNTSGAGWIVRTQKDRGLFYQNYTLALLESKNCV
GWHYFKYMDNDPNAKGVDPSNIDANKGIVTATYEPWTPMLDEMKPLNLQVYHLIKYLDHK

>WP_010522038.1
MKKQFFKIALGSLALLLGLTSQAQINVEVNAHTKHQVGGKSNFDRKKYITVHSANFEADWNGQNGEGGAP
GRLFELTRTLDAWLSRETGAFTRAITGFSAAQINSQSLGVSEGFSYPALSDRFDYERDQDLVVAAQEKAY
VQFNADGFAWGRRAAAWVKKHGNGGTTGRPRPSYIEILNEPVFPLVDFIPEGKSPEPLDRIFAFHKDAAI
TYKNNVPANGRPKVGGWTTAFPLLDKRNFGQWNDRWKRFIDIAGGAMDFYSLHIYDQPVFGSGNRDWAFR
KGGNIEATLDQLENYGFITGKKKPFLISEYGSQLNNNYRERYTPRRDWYSLRSMSSMMMQFMERPDQIVK
AIPFVPIKAEWAYGLAENSPISPGVGFPYSWRLMKNNNEPYGTYWVNRNNPFGSNRNVPNQNYSYTELIR
FYQLWSDVRGTRVDSKQADVNLQVDSYVQGKDLYVIVNNLYRTSKSFNLNIRGTGTNPVEYVQEKHLFAD
ANGNPFYSPRFVGTNKASTMTIGAEATMILRYHFKNNINVNQTSNETKHYANRNAASYLEYIEANQNQNF
NINIGNKPRFGEATLRIGVGRAHNLQKLPRVFVNGTEINSAKQWRGSGAGQNFRERFHSIVEVDVPLSLL
KANANNVSVRFPDAGGFITSMAMQVFRMTANIRGNVGASPSAPSTPAVSGIPVGQVITLQKSGGDFKYLS
AVLELNNDIYANQSQAFGNRQKFRIVSHPLGGVALFNLNSQRYLQVSGFNQNVIVGARGIDTKGWERFEW
VSKGAGKVALKSLHSGKWLQAPHNQSNTAVFPKGNADRSWETFNWKITTSSTSSRTLASTDVKHDAEILI
YPNPVSAGHKVFIANTTKNATINVFDVSGNLVNSIKAENGMSSIATDGFATGIYFIQINGETPKRIIVK

>ANQ52166.1
MKFRFLSLFIGLYITLLSIEVLAQTNITVYPNTQRFIGDVSTFDRSKYLQAHIWFGNEPNDVDFKSFKST
YNIDENYIGSRRFWSPLGLVKNGVIPNIRNNYSGVRSVNNHVDTGRSGSLMYDENVDYSTVDITDFSIEV
AEFVAKNYRDEWNPVPKYIEPFNEPMIHAADHYPGSYNSAKTDEIVTKICQFHQHLGKAVRDIPELDNMK
VIGYASAWPEFENGDFSLWNSRYKKFIDIAGEDIDLFSIHLYDGVGLNNTGGRRSGSNSEAILDLIEAYS
FLKFGKVKPIAITEYGRLVADQEGWTWNNGQSNYHPIENSQAVRSQIHMAMSFMERGEQIEMAIPFSTQK
INPKEKYSKAALWTLNDDQEWELTSRKYFFEIWKDVKGERVKIENDNVDVQSMAFVDDNKMYVVLNNLND
ETQNINLKLNDNIGVDKIDIKQLKVYVDQLPELSNQTLNEVPESLPIQYGETIVVTYHFTDVVDLSNTLF
EKKYYASTYLKPISSNTEQKFTIKNVTKENGFGKLRIGIGRDHGKSLLPTVKVNGQEVIITNDIIKGYDQ
SNRSRFFGVLEIPFDQNLLKSGSNEISVLFSDQGGHIASVGMIVNTTTKAADIVEDEDDITSLDDHGTEK
IKIYPNPADKEINLFFNKVGKAQIKLFDLHGKLIKVYISDLQHQVIPTDDLQKGLYILKVNQADFIETHK
ILVK

>ANQ51568.1
MRTTLLILSLMYSFVLTAQDKAKVTFNPSTKMSYDGVTKFERKNFINVHTTYEHIPQQFRKELYEELGVS
DGRSFGLGTPSNKLSGTNHKHPGTFSEKALKAEMKKIASHSTPRKFEDEIIVTSHPGSYFPVGEFVLEKK
KEQLVGIPAIEEYFQQRLRESTYRKGYIEILNEPFVHDRDMHTSVDTIIDMFKYTAKAIHQKYPQAKVGG

PGHAWPAYELNDFQIWKERMGKFIEVAGNDMDFLSVHLYSTYYDDKASYRAGANAEAILDMIEAKSLQVT
GTIKPLVISEYGSGFKKGTNIVQAYYKERDWYVIHGVNSKMMQFMNRPHRIVKSVPFICGRADWYDNEHP
YPWVLFHKKNNEYQKTELIKFYQFWKDVKGEYVFIQSDHADVQTVGFRDGNKVFICLDNLEDKTIEVDLA
GTGFKHQKAELRRIYSEGNGPILSQQSISDISKITLSSDETAILILELNDDTDEFSSTIKLTEQYHPETI
QKIGQQEKEYQFDVNPDELVFVDLHLGIARPIAEDFLPIVKLNGQKISVSRNQMGRTQDFKELTFENALE
PKIKRPQQQNFFGVHKIALPKALLKKKNVLSLSFEKGEGYISTVKLVQGHQSEMNQ

>ANQ51442.1
MNLNLKHCVSSAILGLALFSTTTFAQNKVTVDPNTQRFLGNKTTRLDRDKYLQAHVWFGEKKDAEFEAFK
KKYNINPEYQGSRRFWSPMNMIKNGKIPKVKNKYSGVREVKPYTVVTGRATDLFYDRKVDYSVEDITDYT
KKVAAYIAQSYKKDWEEMPAIYEPYNEPMVHASDLYPGKRNGEKSEIAIKKISESLSEIGKAIHAVPELK
NMKVAGYASAWPEFEHNNFDVWNQRFKQFIDIAGADMDILSVHLYDGKGLNNSGGRRSGSNAEAILDMIE
AYSYIKLGEVKPIAITEYGRLVDDQPNWKPNNGSSNYHPIENAQAVRSQIHLAMQFMERGDNIVTTTPFS
TGKQDPTKKYAKAGLWTKNDKGEWELTSRKYFFELWKSVKGKRVNIASENVDIQTQAFVNGKQLYVILNN
LNEETQSIPLSVVGDQDFKNVLVKRIKTYVNKMPKMVNKTLKTAPESIDIEYGETILLTYNFNNKIETPQ
KEYRAKYYASEYLKPIQPNKKNTFEFASVTAGTKGSTTLRLGVGRAKGKSLQPTVFVNGQKVNLPNDIIR
GYDQHNRKKFFGVLEIPVPHSMIKNGKNEVSVTFTDGGGHISSMILQVISDKKSFKTKVN

>EFI37873.1
MKNSIVILFLLLLSQLGYGQGRTFKVTARPWVKGQKDLPWKEYDTRTIAQLDGFKATDKVHVNEYGSDWD
APKHRATGFFRVERIGNRWWMIDPDGYRHLQKVVVGVRLGTSERNKQAMLDKFGTEEKWIERTAQMIHSL
GFSGTGSWSNEEAIASYNASHKEVLTRSIILNLMSGYGKKRGGTYQLPGNTGYPNQCIFVFDPEFETYCD
EMAQKLVANKMDKNIIGYFSDNELPFGPKNLEGYLTLQNPGDPGRVYAESWLKKQGITSQQITDEHREEF
AGVVAERYYKVVSEAIRKYDPNHLYLGSRLHGKPKFIRQIVEAAGRYCDVVAINYYGAWTPSEKTMKHWG
EWAQKPFIITEFYTKGMDSGLANTTGAGFTVQTQQERGYAYQHFVLGLLESGNCVGWHWFRYQDNDPTAK
GADPSNLDSNKGLIDNEYNLYKPLADAMKELNINAYRLADWFDQQSNNNQ

>ACY02047.1
MNLNLKHCVSSAILGLALFSTTTFAQNKVTVDPNTQRFLGNKTTRLDRDKYLQAHVWFGEKKDAEFEAFK
KKYNINPEYQGSRRFWSPMNMIKNGKIPKVKNKYSGVREVKPYTVVTGRATDLFYDRKVDYSVEDITDYT
KKVAAYIAQSYKKDWEEMPAIYEPYNEPMVHASDLYPGKRNGEKSEIAIKKISESLSEIGKAIHAVPELK
NMKVAGYASAWPEFEHNNFDVWNQRFKQFIDIAGADMDILSVHLYDGKGLNNSGGRRSGSNAEAILDMIE
AYSYIKLGEVKPIAITEYGRLVDDQPNWKPNNGSSNYHPIENAQAVRSQIHLAMQFMERGDNIVTTTPFS
TGKQDPTKKYAKAGLWTKNDKGEWELTSRKYFFELWKSVKGKRVNIASENVDIQTQAFVNGKQLYVILNN
LNEETQSIPLSVVGDQDFKNVLVKRIKTYVNKMPKMVNKTLKTAPESIDIEYGETILLTYNFNNKIETPQ
KEYRAKYYASEYLKPIQPNKKNTFEFASVTAGTKGSTTLRLGVGRAKGKSLQPTVFVNGQKVNLPNDIIR
GYDQHNRKKFFGVLEIPVPHSMIKNGKNEVSVTFTDGGGHISSMILQVISDKKSFKTKVN

>GAF05493.1
MNKIFVFFLWMTISHLLFADPPKPPLGKRWVMNPDFSDEFNGTVLDTTRWLDHHPTWRGRAPGLFMPSQI
SVKDGFLQIKGERMLRDTVIHAYGKDISFYIKGGAVVSKKSVFLGYYECRVKAAATTMSTTFWFSSTKSF
KGPNGCDKYGLEWDIQECIGRRGDFNGSYFAHGMHSNSHYWYTDCKEEKHDYRAPQVKFEDRQLASDDFH
VYGGWWHDETMASYYYDNGAPKYQKFYHKISDKPFDQPMFMRLVCETYPFPWIELPTDEELADPTKNVVY
YDWVRGYKLVDVKDPHMSQVAPETEIGLYYEDIEFESASMELPQGNVLKIPFCYKANENRDIHFVLKDAE
GKKVASAKYKAYTGYANLIVDLKVDVQLAAETEYTLWADIRPENGTKTDILNSSVLMIELVENGAL

>ADY28280.1
MMKFNTAIVFLLFYVVSCSDSNSTDKVEDEEVIETPETPTEEPEEEPEVTWKTIPVPAEAGEGNVWEFQN
MSDSFDYEAEATNKGAEFDAKWTDFYHNQWSGPGLTEWKRDHSLVKDGYLQMVASRVPNSNKIHLGCITS
KEQVIYPVYIEAYVKIANTTLASDVWLLSSDDTQEIDIVEAYGASYSELAQADQTWYAERIHLSHHMFIR
EPFQDYQPTDAGSWYKDEVGTLWREDFHRVGVYWRDPFHLEYYINGELVRTTSGAEMIDPNNYADGEGLS
KPMDIIINAEDQTWRSDKNITPSDAELANKENNTFKVDWIRIFKPVKAD

>ADV49752.1
MIKLKLIALILIFQIASCSKSSTDEKPTSEEDNQGEVITPEEEVEEEVVKDWKTIPVPADAGAGNVWEFQ
ELSDDFDYDAPADAKGAKFDKKWTDFYHNLWAGPGLTEWRRDHSLVVDGNLQMIANRAEGSNKINLGCIT
SKEQVVYPVYIEANVKIANTTLASDVWLLSSDDTQEIDIVEAYGASYSELADSDQTWYAERIHISHHMFI

RDPFQDYQPTDAGSWYRDGTLWREDYHTVGVYWKDPFHLEYYIDGKLARTTSGTEMIDPNNFAEGKGLYK
PMDIIINAEDQTWRSDNNVTPSDKELENKENNTFKVDWIRIFKPVPAN

>AIA22719.1
MKYFNLLFLSFLCTTVIAQDWSSIPVPADAGNGKTWELQENVSDDFNYTFDAVNSRSNFGNGKWYNFYHN
TWDGPGTTYWKYQNVAVKDGSLVINTSRWDQSNQSNPWNGNSPKMGKPNNGVNSGCVTSNAKVQYPVYVE
SAISVANIDLASCFWLLSPDDTQEIDIIENYGGVDGFKHLTHISHHSFIRSPFHDYQPRDWNSWWPDSRV
NTSYGWGDWAWNNGDRRYLRLGVYWKTPFHFEYYIDGDLVRVMYHNAIATLMNGTWEYTYYNQIHPAGTQ
DSYGNNIGGQPVNLSNGYSAVTNYAYSSEFEFATLQAASNASNGYNVIDPGEYQNGNGFTKEMDIIINVE
SQSWLVSYGKTPTDASLANPAKNAMEVDWVRVYKPVNGGTPTPPVSVSGVSISPTSLSLETGDTGNLTGQ
VIPSNADVQTMTFTSNNDAVATVNQSGLVTAIAEGTAVITATTTDGGFTATSTITVSTTGTTPPPSGETL
VIEAEDFARTSGTFNDGFVPYGANKISGTGINWVNSGDYVEFDVNATAGTYEVSYSISTPSNNAAISLSA
NGTSAGSTPVPNNGSWDNYSDLDAGFTIVLSEGSNVIRATASGSNNWQWNLDKITLTAVEGEQTIPVTGV
SVNPGNLTLEINETGNLTGQVSPANADDQTMTFTSNNTSVATVTSSGVVTAIAEGTAIITATTTDGGFTA
TSNITVNATDEGPGTGNPAETIVIEAEDFIATGGTYNDGNVPFGVNKANNIGINWVNSEDYADYEINVTG
AGTYDIQYMISTPTNGDTRIQLQIDGAAVSTDNVANNGQWDDYQALNGSSVQLSEGTHTVRVYAIGDATW
QWNLDKITLTPQSSSSRIAAEVTRNDMYVFPNPTRGQVNISGISTGDYTFTIYNMNGVQVHSENAYFSIK
KTIDISTLSSGIYFIKVAGKEGSYNARILVK

>AKH41067.1
MMKFNTAIVFLLFYVVSCSDSNSTDKVEDEEVIETPETPTEEPEEEPEVTWKTIPVPAEAGEGNVWEFQN
MSDSFDYEAEAANKGAEFDAKWTDFYHNQWSGPGLTEWKRDHSLVKDGYLQMVASRVPNSNKIHLGCITS
KEQVIYPVYIEAYVKIANTTLASDVWLLSSDDTQEIDIVEAYGASYSELAQADQTWYAERIHLSHHMFIR
EPFQDYQPTDAGSWYKDEVGTLWREDFHRVGVYWRDPFHLEYYINGELVRTTSGAEMIDPNNYADGEGLS
KPMDIIINAEDQTWRSDKNITPSDAELANKENNTFKVDWIRIFKPVKAD

>CAZ98338.1
MKKNYLLLYFIFLLCGSIAAQDWNGIPVPANPGNGMTWQLQDNVSDSFNYTSSEGNRPTAFTSKWKPSYI
NGWTGPGSTIFNAPQAWTNGSQLAIQAQPAGNGKSYNGIITSKNKIQYPVYMEIKAKIMDQVLANAFWTL
TDDETQEIDIMEGYGSDRGGTWFAQRMHLSHHTFIRNPFTDYQPMGDATWYYNGGTPWRSAYHRYGCYWK
DPFTLEYYIDGVKVRTVTRAEIDPNNHLGGTGLNQATNIIIDCENQTDWRPAATQEELADDSKNIFWVDW
IRVYKPVAVSGGGNNGNDGATEFQYDLGTDTSAVWPGYTRVSNTTRAGNFGWANTNDIGSRDRGASNGRN
NINRDINFSSQTRFFTQDLSNGTYNVLITFGDTYARKNMNVAAEGQNKLTNINTNAGQYVSRSFDVNVND
GKLDLRFSVGNGGDVWSITRIWIRKVTSNSANLLAAKGLTLEDPVETTEFLYPNPAKTDDFVTVPNSEIG
SSIIIYNSAGQVVKKVSVVSENQKISLEGFAKGMYFINLNGQSTKLIVQ

>CAZ97711.1
MYLIYLRLVFCCALLLGCGDNSKFDSATDLPVEQEQEQETEQEGEPEESSEQDLVEEVDWKDIPVPADAG
PNMKWEFQEISDNFEYEAPADNKGSEFLEKWDDFYHNAWAGPGLTEWKRDRSYVADGELKMWATRKPGSD
KINMGCITSKTRVVYPVYIEARAKVMNSTLASDVWLLSADDTQEIDILEAYGADYSESAGKDHSYFSKKV
HISHHVFIRDPFQDYQPKDAGSWFEDGTVWNKEFHRFGVYWRDPWHLEYYIDGVLVRTVSGKDIIDPKHF
TNTTDPGNTEIDTRTGLNKEMDIIINTEDQTWRSSPASGLQSNTYTPTDNELSNIENNTFGVDWIRIYKP
VEK

>ADY30677.1
MKKISLLSIIMFLQLLFVNAQYDWDNIPIPANAGSGKTWQLQVQASDDFNYQFLPSTNVTDFGPGNNKKW
YNKYHNLPSGAPNNFEGPGPTKWMQNHVAVSGGHLNIWASRIPGATKSFVGSSGNLISRPETRAGCITNK
TRVKFPVFVEASIKVMNSSLASDIWLLSPDDTQEIDIIECYGGPGDDNRNGFFANKIHLSHHVFIRPPNF
KDYQPADFNSWWQKNGVNQWGGRVVRIGVNWVSATRLEYYVDGQMVRVLDDTAVQTRLADGTWQYTYPAG
VTSTGVNGQLVRENGYQKMNIATSLNDAKNKSNISVIDPFNYLNNQRRFTKEMDIIINVEDQSWQAEANR
SATDKELENFENNNLLVNWIRVYKPVNASNFVNTEISNNSARTLTFNEEEKQELAVFPIPAKDVVNISSK
DYVQAKVYNLQGLIVLRKDVADGTLNVSSLEKGIYVLELAKANGESTKQKIVISK

>ADY28264.1
MKNKILTLISITLCSFCFSQNNSVQIDFTTQKFIGDKSELSREKYFAMHASYNDGALADDPNYLFNDLGI
KFGRTFAGPGPFSKYKKGDLSVNNATKLALANAQRFKKAPLFATQKTTDLIITNHPRDAYKLGMDFEKAA
TFNVAYIKKAYPVMPKYYEVMNEPFVHAKDFVDSYDKTPKVISEMSEFHKIVADKVHAEIPNIMVGGYSA

AWPEYDKNNFAIWNTRMKVFMDTAGESMQFFATHIYDGRNVEGDFNYRSGSNSEAILDLIESYSYQKWGV
VKPHLISEYGYTAKGLQGQPYSAALNGICLTSYNNILMSLLDKPDRLLKAVPFITGKATWFYKDSRNPDG
HPYPWVLLRKGKNGAYKYTHLRKFWQLWKGVQGKRIYATSNNPDIQVHAFANKNKGYVALNNLAGDAQTI
NIDYLNTSNQLVNNLTIKRSYNNKNGIPKLVFFTNEKAVKELTLKPGETIILDYDLADYTFSNVIKENNY
YAKTCLQEIKANAPITFTVNNVISTNKGNATLKMGLGRAHNLSKKPIVKINGIKVTVPNNWAGYDQAGRE
QFFGVIPIPLTVSNIKNGVNTVELTFPDSGGYVSSIILNTENLKK

>ADV51223.1
MKKINLLTLLALFQILLVSSQNDWDNIAIPANAGNGKEWQLQEKPSDNFNYTFNPTNNVVDFGPAGNAKW
YNKFHNQPNGEPNNWPGPGPTVWRQDHVAVSGGSLNMWASRIPGATKSFTGSTGASIARPETRSGCITNK
TRVLYPVFVEASIRVMNSSLASDIWLLSPDDTQEIDIIECYGGPGSDNRNSFFSSKIHLSHHVFIRPPNF
KDYQPADLNSWWTKNSVAQWGGRNVRIGVNWVSPTRLEYFVDGQMVRVLDNNATQTRLGDGTWQYTYPAG
VTSTGVNGQLIRENGYQKMTVASSLNDAKTKSNISVIDPFNYLNNGRKFSKEMDIIINVEDQNWQAEANR
SPTDAEMTNFENNNLLVDWIRVYKPVNISGGSNQNTGATSGFYDFGAISSAIFNGYTRVSYGINDGFWTN
VGNLNYADRGATTGTNALNRDFAYDQNRTRTLQFPVANGTYNITATFGDRLSARVNNSIRSGGKTATVTT
KIAEYKNANLNNIVVTNGLLNVEVFASANQSWALNRLTFTRSQTNLRVSSKAMVESATEASDNFEVYPNP
ASGVLNISSANYVNAKIYNLNGLTILRKDIADKKIDISSLKDGIYILEINAISGEVIKQKIVIAN

>ADV49782.1
MKQVITQLIIGLVFSVSFAQNNEVFIDFTTQKYIGSESELQREKYFAIHASYNDHEIAADPDYLYDNLGI
EFGRTFGGPGPYANHKKGDLSIENAERLAKSNAQRFLNSPLYKDYKTTDLIITNHPRDAFQLKKDYEKAA
AFTIAYLKNAYPEMPKYYEVMNEPFVHAKDYVKTYDETDEVILEMSKFHKLVADKVHAEIPNIMVGGYSA
AWPEYDKNNFAIWNSRMKVFMDTAGESMKFFATHLYDGRNVEGDFNYRSGSNSEAILDLIESYSYQKWNL
IKPHLISEYGYTAKGLQGEPYSPTLNGVCLMSYNKILMSLLDKPDRLLKAVPFITGKATWFYKDDRNPNK
HPYPWVLLKKTEDGSYEYTHLKKFWELWKGVEGKRIHVQSMNPDIQAHAFLNKNKAYIALNNLADEPQSV
PLNFLNGSQNLIENITIRRSYTTASGFPKLVYFTDINDKTNITLKVGETVIIDYDLKENTFQNSITERNY
YSKSCLKKIEANKTLTFKINNVISGEKGVATLKMGLGRTHELSKKPILKINGTTVTIPDNWAGYDQAGRE
QFFGVIPVPTSLKNIRTGENIVELTFPDSGGYVSSIIVNTEVYLN

>AIA22720.1
MKRLLTILILLTYQLQAQDWAQIPVPANAGEGNVWELQDNVSDDFNYSFESTNSKVNFGDDKWYNFYHNH
WDGPGYTYWKHGNVTVEGGNLVIEAGYTSETNKGGTYGVASGCVTSNAKVVYPVYVESAISVANISLASC
FWLLSPDDTEEIDIIENYGNVNFYKQYTHISHHSFIRQPFTDYQPRDWNSWYPDSRVNNNYGWGDWCWNE
GQGRRYMRMGVYWKSPKHFEYYIDGELIRVMYYNAIATNYNGTWEYTYFKSLEWEVNGYKLPTNISSGAQ
NGYTDVIVHSTSSSFDFDKLKEASNASNGHSVIDPAWFQGGDDNDDDNNGVTVEARGFTKEMDIIINVES
QGWLLDQTPSESDLNNKNKNQMRVDWVRVYKPVTDNGGVRPVLGIELQPSEKTLGKGNKLNLSPIFSPSN
ATNQNVTFTSNNENVATVNDKGVVSGVDLGEALITVTSEDGGFTSTSKIIVIENGKGSEFIIEAEDFTST
GGTFDDGEVPFGMFSNDIGVNFVNKEDWAEYTVTVPEAGNYSITYLISTPMDNANISIFDNDTQIGESTR
VEKTGGWETYENQTASFIAQLEAGETTFKILASGSDWQWNLDKIVFSPYVDNTIAVEDINIDQTSASIER
YTTLQLTATIVPEDATNKKFTWSSSNAFVASIDENGLVTANSEGEVEIFATTEEGNIRAISKIMVTSASN
IPVSGLTLSSYSEDIKINSSKTITATVLPSDASNKNVSWLSSDESVATVENGKIKGVSVGSCTITSKTEE
GEFTQTISVNITADTPTSIHDLNNNSLVIYPNPNVGKFKIKGLEVGVYSIKVLNIYGSNVRSLDKVRIEN
EIEIDLTNVPVGIYIIQVTDGKDKKFIQRIIKE

>AME16507.1
MRKKANKLTWLIGVITIGTLFFLAFNPPKSIDEHSLIDFSSEEISNQIKAKDATFELKNNTLIVRNGFTK
QESGVVIYESNNHPWNLEGMYTIEAEVENLGEEYIQVEMFVGDNVDEKGLIRWYCSDYVDLEPGESGKII
VPLAWSPWVFDPQPQYYPGMRGMFGMLKRDVTTIKEITFNSRYSYKENTFAVKKLSAKQLLKKRKPDNVI
PFVDQYGQSKYTDWKGKIHSDRELNQSITNEEKDYLAHQEAPSRSKFGGHIGYENFEAKGHFYTKKHQGK
WWLVDPEGHLFWSSGLNCVNNHSMSTGITGREELFSYLPKGDDPLKKFYSTSKWKPLGFYQQFDEYETFN
FYQANLYRKYGDQWKNKFAELANKRIKSWGMNTIGFVSDKQTIESHKNPYVGSVWIRDTQKIEGSNGYWG
KFHDVFAKDFKSKVKESVADQSLGANDPWCIGYFVDNEMSWGNIGSLAIATLKSPESQPAKKEFISDLKQ
KYKKIEKLNKQWATNYASWKALSKNTNPKLGDGANEDVYAFYQKIAITYFKTIHDELAKVAPNQLYLGCR
FAWGNNAIVMKSAAQYCDILSFNKYEYSVKHVSLPEDVDMPILIGEFHFGAIDRGSFHPGVKVAKDQNDR
GEKYISYIQSALNHPNIIGAHWFQYTDQPLTGRGDGENYNVGLVDVTDQPYQEVVDKFREINYQLYDYRL
GNVNN

>WP_058105461.1
MKFKFLILSSLFLFLLTASNCNSSSDVNETPDVEVDNKDPKEGEEKEEETEEDYSTMAVPADPGSGLEWK
FQDFSDDFEYEAPAGNKGDAFFNKWDDFYHNNWSGPGLTEWSRDIPFVADGLLQIPAKRKAGTNKISTGC
ITNKTRVQYPVYVEARAKIMNSVLANGIWMLSPDDTQEIDIMEAYGAAYSESAQASHSWYEVRMHISHHV
FIRDPFQDYQPKDAGSWFMKDNIKWREDYHTYGVYWRDPWHLEYYIDGELIRTVSGKDQIDPLFYTNATN
PGDTSSDTRTGLSKEMDIIIDVEDQDWRSSPASGNQSDTYTPTDNELSDTEGHTLKVDWIRIYKPVEK

>KSA12273.1
MKFKFLILSSLFLFLLTASNCNSSSDVNETPDVEVDNKDPKEGEEKEEETEEDYSTMAVPADPGSGLEWK
FQDFSDDFEYEAPAGNKGDAFFNKWDDFYHNNWSGPGLTEWSRDIPFVADGLLQIPAKRKAGTNKISTGC
ITNKTRVQYPVYVEARAKIMNSVLANGIWMLSPDDTQEIDIMEAYGAAYSESAQASHSWYEVRMHISHHV
FIRDPFQDYQPKDAGSWFMKDNIKWREDYHTYGVYWRDPWHLEYYIDGELIRTVSGKDQIDPLFYTNATN
PGDTSSDTRTGLSKEMDIIIDVEDQDWRSSPASGNQSDTYTPTDNELSDTEGHTLKVDWIRIYKPVEK

>AIZ41814.1
MIKLKLVAFVLIFQVLSCSKSNGEEKQTVEEESQEETVTPEEEVEAEVEEETTADWKTIPVPADAGTGNV
WEFQELSDDFDYDAPADAKGTEFDKKWTDFYHNQWKGPGLTEWRRENSLVADGNLQMIANRAEGSNKINL
GCITSKEQVVYPVYIEANVKIANTTLASDVWLLSSDDTQEIDIVEAYGASYSELADSDQTWYAERIHISH
HMFIRDPFQDYQPTDPGSWYRDGTLWREDYHIVGVYWKDPFHLEYYIDGKLVRTTSGSEMIDPNNFAEGK
GLYKPMDIIINAEDQTWRSDKNITPSDKELENKENNTFEVDWIRIFKPVPAN

>WP_010925646.1
MKKTYLGLALLFIAYQSTLAQSQPTVNEGEPVAQLEWELVPELSDEFGGRSLDEDKWINADPNGWRGRAP
GLFKANTVSVKRGKLSVTTYKLDESEEVNGKEFTHAGGHIQSNNPAAVGQYFECRMKANKTFMSSTFWLI
NDRSKEEGCDKRVTELDIQESVGQITTDAEWAQDFNTKMNSNTHSRHVVCNEPEGIKGNKAKLPSKVYND
YHVFGAWWKSPKEIQFYMNGKHVGTVEPAADFDIPMYLKMVIETYDWNPVPEDGGMNMKKSDRTTYYDWV
RTWRPVNQ

>WP_013994901.1
MYLIYLRLVFCCALLLGCGDNSKFDSATDLPVEQEQEQETEQEGEPEESSEQDLVEEVDWKDIPVPADAG
PNMKWEFQEISDNFEYEAPADNKGSEFLEKWDDFYHNAWAGPGLTEWKRDRSYVADGELKMWATRKPGSD
KINMGCITSKTRVVYPVYIEARAKVMNSTLASDVWLLSADDTQEIDILEAYGADYSESAGKDHSYFSKKV
HISHHVFIRDPFQDYQPKDAGSWFEDGTVWNKEFHRFGVYWRDPWHLEYYIDGVLVRTVSGKDIIDPKHF
TNTTDPGNTEIDTRTGLNKEMDIIINTEDQTWRSSPASGLQSNTYTPTDNELSNIENNTFGVDWIRIYKP
VEK

>WP_007560915.1
MKRKLFTICLASLQFACAAENLNNKSYEWDIYPVPANAGDGMVWKLHPQSDDFNYIADEKDKGKEFYAKW
TDFYHNHWTGPAPTIWQRDHVSVSDGFLKIRASRPEDVPLKKVVSGPNTKELPGTYTGCITSKTRVKYPV
YVEAYAKLSNSTMASDVWMLSPDDTQEIDIIEAYGGDRDGGGYGADRLHLSHHIFIRQPFKDYQPKDSGS
WYKDDKGTLWRDDFHRVGVFWKDPFTLEYYVDGELVRTISGKDIIDPNNYTGGTGLVKDMDIIINMEDQS
WRAVKGLSPTDEELKNVEDHTFLVDWIRVYTLVPEE

>WP_038234083.1
MYLNHLKLVLCCALLYSCGTSSKFDDSAELPVEGEKKEEQEIEPEAAEEVVKEVDWKDIPVPADAGTDMI
WEFQEISDDFEYNAPGDNKGDEFFDKWDDFYHNGWAGPGLTEWKREFSYVANGHLQMWATRKPSSNKINM
GCITSKTRVVYPVYIEARAKVMNSTLASDVWLLSPDDTQEIDILEAYGADYSESAQKEQSYFSKKVHISH
HVFIREPFQDYQPKDAGTWFEDGTVWNQDFHTYGVYWKNPWYLEYYIDGVLVRTVSGKDIIDPKHFTNIT
DPGNTEVDTRTGLSKEMDIIINTEDQTWRSTPASGLQSQTVTPTDKELENIDNNTFRVDWIRIYKPVSTK

>WP_034666562.1
MIKLKLVAFILIFQILSCSKSNGEEKQTVEEESQEETVTPDEEVEAEVEEETTADWKTIPVPADAGAGNV
WEFQELSDDFDYDAPADAKGTEFDKKWTDFYHNQWKGPGLTEWRRENSLVADGNLQMIANRAEGSNKINL
GCITSKEQVVYPVYIEANVKIANTTLASDVWLLSSDDTQEIDIVEAYGASYSELADSDQTWYAERIHISH
HMFIRDPFQDYQPTDAGSWYRDGTLWREDYHIVGVYWRDPFHLEYYIDGKLVRTTSGSEMIDPNNFAEGK
GLYKPMDIIINAEDQTWRSDKNITPSDKELENKENNTFKVDWIRIFKPVPAN

>WP_034645118.1
MMKFNTAIVFLLFYVVSCSDSNSTDKVEDEEVIETPETPTEEPEEEPEVTWKTIPVPAEAGEGNVWEFQN
MSDSFDYEAEAANKGAEFDAKWTDFYHNQWSGPGLTEWKRDHSLVKDGYLQMVASRVPNSNKIHLGCITS
KEQVIYPVYIEAYVKIANTTLASDVWLLSSDDTQEIDIVEAYGASYSELAQADQTWYAERIHLSHHMFIR
EPFQDYQPTDAGSWYKDEVGTLWREDFHRVGVYWRDPFHLEYYINGELVRTTSGAEMIDPNNYADGEGLS
KPMDIIINAEDQTWRSDKNITPSDAELANKENNTFKVDWIRIFKPVKAD

>WP_025018818.1
MKRKLFTICLASLQFACAAENLNNKSYEWDIYPVPANAGDGMVWKLHPQSDDFNYIADEKDKGKEFYAKW
TDFYHNHWTGPAPTIWQRDHVSVSDGFLKIRASRPEDVPLKKVVSGPNTKELPGTYTGCITSKTRVKYPV
YVEAYAKLSNSTMASDVWMLSPDDTQEIDIIEAYGGDRDGGGYGADRLHLSHHIFIRQPFKDYQPKDPGS
WYKDDKGTLWRDDFHRVGVSWKDPFTLEYYVDGELVRTISGKDIIDPNNYTGGTGLVKDMDIIINMEDQS
WRAVKGLSPTDEELKNVEDHTFLVDWIRVYTLVPEE

>AIY13463.1
MIKLKLVAFVLIFQVLSCSKSNGEEKQTVEEESQEETVTPDEEVEAEVEEENTADWKTIPVPADAGAGNV
WEFQELSDDFDYDAPADAKGIEFDKKWTDFYHNQWKGPGLTEWRRENSLVADGNLQMIANRAEGSNKINL
GCITSKEQVVYPVYIEANVKIANTTLASDVWLLSSDDTQEIDIVEAYGASYSELADSDQTWYAERIHISH
HMFIRDPFQDYQPTDPGSWYRDGTLWREDYHIVGVYWKDPFHLEYYIDGKLVRTTSGSEMIDPNNFAEGK
GLYKPMDIIINAEDQTWRSDKNITPSDKELENKENNTFKVDWIRIFKPVPAN

>KGK30992.1
MIKLKLVAFILIFQILSCSKSNGEEKQTVEEESQEETVTPDEEVEAEVEEETTADWKTIPVPADAGAGNV
WEFQELSDDFDYDAPADAKGTEFDKKWTDFYHNQWKGPGLTEWRRENSLVADGNLQMIANRAEGSNKINL
GCITSKEQVVYPVYIEANVKIANTTLASDVWLLSSDDTQEIDIVEAYGASYSELADSDQTWYAERIHISH
HMFIRDPFQDYQPTDAGSWYRDGTLWREDYHIVGVYWRDPFHLEYYIDGKLVRTTSGSEMIDPNNFAEGK
GLYKPMDIIINAEDQTWRSDKNITPSDKELENKENNTFKVDWIRIFKPVPAN

>AIM59349.1
MMKFNTAIVFLLFYVVSCSDSNSTDKVEDEEVIETPETPTEEPEEEPEVTWKTIPVPAEAGEGNVWEFQN
MSDSFDYEAEATNKGAEFDAKWTDFYHNQWSGPGLTEWKRDHSLVKDGYLQMVASRVPNSNKIHLGCITS
KEQVIYPVYIEAYVKIANTTLASDVWLLSSDDTQEIDIVEAYGASYSELAQADQTWYAERIHLSHHMFIR
EPFQDYQPTDAGSWYKDEVGTLWREDFHRVGVYWRDPFHLEYYINGELVRTTSGAEMIDPNNYADGEGLS
KPMDIIINAEDQTWRSDKNITPSDAELANKENNTFKVDWIRIFKPVKAD

>WP_029446906.1
MIKLKLVAFVLIFQVLSCSKSNGEEKQTVEEESQEETVTPEEEVEAEVEEETTADWKTIPVPADAGTGNV
WEFQELSDDFDYDAPADAKGTEFDKKWTDFYHNQWKGPGLTEWRRENSLVADGNLQMIANRAEGSNKINL
GCITSKEQVVYPVYIEANVKIANTTLASDVWLLSSDDTQEIDIVEAYGASYSELADSDQTWYAERIHISH
HMFIRDPFQDYQPTDPGSWYRDGTLWREDYHIVGVYWKDPFHLEYYIDGKLVRTTSGSEMIDPNNFAEGK
GLYKPMDIIINAEDQTWRSDKNITPSDKELENKENNTFEVDWIRIFKPVPAN

>WP_025764950.1
MFKAPVLFFLAAAYFSCKTNPVSTSSTDNEIDSIRYDWSDIPVPAQPGPGMKWELQHPSDDFNYLAEASD
KGTEFSKKWTDFYHNNWTGPGLTIWDRAHSLVADGRLQIPASRVPGTNKVHTGCITSKERIVYPVYVEAY
AKISNSTLASDVWLLSPDDTQEIDILEAYGSSYSENTKKDLSWFAQRIHISHHVFIRSPFQDYQPTDPGS
WYYDGTLWREQYHRFGMYWKDPLHLEYYIDGKLVRTVSGLDIIDPKNYTGGTGLNKAMDIIINVEDQTWR
SDQGLTPTDNELANRDDQTFKVDWIRVYKPVKNQ

>WP_025615691.1
MIKLKLVAFVLIFQVLSCSKSNGEEKQTVEEESQEETVTPDEEVEAEVEEENTADWKTIPVPADAGAGNV
WEFQELSDDFDYDAPADAKGIEFDKKWTDFYHNQWKGPGLTEWRRENSLVADGNLQMIANRAEGSNKINL
GCITSKEQVVYPVYIEANVKIANTTLASDVWLLSSDDTQEIDIVEAYGASYSELADSDQTWYAERIHISH
HMFIRDPFQDYQPTDPGSWYRDGTLWREDYHIVGVYWKDPFHLEYYIDGKLVRTTSGSEMIDPNNFAEGK
GLYKPMDIIINAEDQTWRSDKNITPSDKELENKENNTFKVDWIRIFKPVPAN

>WP_024481472.1

MIKLKLVAFILIFQILSCSKSNGEEKQTVEEESQEETVTPDEEVEAEVEEENTADWKTIPVPADAGAGNV
WEFQELSDDFDYDAPADAKGIEFDKKWTDFYHNQWKGPGLTEWRRENSLVADGNLQMIANRAEGSNKINL
GCITSKEQVVYPVYIEANVKIANTTLASDVWLLSSDDTQEIDIVEAYGASYSELADSDQTWYAERIHISH
HMFIRDPFQDYQPTDAGSWYRDGTLWREDYHIVGVYWRDPFHLEYYIDGKLVRTTSGSEMIDPNNFAEGK
GLYKPMDIIINAEDQTWRSDKNITPSDKELENKENNTFKVDWIRIFKPVPAN

>AIA22721.1
MKKIYHSISKVLACVSILAFSQETTAQTAVTIDFQTQRYLDQVSELDRSKFFTIHASAGKDYSDQDMAHL
KDWDVKFGRSFWGAFGRYNDYISSNQVFPTDDKIISDGIKNINNTKKQAFYKHITRQLVVTDHPKSMIHM
DLDTKEAGAFAAKYFKYNYEDELRPMFYEPVNEPFVHAREFMGDNYQESEVRRKTAEYFKEIGKAFDEEG
IDTYVIGYSSAWPSMELWDFGHWESRMKMFMDVAGDYMDGFSTHLYDGINVTGQDTERSGSNADAILDLI
ETYSYIKWGTIKPHAITEYGGISQGYGDTYTDVESIQTVRSINHMIFGLMDREDRLMISIPFITGKSSWY
YEANDYEPYGANLWKPVKSSVVNGVIHEYDFTEKVMFYDLWKNVSGKRVRCSSSNPDIFIQAFVDEDKAY
VCLNNIATDDQDVSMQFISSVGDVEKINKRSLKIYQNEKAIYEDVDLDNQPESITLQPHETVILTYDLKN
NVTFHKKAIRKEYYSKTYMQPIESNETLSFEFNDVELNTAHGEARLRMAVGRTHNLTKRPTIKVNGTKVN
ILSNWKGYDQANRDDFFGVIEVPIPHQLLTVNNKVDISFPDEGGTLSSIILSVNNIYDLSSRLPFKGQVF
ELPQKIEAEDFDLGGQGVAYYSTDNINSIGAYRINEGVNIDKNGDAFEINLKQNEWTSYSVEVPESAIYT
VTVNTSTISTESQIDLIVDNEKICETIEVNNSDNALKEVKVELAKGIYPIHVLGNGDVKIDFLDFSNAEL
LTDDIKFTSNNSEIKTGETNNFEIEYTAANDRDIVLELWGDSFLLGQTRVNVAAGSSSINVPLDVYAEPL
NYENVELVAKVVKVNSNSLNSFAETVFNEFEFDPVLSVSDDLLDEDLITVFPNPVLNNQKLGIRGENKIY
RIDILNTQGQVFDSFEFQQPSLQVSLSLNNQYSRGIYLVRIYNENGSYTKKIMIQ

>EWH13636.1
MKFNTAIVFLLFYVVSCSDSNSTDKVEDEEVIETPETPTEEPEEEPEVTWKTIPVPAEAGEGNVWEFQNM
SDSFDYEAEAANKGAEFDAKWTDFYHNQWSGPGLTEWKRDHSLVKDGYLQMVASRVPNSNKIHLGCITSK
EQVIYPVYIEAYVKIANTTLASDVWLLSSDDTQEIDIVEAYGASYSELAQADQTWYAERIHLSHHMFIRE
PFQDYQPTDAGSWYKDEVGTLWREDFHRVGVYWRDPFHLEYYINGELVRTTSGAEMIDPNNYADGEGLSK
PMDIIINAEDQTWRSDKNITPSDAELANKENNTFKVDWIRIFKPVKAD

>WP_008615257.1
MKIQILIYLLCLLQISACDSSSVNEDEEIIPEEEVVGDPTESYTYGSKEVPANTGNPPIGTNWEVVGDFS
DEFNYEGKQSEFTQNWNDTYFNAWKGPGLTEWTSENSAVSNGNLIISASRKPNTDQVYCGVISSKKKIKY
PIYSEVRAKIANQVLSSNFWFLSEDDEREIDVLECYGSDRPDQTWFAARASSNTHVFIRNEESNTIIEDI
NQQTHHTLPNEEAWRNDFHRFGVYWKDPFTLDIYYNGVLVDEIRTDDIQDPEGLGIDREAFMIIDIEDHA
WRSSQDPPIVATDEELNDANKNKYLIDYVRTYSPTQSYDGGLLKNGTFNQSELTHWYWKGEVSVVTNTSI
NLQEAYTLQVNNNASVIQKVTVSPNSDYRLQWKHIASEKGATLSIIGIKEHSLTTTDKWNSEELLFNSGE
KSEVFIKVENKANQALYIDAFKLNKK

>WP_013620028.1
MMKFNTAIVFLLFYVVSCSDSNSTDKVEDEEVIETPETPTEEPEEEPEVTWKTIPVPAEAGEGNVWEFQN
MSDSFDYEAEATNKGAEFDAKWTDFYHNQWSGPGLTEWKRDHSLVKDGYLQMVASRVPNSNKIHLGCITS
KEQVIYPVYIEAYVKIANTTLASDVWLLSSDDTQEIDIVEAYGASYSELAQADQTWYAERIHLSHHMFIR
EPFQDYQPTDAGSWYKDEVGTLWREDFHRVGVYWRDPFHLEYYINGELVRTTSGAEMIDPNNYADGEGLS
KPMDIIINAEDQTWRSDKNITPSDAELANKENNTFKVDWIRIFKPVKAD

>WP_013551224.1
MIKLKLIALILIFQIASCSKSSTDEKPTSEEDNQGEVITPEEEVEEEVVKDWKTIPVPADAGAGNVWEFQ
ELSDDFDYDAPADAKGAKFDKKWTDFYHNLWAGPGLTEWRRDHSLVVDGNLQMIANRAEGSNKINLGCIT
SKEQVVYPVYIEANVKIANTTLASDVWLLSSDDTQEIDIVEAYGASYSELADSDQTWYAERIHISHHMFI
RDPFQDYQPTDAGSWYRDGTLWREDYHTVGVYWKDPFHLEYYIDGKLARTTSGTEMIDPNNFAEGKGLYK
PMDIIINAEDQTWRSDNNVTPSDKELENKENNTFKVDWIRIFKPVPAN

>WP_010179387.1
MNKTITFTLLFLINNLLFSQDWKNIDVPVKLDEGYKWKLHPQSDDFNYQALGNSKNLTFKNRWVDSFHNP
WKGPGLTEWNENFSEISDGYLKIKAGQKKGSKKINTGCITSKVRIKYPVFVESKVKISNSSLSCAVWMLS
PDDTQEIDIIEAYGSDNQNNEWYSKRIHLSHHMFKRSPFEDYQPTDDDSWYFSNGTTWHEKWVRVGIYWK
DPFTLEYYIDGEKVRVVEGMDVIDPNNYSETKGIYKEMDIIINAEDQTWRASNGLTPSKKELKDIEKNTF

LVDWIRIYKPVMTDEFLSLQKQQELEKNKEVNEEKAIVSNDISPSIIENRESISVNTLTETEQVAQTKAI
NNNTIPEIVKRTSIEVAKIENNSKVPTDSLKTHNNLIKENLMNQEVTVKENSINIDYQSFTMHQTKDEND
ILEITSDKYINKVQLLTHKLNLFQEFTIDNKTTKLNVSKLPKGFYYINVLFDDKLATQAYTAKD

>WP_010523252.1
MNKKVLSIFGCLSLLTSCVLAQVNKSKGKDIGKTYETTRSGAASPITGAYKNGNCVLPNSVGNPPVTLDW
EVVPTHSDEFNYSGKGAQFKKKWKDTYFNAWTGPGLTYWEAKNTNVKNGNLKVMASRKSGTNKVNCGVIT
SKKQIKYPIYSEVRAKVANHVLSSNFWFLSPDDKREIDVLEIYGSDRDDHRWFAARPSTNYHVFVREEEG
NAIIEDLNKQHHHTLPNEKPWREDWHRFGAYWKDPFTIDFYYDGKIVHELRKEGINDPEGLGMDRDSYMI
IDLEDHDWRSNAGNVATDRELADSSRDYLVDYVRTYRPIAAQEDSGLLKNGSFNDPTLTNWYWTKGATIS
ANIEENEGDVYALKLENEATVIQKISVTPSKTYELSWKIGGENGAGVMVEIYNYPIGKKVVSNGEMTYSK
LKFHSGKKEFIYVIVKGINNSGVIVDDFELIEK

>WP_010523251.1
MRSFVKKSVLAIWGSFFVFNFSYANTEDGKKKKTESKTKASVSLLIVGGSPITNDFTKGGFKITSYLGVP
PFKGTWEVIDDLTDEFNYTGKNAEFYKKWNDTYFNEWLGPGLTEWNPQNTNVIGGNLELKASRKTDTDKV
YCGVISSKKQIKYPIYSEVKAKVANQVLSSNFWFLSPDDKREIDVLEIYGSDREDHKWFAARPSTNYHVF
VRDNGNHILEDLNDQNHHTLPNDEPYREGWHRFGAHWIDPFTVDFYYDGKVVRKLRKEKVNDPEGLGMDR
DSFLIIDLEDHDWRSNQGFFPTDEELSNPAKNKYLVDYVRTYRPTRSFRSQGGLIQNGSFDEPDLNQWYW
NKKVKISADLNDNGGEVFSVNLAGTGAKIIQEVEVEKNTLYKLSYKVKGAGGKALVGVYTIDEEKVESNE
GWTYETLSFNSENNKVIYVTAENISKTNILIDSFKLKKIK

>WP_010522361.1
MKKRILVSSLLGLVSIVMNGQDWASTPIPAYPGPGKVWKLQDQHSDDFNYNGKNQKFYKNWNDHYFNAWS
GPGLSNFTSANSHVADGNLIIKASPNGNQRVYCGVVTSKTKVKFPIYMEARLQTMNQTLSSNFWMLSQND
RQELDAVESYGSDRSSFGDEHVFDSRHMNSNYHIFDRNHENNTIINDNTWQRDHYVTDHQPLKNRPALRN
GFHNYAIHWIDEWNIDWYLDGKLVRRIAPGTNDVIKDPLQNKGIYEEMFMILDVEDHDWRSNAGHIATVK
ELSDESKNKMYVDWVRVYKPVNGRGTPAPKNIFSDNNVPVKGITVTPIEISMLTGDTKFVGGAVIPAFAT
DQTVSFTSSNPSVATVNNGGAIVGVSAGTATIWGKTTDGGYVGQTQVRVAQNNKPKVAVSGVAITPSNIQ
IGVGGTRQVAGRVIPAEATEKSVYFVSQNTAVATINAFGIVTGVNPGTTTITVTAIDGNFTNTATVRVTG
TAVVVTPVGPKPSQNAVPVKGITLSPNSLKVQIGTTKQLTGRVSPNNATDKTMTFTSSNPAIVKVNQSGK
VTALRRGKATITATSTDGNFKDSMTVVSVILDTTNPTSNTPDAAPTKVTGVILTPTVLNVAVGQTKQFAG
TVQPSTAADKRVFFTSSNTAIATVNQLGEVTGLKNGNVTITATSVNGNFTDTANVSVGNAKASFPNNTKP
VTTVSSTTYYLQNRYTGKRIGVTNNANGTVVVQTPVNVSNTRNQWKKIDTKDGYFYLQNIASGKNFTPIN
EFSGSWLKQEAEAGNEGLWKIVTTDNGYFHLENKATHMYVRPINYDDTANATGNNYQIIQRPTSAKGWWT
QWAFIPVKSGAKEIDELSIATKIYPNPATDLVTVGLTEVFANENATITLVTINGSIVKSQNYSETTTINV
ADLATGVYFIVIDVNGRKVSKKLIIE

>WP_010521893.1
MKLTKLLAFTLMSASFATSVSAQDWKNRPVPANPGDGNVWKLQESYSDDFNYSGKNAKFRKNWRDTYFHS
WTGPGLTQWQSNHSDVSGGNLVIRASRHGANRVNCGVVTSKTKVIYPIFMEARVKVSNLELSSNFWMLSE
DDRREIDVLEVYGGASDVWFAQRMSTNFHMFVRGGGNQIISDFNDQNHVTLPNNELWRNKFHTFGVYWKS
PQEVYFYIDGKQTKKGSWQESRFVDKDYTGAVMDKNKFNMDRPVFMIIDTEDHSWRSAQGIVASDADLRN
PSKNKMLVDWVRTYKPVKKNSVQPSNPVVSNPPSNGNGKPSLNISAPANNANIRIGSDLVVRATASDNDG
IANLKLYINNQFVRQENIAPYEWGNTNQDGILKNMKLGEYTLKIIATDKKGATRERSINVSVTNRVNVPT
NPGNNRNQLIANGLHTIKDPFKNQAVLARELDNYNATMIDQGNYTDQQWVFTHLGNNTYTIKNNRNNRYL
EVPFGECKNAANVGTWTGSAQNHQKWKVEKNGKYYALKPLHCTSQALDRAGGLVDANLTTWAYNSSNNNQ
KWEISGTSSGLKVLDDSAIALEVYPNPSTVGGTITLAGINAKASDATVVVYSVSGQVVKTLAVDASGTQD
LSLNGISTGIYFVKVGNQAAAKLIVE

>WP_010521157.1
MKFNLLLISLGVSMLIACNGKSKQNEQNNSENESKDTTPVVEMEFQKIPVPANAGDSMKWELQTDTSDDF
NYLHKPATIRTDFGDGKWYNFYHNAWNGPGTTYWQYDHVEVNGDDLIIKSSRNPSTEKMGVPGVNAGCIT
SNKKVKYPVFVEASISVANIALASDVWLLSPDDTQEIDIIECYGGKEPKNEYFSQFIHLSHHSFIREPFT
DYQPTDLKSWWRRNGVTSWGEYSWNNGNRKYVRVGVNWVSPIHFEYYIDGELVRVLYDKAMANKINGKWD
YDYPSMTDGKLDMDAGYQKLNNFETSTTYSLATLKKASEQSKVNVIDPYNFQKGTGFNKEMDIIINVESQ
NWHVLDKRTPSDEDLADPNKNSMKVDWIRVYKPIASNISA

>WP_010182338.1
MEFKKLFALALMSSSLLTQAQDWKNTPVPADPGQGKKWELQKGYSDDFNYNGKNAQFRSNWRDAYFHSWT
GPGLTQWQSDHSDVKGGNLIISASRHGANRVNCGVVTSKTKVIYPIFMEARIKVSNLELSSNFWMLSEDD
KREIDVLEVYGGASDVWFAQRMSTNFHVFERGAGNRILSDYNDQNHVTLPNNELWRNKFHTFGVYWKSPS
EVYFYIDGKQTKDGSWAQSDMFDKDYTRRKMDKNRFKMDKPVFMIIDTEDHSWRSEQGIVASDADLKNPN
KNKMLVDWVRTYKPVKSNDTKPTPKPDPVVVNPGSGSNTKPNLNFVSPKNNATLARGKNIEVAINASDND
GIANVKLYLNNKFVRQENVTPYTWGETNQNDGILKNMKSGNYTLKAVATDKKGATSEKVIRFTVTNGTVR
PTRPTPVNNNQLVANGLYSIKNPFEDQAVLARELENYNAQMVDVGSYTDQQWIFTHLGNNVYTIKNSRNN
RYLEVPFGGCGNGENVSTWREASQNHQKWKVIKNGRYYALMPLHCTAQALDRAGGLFDANLITWAYNPSN
NNQKWEINGTAKGLKVLGDTDARTFEIYPNPAQVDQSVTISGIALGQVAVVYNVAGQVVKTVSEATTETL
VVDVTGLSAGMYFVKVGNQAAAKLIIE

>WP_010180878.1
MKFNVLMVSIGVSMLIACNGKSKQKDTPSSEKENTPPVEVLEFEKIKVPANAGESMKWELQKEASDDFNY
THKPVNQKTDFGNGKWYNFYHNAWNGPGSTYWQYDHVEVDGSHLIIKSSRNPSTEKMGVPGVNAGCITGN
KKVKYPVFVEASVSVANIALASDVWLLSPDDTQEIDIIECYGGKEPNNSYFSQFIHLSHHSFIREPFTDY
QPRDIKSWWKKDGVTSWGEYSWNNGNRKYTRIAVNWISPNHFEYYIDGELVRVLYNKAMANKINGKWDYD
YPSMTDGKLDMDGGYQKLNNLKSNDTYSFEMLKEASLQSKVDVIDPYNFQKGTGFNKEMDIIINMESQNW
HVLDKRTPSDEDLADPNKNTMKVDWIRVYKPIKK

>WP_010179868.1
MKKRLLVSSLIGLASLAMNGQDWSSTPIPAYPGPGKVWKLQNQHSDDFNYQGKNNKFYKNWNDRYFNAWT
GPGLSNFSSANSHVADGNLIIKASPNGKNRVYCGVVTSKTKVKFPIYMEARLQTMNQTLSSNFWMLSQND
RQELDAVESYGSDRSSFGDEHVFDSRHMNSNYHIFDRNANDNTIINDNTWQRDHYVTDHQPLKNRPALRN
GFHNYAIHWIDEWNVDWYLDGKLVRRIAPGTNDVIKDPLQNKGIYEEMFLILDVEDHSWRSDAGHIATVK
ELNDESKNKMYVDWVRVYKPVNGQGTPAPKNIFSSTKVPVKGITVTPIEISMLTGEKKFVGGAVIPAFAT
DQTVSFASSNPAVATVDNNGAIIGVSQGTATIWGKTTDGGYVGQCQVRVKQSNKPQVAVTGVTITPANVQ
IGVGGTRQLAGRVTPTEATDKSVYFVSQNTSIATVNQLGIVTGINPGKTTITVTAIDGFFTVAADITVTG
KAVINTPVVAAPKPANAIPVKGIALTPKNLSIQIGSIKQLAGRVSPANATDNTMVFTSSNPAVASVNQAG
QVKALKRGVVTITATSTDGNFKDSATITSVILDTTAPSPVVNNTKVSAVVLTPAKLNIAVGQTKQLAGKV
QPNTAKDKRVLFTSSNTSIATVNQLGEVTGIKNGTVIITATSMDGGFTDTSNVTIGKASPNTGANVVPNN
PTTVNTNANIYYLQNRLTGKKVGVINDAKGALVVQTPSNTTNTKSQWKKIDLSNGYFYLQNVSSGKNFIP
FNEFSGSWLKQENNSGDIAQWKIVNATDGFFYLENKESDMYIRPRTKDDTGANTGSTYQIEQRPTSFNGF
WTQWAFIPVNSGAKNIIDQNSDAVILYPNPANEVVSVKLNDLFSDAVARISLIDINGAVISTQNFTNGTD
KFDVSNLTAGVYFVVIRTNERKLSKKLIIE

>WP_010177128.1
MRSFVTKSALVILSSFFVFNSIYANGGEGEKKKSKKSKQKNKSFLLKEESPITGDFTKGSFKITSYLGVP
PFKGTWEAIDDLTDEFNYTGKNAEFYKKWKDTYFNQWLGPGLTEWNPENTNVIGGNLVLKASRKPGTDKV
YCGVISSKKQIKYPIYSEVKAKVANQVLSSNFWFLSPDDKREIDVLEIYGSDREDHKWFAARPSTNYHVF
VRDNGNRILEDLNDQNHHTLPNDEPWREGWHRFGAHWIDPFTVDFYYDGKVVRKLIKEKVKDPEGLGMDR
DSFLIIDLEDHDWRSNQGFFPTDEELNDASKNKYLVDYVRTYRPARSFRLEGGLIQNGSFDEPDLNQWYW
NKKVRLSADLNENSGEVFSVNLAGKGAKIIQEVEVEKNTLYKLSYKVKGANGGAVLVGVYSIDEEKVESN
EGWKYETLSFNSGNNKTIYITAENVSDSKVLVDSFKLKKIE

>ACY02061.1
MKNIYLILLLSFNLGHLVNAQEWSNIPVPAYAGPGNTWELQSNLSDDFNYNFNAVNYKSNFGNGKWYNFY
HNGWDGPGTTYWTYNKVKVDGDNLVITVAKSNNTSKMGIPGVFSGCVTSNNRVVYPVYVESAISVANISL
ASCFWLLSPDDTQEIDIIENYGNVPWFKQFTHISHHSFIRTPFTDYQPKDWNSWYNDNRVTANYGWGDWC
WNNGNRRYMRMGVYWVGPKHFEYYIDGQLVRVMYHNATATKVNGTWEYQYFNSMNGQFPANNASGYTAVT
TYATSTNYNFSTIQAASNNSNGISVIDPGNFQGGAGFTKAMDIIINVESQQWLALNHTPSDADLASSARN
QMKVDWVRVYKPKSSSGGGSNGTTCADAPDYNGNSNSYTAGQYVINGGILYRKRSDGQWDWIANCNSSSR
VSELVIEDIPEAQIALSPNPAKGFVKISGLGEGSYQAEIVTMQGGQILSTQTVSRATNTLSTADLSPGVYI
IKVAGEAQKLIVH

>ABW77762.1

MKGFTKHSILMACSIGLAINATAADWDNIPIPAELDAGQSWELQQNYSDSFNYSGKNSTFTGKWKDSYFH
SWTGPGLTHWSSDESWVGDGNLIISASRRQGTNKVNAGVITSKTKVKYPIFLEASIKVSNLELSSNFWLL
SENDQREIDVLEVYGGARQDWYAKNMSTNFHVFFRNNDNSIKNDYNDQTHFTPTWGNYWRDGFHRFGVYW
KSPTDVTFYIDGQKTTKGAWSQVVMKDKDYTGAILDKSRYNMDQEAFIIIDTEDHSWRSEAGHIATDADL
ADSDKNKMYVDWIRVYKPTGGSTTPPTGDITPPSGYTNLQLAHSNRCVDVINGALWNGSTYQQYSCNTGN
NNQRFKFTKIANNQYSINAKVSQLCMELASGSSANGAKVQQWICNHANSNQTWSLEDKGSNTFEIRNKQS
GKCLEVANSSNANGGQIRQWACTGATNQRFKFL

>AAR87712.1
MKQLKLLIGSTLFMSITSVQAADWSPFSIPAQAGAGKSWQLQSVSDEFNYIAQPNNKPAAFNNRWNASYI
NAWLGPGDTEFSAGHSYTTGGALGLQATEKAGTNKVLSGIISSKATFTYPLYLEAMVKPTNNTMANAVWM
LSADSTPEIDAMESYGSDRIGQEWFDQRMHVSPHVFIRDPFQDYQPKDAGSWVYNNGETYRNKFRRYGVH
WKDAWNLDYYIDGVLVRSVSGPNIIDPENYTNGTGLNKPMHIILDMEHQPWRDVKPNASELADPNKSIFW
VDWLRVYKAQ

>ACF40223.1
MKNNLLLIGCVLTSTNLMANDWDAIPLPVAPDNGKVWQLQEAYSDSFNYTGKPAAFTSKWNDTYFNSWTG
PGLTYWQRDESWVSDGNLIISASRRAGTDQVNAGVITSKTKVTFPIFLEASIKVSNLELSSNFWLLSDND
EREIDVLEVYGGARDEWFARNMSTNFHVFIRDQQTNQIISDYNDQTHNTPSWGTYWREGFHRFGVYWKSP
TDVTFYIDGQQTPDGSWAQVVMKDKDYSGATLNKNTHNMDQSAYIIIDTEDHDWRSEAGNIATDADLADD
SKNKRYVDWVRVYKPVNAANTSSVTSGAQIKAKHSQKCIDITNGAMNNGSTYQQWNCNSNNENQAFELVE
LTNNEYAISSQLTGLCMQIANSSTSNGAGVEQWVCDHTKANQRFTLNNTGDGYFELRSSLSNKCIDIAGK
LQTNGASVVQWQCYNGDNQRFQLIE

>BAF34350.1
MKGFTKHPLLLACGLGLSLSTYAADWDNIPIPAELDPGQSWELQESYSDSFNYSGKPSSFTSKWKDAYFH
NWTGPGLTYWSSDESWVGDGNLIISASRRQGTTQVNAGGCHLKTKVKYPIFLEANIKVSNLELSSNFWLL
SENDQREIDVLEVYGGARQDWFAKNMSTNFHVFFRNNDNSIISDFNDQTHNTPTWGNYWREGFHRFGVYW
KSPTEVTFYINGQKTTKGAWSQVVMKDKDYTGAILDKSRYNMDQEMFIILDTEDHSWRSEAGHIATDADL
ADGDKNKMYVDWIRVYKPTGGSTTPPTGGINPPSGYTNLQVAHSNLCLDVKSGALWNGSTYQQWSCNTGN
NNQRFQFTALGNDEYSISAKVSQLCMELASGSSADGAKVQQWVCNHANTSQVWSLVDKGSNTFEIRNKQS
GKCLEIANDSGANGADLRQWSCDGGTNQRFKFQ

>WP_064385033.1
MNKTTLFIGCLLTTTNLFANDWDSIPLPVTPGDGKVWQLQETYSDSFNYTGKPAAFTSKWNDTYFNSWTG
PGLTYWQQDESWVSDGNLIISASRRAGTDKVNAGVITSKTKVSFPIFLEANIKVSNLELSSNFWLLSDND
EREIDVLEVYGGARDDWFAKNMSTNFHVFIRDQQSNQIISDYNDQTHNTPSWGTYWREGFHRFGVYWKSP
TEVTFYIDGQQTPDGSWAQVVMKDKDYTGATLNKNTHNMDQSAYIIIDTEDHDWRSEAGNIATDADLADG
SKNKMYVDWVRVYKPVNASNTNSVSNGAQIKAKHSQKCIDITAGAMSNGSYYQQWGCGSDNANQQFNLVE
LSNNEYAISSQLSGLCMQIENASTSNGAKLEQWVCDHAKASQRFTLNSTGDGYFELKSSLSNKCVDIAGK
LQTDGADIVQWQCYNGDNQRFQFIE

>ANB23850.1
MKYLSILLAGIITSTVAHCEDWDNIPLPTSPGGGKVWQLQTQYSDSFNYIGKPSDFTDKWNDSYFNNWTG
PGLTYWSSNESWVANGNLIISASRRQGTNQVNAGVITSKTKVKFPIYLEARIKVSNLELSSNFWLLSQND
EREIDILEVYGGAADTWFAKNMSTNFHVFLRDEQTNQIISDFNDQTHNIPSTGTYWRDQFHRFGAYWKSP
TEVTFYIDGQQTPDGSWAQVVMKDKDYTGATLDKSQYNMDQEAFIIIDTEDHDWRSNQGIVASDAELADG
SKNKMYVDWIRVYKPVDGVVTGNTNLQAKHSGRCIDVAQGAMINGSQYQQWTCDTTNTNQSFKFISAGNN
EYLIQSTQSNLCVELKDNNSANGANIHQWVCNSANDNQKWTLHDKGDQHFEIRSKVTGKCIDVAGKATTN
GANIVQWSCYNGQNQRFKFLQ

>WP_057791544.1
MKYLSILLAGIITSTVAHCEDWDNIPLPTSPGGGKVWQLQTQYSDSFNYIGKPSDFTDKWNDSYFNNWTG
PGLTYWSSNESWVANGNLIISASRRQGTNQVNAGVITSKTKVKFPIYLEARIKVSNLELSSNFWLLSQND
EREIDILEVYGGAADTWFAKNMSTNFHVFLRDEQTNQIISDFNDQTHNIPSTGTYWRDQFHRFGAYWKSP
TEVTFYIDGQQTPDGSWAQVVMKDKDYTGATLDKSQYNMDQEAFIIIDTEDHDWRSNQGIVASDAELADG
SKNKMYVDWIRVYKPVDGVVTGNTNLQAKHSGRCIDVAQGAMINGSQYQQWTCDTTNTNQSFKFISAGNN

EYLIQSTQSNLCVELKDNNSANGANIHQWVCNSANDNQKWTLHDKGDQHFEIRSKVTGKCIDVAGKATTN
GANIVQWSCYNGQNQRFKFLQ

>KYL34728.1
MNKTTLFIGCLLTTTNLFANDWDSIPLPVTPGDGKVWQLQETYSDSFNYTGKPAAFTSKWNDTYFNSWTG
PGLTYWQQDESWVSDGNLIISASRRAGTDKVNAGVITSKTKVSFPIFLEANIKVSNLELSSNFWLLSDND
EREIDVLEVYGGARDDWFAKNMSTNFHVFIRDQQSNQIISDYNDQTHNTPSWGTYWREGFHRFGVYWKSP
TEVTFYIDGQQTPDGSWAQVVMKDKDYTGATLNKNTHNMDQSAYIIIDTEDHDWRSEAGNIATDADLADG
SKNKMYVDWVRVYKPVNASNTNSVSNGAQIKAKHSQKCIDITAGAMSNGSYYQQWGCGSDNANQQFNLVE
LSNNEYAISSQLSGLCMQIENASTSNGAKLEQWVCDHAKASQRFTLNSTGDGYFELKSSLSNKCVDIAGK
LQTDGADIVQWQCYNGDNQRFQFIE

>KXI28844.1
MLKLAFLPLSLLASLSLFAADWDDIPVPATLPSGQTWQLLPLSDDFNYSAAAEGKSDEFKQRWHEGYINA
WTGPSWTEWHPSQSAVSDGVLKLTAKRKPGSWDIYMGSITSKQSVHYPLFLEIRAKLSNSVLASDFWLLS
EDSTQEIDVLEAYGGDRPGHEWFAERLHLSHHVFVREPFQDYQPKSDDTWYHTGTKWRDDYHRIGVYWRD
PWHLEYYVDGKLVKTSSGKEVIDPLDYTKGTGLSKPMRVIINMEDQQWRTEQGLTPTDEELAEREKVTYS
IDWVRFYKPVDSK

>AMJ94778.1
MKYLSILLAGIITSTVAHCEDWDNIPLPTSPGGGKVWQLQTQYSDSFNYIGKPSDFTDKWNDSYFNNWTG
PGLTYWSSNESWVANGNLIISASRRQGTNQVNAGVITSKTKVKFPIYLEARIKVSNLELSSNFWLLSQND
EREIDILEVYGGAADTWFAKNMSTNFHVFLRDEQTNQIISDFNDQTHNIPSTGTYWRDQFHRFGAYWKSP
TEVTFYIDGQQTPDGSWAQVVMKDKDYTGATLDKSQYNMDQEAFIIIDTEDHDWRSNQGIVASDAELADG
SKNKMYVDWIRVYKPVDGVVTGNTNLQAKHSGRCIDVAQGAMINGSQYQQWTCDTTNTNQSFKFISAGNN
EYLIQSTQSNLCVELKDNNSANGANIHQWVCNSANDNQKWTLHDKGDQHFEIRSKVTGKCIDVAGKATTN
GANIVQWSCYNGQNQRFKFLQ

>AMJ90896.1
MKYLSILLAGIITSTVAHCEDWDNIPLPTSPGGGKVWQLQTQYSDSFNYIGKPSDFTDKWNDSYFNNWTG
PGLTYWSSNESWVANGNLIISASRRQGTNQVNAGVITSKTKVKFPIYLEARIKVSNLELSSNFWLLSQND
EREIDILEVYGGAADTWFAKNMSTNFHVFLRDEQTNQIISDFNDQTHNIPSTGTYWRDQFHRFGAYWKSP
TEVTFYIDGQQTPDGSWAQVVMKDKDYTGATLDKSQYNMDQEAFIIIDTEDHDWRSNQGIVASDAELADG
SKNKMYVDWIRVYKPVDGVVTGNTNLQAKHSGRCIDVAQGAMINGSQYQQWTCDTTNTNQSFKFISAGNN
EYLIQSTQSNLCVELKDNNSANGANIHQWVCNSANDNQKWTLHDKGDQHFEIRSKVTGKCIDVAGKATTN
GANIVQWSCYNGQNQRFKFLQ

>AMJ87035.1
MKYLSILLAGIITSTVAHCEDWDNIPLPTSPGGGKVWQLQTQYSDSFNYIGKPSDFTDKWNDSYFNNWTG
PGLTYWSSNESWVANGNLIISASRRQGTNQVNAGVITSKTKVKFPIYLEARIKVSNLELSSNFWLLSQND
EREIDILEVYGGAADTWFAKNMSTNFHVFLRDEQTNQIISDFNDQTHNIPSTGTYWRDQFHRFGAYWKSP
TEVTFYIDGQQTPDGSWAQVVMKDKDYTGATLDKSQYNMDQEAFIIIDTEDHDWRSNQGIVASDAELADG
SKNKMYVDWIRVYKPVDGVVTGNTNLQAKHSGRCIDVAQGAMINGSQYQQWTCDTTNTNQSFKFISAGNN
EYLIQSTQSNLCVELKDNNSANGANIHQWVCNSANDNQKWTLHDKGDQHFEIRSKVTGKCIDVAGKATTN
GANIVQWSCYNGQNQRFKFLQ

>AMJ74593.1
MKYLSILLAGIITSTVAHCEDWDNIPLPTSPGGGKVWQLQTQYSDSFNYIGKPSDFTDKWNDSYFNNWTG
PGLTYWSSNESWVANGNLIISASRRQGTNQVNAGVITSKTKVKFPIYLEARIKVSNLELSSNFWLLSQND
EREIDILEVYGGAADTWFAKNMSTNFHVFLRDEQTNQIISDFNDQTHNIPSTGTYWRDQFHRFGAYWKSP
TEVTFYIDGQQTPDGSWAQVVMKDKDYTGATLDKSQYNMDQEAFIIIDTEDHDWRSNQGIVASDAELADG
SKNKMYVDWIRVYKPVDGVVTGNTNLQAKHSGRCIDVAQGAMINGSQYQQWTCDTTNTNQSFKFISAGNN
EYLIQSTQSNLCVELKDNNSANGANIHQWVCNSANDNQKWTLHDKGDQHFEIRSKVTGKCIDVAGKATTN
GANIVQWSCYNGQNQRFKFLQ

>ALO78721.1
MRALLTAVLGLSCTHALAADWDNTPVPANAGNGKVWELQAVSDDFNYSSSLNNYHSEFTSRWHEGFINPW

TGPGLTEWTDGHAYVTGGNLGIAATRKLGTDKVRAGSITSHDTFTYPLYVETKAKISKLVLASDVWLLSA
DSTQEIDVLEAYGSDRAGQEWFAERIHLSHHVFIRDPFQDYQPTDAGSWYTDGQGTVWSDDFHRIGVHWK
DPWNLDYYIDGQLVRSVSGDNIIDPNGFTNGTGLSKPMHLIINTEDQDWRSDNGISPTDAELANTNKSIY
WVDWIRVYKPVDGGDNGENTDVPASATSIKGRQSGKCIDLASGSSANGANIQQWACGTNNANQEFTFVPV
DSGWYELRTKHNKCVGVGGNSSANGAVVIQWDCFNGQNLHVKPVDLGNGYVELRARHSNKCLDVADASTA
NGADIRQWQCNGNTNQQFSFN

>GAC10035.1
MAITSAQGADWSSFSIPAQAGAGKTWELQSVSDEFNYIAPTNNKPSAFTSRWNDSYINSWLGPGDTEFSS
EHSYTTGGALGLQATEKTGTNKVLSGIVSSKATFTYPLYLEAMVKPTNNTMANAVWMLSADSTREIDAME
SYGSDRPGQEWFDQRMHVSHHVFIREPFQDYQPKDGGSWIYNNGETYRNKFRRYGVHWKDAWNVDYYIDG
VLVRSVSGPNIIDLKNYTNGKGLNSPMHIILDMEYQPWRDVKPSSAELADSSKSIFWIDWIRVYKAQ

>GAC03896.1
MAITSAQGADWSSFSIPAQAGAGKTWELQSVSDEFNYIAPTNNKPSAFTSRWNDSYINSWLGPGDTEFSS
EHSYTTGGALGLQATEKAGTNKVLSGIVSSKATFTYPLYLEAMVKPTNNTMANAVWMLSADSTREIDAME
SYGSDRPGQEWFDQRMHVSHHVFIREPFQDYQPKDEGSWIYNNGETYRNKFRRYGVHWKDAWNVDYYIDG
VLVRSVSGPNIIDPKNYTNGKGLNSPMHIILDMEYQPWRDVKPSSAELADSSKSIFWIDWIRVYKAQ

>ALQ08013.1
MNILKLLSCSTCAILCTATHAADWDAYSIPASAGSGKTWQLQTVSDQFNYQAGTSNKPAAFTNRWNASYI
NAWLGPGDTEFSSGHSYTTGGALGLQATEKAGTNKVLAGIVSSKATFTYPLYLEAMVKPSNNTMANAVWM
LSSDSTQEIDAMESYGSDRVGQEWFDQRMHVSHHVFIREPFQDYQPKDAGAWVYNNGETYRNKFRRYGVH
WKDAWNLDYYIDGVLVRSVSGPNIIDPEGYTGGTGLNKPMHIILDMEHQPWRDVKPNSTELADSNKSIFW
IDWVRVYKAN

>WP_055733246.1
MLPRHYKNLLLLTQGLSLSFASLAANDWDTIPIPANPGTGYVWELQEAYSDSFNYSGKTNEFTSKWNDSY
FKSWTGPGLTHWDSSESWVADGNLIVSASRRQGTDKVNAGVITSKTKVKYPIFLEASIKVSNLELSSNFW
LLSENDEREIDILEVYGGAADTWYAKNMSTNFHVFIRDAASNQIISDFNDQTHNEPSWGTYWRDGFHRFA
AYWKSPTEVTFYIDGQQTPEGSWAQVVMKDKDYTGAVLDKSVYNLNQEAFIIIDTEDHSWRSEMGIIASD
TDLADNSKNKMYVDWVRVYKPVTDDSSGGENGNVSVPSTYTNLQLVHSDLCLDVASGATWNGSTYQQWIC
NTGNSNQRFQFSALGNGQYAISSEVSQLCLELDQASHADGATVHQWVCNHSDSKQTWTLFDKGSSTFEIR
NKVSGKCLEIANASSNNGAPLQQWSCDGGNNQRFKFL

>WP_055024616.1
MNVIYATTLMATSALTTVCIAADWDNIPLPADPGNGLVWQLNEQYSDSFNYQGKNATFNSKWNDTYFNNW
EGPGLTRWSQNESWVSDGNLIISASRKAPNLVNAGVITSKTKVKYPIFLEANIKVSNLELSSNFWLLSEN
DEREIDILEVYGGSRETWFTQNMSTNFHVFLRNSDNSIRSDFNDQTHNTPTWGNYWREGFHRFGAYWKSP
TDVTFYIDGQETPQGSWAEVVMKDKDYTGAIMDKYQYTMNEEMFIILDTEDHSWRSEAGNVATDADLADQ
SKNKMYVDWIRVYQPVTNGGSGGDNGGNDGSIEPNTQYNFVAKHSNKCIDVINSQTYNGSQYQQASCTNT
NAQKFTLNQLSNGFYTIRSNVSNLCLDLSAGSTSNGAKIQQWVCNSSNQNQMWNLADKGDGYYEIISKVS
NKCLDIAGKSTNDGASLTQWSCYNGTNQQFKVE

>WP_055014357.1
MKNNLLLIGCVLTSTNLMANDWDAIPLPVAPDNGKVWQLQEAYSDSFNYTGKPAAFTSKWNDTYFNSWTG
PGLTYWQRDESWVSDGNLIISASRRAGTDQVNAGVITSKTKVTFPIFLEASIKVSNLELSSNFWLLSDND
EREIDVLEVYGGARDKWFARNMSTNFHVFIRDQQTNQIISDYNDQTHNTPSWGTYWREGFHRFGVYWKSP
TDVTFYIDGQQTPDGSWAQVVMKDKDYSGATLNKNTHNMDQSAYIIIDTEDHDWRSEAGNIATDADLADD
SKNKMYVDWVRVYKPVNAANTSSVTSGAQIKAKHSQKCIDIKNGAMNNGSTYQQWNCNSNNENQAFELVE
LTNNEYAISSQLTGLCMQIANSSTSNGAGVEQWVCDHTKANQRFTLNSTGDGYFELRASLSNKCIDIAGK
LQTNGASVVQWQCYNGDNQRFQLIE

>KPZ59655.1
MKNNLLLIGCVLTSTNLMANDWDAIPLPVAPDNGKVWQLQEAYSDSFNYTGKPAAFTSKWNDTYFNSWTG
PGLTYWQRDESWVSDGNLIISASRRAGTDQVNAGVITSKTKVTFPIFLEASIKVSNLELSSNFWLLSDND
EREIDVLEVYGGARDKWFARNMSTNFHVFIRDQQTNQIISDYNDQTHNTPSWGTYWREGFHRFGVYWKSP

TDVTFYIDGQQTPDGSWAQVVMKDKDYSGATLNKNTHNMDQSAYIIIDTEDHDWRSEAGNIATDADLADD
SKNKMYVDWVRVYKPVNAANTSSVTSGAQIKAKHSQKCIDIKNGAMNNGSTYQQWNCNSNNENQAFELVE
LTNNEYAISSQLTGLCMQIANSSTSNGAGVEQWVCDHTKANQRFTLNSTGDGYFELRASLSNKCIDIAGK
LQTNGASVVQWQCYNGDNQRFQLIE

>GAA77741.1
MNILKLLSCSTCAILCTATHAADWEAYSIPASAGSGKTWQLQTVSDQFNYQAGTSNKPAAFTNRWNASYI
NAWLGPGDTEFSSGHSYTTGGALGLQATEKAGTNKVLAGIVSSKATFTYPLYLEAMVKPSNNTMANGVWM
LSSDSTQEIDAMEAYGSDRVGQEWFDQRMHVSHHVFIREPFQDYQPKDAGSWVYNNGETYRNKFRRYGVH
WKDAWNLDYYIDGVLVRSVSGPNIIDPEGYTGGTGLSKPMHILLDMEHQPWRDVKPNSAELADSNKSIFW
IDWIRVYKAN

>WP_045984435.1
MNILKLLSCSTCAILCTATHAADWDAYSIPASAGSGKTWQLQTVSDQFNYQAGTSNKPAAFTNRWNASYI
NAWLGPGDTEFSSGHSYTTGGALGLQATEKAGTNKVLAGIVSSKATFTYPLYLEAMVKPSNNTMANAVWM
LSSDSTQEIDAMESYGSDRVGQEWFDQRMHVSHHVFIREPFQDYQPKDAGAWVYNSGETYRNKFRRYGVH
WKDAWNLDYYIDGVLVRSVSGPNIIDPEGYTGGTGLNKPMHIILDMEHQPWRDVKPNSTELADSNKSIFW
IDWVRVYKAN

>WP_050482451.1
MNILKLLSCSTCAILCTATHAADWDAYSIPASAGSGKTWQLQTVSDQFNYQAGTSNKPAAFTNRWNASYI
NAWLGPGDTEFSSGHSYTTGGALGLQATEKAGTNKVLSGIVSSKATFTYPLYLEAMVKPSNNTMANAVWM
LSSDSTQEIDAMESYGSDRVGQEWFDQRMHVSHHVFIREPFQDYQPKDAGAWVYNSGETYRNKFRRYGVH
WKDAWNLDYYIDGVLVRSVSGPNIIDPEGYTGGTGLNKPMHIILDMEHQPWRDVKPNSTELADSNKSIFW
IDWVRVYKAN

>KJZ27689.1
MLCTATHAADWDAYSIPASAGSGKTWQLQTVSDQFNYQAGTSNKPAAFTNRWNASYINAWLGPGDTEFSS
GHSYTTGGALGLQATEKAGTNKVLAGIVSSKATFTYPLYLEAMVKPSNNTMANAVWMLSSDSTQEIDAME
SYGSDRVGQEWFDQRMHVSHHVFIREPFQDYQPKDAGAWVYNSGETYRNKFRRYGVHWKDAWNLDYYIDG
VLVRSVSGPNIIDPEGYTGGTGLNKPMHIILDMEHQPWRDVKPNSTELADSNKSIFWIDWVRVYKAN

>WP_041248063.1
MAITSAQGADWSSFSIPAQAGAGKTWELQSVSDEFNYIAPTNNKPSAFTSRWNDSYINSWLGPGDTEFSS
EHSYTTGGVLGLQATEKTGTNKVLSGIVSSKATFTYPLYLEAMVKPTNNTMANAVWMLSADSTREIDAME
SYGSDRPGQEWFDQRMHVSHHVFIREPFQDYQPKDGGSWIYNNGETYRNKFRRYGVHWKDAWNVDYYIDG
VLVRSVSGPNIIDPKNYTNGKGLNSPMHIILDMEYQPWRDVKPSSAELADSSKSIFWIDWIRVYKAQ

>WP_026971821.1
MFNVQRVLISAVSLSVCSAVQANDWDDIPIPADPGSGYVWELQEAYSDSFNYTGKPSAFSSKWNDTYFHG
WTGPGLTYWSSDESWVSDGNLIISASRRAGTNQVNAGVVTSKTKVKYPIFLEASIKVSNLELSSNFWLLS
ENDQREIDILEVYGGAEDEWFAKNMSTNFHVFFRDGGNNIISDFNDQTHNTPSWGTYWRDGFHRFAAYWK
SPTDVTFYINGQPTPEGSWAQVVMKDKDYTGAILDKSRYNMDEEMFIILDTEDHSWRSEQGIVASDADLA
NPNKNKMYVDWIRVYKPVAEDGGGSGGDGSVDVPSGNTNLQLVHSNLCLDVANGATWNGSTYHQWVCNTG
NNNQRFTLSSLGNGEYALQSKVSQLCMELKDGSSSNGATVQQWVCNHSDSNQRWSLVDKGSNTFEIRNKA
SGKCLDIAGASMSNGGKLQQWACTGANNQRFRFVQ

>KDC53878.1
MNILKLLSCSTCAILCTATHAADWDAYSIPASAGSGKTWQLQTVSDQFNYQAGTSNKPAAFTNRWNASYI
NAWLGPGDTEFSSGHSYTTGGALGLQATEKAGTNKVLSGIVSSKATFTYPLYLEAMVKPSNNTMANAVWM
LSSDSTQEIDAMESYGSDRVGQEWFDQRMHVSHHVFIREPFQDYQPKDAGAWVYNSGETYRNKFRRYGVH
WKDAWNLDYYIDGVLVRSVSGPNIIDPEGYTGGTGLNKPMHIILDMEHQPWRDVKPNSTELADSNKSIFW
IDWVRVYKAN

>KDC50284.1
MNILKLLSCSTCAILCTATHAADWDAYSIPASAGSGKTWQLQTVSDQFNYQAGTSNKPAAFTNRWNASYI
NAWLGPGDTEFSSGHSYTTGGALGLQATEKAGTNKVLAGIVSSKATFTYPLYLEAMVKPSNNTMANAVWM

LSSDSTQEIDAMESYGSDRVGQEWFDQRMHVSHHVFIREPFQDYQPKDAGAWVYNSGETYRNKFRRYGVH
WKDAWNLDYYIDGVLVRSVSGPNIIDPEGYTGGTGLNKPMHIILDMEHQPWRDVKPNSTELADSNKSIFW
IDWVRVYKAN

>AGW43026.1
MSSMKKMVYIFVAVSTAVAAIPFISKATEKSFDVDEASPLMPSFVAFEDTTPAGMTWQKVEALSDEFNQS
WDASKWKRSNWNYSDTPVNMVDTNSGVENGYLWISATLDDSTEESWFKTSRVHSKAKISFPMYTETRLKV
AHIAAYNTFWLNNGDADNRDEIDIIEINSDPTCGENDEYPWQMNSQYFIVKNGETERNKGPWSTKKLSDA
NTRKGVTWNQDYHVFGAWWKDEHNVQFYLNGEPAGHVVSSQPFTLQQELIWDLWTQDSSWVCGLPEKEDL
LDHKRNTMKVDWVRTWKLVSK

>ADF46235.1
MKLLNKFSRAQLFGLLAGVCCSTTAVQAATFTVYNETQLNNALTSAQNNGLDTLDTIKISGTINIYGQID
IKSSVKITGAQTNRASKLTRRNTGFYNPLINVQYKNVTVENITLEGIGANTNQQLLSLAGDDTSNSALIN
LPATAYATNPKGFTATNVNFFDSAIGVASVGILPQDLNVSYNTFRNINRAVELLRDVDRVDDALLNNAQI
KVNGNIIGLYGGKLNISNNLINAENMRLAISLDGGNDGAGFIGSGFLNPWSEKRQTYTDKPVYYSSVIGE
AVINNNRIGYYQHTDGAVWTVGQTREFGIALATVANIYVVGNHVRTGLNFVTQGDGVTPVNNASFGFGSA
INVEHNGENIVIQSNHIHVGAKGNGANAFTILAFNDHGAYWNLAQTSKNLTYANNVISGSGANVFYAIGY
RNLNMLNNDVRNFSSSGAVFGCGGVVSPINNFLANVPGGYSNSYIEQNPATHGRLWFDAVRNNGQVVGAF
TGQGSRAPRSFYYLSGDDPKNPRFGDAQVVDSVSIMGCN

>WP_008302724.1
MAITSAQGADWSSFSIPAQAGAGKTWELQSVSDEFNYIAPTNNKPSAFTSRWNDSYINSWLGPGDTEFSS
EHSYTTGGALGLQATEKAGTNKVLSGIVSSKATFTYPLYLEAMVKPTNNTMANAVWMLSADSTREIDAME
SYGSDRPGQEWFDQRMHVSHHVFIREPFQDYQPKDEGSWIYNNGETYRNKFRRYGVHWKDAWNVDYYIDG
VLVRSVSGPNIIDPKNYTNGKGLNSPMHIILDMEYQPWRDVKPSSAELADSSKSIFWIDWIRVYKAQ

>AEB71854.1
MRFNKNTIALAIIASTLAAGSIAAPKTPDTSNNETTQQDMSSSVQPVVMTDDGFLSKTANTHTSYNVAGD
NLEIVFDAISESEANSKWPNMKFRPESGSYDWNTKGGLQLTLENPGDKEVRIEMKVADNIGIMGAATHQL
DLPIYLPAGKTTTVDFLFNGAEMNIEGYRGGSELDLRNIAEFQFYAVGPIGEQKVVVHDIDFIERTGDFV
LSEARQGQVIEAQIPTILTVTDFESGVEGIVERHSGSNVDIVKSDTGSAIKVHYTTDDNYPTIKFSAGKD
GQAWDWSKYGDVALAFDAKNIGDSGMQLFVRVDDALDEKLGGTATGAVNSRTGYVQIPANSEDKYYFTFK
DLAEGLDSGMRGEPPKKSFSASQVVFGWGESELDLSNIVSVQLYMMNPQEEATLVIDNLSLIPNLSTDTT
RYANLLDEFGQYQEETWPEKITDLDQLKAQAKVDKKLLKNASLMSDRSKFDGWAKGPKLEATGYFRTEKV
NDKWALVDPEGYLYFATGVDNIRMDDSYTVTGMDFADAVEADTKGMRPSQVATERYVDDKRERVEASTLR
RGMFDWLPDFNDPLADNYSYTQMVHKGPLKHGEVFSFYSANLQRKYDTDTAQEAIEIWKDVTLARMQDWG
FTSLGNWSDPMFRKNGKVPYTAHGWITGDHQRVSTGNDYWGAMHDPFDPQFRTSVATMARELGQEVDNDP
WCIGYFVDNELSWGNTVNDTNHYALAVSGLRESADVSSTKAAFDELLKTKYGSIEKFNKAWGVEVTSWDV
FAEGFNYQGEYTDTVKADLSVLLDAFADKFFAVVSEEMEKVLPNHLYMGVRFSDWGITPEAATAAARYVD
VMSYNLYATDLNAKGDWSRLPELDKPSIIGEFHFGSTDSGLFHPGIISSDDQKGRAESYAKYMESVIDNP
YFVGAHWFQYMDSPVTGRAWDGENYNVGFVTVTDTPYEPLVESAKEINRNLYNRRFGSLN

>ADM25828.1
MYCSFYWLSNNYVKERRISVRFNKNTIALAIIANTLAAGSIAATKTPDTSNNETTQQDMSSSVQPVVMTD
DGFLSKTANTHTSYNVAGENLEIVFDAISESEANSKWPNMKFRPESGSYDWNTKGGLQLTLENPGDREVR
IEMKVADNIGIMGATTHQLDLPIYLPAGKTTTVDFLFNGAEMNIEGYRGGSELDLRNIAEFQFYAVGPIG
EQKVVVHDIDFIEHTGDFVLSEARQGQVIEAQIPAILTVTDFESGVEGIVERHSGSNVDIVKSDTGSAIK
VHYTTDDDYPTIKFSAGKDGQAWDWSKYGDVALAFDAKNIGDSGMQLFVRVDDALDEKLGGTATGAVNSR
TGYVQIPANSEDKYYFTFKDLAEGLDSGMRGEPPKKSFSASQVVFGWGESELDLSNIVSVQLYMMNPQEE
ATLVIDNLSLIPNLSTDTTRYANLLDEFGQYQEETWPEKITDLDQLKAQAKVDKKLLKNASLMSDRSKFG
GWAKGPKLEATGYFRTEKVNDKWALVDPEGYLYFATGVDNIRMDDSYTVTGMDFADAVEADTKGMRPSQV
AAERYIDDKRERVEASTHRRGMFDWLPDFNDPLADNYSYTQMVHKGPLKHGEVFSFYSANLQRKYDTDSA
QGAIEIWKDVTLARMQNWGFTTFGNWSDPMYRKNGKVPYTAHGWITGDHQRVSTGNDYWGAMHDPFDTNF
RVSVAIMAKELGEEVDNDPWCIGYFVDNELSWGNTVNNTNHYALAISGLRASTKESSAKAAFDALLKAKY
GSIKKFNAAWGVDVASWDAFAKGFNYQGEYTETVKADLSVLLDAFADKYFAVVSEEMEKVLPNHLYMGVR
FSDWGITPEAATAAARYVDVMSYNLYATDLNAKGDWSRLPELDKPSIIGEFHFGSTDSGLFHPGIISSDN

QQGRAQSYAKYMESVVDNPYFVGAHWFQYMDSPVTGRAWDGENYNVGFVSVTDTPYEPLVKSAKEINRNL
YNRRFGSINK

>BAA04744.1
MVEVMKFTKNKIAALLSLTLLGVYGCGSTPSSSDAEGAVEDVGGTIPDFESAAFFKKVKKDHRKAEVVSD
QGVTSGSSALKVNFDSVSEANKFKYWPNVKVHPDSGFWNWNAKGSLSLDITNPTDSPANIILKLADNVGV
MGSGDNQLNYAVNVPAGETVPVEMLFNGTKRKLDGYWGGEKINLRNIVEFQIFVQGPMDAQTVIIDNFNL
VDATGDFIEASGQEVKVSGPIPTVASITSFDEGQPTFVAFDRSAAATVTELKTDMGGLLAVKLAATNAYP
NITFKAPQPWDWSEYGDFSLAFDLESKADEPLQLFVRVDDAENENWGGTANGVVDSMSSYVTLAPGDDGT
FYLPLGQTGSQIVSGMRAEPPKKSYNAQAISYGWGEKSLDTSNIVSFQLYLQNPTKDAEFNIKSVRLIPN
IDADATRYEGLIDQYGQFTGSEWPKKITEDEELETMGKLAKMSLKSTSQMPGRSIYGGWADGPKLKGTGF
FRTEKVDGKWSLVDPQGNLFFATGVDNIRMDDTVTITGHDFADKDKRSGKEVASEVRRSMFTWLPEDDDV
LAENYDYANWVHSGALKKGEVFSFYGANLQRKYGGTFSEAEKVWKDITIDRMVDWGFTTLGNWADPMFYD
NKKVAYVANGWIFGDHARISTGNDYWGPIHDPFDPEFVNSVKAMTKKLMTEVDKNDPWMMGVFVDNEISW
GNTKNDANHYGLVVNALSYDMKKSPAKAAFTEHLKEKYWAIEDLNTSWGVKVASWAEFEKSFDHRSRLSK
NMKKDYAEMLEMLSAKYFSTVRAELKKVLPNHLYLGAPFADWGVTPEIAKGAAPYVDVMSYNLYAEDLNS
KGDWSKLAELDKPSIIGEFHFGSTDSGLFHGGIVSAASQQDRAKKYTNYMNSIADNPYFVGAHWFQYIDS
PTTGRAWDGENYNVGFVSITDTPYVPLVEAAKKFNQDVYMLRYKK

>BAD88713.1
MRHTLSTLTALLLCSSSFAADWDGLAVPADAGDGKTWQLQSSLSDDFSYSAPAEGKSQAFYERWSEGFIN
AWQGPGLTDYHNPNSRVENGELVITATRKAGTNEVYTGAIHTNESVQYPVYIETSSKIMDQVLANAVWML
SSDSTQEIDIVEAYGSSRADQTWFAERMHLAHHVFIRDPFQDYQPKDAGAWYADGRLWREQYSRVGVYWR
DPWHLEYYIDGQLVRTVSGVDMIDPYNYTNGLGLSKPMQIIVDAEDQDWRSDNGILATDAELSDTSKNQF
YVDWIRVYKPVADNGDDDGGNGNGGDNSAATIETNFADFISTGKEGTSVAGDMVTGFNPSGSGNINYNTI
GDWAEYSINLPEAGQYRVELDTASTVASGLGADISIDDVYVGRIELQQTGGWESYQTFSLNDALTIGAGS
HTVRIQSAGSAEWQWNGNSIRFVKVSESTDTSGDSNATAQPVTIEAESFVRTDGPYDGFQTYTQNGVGAI
NYNQRGDYAEYNFSVSAAGNYLFSAYTATPESGAALQVSIDGNAVATIDVASTGGWSNFAKTSASAGVAL
GAGSHTMTVKSAGNTTNTWEWNADRFELIPQ

>BAQ95400.1
MRNFTLALVCGLFAATTQAADWDNYPVPADAGTGNQWELQPLSDDFNYTAPAVGKSATFYQRWSEGFINA
WTGPGRTIFAANHSSVEDGTLKLRASRRAGTNQVYTGAIHSKTSMTYPIFMETRSKITNLTMANAFWLLS
ADSTQEIDVQESYGSDRPDQTWFDERLHLSHHVFIRDPFQDYQPKDEGSWYKDPNLSTWRNAYHTIGVYW
IDPWNLKYYVDGKLVRTVSGVSMIDPNGYTGGTGLSKPMQAIFDVEDQDWRSDAGIFATDAELADNSKNT
YHIDWVRFYKPVPITGGGTPPNTGDTTVVELGSFTSTGKQGAAVAGDTVVGFSKNSTNINYNTVGDWADY
SVNLPVAGNYKIELVTASPQTGIGADITFDGSFVGTIALANTGGWEIYKTYALSDTVYVASAGNHTVRVQ
SSGSASWQWNGDEIRFIKVEDGSVPPPATPATITVEAESFDLVGGTFADGQAQKISTYSTGGKTAINYVN
KGDFADYTINVATAGSYQLSYQIGSGVVGGQIDFLVKENGSWINKTQTPVPNNGWNNFQALNGGIVTLQA
GNQQVRVFGGGSNDWQWNLDKFSLTPQ

>AMX03457.1
MKFFASALTALAISSPTIAADWDGLSVPADAGAGNIWQLQDNVSDDFNYSAPANGKSQAFYDRWAEGFIN
AWQGPGLTDYHNPNSRVENGQLVIQATRKPNTDQVYTGAIHSNDSVQYPVYIETRVKIMDQVLANAVWML
SSDSTEEIDIVEAYGSSRPDQTWFAERMHLAHHVFIRDPFQDYQPQDMGAWYSDGRLWREQYSRVGVYWR
DPWHLEYYIDGQLVRTVSGTDMIDPYGYTNGTGLSKPMQIIIDAEDQDWRSDNGILATDAELADSSKNQF
YVDWVRVYKPVPADDNNDDSDVTVSFDFDSFYATGKEGDAVAGDTVTGFNPSGNGNINFNTLGDWAEYSF
NIPEAGDYRLELDTASTVTSGIGADLQIDDIYIGQIRMQATGGWENYQTFSLSNNLTIGAGAHILRVQSS
GSAPWQWNGDKIRLIKVGDSTGNNGGNDGGNDNGQQTQVITLEAENFTSTGGTYDGFQTYTQNGVTAINY
NQRGDYGEYTLSVPSGGNYIFSAVVATPETGAAMALSLNGNALLNLDVPSTGSWSHFSTISAANAVTLPA
GTHTLRITSAGNTSNTWEWNADSFEFSPQ

>WP_062064993.1
MKKITSCIAAALLLSAPLASFAADWDSVPIPAAAGTGKTWQLQSISDEFNYTASPTVKPAEFNSRWVPSF
INSWTGPGDSEFNAGHSYTSGGSLALQSSAKAGTNKIYTGIISSKETFTYPLYLEARVKHTGNTLANAVW
MLSADSTQEIDAMESYGSDRVGQEWFDQRMHVSHHVFVRSPFQDYQPTDAGSWILNSAGTWRGAMHTYGV
HWKDPWNLDYYIDGVKVRTVSGPAMIDPNNFTGGTGINKSLHIIIDVEHQDWRDVRPTAAELADTSKSIM

FVDWIRVYKPVTSSATSSSNSVSSSSSSLSNSSSSSATTVIDFANYFDTGKSTASVAGDTYVGFNKSGSG
NINYNTAGDWADYLVTLPSDGQYKIEVTTASPLISGIGAKLSIDGIYVSTTSLAATGGWELYSASTLANN
LSIGAGTHTVRIESAGTSTWQWNGDEIRVTKVGSAAVGSPTTPAPVAMIIQAESFSATGGVYDGFKTYSV
NGVSAINYNQRGDWADYAINVAADGSYTFNAYVSSPMSGAALEVSVDGVKVLTQAVPNNGSWDSFQKVSS
VSKIALTKGAHTIRVTSAGATSSTWEWNADKFELVP

>WP_049721028.1
MKHSISTLAALLLSSSSLAADWDDLPVPANAGSGNTWQLQSNVSDDFNYSAPANGKSSAFYDRWSEGFIN
AWQGPGLTDYHNPNSRVENGELVIQATRKPGTNQVYTGAVHTNDSIQYPVYIETSSKIMDQVLANAVWML
SSDSTEEIDIVEAYGSSRPDQTWFAERMHLAHHVFIRDPFQDYQPKDVGAWYADGRLWRDQYSRVGVYWR
DPWHLEYYIDGELVRTVTGVDMIDPYGYTNGNGLSKPMQIIVDAEDQDWRSDNGIVATDTELADNSKNQF
YVDWIRVYKPVPNSNGGGGNDITSSVDFDNFFATGKDGSAVAGDSFNGFNPSGNGNINYNTVGDWAEYSI
NLPEDGEYRLELDTASTVSSGLGADISIDGVFVGTVAISQTGGWESYQTFSLANTINIGAGTHTLRVQSA
GSSQWQWNGEAIRMVKMGEGSSNNQTTAPTPTEAISLEAESFNNTGGPYGGFQTYTQSGITATNYNQRGD
YAEYSLSVPVAGNYNVSAFVATPESGAAMTLTLNGSALVSLDVPSTGGWNTFAEVSASGGVVLPAGTHTL
RVTSSGNTSNTWEWNADRFAFTPQ

>WP_049629425.1
MKKITSCIAAALLLSAPLASFGADWDSVPIPAAAGTGKTWQLQPISDEFNYTASPTVKPAEFNSRWVPSF
INSWTGPGDSEFNAGHSYTSGGSLALQSSAKAGTNKIYTGIISSKETFTYPLYLEARVKHTGNTLANAVW
MLSADSTQEIDAMESYGSDRAGQEWFDQRMHVSHHVFIRSPFQDYQPKDEGSWVVNPAGGTWRGAMHVYG
VHWKDPWNLDYYIDGVKVRTVSGPAMIDPYNFTNGTGINKPLHIIIDVEHQDWRDVRPTAAELADTSKSI
MFVDWIRVYKPVTSSATSSSNSVSSSSSSLSNSSSSSATTVIDFANYFDTGKSTASVAGDTYVGFNKSGS
GNINYNTAGDWADYLVTLPSDGQYKIEVTTASPMTSGIGAKLSVDGIYVSTTSLAATGGWELYSASTLAN
NLSIGAGTHTVRIESTGTSAWQWNGDEIRVTKVGSAPVGSPTTPAPVAMIIQAESFSATGGVYDGFKTYS
VNGVSAINYNQRGDWADYAINVAADGSYTFNAYVSSPMSGAALEVSVDGVKVLTQAVPNNGSWDSFQKVS
SASKIALTKGAHTIRVTSAGATSSTWEWNADKFELVP

>WP_041522726.1
MKHTISTLTALLLCSSSFAADWDGLPVPADAGSGNTWQLQSNVSDDFNYSAPANGKSAAFYDRWSEGFIN
AWQGPGLTDYHNPNSRVENGELVIQATRKPGTNQVYTGAVHTNDSIQYPVYIETSSKIMDQVLANAVWML
SSDSTEEIDIVEAYGSSRPDQTWFAERMHLAHHVFIRDPFQDYQPKDAGAWYADGRLWRDQYSRVGVYWR
DPWHLEYYIDGQLVRTVSGVDMIDPYGYTNGNGLSKPMQIIVDAEDQDWRSDNGIIATDADLADSSKNQF
YVDWIRVYKPVPDANGGGDNGGDNGGDNGGDNGGDNDITSSVDFDSFFATGKDGSAVAGDSVNGFNPSGN
GNINYNTVGDWAEYSINLPEAGEYRLELDTASTVSTGLGADISIDDVFVGTVAISQTGGWESYQTFSLAN
TINIGAGTHTLRVQSAGSSPWQWNGNAIRMVKVGEGSSNNQTTTPTPSEMISLEAESFNSTGGPYDGFQT
YTQSGITATNYNQRGDYAEYTLSVPTAGNYNVSAIVATPESGAAMTLTLNGNALVSLDVPSTGGWNTFTE
VNASGAVALPAGTHTLRVTSSGNTANTWEWNADRFIFTPQ

>WP_040391904.1
MSSVAATGFAADWDGVPIPAPAGAGKTWQLQSISDEFNYAASPTFKPTEFNSRWVPSFINSWTGPGDSEF
NAGHSYTSGGSLALQSSAKAGTNKIYTGIISSKETFTYPLYLEARVKHTGNTLANAVWMLSADSTQEIDA
MESYGSDRAGQEWFDQRMHVSHHVFIRSPFQDYQPKDEGSWVVNPAGGTWRGAMHVYGVHWKDPWNLDYY
IDGVKVRTVSGPAMIDPYNFTNGTGINKPLHIIIDVEHQDWRDVRPTAAELADTSKSIMFVDWIRVYKPV
TSSATSSSNSVSSSSSSLSNSSSSSATTVIDFANYFDTGKSTASVAGDTYVGFNKSGSGNINYNTAGDWA
DYLVTLPSDGQYNIEVTTASPMTSGIGAKLSIDGIYVSTTSLAATGGWELYSASTLANNLSIGAGTHTVR
IESTGTSAWQWNGDEIRVTKVGSAAVGSPTTPAPVAMIIQAESFSATGGVYDGFKTYSVNGVSAINYNQR
GDWADYAINVAADGSYTFNAYVSSPMSGAALEVSVDGVKVLTQAVPNNGSWDSFQKVSSVSKIALTKGAH
TIRVTSAGATSSTWEWNADKFELVP

>AFV00509.1
MNKLPLALAAIALASQTHAADWDGIPVPADPGAGNKWELHPLSDDFNYSAPAVGKSAAFFERWNEGFINP
WLGPGLTEFTASQSQVANGTLQLKASRKAGTNKVLTGAIHSKESLIYPLYMEARTKITNLTAANAFWLLS
SDSTQEIDVQESYGSDRPDQVWFDERLHLSHHVFIRDPFQDYQPKDEGSWYKKQGQSTWRDAYHTIGVYW
IDPWNLEYYVDGVHVRTVSGSSIIDPYGYTGGTGLSKPMQAIFDVEDQDWRSDNGITATDAELADPSKNT
YFVDWVRFYKPVPDSGGTTPPPANGETVVKEMADFTATGKTGAAVAGDTITGFNKNGTNINYNTLGDYGD
YTVNLPSAGSYKIELVAASPSSGNLGADISIDGSYVGSINVAATGGWEVYQTSTLATNVYVASAGNHSVR

VQSSGTASWQWNGDEIRFVKIDDGNTQPPINPTPTTVTVEAENFDAVGGTYADGQAQKISTYTTGGVTAI
NYVNKGDYADYTLNVSAAGNYALTVYAGSGVVGGQVDFLVNTNGSWVNQSQTTVPNNGWNNFQALNGGVV
SLPAGSVKVRLFGGGSHDWQWNMDKFVLTPQ

>WP_010133209.1
MKTTSLTLAALALSSSALASDWDNIPVPADAGAGNTWKLHSLSDDFNYAAPPVGKSATFFERWSEGFINP
WLGPGETEYYAPNSYVEGGNLVIKASRKPGTIKVHTGAIHSKESMTYPLFMEARVKITNLTLANAFWLLS
SDSTEEIDVLESYGSDRPSETWFDERLHLSHHVFIREPFQDYQPKDAGSWYPNPDGGHWRDQFFRIGVYW
IDPWTLEYYVNGEHVRTVSGVEMIDPYGYTNGNGLSKPMQVIFDAEHQPWRDAQGTLPPTDEELADPSRN
KFLVDWVRFYKPVADSNGGGDPGNGGDPGNGGNPGSGETIRVEMGSFSATGKAGAAVAGDTVAGFNANGD
NINYNTLGDWGDYTVNFPEAGSYNVELLAASPTTSGIAADVQVDGSYVGTIPLSSTGDWELYNTFTLPST
IYIASAGNHTIRVQSAGGSAWQWNGDEIRFTKTEDDSTPPPPPATGATITVEAESFASVGGTYADGQAQP
ISVYTTNGSTAINYVNAGDFADYTINVANAGTYAITYHVGSGVTGGSIEFLVNEGGSWNSKTATPVPNQG
WDNFQPLNGGSVYLEAGTYQVRLHGVGSNDWQWNLDKFVLSN

>WP_011467657.1
MKTTKCALAALFFSTPLMAADWDGIPVPADPGNGNTWELQSLSDDFNYAAPANGKSTTFYSRWSEGFINA
WLGPGQTEFYGPNASVEGGHLIIKATRKPGTTQIYTGAIHSNESFTYPLYLEARTKITNLTLANAFWLLS
SDSTEEIDVLESYGSDRATETWFDERLHLSHHVFIRQPFQDYQPKDAGSWYPNPDGGTWRDQFFRIGVYW
IDPWTLEYYVNGELVRTVSGPEMIDPYGYTNGTGLSKPMQVIFDAEHQPWRDEQGTAPPTDAELADSSRN
QFLIDWVRFYKPVASNNGGGDPGNGGTPGNGGSGDTVVVEMANFSATGKEGSAVAGDTFTGFNPSGANNI
NYNTLGDWADYTVNFPAAGNYTVNLIAASPVTSGLGADILVDSSYAGTIPVSSTGAWEIYNTFSLPSSIY
IASAGNHTIRVQSSGGSAWQWNGDELRFTQTDADTGTNPPSTASIAVEAENFNAVGGTFSDGQAQPVSVY
TVNGNTAINYVNQGDYADYTIAVAQAGNYTISYQAGSGVTGGSIEFLVNENGSWASKTVTAVPNQGWDNF
QPLNGGSVYLSAGTHQVRLHGAGSNNWQWNLDKFTLSN

>WP_015048661.1
MNKLPLALAAIALASQTHAADWDGIPVPADPGAGNKWELHPLSDDFNYSAPAVGKSAAFFERWNEGFINP
WLGPGLTEFTASQSQVANGTLQLKASRKAGTNKVLTGAIHSKESLIYPLYMEARTKITNLTAANAFWLLS
SDSTQEIDVQESYGSDRPDQVWFDERLHLSHHVFIRDPFQDYQPKDEGSWYKKQGQSTWRDAYHTIGVYW
IDPWNLEYYVDGVHVRTVSGSSIIDPYGYTGGTGLSKPMQAIFDVEDQDWRSDNGITATDAELADPSKNT
YFVDWVRFYKPVPDSGGTTPPPANGETVVKEMADFTATGKTGAAVAGDTITGFNKNGTNINYNTLGDYGD
YTVNLPSAGSYKIELVAASPSSGNLGADISIDGSYVGSINVAATGGWEVYQTSTLATNVYVASAGNHSVR
VQSSGTASWQWNGDEIRFVKIDDGNTQPPINPTPTTVTVEAENFDAVGGTYADGQAQKISTYTTGGVTAI
NYVNKGDYADYTLNVSAAGNYALTVYAGSGVVGGQVDFLVNTNGSWVNQSQTTVPNNGWNNFQALNGGVV
SLPAGSVKVRLFGGGSHDWQWNMDKFVLTPQ

>BAE06228.1
MKTTSLTLAALALSSSALAADWDNIPVPADAGAGNTWELHSLSDDFNYAAPPVGKSATFFERWSEGFINP
WLGPGETEYYAPNSYVEGGNLVIKASRKPGTIKVHTGAIHSKESMTYPLFMEARVKITNLTLANAFWLLS
SDSTEEIDVLESYGSDRPSETWFDERLHLSHHVFIREPFQDYQPKDAGSWYPNPDGGHWRDQFFRIGVYW
IDPWTLEYYVNGEHVRTVSGVEMIDPYGYTNGNGLSKPMQVIFDAEHQPWRDAQGTAPPTDEELADPSRN
KFLVDWVRFYKPVADNNGGGDPDNGGDPGNGGNPGSGETIRVEMGSFSATGKAGAAVAGDTVAGFNSNGD
NINYNTLGDWGDYTVNFPEAGNYNVELLAASPTTSGIAADVQVDGSYVGTIPLSSTGDWELYNTFTLPST
IYIASAGNHTIRVQSAGGSAWQWNGDEIRFTKTEDDNTPPPPPATGATINVEAESFASVGGTYADGQAQP
ISVYTTNGSTAINYVNAGDFADYTINVADAGTYAITYHVGSGVTGGSIEFLVNEGGSWNSKTATPVPNQG
WDNFQPLDGGSVYLEAGTHQVRLHGVGSNDWQWNLDKFVLSN

>BAD29947.1
MRKITSILLTCVMGCTATYAADWDGVPVPANPGSGKTWELHPLSDDFNYEAPAAGKSTRFYERWKEGFIN
PWTGPGLTEWHPHYSYVSGGKLAITSGRKPGTNQVYLGSITSKAPLTYPVYMEARAKLSNMVLASDFWFL
SADSTEEIDVIEAYGSDRPGQEWYAERLHLSHHVFIRDPFQDYQPTDAGSWYADGKGTKWRDAFHRVGVY
WRDPWHLEYYVDGKLVRTVSGQDIIDPNGFTGGTGLSKPMYAIINMEDQNWRSDNGITPTDAELADPNRN
TYYVDWVRFYKPVPINGNATTVELGNFHNTGKDGANVTGDTVLGFNKNGNNINYNTKGDWADYTVNLPAA
GEYRVDLVIASPMSSGLGAELTFAGNAAKTVTLSNTGGWESYQTFTLPQTISVSSPGNYNFRLKSTGSSN
WQWNGDEIRFVKL

>BAC99022.1
MKTTQCALAALVFSTPLMAADWDGTPVPADAGPGNTWELHPLSDDFNYSAPASGKSATFFERWSEGFINP
WLGPGETEYYGPNSSVESGNLVIKASRKAGTTKIHAGAIHSNESVTYPLYMEARVQVTNLTMANAFWLLS
SDSTQEIDVLESYGSDRPSETWFDERLHLSHHVFIREPFQDYQPKDDGSWYPNPNGGTWRDQWIRIGTYW
VDPWTLEYYVNGEHVRTVTGPSMIDPYGYTGGTGLSKPMQVIFDAEHQPWRDTQGTAPPTDEELADPSRN
KFLVDWVRFYKPVPDTNGGGPGNGSISVEKEAEDFDNVGGYFSDGQSQAISTYTTGATTAINYVNREDYA
DYTVTVPEDRIYNITYNISSGITGGRIDFLVNESGTWSNKTQTAVPNAGWNNFQPLSGGTVYLEAGTHTV
RLYGAGTHDWQWNLDKFTLSN

>WP_062064997.1
MKKHISCCIALMLTSVASLAADWDVYPVPASAGAGKVWQLQPQSDEFNYNFSATTSAATFGGKWTNFYHN
TWEGPGPTRWMRENTSVSDGQLQIKATRVAGETKTYDVDLNLDGINEQFTSPATRAGCITSTTRVKYPVF
VEARVKIANAVMASDVWMLSPDDTEEIDILEAYGGKAARNDWFAQRLHLSHHLFIRNPFTDYQPRDASTW
YAGDGVTYWADNWVRIGVNWVSPTRLEYYVNGQLVKVMDKLNTVNGIDGIDPWNITGGKGITKEMDIIIN
MEDQNWNAAQGRQPTDAEITNTSNHTFKVDWIRVYKPVTATTSSSSSSSAASSASSNTVQLIDFANYYDT
GKSTASVSGDNYIGFNKSGGGNINYNTVGDWGDYLVTLPSDGKYKFEIITASPMTSGLGAKLIIDGIYVG
TISVGSTGGWEVYSAFALANSISIGAGTHTVRIESTGSSTWQWNGDQIRITRVGSL

>ALN70307.2
MRKITSILLTCVMGCTATYAADWDGVPVPANPGSGKTWELHPLSDDFNYEAPAAGKSTRFYERWKEGFIN
PWTGPGLTEWHPHYSYVSGGKLAITSGRKPGTNQVYLGSITSKAPLTYPVYMEARAKLSNMVLASDFWFL
SADSTEEIDVIEAYGSDRPGQEWYAERLHLSHHVFIRDPFQDYQPTDAGSWYADGKGTKWRDAFHRVGVY
WRDPWHLEYYVDGKLVRTVSGQDIIDPNGFTGGTGLSKPMYAIINMEDQNWRSDNGITPTDAELADPNRN
TYYVDWVRFYKPVPINGNATTVELGNFHNTGKDGANVTGDTVLGFNKNGNNINYNTKGDWADYTVNLPAA
GEYRVDLVIASPMSSGLGAELTFAGNAAKTVILSNTGGWESYQTFTLPQTISVSSPGNYNFRLKSTGSSN
WQWNGDEIRFVKL

>WP_049721027.1
MASSFSSSSSRQSSSSSDATGVDWADLPVPADAGAGNTWELVDALSDDFNYSAPGDGKSQVFYERWSEGF
INAWQGPGLTDYHDPNSSVVDGNLVIEATRKPNSDEVYTGAIHSKASVRYPVYVETRVKIMDQVLANAVW
MLSSDSTEEIDIVEAYGSSRSDQAWFAERMHLSHHVFIRDPFQDYQPKDDASWYVDGRLWRDQFSRVGIY
WRDPWHLEYYIDGELVRTVSGEEIIDPKGYTNGAGLSKDMQIIVDAEDQGWRSDNGIKASDEELSDPDKN
RFYVDWIRVYKPASTTPGDSDNTASETGISVTTDFGAFFATGKEGDTVVEDTVDGFNAADGNITHNTLGD
WGEYTINIPEDGDYRLEIDVASPTESGLAANIMIDDTDVGQIAISTTGSWEVYDTFSLDTPVTLTAGTHT
LRVQSAGIATWQWNGDVIRFLQLSE

>WP_049720972.1
MTLACAVSAVVAGTVASHALAQTVHIQAENFTAVGGSYADGQPQAVSTYAVNGVQAINYVNRGDYVEYEV
NIAQAGVYTVQYLIGTAVPSGAAVELSVKNGNTWQSQDTTTVPTGGWDNFQPLDGTHEITLPAGNVSIRL
TGSGANDWQWNLDEFALTLTQSEVEPEIPSDISQEAEDYHAVSGTFADGQTAPVSTYSVNGGTALNYINR
GDAIEYQIAVDRAGIYDLTYFIGTAITSGAQVEFQVKQNGNWVSQGVESVTANGWDNFQAQSAGHTVQLP
AGVVEVRLLASGSSDWQWNIDKFDLVFVSDNGASSSSSVSSSSSSVSSTSSSVSSSSSVSSTSSSSSSSVS
SGSSSSQVPGGVVTVSGDFSLEAESFNAVGGSVEIYSVNGGRAVNYFNAGDYLEFDIDNAQGGLYEAVYR
VGTGNTSGTAVGLMLTDHQGNLVLKNTTDVVSQGGWDTFYDLTSSTRFNIYPGTSKIRITGAGSQDFQFN
IDNIVFKYLGTVDNNLDGDGDGVADINDSCSGTPSGEAANGEGCSPSQLDTDVDGINDAEDQCPSSAIDA
FVNSVGCATSGGDDDDFDGVFNWDDQCPNTFRMNVGPDGCLASGRDSDGDGVVNSVDVCPATPGSEFANG
QGCSSSQVSTVESVDVHVNANIEHIVNGVSDFGRDRHMTVHSTVYEQDWDGHADKLNYLLNELDVYMGRD
NGSATWKFQDTQEDPNRPNMADLAWMEGRGAELRQVHEDNPFYSRFPDSKTQMIAGTNPHPLYPTLSWYD
NGKTWSGWQPMNIETSAEWVGKYLESYFANSRNGDIGEPTPTFWEVINEPDMKMKTGQFMVTNQEQIWEY
HNLVAQEIRSRLGNEAPLIGGMTWGQHDFYRRDGISRFADDSYDQWIVADDPAVEAESEAFFESRMATTV
DDTRALDWYQWDVMWKGFMDAAGDSMDFYAVHIYDWPSANEGGKGSLRRGGHVQGMLDMMEWYDVHEN
GM
NNRKPIVLSEYGAVQGGWDNLPHNPRYESEVLKSFNGMLMQFLERPDYVIKSMPFTPAKPLWGYVGAQDG
YTTVGVCGYKEVPDCNVRYHYAMMIEENLNQGDWVWSDYIRFYELWDDVDGTRVDTRSSDADVQIESYVD
GDEVFVILNNLEDESTTVNLNLHGLSLGNVSKVEMRDMHFDASHKTQLDRRFMDSAPSTVTLAPDGTVVL
RYTMSNNVAVNEQVQEKKYFGSSVSGGSEPHRVSIAGGDIPVSINNVQVPSGPAEAMLRLTVAMFMSEDD
VPNGNLTINTLTINGVEVATPIDWRGPADYQADRYFATLEIPVPVNTLQANNDIKVDFRHNGELTVVNLV

VWEFSTEPQR

>WP_049629424.1
MKKHISCCIALMLTSVASLAADWDAYPVPASAGAGKVWQLQPQSDEFNYNFSATPSAATFGGKWTNFYHN
SWEGPGPTRWMRENTSVSGGQLQIKATRVAGETKTYDVDLNLDGVNEQFTSPATRAGCITSTTRVKYPVF
VEARVKIANAVMASDVWLLSPDDTEEIDILEAYGGKAARNDWFAQRLHLSHHLFIRNPFTDYQPRDASTW
YAGAGVTYWADNWVRIGVNWVSPTRLEYYVNGQLVKVMDKLNTVNGIDGIDPWNITGGKGITKEMDIIIN
MEDQNWNAAQGRQPTDAEITNASNHTFKVDWIRVYKPVTATTSSSSSNSSAASSASSNTVQLIDFANYFD
TGKTTASVSGDNYIGFNKSGGGNINYNTVGDWGDYLVTLPSDGKYKFEIITASPMTSGLGAKLIIDGIYV
GTISVGSTGGWEVYSAFALANSISIGAGTHTVRIESTGSSSWQWNGDQIRITRVGSL

>AEE61375.1
LEVIPVRVKRFSRLTLACAVSAVVAGTVASHALAQTVHIQAENFTAVGGSYADGQPQAVSTYAVNGVQAI
NYVNRGDYVEYEVNIAQAGVYTVQYLIGTAVPSGAAVELSVKNGNTWQSQDTTTVPTGGWDNFQPLDGTH
EITLPAGNVSIRLTGSGANDWQWNLDEFALTLTQSEVEPEIPSDISQEAEDYHAVSGTFADGQTAPVSTY
SVNGGTALNYINRGDAIEYQIAVDRAGIYDLTYFIGTAITSGAQVEFQVKQNGNWVSQGVESVTANGWDN
FQAQSAGHTVQLPAGVVEVRLLASGSSDWQWNIDKFDLVFVSDNGASSSSSVSSSSSSVSSTSSSVSSSS
SVSSTSSSSSSVSSGSSSSQVPGGVVTVSGDFSLEAESFNAVGGSVEIYSVNGGRAVNYFNAGDYLEFDI
DNAQGGLYEAVYRVGTGNTSGTAVGLMLTDHQGNLVLKNTTDVVSQGGWDTFYDLTSSTRFNIYPGTSKI
RITGAGSQDFQFNIDNIVFKYLGTVDNNLDGDGDGVADINDSCSGTPSGEAANGEGCSPSQLDTDVDGIN
DAEDQCPSSAIDAFVNSVGCATSGGDDDDFDGVFNWDDQCPNTFRMNVGPDGCLASGRDSDGDGVVNSVD
VCPATPGSEFANGQGCSSSQVSTVESVDVHVNANIEHIVNGVSDFGRDRHMTVHSTVYEQDWDGHADKLN
YLLNELDVYMGRDNGSATWKFQDTQEDPNRPNMADLAWMEGRGAELRQVHEDNPFYSRFPDSKTQMIAGT
NPHPLYPTLSWYDNGKTWSGWQPMNIETSAEWVGKYLESYFANSRNGDIGEPTPTFWEVINEPDMKMKTG
QFMVTNQEQIWEYHNLVAQEIRSRLGNEAPLIGGMTWGQHDFYRRDGISRFADDSYDQWIVADDPAVEAE
SEAFFESRMATTVDDTRALDWYQWDVMWKGFMDAAGDSMDFYAVHIYDWPSANEGGKGSLRRGGHVQGML
DMMEWYDVHENGMNNRKPIVLSEYGAVQGGWDNLPHNPRYESEVLKSFNGMLMQFLERPDYVIKSMPFTP
AKPLWGYVGAQDGYTTVGVCGYKEVPDCNVRYHYAMMIEENLNQGDWVWSDYIRFYELWDDVDGTRVDTR
SSDADVQIESYVDGDEVFVILNNLEDESTTVNLNLHGLSLGNVSKVEMRDMHFDASHKTQLDRRFMDSAP
STVTLAPDGTVVLRYTMSNNVAVNEQVQEKKYFGSSVSGGSEPHRVSIAGGDIPVSINNVQVPSGPAKAM
LRLTVAMFMSEDNVPNGNLTINTLTINGVKVATPIDWRGPADYQADRYFATLEIPVPVNTLQANNDIKVD
FRHNGELTVVNLVVWEFSTEPQR

>WP_043317023.1
MKLPYPRRPLALAQSVLAAGIAFSTAATAADYRIEAEAFSSVGGTYADGQPQKISTYSVNGVTAINYVNR
SDYAEYSLQIAEAGTYNLQYLIGTGVASGAEIDFQIGSGSSWNSLVKKAVPTGNWDNFQPLDAGNVTLPA
GTVNLRVVGSGSNDWQWNLDALELTQVNTGGSSSSSSSSGGSSSSGGGSSAFTVEAESFTQVGGTYADGQ
PQKIGTYTTNGATAINYVNKGDYAEYTISVPQAGNYDLTYFAGTAINGGRIDFQHNSSGSWQTLAQTSVP
NAGWDNFQALAGGSVYLPAGTQQIRVYGGGTHDWQWNLDRFELSYDGAGSGSGGGSSSSSSSSSSSSSSS
SSSSSGGSSSSSSSSSSSSSSSSSGGSSSGGSSPGNGSPVSGTFTLQAESAHIVGGEIETYAINGGTAVN
YFNSGDYLEYNLSLDQSGLYRPKFFVGTGNTSGTSVGLMATDHEGELVIKNTTDVQSQGDWDSFYLLNAS
SEINLFAGDLTIRIYGAGSQDFQFNIDYAIFERVGDADLALDGDGDGTPDVSDQCPSTDPAETANSVGCA
PSQLDTDEDGITDNLDQCPTTGADEFVNAVGCASAGGDDDDFDGVLNSADNCANTPYGQNVDPSGCTGFA
DSDGDGVANSADNCPSTPAGEFANESGCSATQVGNSHSATVTVNANIKHSVNGVSDFGRNRHITAHTTIY
ENDWKGHADKLNYFLNTLDVTLGRDNGTATWKFQDTKEDPNKANWPDMDYMVTRGQELRESYEANAFYKR
FSPESTELIAGTNPHPTYPTLSWYDNGKTWHGWQPMDIETSAAWMGQYLKHYYANSSNGNVGDPMPKFWE
VINEPDMEMKTGKFMVTNQEALWEYHNLVAQEIRSKLGNEAPMIGGMTWGQHDFYRRDGISRFGDDNYDQ
WITNEDPEVQEAARAFFRNAMTTTVDDTRSQDWYQWDVMWKGFMDAAGHNMDFYAVHVYDWPGVSNDST
S
TLRRGGHLPAMLDMMEWYDVHQNGAANRTPIVLSEYGAVQGGWDYLAHNSRYESEVVKSFNAMLMQILDR
PDYVIKSMPFTPAKPLWGYKPFGCGYEEVRTCTAPYHYSMMKESQLNNDDWHWSDYIQFFELWADVDGTR
VDAVSSDADVQVQSYVDGNEVFVIINNLETVATTIDLDVAGLGSAVQNVEMRNMHFNSSFDTITDRHHMQ
QAPSKLTLAADGTVVLRYTLSNNVAINQSMNEKKYFGNSVSGGSEPHRITVAGGAKSLQVNNVAVPSGYA
EAQLRLTVALFPGEDDTPDSLLQIDSLTINGQTVETPLDWRGRKQNAAERYFNTLEIPVPADILQANNTI
SVDFRHNGDLTVANLIIKDYSTTPVRN

>WP_041522672.1

MRVKRFSRLTLACAVSSVVAGAMASHALAQTVQVQAENFTAFGGSYADGQPQAVSTYAVNGVQAINYVNR
GDYVEYEVNIAEAGVYTVQYLIGTAVPSGAEVELSVKTGNTWQSQGTTTVPAGGWDNFQPLDGAHEITLP
AGNISIRLTGSGANDWQWNLDEFALTLTQSEVEPETPSDISQEAEDYHAVSGTFADGQAAPVSTYSVNGS
TALNYVNRGDAIEYQISVVRAGIYDLTYFIGTAITSGAQVEFQVKQNGNWVSQGAKSVTASGWDNFQAQI
AGHTVQLPAGVVEVRLLASGSNDWQWNIDKFDLAFVSDSDASSSSVSSSSSVSSSSSSISSSSSSVSSSS
TSSSSVSSSSSSSSVSSGSSSSQVPGGVVTVSGDFSLEAENFNAVGGEVEIYSVDGGHAVNYFNAGDYLEF
DIDNAQGGLYEAVYRVGTGNASGSAVGLMLTDHQGDLVLKNKTDVVSQGGWDVFYDVTSSTRFNVYPGIS
KVRITGAGTEDFQFNIDNIVFKYLGPVDDNLDGDGDGVADVNDICSSTPSGETANGEGCSPSQLDTDIDG
INDANDQCPNTALGAFVNAEGCESSGGDDDDFDGVFNDNDQCPNTFGINVGSDGCSASGRDNDGDGVVNA
EDVCPATASGEMADAEGCGSSQVSNVASVDVFVNANIEHVVNGVSDFGRDRHMTMHSAIYEKDWDGHSDK
LNYVLNTLDVYMGRDNGSSTWKFQETPEDPNRANKADLDWMTDRGAQLRADYDSVELYKRFSEDKTQLIA
GTNPHPLYPTLSWNDNGMTWTGWQPKDTATSAEWVGKYLESYFALSSNGNVGEPLPTYWEVINEPDMKMK
TGQFMVTNQEQIWEYHNQVAQEIRSRLGSEAPLIGGMTWGQHDFYRRDGISRYQDDSYDQWIVNEDPAAE
AEAEAFFQSRMSTTVDDTRELDWYQWDVMWKGFMDAAGDNMDFYSVHIYDWPSVTYGGNATLRRGGHVQG
MLDMMEWYDVHENGVSNRKPIVLSEYGAVQGAWERQAHEERFDVANLKAFNGMLMQFLERPDYVIKSMPF
TPARPLWGYLGSQDGYTTIGVCAFKEVPDCNTRYHYSMMIEENLNQGDWRWSSYIHFYELWQGVNGTRVD
TASTDPDVQVDSYVDGDNVYVIINNLENETTTINLDMNGLSAAVSDVEMRQMYFENQDQHTELDRRHMAR
SPDSLTLNPDATVVLKYSLAGEQQVAEQVVEKKYFGNSVSGGSVPHRVSIAGGAVPVSIDNVQVPSGPAE
AMLRLTVAMYMSEDDVPNGNLTINTLTINGVEVDTPIDWRGPADYQADRYFATLEIPVPVEVLQSNNDIQ
VDFRHNGELTVANLVVWDFTTSPGR

>AFV00504.2
MRTPFQRSALAVALSCASLGLAANAVAADYRLEAENFTAVGGTYNDNQPQKISIYNVNGATAINYVNKGD
YADYSLNVATAGTYSLTYFAGTSIVGGEIEFQINTGSGWTSLKKTAVPQGSWDNFQSLNGGSVVLPAGNI
QVRLVGSGSHDWQWNLDALELTLQSAPTPTPTPTPTPPPSALTVQAESFASVGGSYADGQPQKISVYTVN
GATAINYVNKGDYAQYSITVADAGNYDLAFYAGTAVTGASIHFQVLEGGSWVTKTQTNVPNVGWDNFQLL
SGGTVALSAGTHQVRVYGGGTNDWQWNLDRFVLTKQSGTTPTPTPTPTPTPTPTPTPTPTPTPTPTPT
PTPTPTPTPTPTPTPPVGSVPVSGTFVLEAENYQAVGGEVETYPINGGLAVNYFNKGDYLEFYVTLDKSG
LYSASYYVGTGNASGTAVGLMATDHQGKLVEKSITPVTSQGGWDNFYTANASSQLNLFEGAATLRIYGAG
TEDFQFNIDRITFTRVGDADLNLDADGDGVPDVSDTCASTPAGEQANSVGCAPSQLDTDKDGINDKLDQC
PNTALGAFVDAKGCESSGGDDDDFDGVHNVFDQCPATPFGVNADASGCSVGADSDGDGVANSEDQCAATP
AGEFANEAGCSSSQVANTQVATVTVNANIKHTVNGVSDFGRNRHMTLHSAFYEQDWNTHHDKLNYLINTL
DVYMGRDNGSATWRFQDTVQDPLKPNWPDMNYMVTRGKELREAYAADELLARFPESKTEMINGTNPHPTY
PTLSWYDNGFTWDGWQPKNIETSAAWMGQYLYEYFGHSGNGYVGQPVPKYWEVINEPDMKMKTGQFMVTN
QEAIWEYHNLVAQEIRAKLGNEAPMIGGMTWGQHDFYRRDGISRYADDNYDAYIKVEGDPAAEAAAKAFY
RDAMVTSVDDTKDQDWYQWDVMWKGFIDHAGHNMDFYAVHVYDWPSVNYGGMPTLRRGGHLNAMLEMVE
W
YDVYSNGAANRKPIVLSEYGAVQGGWDYIGHEERYDVESLKSFNAFLMQVLDHPDYVIKSMPFTPVKPLW
GYLPGGCGYKPVPDCDTRYHYAMLIEPTLNSGNWEWSSYIKFFELWKGVDGTRVDSVSSDADVQVQSYVK
DNELFVVINNLETTDTTVNLNVAGLTGSLQNVELRNMHFSNQDQETHLDYHHMKQAPSSVTLKGSATVVL
RYSMAANVAVNQSMTEKKYYGNSPSGNVPHRISVNGGAKSLHINGVSVPAGYAEAKLLLTVSLYPGEDDM
ANGHLSIDSLTVNGVAVTAPIDWRGQRVNGAERFFNTLEIPVPVSALQANNVITVDFHHNGELAVANLLV
KEFSAIPAR

>WP_026286894.1
MSVRVKRFSRLALACAVSSVVAGAMASHALAQTVQIQAENFTALGGSYADGQPQAVSTYTVNGVQAINYV
NRGDYVEYEVNIANAGIYTVQYLIGTAVPSGAEVELSVKSGNTWQSQGTTTVPTGGWDNFQPLDGTHEVT
LPAGNVSIRLTGSGANDWQWNLDEFSLTLTQSETEPEAPSDISQEAEDYHAVSGTFADGQATPVSTYSVN
GSTALNYINRGDAIEYQISVSRAGIYDLTYFIGTAITSGAQVEFQVKQNGNWVSQGAESVTANGWDNFQA
QVAGHTVQLPAGVVEVRLLASGSNDWQWNIDKFDLAFVSDSGASSSSSSSSVSSSSSSVSSSSSSVSSSS
SVSSASSSSSSVSSGSSSSQVPGGVVTVSGDFSLEAESFNSVGGEVEIYSVNGGHAVNYFNTGDYLEFDI
DNAQGGLYEAVYRVGTGNASGTAVGLMLTDHQGNLVLKNTTDVVSQGGWDTFYDLTSSTRFNIYPGISKI
RITGAGSQDFQFNIDNIVFKYLGPVDNSLDGDSDGVADINDTCPSTPSGETANGEGCSPSQLDTDVDGIN
DAEDQCPNSAIDAFVNSVGCATSGGDDDDFDGVFNWDDQCPNTFRMNVGPDGCLASGRDSDGDGVVNSVD
VCPATPGSEFANGQGCSSSQVSTVESVDVHVNANIEHIVNGVSDFGRDRHMTVHSTVFEQDWEGHADKLN
YLLNELDVYMGRDNGSATWKFQDTQEDPNRPNMADLEWMEGRGAELRQAYEDNPFYSRFPESKTQMIAGT
NPHPVYPTLSWYDNGKTWAGWQPMNIETSAEWVGKYLESYFANSGNGNVGEPTPTYWEVINEPDMKMKTG

AFMVTNQEQIWEYHNLVAQEIRSRLGSEAPLIGGMTWGQHDFYRRDGISRYADDSYDQWIVADDPAVEAE
SEAFFQSRMATTVDDTRALDWYQWDVMWKGFMDAAGDNMDFYAVHIYDWPSVNEGGKGTLRRGGHVQGML
DMMEWYDVHENGMNNRKPIVLSEYGAVQGGWDNLPHKPRYESEVLKSFNGMLMQFLERPDYVIKSMPFTP
AKPLWGYVGAQDGYTTLGVCGYKEVPDCNVRYHYAMLIEENLNQGDWTWSDYIRFYELWDDVDGTRVDTR
SSDADVQIESYVDGDEVFVILNNLEDESTTVNLNLHGLSLGNVSKVEMRDMHFDANHKTQLDRRFMDEAP
STVTLAPDGTIVLRYTMNNNVAVNEQVQEKKYFGNSVSGGSEPHRVSIAGGDIPVSINNVQVPSGPAEAM
LRLTVAMYMSEDDVPNGNLTINTLTINGVEVDTPIDWRGPADYQADRYFATLEIPVPVEVLQSNNDIKVD
FRHNGELTVANLVVWDFTTSPGR

>WP_010133215.1
MKLPYPRRPLALAQSVLAAGLAFSTAATAADYRIEAESFSSVGGTYADGQPQKISVYNVNGVTAINYVNR
GDYAEYALQVAEAGTYNLQYLIGTSMTSGAEIDFQIGSGSNWTSLVKKAVPAGHWDNFQPLDAGNVSLPA
GTINLRVVGSGSNDWQWNLDALELTLVSTGGSSSSSSSSSSSSSGGSSSSSSAGGSTEFTVEAESFAQVGG
TYADGQPQKISVYSANGATAINYVNKGDYAEYSVSVPQAGNYDLTYFAGTAVNGAQIDFQLNSSGSWQTL
AQTNVPNIGWDNFQPQAADTVYLPAGSQQIRVYGGGSNDWQWNLDRMEFAYTGGSSSSGGSSSSSSSSSS
SSSSSSSSSSSSSSGGSSSSSSSSSSSSSSSSSSSSSGGSSSSSSSSGGTSPGNGSPVSGTFTLQAESAHVV
GGDIDTYAINGGMAVNYFNSGDYLEYNLSLDQSGLYRPKFYVGTGNTSGTAVGLMATDHEGELVIKNTTD
VVSQGDWDSFYLVNASSEVNLFAGDLTIRIYGAGSEDFQFNIDYAIFERVGDADLNLDGDGDGTPDVSDQ
CPSTDPAETANSVGCAPSQLDTDEDGINDAEDQCPTTGPGEFVNAVGCASPGGDDDDFDGVLNTTDQCPN
TPYGQNVDPTGCSSFSDSDSDGVADSADNCPSTAAGAFANESGCSAAQVGNTHSATVTVNANIAHSVNGV
SDFGRARHITAHTAIYEQDWVGHSDKLNYLLNTLDVTLGRDNGTATWKFSDTKEDPNRENWPDMDYMVTR
GQELRENYEANAFYKRFSPESTELIAGTNPHPTYPTLSWYDNGKTWHNWQPMDIQTSAAWMGQYLKHYYA
NSSNGFIGDPMPKYWEVINEPDMEMKTGKFMVTNQEALWEYHNLVAQEIRAKLGNEAPLIGGMTWGQHDF
YRRDGISRYGDDNYAQWITAEDPAEEAAAEAFFENAMATTVDDTRDQDWYQWDVMWKGFMDAAGHNMDFY
AVHVYDWPGVDGDNMSVLRRNGHLPAMLDMMEWYDVYQNGEANRKPIVLSEYGAVQGGWDYLVHNNRYES
EVMKSFNAMFMQILDRPDYVIKSMPFTPAKPLWGYKPFGCGYDEVRTCTAPYHYSLMKETALNSDEWKWS
DYIQFYELWADVDGTRVDSVSSDADVQVQSYVNDNELFVILNNLETYATTINLNVAGLGGAAVQNVEMRN
MRYDSSYDTVTDRHHMQQAPSTLTLGADATVVLRYTLGNNITINQSMDEKKYFGNSVSGGSEPHRIAVAG
GAKTLSINNVSVPAGYAEAQLRLTVALFPDEDDKVDTNLGIVSLTVNGEVVETPIDWRGRRANNAERYFN
TLEIPVPVELLQANNTIAVDFRHDGQLAVANLVIKEFSAAPVRN

>WP_011469132.1
MRNLNKNKVHILRAAIAASMSVLPLAAGAADYVIEAENFVAQGGTYVDGQPNKVSVYSVNGATAINYVNR
ADYTDYQINVATHGYYNVQYAIGTSVASGAAIELLVQNGSSWESQGQTNVPVGHWDSFQPLNASHEVILP
AGTVNLRVYGAGSNDWQWNLDSISLTLESAINPQPDPDPDPSPQLVKTEAEAFNAQSGTFADGQPTPVSI
YTVNGKTAINFVNKGDAVEYNLVAPAAGSYALKYSIGTSVASGSEVEFFVLKNNVWVSQGKTPVPAVGWD
NFTSVASAQTVELAAGSNKVKLVGAGTNDWQWNLDFFELTLGNVEPEPEPEPEPEPEPEPEPEPEPEPEP
EPEPEPEPQPEPDGDPVPVSGSFKLEAEHFQKVGGEVQIYSLSPGNAVNYFNSGDYLEFYVDLDAGGLYE
ASFRVGTGVASDVAVGLMVTDHKGDLTLKSVTPVTDQGGWDAFYNLTAQSQLNIYSGINTIRITGAGSAD
FQFNIDSITLTRVGPINPALDGDNDGVPDTSDNCPSSPANETANAEGCVPSQLDTDEDGINDKIDQCDAT
PAGDFVDALGCTSTGGDDDDFDGVLNGADQCGNTPYGMNVNAQGCSVFSGSDADNDGVANSEDTCANTPA
LEFANEQGCSSSQVANTHVVNVSVNANFKRSVNGVFDFGRRRHMTAHTAIHEPDWVGHTDKLNYLFNTLD
VYMGRDNGSATWKFNDTTEDPNKPNWPNMDYMVERGKGLREAHDQNPLFKRFSAEKQLLIAGTNPHALYP
TLSWFPNAFTWSGWQPKNIETSAAWVGQYMEHYFANASNGYVGEQLPEYWEVVNEPDMKMKTGQFMVTNQ
EAIWEYHNLVAQEIRDHLGAEAPPIGGMTWGQHDFYRRDGISRFADDSYDQWITNDDQVLQAEARAFYRN
AMATTVDDTRDQDWYQWDVMWKGFMDAAGDNMDFYSVHIYDWPGENVGDTTVVRRGGHTSAMLEMME
WYD
VKRNGFNNRKPIVLSEYGSVNGAWDNRAHEERYDIASIKAFNGMLMQFLERPDYVIKSLPFTPAKPLWGY
LPGGCGYDDAVACTTRYHYAMLIEDELNSGNWEWSSYIKFYELWADIDGTRVDSKSSDVDVQVDSYVKGN
ELFVILNNLEAADTTVNLDVSGIASVQNVELRNMHFDIQETHLDRHHMSAAPKTVTLAADATVVLRYTLA
SSVAVNNTVVEKKYFGESVSGGIEPHRISVAGGAKTLYINNVSVPSGYSEAILRLTVSLYPDEDDKVGGH
LSLDSITVNGTAIEAPIDWKGPKANRAERFFGVLDIPVPVELLQSTNTIAVDFRHNGELTVANLIVSEFT
SEPNR

>WP_016389816.1
MRTPFQRSALAVALSCASLGLAANAVAADYRLEAENFTAVGGTYNDNQPQKISIYNVNGATAINYVNKGD
YADYSLNVATAGTYSLTYFAGTSIVGGEIEFQINTGSGWTSLKKTAVPQGSWDNFQSLNGGSVVLPAGNI

QVRLVGSGSHDWQWNLDALELTLQSAPTPTPTPTPTPPPSALTVQAESFASVGGSYADGQPQKISVYTVN
GATAINYVNKGDYAQYSITVADAGNYDLAFYAGTAVTGASIHFQVLEGGSWVTKTQTNVPNVGWDNFQLL
SGGTVALSAGTHQVRVYGGGTNDWQWNLDRFVLTKQSGTTPTPTPTPTPTPTPTPTPTPTPTPTPT
PTPTPTPTPTPTPTPPVGSVPVSGTFVLEAENYQAVGGEVETYPINGGLAVNYFNKGDYLEFYVTLDKSG
LYSASYYVGTGNASGTAVGLMATDHQGKLVEKSITPVTSQGGWDNFYTANASSQLNLFEGAATLRIYGAG
TEDFQFNIDRITFTRVGDADLNLDADGDGVPDVSDTCASTPAGEQANSVGCAPSQLDTDKDGINDKLDQC
PNTALGAFVDAKGCESSGGDDDDFDGVHNVFDQCPATPFGVNADASGCSVGADSDGDGVANSEDQCAATP
AGEFANEAGCSSSQVANTQVATVTVNANIKHTVNGVSDFGRNRHMTLHSAFYEQDWNTHHDKLNYLINTL
DVYMGRDNGSATWRFQDTVQDPLKPNWPDMNYMVTRGKELREAYAADELLARFPESKTEMINGTNPHPTY
PTLSWYDNGFTWDGWQPKNIETSAAWMGQYLYEYFGHSGNGYVGQPVPKYWEVINEPDMKMKTGQFMVTN
QEAIWEYHNLVAQEIRAKLGNEAPMIGGMTWGQHDFYRRDGISRYADDNYDAYIKVEGDPAAEAAAKAFY
RDAMVTSVDDTKDQDWYQWDVMWKGFIDHAGHNMDFYAVHVYDWPSVNYGGMPTLRRGGHLNAMLEMVE
W
YDVYSNGAANRKPIVLSEYGAVQGGWDYIGHEERYDVESLKSFNAFLMQVLDHPDYVIKSMPFTPVKPLW
GYLPGGCGYKPVPDCDTRYHYAMLIEPTLNSGNWEWSSYIKFFELWKGVDGTRVDSVSSDADVQVQSYVK
DNELFVVINNLETTDTTVNLNVAGLTGSLQNVELRNMHFSNQDQETHLDYHHMKQAPSSVTLKGSATVVL
RYSMAANVAVNQSMTEKKYYGNSPSGNVPHRISVNGGAKSLHINGVSVPAGYAEAKLLLTVSLYPGEDDM
ANGHLSIDSLTVNGVAVTAPIDWRGQRVNGAERFFNTLEIPVPVSALQANNVITVDFHHNGELAVANLLV
KEFSAIPAR

>BAF44076.1
MITSSKKIVSAMLSTSLWIGVASAAYAETTNVEAEGYSTIGGTYQDGNPQPINIYSVNGVQAINFVNRGD
FAEYDVSVSTAGEYSIEYLIGTSIASGSAVEISVLVDGNWQSAGSTNVPLGQWDNFQALAANNNISLAQG
TNRIKITGAGTHDWQWNLDAFSLTLVTPENPDNPDNPDNPDDGNTGQPGTPFTIEMEAFDATGSDDPRAQ
GMVIGERGYPEDKHTVVDSNQTTDWVDYNINFPVSGNYRIEMLASGQTSHATAILFVDNVQINEVAVDTG
NQAVFLDFELTDSTYISAGAHTIRVQSGSQINEFSWMWFGDALTFTPLDGGSTDGDADNDGVLDSVDTCP
NTPAGAQVDANGCEIIVDNDTDNDGVDNSIDQCPNTPAGAQVDANGCEIVAVVDADNDGVEDSLDMCPNT
PAGAPVNGQGCADSQLDADNDGVSDDIDQCPSTPAGSVVDGTGCIVVTPPADSDNDGVVDTLDMCPNTAA
GLTVDSQGCALSQLDSDNDGVTDDIDQCANTPSGETANATGCSSSQEGGGTDPDTPQPGLLYGELAGAMN
VSDTNPNWERTTDLLQTEDSVKGNTTEVYTGFIYDADGHISFYEHIDDSVRLYIDGVLVLSNDSWEASSQ
TTDLNLTPGTHEIELRIGNADGGSGAVDGIGFGIDVDGGTNFVHPSTLSESIFTSVGEETGNPDLEQEGD
IIVELESFVFTSTNGRVGSDSVEGFSPTATGVNWVTNGDYGDYMVTFEEPGTYGAYITISAANDGSYGAR
VDVDGWPVAWGYFGGTGSWDVSSENLLYGGTFVVEQAGEKVVRVEAIGGSDWQWSGDRVRFTRLGDVTAI
PSPIYNPDDHFVAEIQGPQTDVTYLKKPVEIPANKKVLKSDVWYTYPQNRELEGYDNFGATGAFWGHPPE
HDFYDDTVIMDWAVDAVYAFQAEGYEYTARGEFDWGYGWFTEYTTNPQPHYVRTLDDRNVRMTFMGYLSH
DGYNNNWLSNHSPAFVPFMKSQVDQILKANPDKLMFDTQTNSTRSTDMRDFGGDFSPYAMENFRVWLSKK
YSTGELAALGINDINSFDYGDFLRAQGVTHTSWSNAGDTLSGNIPLQEDYIYFNRDVWNQKFAEVLDYIR
QQQPDIEIGASTHLFESRGYVFNENLTFLSGELNLGARTTISELPTNILVHLKGAQAVDKTLVYFPYPWE
FDELRLQDAPRFGRGWVAQAYAYGGLFSIPANVWVGGEVWTWSPGADNYRDIYLFVRAQADLLDDYTSYS
KVGLVHAMYSSMKAGFIDGGNQIQSSTKLLTEGNINFDLLVFGDEGYPVVPRPEDFDKFDHIFFDGDEQY
LTAEQQALLDQQGDKVRHIGQRGTVSGIEITVSISGTESNETVSAVSRIHETDAAAPYVVHLVNRPFAGG
VTPTLNNVEVAIPQSYFPEVVTGATLHLPDGTSTSLTLSTNADGDVVLPVNNLEVWGILELAH

>BAD86832.1
MRLSKSQGILPLAHAVLAAAIAYSTAATAADYRLEAEDFTNVGGTYNDGQPQKISVYTVNGITAINYVNK
GDYAEYTLSVPQAGQYDLTYFAGTAIDGARIDFQVNNNGSWQTLARTDVPNAGWDNFQPLPAGSIHLSSG
SQQIRLFGGGDHDWQWNLDKMELAYIDDSSSSSGGGSTSSSSSGGSSSSSGSGSSSSSGGSPEEGGHVSGT
FKLEAESAHHVGGEIDTYAINGGVAVNYFNSGDYLEYNLHLDQSGLYRPKYYVSTAHSSGVAVGLMATDH
EGALVTKNTSEVQSQGGWDSFYLLNAASDINLFSGDLTIRIYGAGTQDFQFNIDYVIFERISDVDLDLDG
DSDGIADVNDSCPGTDPSETANSEGCAPSQLDTDKDGIADNRDQCPTTAPGDFVDSEGCASTGADDDDLD
GIANQEDQCPDTPFGENVAPSGCTGFEDSDSDGIANGTDQCPSTPAKEFTNESGCSPSQVANPHSVKVTV
NANIKHSVKGISDFGRNRHITAHTTIYEKDWEGHADKLNYLVNTLDVTLGRDNGTATWKFQDTKEDPNRE
NWPDLDYMVTRGKELRENYEANPFYKRFSADRTELIAGTNPHPTYPTLSWNANGSTWHDWQPMHIETSAA
WMGQYLKHYYANSSNGYIGDPMPKFWEVINEPDMEMKTGKFMVTNQEAIWEYHNLVAQEIRSKLGNEAPL
IGGMTWGQHDFYRRDGISRYADNAYDQWIVADDPAEEAAAEEFFRQAMATTVDDTRDQNWYQWDVMWKGF
MDAAGHNMDFYSVHVYDWPGVNSDAKSTLRRNGHLPAMLDMIEWYDVYQNGQANRKPIVISEYGAVQGGW
NTLAHQPRFESEVLKSFNAMLMQILERPDYVIKSMPFTPAKPLWGYYPGGCGYEEVRNCTAPYHYSLLIE

PVLNSDNWQWSDYIKFYELWADIDGTRVDSVSSDPDVQVQSYVNNNELFIIINNLETVDTTIDLTVAGLN
NAQLQNVELRNMHFDNNFDTQLERHHMKQMPTKVTLAADATLVLRYTLNSTIAINQSVDEKKYFGNSVSG
GSVPHRISVAGGAKNLQVNNVSVPSGYAESQLRLTVALYPSQDDTPDSLLQIDTLTINGHTIETPIDWRG
RKENSVERYFNTLEIPVPVDVLQKNNTISVDFRHNGELTVANLVIKEYTTTPVRH

>WP_051235408.1
MIATLSGGTLAGYHAVAQTLEVEAEAFDGSGGSYADGQPQPVSVYTVNGTDAINYVNHGDYVEYEIELAD
AGTYGVEYLVGTSVSSGSEVEFLVNDGSSWNSQGQTTVPTGHWDYFESVLAPHTVQLPAGTVRIRLHGSG
SNAWQWNMDKFRLTLMESDVEPEPTPGLRQEAENYHAIGGTYADGQPDPISVYTVNGATALNYVNRGDYA
DYQVSVPQAGLYDLTYYIGTAISDGAAVELLVNESGSWVSKHTTSVPASSWDTFNALEATSSVYLPQGVV
DIRLQGAGSNDWQWNLDAFELSFAGTASSSSSASSSSSMPSSSASSSASSSVTSSVSSSASSSASSAPP
SGDPIPVSGTFTLEAEHYQASAGELETYQVDGGTAVNYFNAGDHIEFVINHSDGGLYEAVYRVATGNSSG
TAAGLMVTDHSGDLVLKNTTAITSQGDWDTFYDQTSSTRFNIFPGESRIRITGAGSEDFQFNIDAIEFRR
IGAVDNNLDGDGDGISDINDVCPDTPDSEVANGEGCSASQRDGDEDGISDLIDQCPSTPQNAFVDEQGCE
SSGGDDDDFDGVANESDSCPTAYGVNVGPDGCSATGRDNDGDGVVNAEDTCSATPAGEFANRNGCSSSQV
SNAETVTVNVNANIEHIVNGVSDFGRDRHMTMHSTVFEQDWNGHTDKLNYVLNTLDVYFGRDNGSATWKF
GETPEDEQRPNHADLEWMVTRGEELRQLYENTPLYDRFPESKTEMIAGTNPHPTYPTLSWYSTGFTNTDW
QPKDIQTSAEWMGQYLEQYFAHSGNGNVGEPMPTFWEVINEPDMKMKTGAFMVTNQEAIWEYHNLVANQI
RSRLGSEAPMIGGMTWGQHDFYKRDGISRYADDNYDQWITHEDPQQEAEAEAFYENAMATPVDDTRAQDW
YQWDVMWKGFMEAAGDNMDFYAVHIYDWPSVNMTGPETLRRGGHVQAMLDMMEWYDVHENGVNNRKPI
VL
SEYGAVQGGWDQRAHEERFDAESLKSFNAMLMQFLERPDYVIKSMPFTPAKPLWGYLPGGCGYDDATPCT
TRYHYAMLIEDQLNSGNWEWSSYIHFYELWKDVQGTRVDTASTDPDVQVDSYVDGSNLFVILNNLETAAT
TVDLNVSGLGGNSLSNVELRQLYYPGDDSVLLDRRHMNQAPSTVTLEGNGTVVLRYSTNANIAVNEQVQE
HKYFGDSVSGGSEPHRVSVAGGAVELNVDGVQVPSGAAEAMLRLTVALYPGEDDVEGGNLTLDSLTINGT
AVETPIDWRGPKENSAERYFATLEIPVPVSVLQSNNQILADFHHNGELTVSNLTVWDFSTQPQR

>EWH08789.1
MFKLKHPLLSSGIALACALFGAHTQAEVLVLEAESFDNLGGTYSDGQPNPVTIYNVNGQGAINFVNAGDF
VDYNINAQGGEYSIEYFVGTSVQSGPNIEVLVNSNGTWQSQGSVAVPFGHWDDFQSLTPSHKVNLPAGAS
TVRLLAVGSTWQWNLESFVLTQTSAPVTDSDNDGVNDSQDQCPGTPAGTAVDANGCPTGGGTTPPPSGES
FVIQMEAFDATGSDDARAQGVLVGERGYPQDKHTVVDSVQTTDWVDYTINFPSSGNYSISMLASGQTSHA
TAVLFIDGTEINEVPVHTGNQAEFEEFQLAGSVYVTAGTHTVRVQAQSSTGEFSWLWFGDALTFTNLDGD
NGGGDPVQDADNDGVLDGSDSCSNTPAGEPTDLNGCSDSQLDDDNDGVNNNIDQCPSTTPGASVDANGCE
IAVDNDSDNDGVNNNVDMCPNTPAGEAVNGAGCGASQLDDDNDGVNNNLDQCPNTPAGTPVDAAGCETNN
GGDPGNGGEPGEDEYYHNGQGLLFGRVDGAMNFAGEAGYVANPPNYDVTTDLLETDDAIRGNSTEVFRGE
IYDADGHISFYEHIDDSVRLYIDGQLVLSNDSWENSSQTTDLNLTPGWHDFELRLGNADGGSGAVSGIGF
GIDINGGSNFVHPSTLSPSMFRSSGQVVVDPILPPPCGIQIELEHFDETGTVGRVASDPNDGFVAGDTNV
GWVTNGDFGKYHNVFLEAGTYRAFITVSTPAGGSYGARIDIDAEPFAWGYFDSTGGWDIAAEYELYGGDL
VVDSTGNHTVHIEAIGGSDWQWSGDFVCFAKVSDSTAKQPRVYNPNDHVVAEIDGPATGLQYLKEPVQIP
LANKVLKSDVWYTYPQNRELEGFDDFGATGAFWGHPPEHDFYDDTVIMDWAVNVVDDFQSEGFEYTARGE
FDWGYGWFTEFTTNPQPHYVQTLDGRNVRMTFMGYLSHDGYNNNWLSNHSPAFVPFMKSQVDQLLKANPD
KLMFDTQTNSTRSTDMRTFGGDFSPYAMANFRVWLSKKYSSAQLASMGINDINTFDYGAYLRSQGITHTE
WTNAGDTISGNIPMLEDFIYFNRDVWNQKFAEVLEYIRQQRPNIEIGASTHLFESRGYIFNENITFLSGE
LNLGARTTISELPTNILVHLKGAQAVDKTLAYFPYPWEFDELRIQNAPRFGRGWVAQAYAYGGLFSVPAN
VWVGGEVWTWSPGADNYRDIYQFVRAQANLLDGYTSYSKAGYVHAMYSSMKAGFIDGGNQVQSSVKILTE
DNINFDMLVFGDAGYPVVPRQADFDKFEHIFYDGDLNYLTPEQRAVLDAQGSKVRHIGQRGTIDGLQINV
SINGSVANETVSAVSRIHETDSSAPYVVHLINRPFAGGVTPTLNNVEVAIPASYFPQGVTSAKLHLPDGS
SSTVAVSTNANGDAVVSVNNLEVWGILELAH

>ADY17921.1
MKGFTKHPLLLACGLGLSLSTYAADWDNIPIPAELDPGQSWELQESYSDSFNYSGKPSSFTSKWKDAYFH
NWTGPGLTYWSSDESWVGDGNLIISASRRQGTNQVNAGVVTSKTKVKYPIFLEANIKVSNLELSSNFWLL
SENDQREIDVLEVYGGARQDWFAKNMSTNFHVFFRNNDNSISSDFNDQTHNTPTWGNYWREGFHRFGVYW
KSPTEVTFYINGQKTTKGAWSQVVMKDKDYTGAILDKSRYNMDQEMFIILDTEDHSWRSEAGHIATDADL
ADGDKNKMYVDWIRVYKPTGGSTTPPTGDITPPSGYTNLQVAHSNLCLDVKSGALWNGSTYQQWSCNTGN
NNQRFQFTALGNNEYSISAKVSQLCMELASGSSADGAKVQQWVCNHANTSQVWSLVDKGSNTFEIRNKQS

GKCLEIANNSGANGADLRQWSCDGGTNQRFKFQ

>BAB79291.1
MRALLTAVLGLSCTHALAADWDNTPVPANAGNGKVWELQAVSDDFNYSSSLDNYHSEFTRRWHEGFINPW
TGPGLTEWIDGHAYVTDGNLGIAATRKPGTDKVRAGSITSHDTFTYPLYVETKAKISKLVLASDVWLLSA
DSTQEIDVLEAYGSDRAGQEWFAERIHLSHHVFIRDPFQDYQPTDAGSWYTDGQGTVWSDDFHRIGVHWK
DPWNLDYYIDGQLVRSVSGPNIIDPNGFTNGTGLSKPMHLIINTEDQDWRSDNGISPTDAELANTNKSIY
WVDWIRVYKPVDGGSNGENTDVPASATSIKGRQSGKCIDLASGSSANGANIQQWACGTNNANQKFTFVPV
DSGWYELRTKHNKCVGVGGSSSANGAVVIQWDCFNGQNLHVKPVDLGNGYVELRARHSNKCLDVADASTA
NGADIRQWQCNGNTNQQFSFD

>ODP38961.1
MRGQAIRMMLFASGAIAVLAQSALAQDTVIRTIEDFESSRSIAPQTENASARIVPATNGPGKSLRVDFQP
RAEDDVVLKPATPWDFTGKGDVNLAFDIANPAKISTQIFLIITDANGASQTTMAVVPAGKSVTAYAVLSG
FEAMVQSGMREVPPGWASDETKLFWRTGSKKIDLSRIVSITIRTQAMTTPRSLIFDNFRLRRNPPTDPFF
ITDIVDPFGQAAKVEYPIKIHSEAELKAAAQKELAQLAASNGPPDRSRFGGWASGPKVRGTGYFRTEKVD
GKWWLVDPEGHLFFSSGIANVRMANLETVTGYDFVDSSVRKIDPEELTPEDSRDFAPVAPEVRGTRFLAS
PIRRQMFQWLPSYDEPLGKHYGYRRTFHQGALKHGEIYSFYGANLERRYGDNYMAKWRQVTLDRMKDWGM
TSFGNWIDPMYYDNQKMPYFANGWIIGDFKTLSSGFDYWSPLPDVYDPEFKRRARLTIEQIAREVKGSPW
CVGVFVDNEKSWGRVDTNRNRYAAVINALAKSAADSPAKARFVEMLRGRYPTIAALNAAWKSQYASWDAF
GAGASLPDVEAAVPDLARLFADYADTYFRTVRDEIKRVMPNHMYMGVRMAEWGMPEEVTQAAIKYSDVLS
YNVYREDFHEDTWGFLKKVDRPTIIGEFHIGSTSDTGLYHPGLVIATDQTDRGRIYEQYMNSILANPMMV
GAHWFQYVDDPVTGRAYDGENYNVGWVSNTDMPYPELVAAGKRFNYDLYRRRYGN

>ODP36570.1
MRWRASVKRGSFAILALLSSSAGILDNSAFGEPRKAAAPAQDMLFDFEDGMVPSLLQAANATLSIEKSGR
ETVLRAALHSRENLYTSLNFRPEKPWDWRGGEVVGLAMEIGNPGRESVQINLDIVDGQGRTATRSTVIPA
GGSGTYYAPLKGSDLERDTGLRDDPDYWRMAGRKFTWMWGTKQLDLSSIREFKIGSISLATDRTITIDKV
RIVRNPVIDPNYLKGIVDRYGQAAKIDFAGKVKSDADLRAAARAEVAQLRGAALPDRSRYGGWKNGPKLK
ATGFFRTEKVDGKWAMVDPEGYLYFATGIDNIRMANLTTMTGYDFRPGSVKPRDADDVTPEDSAGLYRVP
DSALAGRFVASQLRRDMFQWIPEYNDPLGDHFDYRREGHSGPLDKGEAYSFYRANLERRYGQTSPASYMK
AWRDVTTKRMIDWGFTSFGNWLDPSYYATAQLPYFANGWIIGNFKTVSSGDDYWAPLPDPFDPLFAERAR
ATARSLASEVRGSPWCAGIFIDNEKSWGRVGTPQGQYGIVINTLSRSASDSPTKAVFVKLLRKKYGTVAA
LNTAWGADIASWGALAKGVVLKEHGAARQADYAMLLKAYAREYFRVVDGALNEVLPKHLYMGVRFATWGM
TPEVIEAASEFVDIMSYNEYREIPHEGAWSFLAKIDKPSLIGEFHMGASDAGLYHPGLILASDQKDRAAQ
YERYMDAVIANPWFVGAHWFQYVDSPLTGRSYDGENYNVGFVSVADVPYPEMVAAAKRMNGRLYPVRFGK
KALK

>WP_051333536.1
MRFTRKKTAVFLPSVLLLSAACWASIAQTSSVTVRTEAEDFAYSDGTWPDGQADPVSIYSVNGVTAINYI
NRGDLVGYYIDIPEDGNYQVSYQIGTAMTSGAEISVQLLENGSWKSYAIMPVSPSGWDNFYPLSSTQDIP
IYAGVQEVRVVASGSNDWQWNLDYFELTKVSDLDTGPVDPPVDPPVDPPVEPPVGTDPIAVSGDFTLEAE
WYQGSQGELDTYTINGIEAVNYFNTGDHIEFYIDLDKAGLYSVSYELASGHRSGVAADLLVTDQDGKLVL
RNTTEVASPYADWDIFFSQPASSQLNIFAGVSTVRLAGAGSEDFQFNIDKLVFTRVGDADLSFDSDGDGI
PDVDDICPNTPPGEVANAQGCSPSQLDSDGDGVNDAIDQCPNTPPGAFVDEFGCESLGGDDDDFDGVPNA
LDQCPNTPFGVNVDAHGCPLSNNDADGDGVEDSLDMCPNTPAGEFADAQGCSASQRSLPLTVKVDVNANI
RHEVNGVSDFGRNRHMTLHSAIYEQDWNGHSDKLNYLLNTLDVYMGRDNGTATWRFNETVEDPNRANWPD
LDWMVTRGVTARQEYENNDFLARFPASKTELIGGTNAHATYPTLSWYPNAITWTGWQPKEIETSATWVAQ
YLKHYYANASNGYVGEPMPKYWEVVNEPDMYMKTGQFMVTNQEMIWEYHNLVAQKVREALGADAPMIGGM
TWGQHDFYRRDGISRFADDAYDQWIVHEDPVIQEEARAFYRSRMTTTVDDTRADNWYQWDVMWRGFMEAA
GDQMDFYAVHIYDWPSIEHGGVMPAIRRGGHVQAMLDMMEWYDVSRGGLAARKPIVLSEYGAVQGAWETM
PHEERFDVEGLKSFNAMLLQFLERPDYIVKSMPFTPAKPLWGYMPSGCAFDDTIACTYPYHYGMLLESEL
NNGDWQWSPFIKFFELWADVDGTRVATHSGDIDVQVDAYVDGKDLFIIMNNQEDYATGIDLGLFGLDGLS
VANVEQRHMYYPDGLEVVLDRRHMKQLPTSVALEANSTMVLRISLDQDIAVNQQVLEHKFFADSVSGGVM
PHRISVNGGAKSLQINGVDLPASPAEAMLRLTVSMFPAPDDVEGGLLTINQLKINGQIVATPIDWRGPAD
NKSERIFTTMEIPVPASVLAANNQIEVDFKHNGELSVASLVVWDYSTAPQRQP

>AIW39921.1
MTPTIHDVVKHSEHDRSVALFDFSTNTPPSTFRFNNIDASMTEDSRLKIHCNSAKNMYTSVFIEPTQGEK
WDWSQMPGFCFAFDAQNLRSRSTQVFINIFDSKGQMHSRCVNVTGETENSFLVELKGEYLKGNTNYYSGF
RSNPAPWDSPFVYATWMWGLMNIDLSDIVQIELSIHGTLIDHELELSNFRLMLSPETNPHYLSNIIDRYG
QNAGFEYPEKVHTDQELADFTTKELQQLKEGAMPDRSRFGGYKEGKRYEATGFYRTEKIDGKWSLVDPDG
YPYFATGIDIIRLANSYTQTGVDYDHSKVEQRSPDDLTPEDSIEKFEVSMEAKQTAFVGSEVRRNCFQWL
PTYEEELGEHYAYMRENFEGALDQGETFSFYAANLQRKYGKDYMQQWREVTMDRMLNWGFTSLGNWTAPE
FYSNEKVPFFANGWIIGNFKTVSSGDDFWSPLPDPFDPVFKERAEATVKQVRAEINDTPWCVGIFIDNEK
SWGRMGTIEGQHGIAIHTLSRDAQESPTKAEFMKVLTEKYGDIAALNARWGTNITSWEALSQGVKGLAHN
EAQLEDYGILLEAYASQYFKVVREALKAELPNHLYLGCRFADWGMTPDVVRAAAKYCDVISYNYYKEGLH
PQPWSFLSEVDMPSIIGEFHIGSKDTGLYHPGLVTAGNQQERGEMYEAYMHSVIDNPYFVGAHWFQYIDS
PITGRSYDGENYNVGFVSIADTPYEPMVEAAKRLHSSMYKRRYQ

>ABG40858.1
MLKVIPWLLVTSSLVAIPTYIHATTEVVVNLNVKHSVEGKSEFERKNHIKLHSTLNDNDWQGEEDKLKYM
MEELDVYFGRDNGGTVWNFNQAIEDPANIGYADPQNIIARGQAQRETNWGQNKSALHQYDGRGDLMIGGQ
PRAHYLGNTSPCCGGSAWQAKGGDAVGDFLGQYVNEFFRSAGDPVTKGHLAPVYFEVLNEPLYQVTDAPH
ELGLEQPIPPIDIFTFHNDVADAFRQHNTHIKIGGFTVAFPIFEQREFARWEERMKLFIDTSGSHMDVYS
THFYDLEDDNRFKGSRLEATLDMIDQYSLLALGETKPHVISEYGGRNRPMENAPWSALRDWWFLKTASPM
LMQFLSRPDSVLTSIPFVPIKALWGTAADGTPYNWRLLRQQKEAPNETGENWVFTEMVKFYQLWSDVKGT
RVDTFSTNSDFLIDSYVQNDKAYVLISNLTEQAEKIVVHKYGAPASSQPTTRIKHLYLKGAAPALDETSH
ASDIQEVTIAAEATMVIEYDYPSDIVINETSQEKKYFATEYLKPISANQISRFNINSVATSALGEGILRV
VVGRKLGKSLAPTIAVNGETLTASAQISGDIQNTRGDFFGVIEFPVPIDLLRTNNEIDVTFGDDGGHIAS
VNLKVFSFTSDVRPSAGPVKGITIEPTSAVVAVGSTLQLNPTITPYFATNQNYFLQSSAPEVATVTQTGL
VSALMQGEARITATTEEGSFIAQVDIEVELPSPTSITFDDQSIYASTVYTAGEAMHVTTEYDAGTGHTVT
AALGGVEYRLRHLTASFGLISDVAIVQDGHAVNTQRGTSSVELALPTNLQASADLPDGEFYFLFVRVVSS
NGETQSTSAFPVSIEAGNIDTSPSLTLDDARKYRDTIYKTDQQLTVTAHYQAGDGNTVTSEQGGVRFYLR
ELDANFGLINDIIIEDASAIGQQVGAATATFSLADLTPSAALPAGHFYYLFAVFNSTNGDKYNIPGVFPI
RIEQEVSELSLTFSEPNLYRSTDYEVGGSLAVSVDFDMGTGNAVSDELGGIRFFLRHLREDYSMVKDIIL
EDANAIGQQSGSASVTFSLANIAASDALPANDFYFLYVLVKSTDGATQDLAVQRINIVSPALVGDYDLDG
DVDINDIQSLIVAIHMRQSIDLSFDMNSDGTVNLLDTRLLMNACTRTRCAP

>ABG40423.1
MIKKYNNKWSLQKVAVTLLLTTTSNSFNIVHAAENKVSVDVNLDTKHRIGNIERFNREKFISLHSAPTEN
EWDHDNKGNNAKSDLIGSFINGYDVYFGRDTGYMKNQLFAQKQDGNRLGFIDESVLTTKGNAAINSFENS
NATRFVNARRFKNRSKDMVVGGQVHPYYPDGTNIGNSNWAFSQKNTDQEPIGTATGHYMGQFLQKYFAQT
NTAAGAPKPAYLEVVNEPLYDLNIAPKDGRDKAPIADIFKYHKSVAQEIRKSNNQALNKNIKVAGYTVAF
PNYDWDNFERWESNDKFFIDTAGADMDVFSIHLYDFPTHPRGEEYRRGSNVEATLDMLEQYSNIKFGRTK
PLLISEYGASVHALRNKPWSQVRDAEKLRGYNALLTHFLERPDVIAKAIPFIPVKAEWGRHGDTGYPYEN
RLMRQQFEKAGQTGTDWIYTDLVKFFELWSEVKGIHVDSWASDLDIMVDAYVQNNKAHIIMTSLEFQDTD
VALKALGIDGNTIKSVQVKTLSYDNNNHAKLATKTMSSVPKTVSLPKESTQIMTITYHNKINIDKVNQQN
KYYATTYKQPIKANVDMYFNIKDVEVGAQGEAVLRLGIGREHGKSLTPTVNVNNVPVEVPTDFRGYDQKQ
GKTRNGRASFFGVIEIPVSHDLLQGSNWIRINFDDAGGFVTSAALQVVNSSDEITRSL

>AHC72907.1
MNASKKLLCVAIVTALTGCSASETKDNVSHSSAAEAQAIGHVEQQEKTLQTLFIATNSSSLKQVKYTNAT
GTVVDSASTGLKVLFHGKDNINSAVEFIPDVAWDWSDLDDFNIAFDIGNEGEHSVQLFLNISDTNGDTYT
RSVSVPVGPQSTYYAKMAGHDLAKSISDDKNEFNFTSGLRSNPDTWHSDDKQFISLWGKKNLKLSGISKI
SLSVQNNLFDKQITINDIRLRQNPPMDTLYLTGVVDQFGQNAKREFDGKVHSLAELNSARDKELKILDGK
WNAPRSKWGGWLNGPKLEATGNFRTAKYQGKWSLVDPEGYLYFATGIDIIRLANSSTMTGYDFPPEVLVK
ADNADVTPSDSQGLNRVADSAAPARFVASELRKDLFTWLPSYDEPMGQHYGYRTGVHSGPLKQGETYSFY
SANLDRKYSEMTPNYMQKWHDVTLDRMRNWGFTSLGNWTDPSFYDNQQVPFFANGWIIGDYKTVSSGDDF
WGNMPDVFDPKFKERALHTVSVIAKEVKNTPWCVGVFIDNEKSFGRSETPQSRYGIVFNTLKLDGSEVPT
KAAFTQMAKDKYTSIEALNAAWGKDISSWSAFDKGIDSVLATDETQAKQQLADYSDMLYAYADKYFSTVD
AAMQTYLPNHLYLGSRFADWGMPMEVAKAATQYVDVMSYNIYKEGLHPKGWGFLEDFDMPSIVGEFHIGA
TDSGLFHPGLVHAANQQDRADMYQDYMGTIIDNPYFIGAHWFQYMDSPITGRAFDGENYNVGFVNVTDTP
YAPMVNAAKDLHKGMYERRFGSK

>AFP32918.1
MRIDKLPFAGALLAILIGAYGCTSNLNNAPSTKQTGNSQLPDFESRAFFSSVLTEHATATMVTDSGVMAG
KHALQLEFEGVNVAEQFSRWPNAKFHPAVQAWDWSAYQSLSIDLTNPDMQAATVILKLADKLGVKGSPPN
QLNYQFRIPAQRTQQLTLEFDGGTKQRPGYWGGTQLDLSSILELQIFVQGPIKEQQVILDNLRLNKASSE
QSVVDTKTIIRKIPTLKNLTSSSQGHSNLVSYERSEGTIITELTNGMEGLLDIRFSANTDYPNVTFKEAY
PWDWSEYQELSLAFDIDNQQNQDLQLFVRVDDAEDQRWGGSADGVNNSLSAMVTLPANSSGSYYLPLKQL
NQQINAGMTGLPPKPNYLAEAIKYGWGQRQLDLSNIVSFQLYLQQPKQDAHLQLKRLRLIPDLDSEQQGF
AQIVDKFGQYTGYDWPKKVHSDEELRTMGHVAKLSLKSSSAMPQRSKFGGWLEGPKSKASGHFRTAKLGD
KWSLVDPLGYPFFATGLSNIRLDDSYTLTGYDSSDQTKSMLSPVRASMFTWLPERGERLADGFSYAEKLH
SGAQQQGEIFSFYAANLQRKYGGSLEQALNSWQQVTLSRMQDWGFTSLGNWTDPAFYNNGSIAYVAHGEI
TGEHARIATPNDYWGAMHDPFDPVFRQSARAMAQGLSGQINRNDPWLIGIFVDNELSWGNQQSDAKHFGL
VAAVLAKDQRNSAAKTAFTDYIKSKYWTIEDLNQSWQTTLTSWVQFERGFDYHSELHQAMRRDYSELLYL
FAEKYYAIVRAELKKELPNHLYLGSRFADWGHTEEVLHAASLYVDVLSFNHYSDDFSSSGPWAQLAELDK
PAMVSEFHFGATDMGMFAGGVVSAKDQEQRAAKYSHYMRGVVAHPNFVGAQWFQYIDAPLTGRAWDGENY
NNGFVSVSDSPYPQLVEAAKKFNQQLYQSRFK

>ADY17919.1
MVEVMKFTKNKIAALLSLTLLGVYGCGSTPSSSDAEGAVEDVGGTIPDFESAAFFKKVKKDHAKAEVVSD
QGVTSGSSALKVNFDSVSEANKFKYWPNIKVHPDSGFWNWNAKGSLSLDITNPTDSPANIILKLADNVGV
MGSGDNQLNYAVNVPAGETVPVEMLFNGTKRKLDGYWGGEKINLRNIVEFQIFVQGPMDAQTVIIDNFNL
VDATGDFIEASGQEVKVSGPIPTVASITSFDEGQPTFVAFDRSAAATVTELKTDMGGLLAVKLAATNAYP
NITFKAPQPWDWSEYGDFSLAFDLESKTDEPLQLFVRVDDAENENWGGTANGVVDSMSSYVTLAPGDDGT
FYLPLGQTGSQIVSGMRAEPPKKSYNAQAISYGWGEKSLDTSNIVSFQLYLQNPTKDVEFNIKSVRLIPN
IDADATRYEGLIDQYGQFTGSEWPKKISEDEELETMGKLAKMSLKSTSQMPGRSIYGGWADGPKLKGTGF
FRTEKVDGKWSLVDPQGNLFFATGVDNIRMDDTVTITGHDFADKDKRSGKEVASEVRRSMFTWLPEDDDV
LAENYDYANWVHSGALKKGEVFSFYGANLQRKYGGTFSEAEKVWKDITIDRMVDWGFTTLGNWADPMFYD
NKKVAYVANGWIFGDHARISTGNDYWGPIHDPFDPEFVNSVKAMTKKLMTEVDKNDPWMMGVFVDNEISW
GNTKNDANHYGLVVNALSYDMKKSPAKAAFTEHLKEKYWAIEDLNTSWGVKVASWAEFEKSFDHRSRLSK
NMKKDYAEMLEMLSAKYFSTVRAELKKVLPNHLYLGARFADWGVTPEIAKGAAPYVDVMSYNLYAEDLNS
KGDWSKLAELDKPSIIGEFHFGSTDSGLFHGGIVSAASQQDRAKKYTNYMNSIADNPYFVGAHWFQYIDS
PTTGRAWDGENYNVGFVSITDTPYVPLVEAAKKFNQDVYMLRYKK

>ABK51379.1
MKIKFLSAAIAASLALPLSAATLVTSFEEADYSSSENNAEFLEVSGDATSEVSTEQATDGNQSIKASFDA
AFKPMVVWNWGSWNWGAEDVMSVDVVNPNDTDVTFAIKLIDSDILPDWVDESQTSLDYFTVSANTTQTFS
FNLNGGNEFQTHGENFSKDKVIGVQFMLSENDPQVLYFDNIMVDGETVTPPPSDGAVNTQTAPVATLAQI
EDFETIPDYLRPDGGVNVSTTTEIVTKGAAAMAAEFTAGWNGLVFAGTWNWAELGEHTAVAVDVSNTSDS
NIWLYSRIEDVNSQGETATRGVLVKAGESKTIYTSLNDNPSLLTQDERVSALGLRDIPADPMSAQNGWGD
FVALDKSQITAIRYFIGELASGETSQTLVFDNMRVIKDLNHESAYAEMTDAMGQNNLVTYAGKVASKEEL
AKLSDPEMAALGELTNRNMYGGNPDSSPATDCVLATPASFNACKDADGNWQLVDPAGNAFFSTGVDNIRL
QDTYTMTGVSSDAESESALRQSMFTEIPSDYVNENYGPVHSGPVSQGQAVSFYANNLITRHASEDVWRDI
TVKRMKDWGFNTLGNWTDPALYANGSVPYVANGWSTSGADRLPVKQIGSGYWGPLPDPWDANFATNAATM
AAEIKAQVEGNEEYLVGIFVDNEMSWGNVTDVEGSRYAQTLAVFNTDGTDATTSPAKNSFIWFLENQRYT
GGIADLNAAWGTDYASWDAMRPAQELAYVAGMEADMQFLAWQFAFQYFNTVNTALKAELPNHLYLGSRFA
DWGRTPDVVSAAAAVVDVMSYNIYKDSIAAADWDADALSQIEAIDKPVIIGEFHFGALDSGSFAEGVVNA
TSQQDRADKMVSFYESVNAHKNFVGAHWFQYIDSPLTGRAWDGENYNVGFVSNTDTPYTLMTDAAREFNC
GMYGTDCSSLSNATEAASRAGELYTGTNIGVSHSGPEAPDPGEPVDPPIDPPTPPTGGVTGGGGSAGWLS
LLGLAGVFLLRRRKV

>ACU52709.1
MKIKFLSAAIAASLALPLSAATLVTSFEEADYSSSENNTEFLEVSGDATSEVSTEQATDGNQSIKASFDA
AFKPMVVWNWGSWNWGAEDVMSVDVVNPNDTDVTFAIKLIDSDILPDWVDESQTSLDYFTVSANTTQTFS
FNLNGGNEFQTHGENFSKDKVIGVQFMLSENDPQVLYFDNIMVDGETVTPPPSDGAVNTQTAPVATLAQI
EDFETIPDYLRPDGGVNVSTTTEIVTKGAAAMAAEFTAGWNGLVFAGTWNWAELGEHTAVAVDVSNTSDS
NIWLYSRIEDVNSQGETATRGVLVKAGESKTIYTSLNDNPSLLTQDERVSALGLRDIPADPMSAQNGWGD
FVALDKSQITAIRYFIGELASGETSQTLVFDNMRVIKDLNHESAYAEMTDAMGQNNLVTYAGKVASKEEL

AKLSDPEMAVLGELTNRNMYGGNPDSSPTTDCVLATPASFNACKDADGNWQLVDPAGNAFFSTGVDNIRL
QDTYTMTGVSSDAESESALRQSMFTEIPSDYVNENYGPVHSGPVSQGQAVSFYANNLITRHASEDVWRDI
TVKRMKDWGFNTLGNWTDPALYANGDVPYVANGWSTSGADRLPVKQIGSGYWGPLPDPWDANFATNAATM
AAEIKAQVEGNEEYLVGIFVDNEMSWGNVTDVEGSRYAQTLAVFNTDGTDATTSPAKNSFIWFLENQRYT
GGIADLNAAWGTDYASWDAMRPAQELAYVAGMEADMQFLAWQFAFQYFNTVNTALKAELPNHLYLGSRFA
DWGRTPDVVSAAAAVVDVMSYNIYKDSIAAADWDADALNQIEAIDKPVIIGEFHFGALDSGSFAEGVVNA
TSQQDRADKMVSFYESVNAHKNFVGAHWFQYIDSPLTGRAWDGENYNVGFVSNTDTPYTLMTDAAREFNC
GMYGTDCSSLSNATEAASRAGELYTGTNIGVSHSGPEAPDPGEPVDPPIDPPTPPTGGVTGGGGSAGWLS
LLGLAGVFLLRRRKV

>BAG71427.1
MKIKFLSAAIAASLALPLSAATLVTSFEEADYSSSENNAEFLEVSGDATSEVSTEQATDGNQSIKASFDA
AFKPMVVWNWASWNWGAEDVMSVDVVNPNDTDVTFAIKLIDSDILPDWVDESQTSLDYFTVSANTTQTFS
FNLNGGNEFQTHGENFSKDKVIGVQFMLSENDPQVLYFDNIKVDGETVTPPPSDGAVNTQTAPVATLAQI
EDFETIPDYLRPDGGVNVSTTTEIVTKGAAAMAAEFTAGWNGLVFAGTWNWAELGEHTAVAIDVSNTSDS
NIWLYSRIEDVNSQGETATRGVLVKAGESKTIYTSLNDNPSLLTQDERVSALGLRDIPADPMSAQNGWGD
FVALDKSQITAMRYFIGELASGETSQTLVFDNMRVIKDLNHESAYAEIADAMGQNNLVTYAGRVASKEEL
AKLSDPEMAVLGELTNRNMYGGNPDSSPATDCVLATPASFNACKDAGGNWQLVDPAGNAFFSTGVDNIRL
QDTYTMTGVSSDAESALRQSMFTEIPSDYVNENYGPVHSGPVSQGQAVSFYANNLITRHASEDVWRDITV
KRMKDWGFNTLGNWTDPVLYANGDVPYVANGWSTSGADRLPVKQIGSGYWGPLPDPWDANFATNAATMAA
EIKAQVEGNEEYLVGIFVDNEMSWGNVTDVEGSRYAQTLAVFNTDGTDATTSPAKNSFIWFLENQRYTGG
IADLNAAWGTDYASWDAMRPAQELAYVAGMEADMQFLAWQFAFQYFNTVNTALKAELPNHLYLGSRFADW
GRTPDVVSAAAAVVDVMSYNIYKDSIAAADWDADALSQIEAIDKPVIIGEFHFGALDSGSFAEGVVNATS
QQDRADKMVSFYESVNAHKNFVGAHWFQYIDSPLTGRAWDGENYNVGFVSNTDTPYTLMTDAAREFNCGM
YGTDCSSLSNATEAASRAGGLYTGANIGVSHSGPEAPDPGEPVDPPIDPPTPPTGDVSGGGGSAGWLSLL
GLAGVFLLRRRKV

>BAG71428.1
MVEVMKFTKNKIAALLSLTLLGVYGCGSTPSSSDAEGAVEDVGGTIPDFESAAFFKKVKKDHAKAEVVSD
QGVTSGSSALKVSFDSVSEANKFKYWPNVKVHPDSGFWNWNAKGSLSLDITNPTDSPANIILKLADNVGV
MGSGDNQLNYAVNVPAGETVPVEMLFNGTKRKLDGYWGGEKINLRNIVEFQIFVQGPMDAQTVIIDNFNL
VDATGDFIEASGQEVKVSGPIPTVASITSFDEGQPTFVAFDRSAAATVTELKTDMGGLLAVKLAATNAYP
NITFKAPQPWDWSEYGDFSLAFDLESKADEPLQLFVRVDDAENENWGGTANGVVDSMSSYVTLAPGDDGT
FYLPIGQTGSQIVSGMRAEPPKKSYNAQAISYGWGEKSLDTSNIVSFQLYLQNPTKDAEFNIKSVRLIPN
IDADATRYEGLIDQYGQFTGSEWPKKITEDEQLETMGKLAKMSLKSTSQMTGRSIYGGWADGPKLKGTGF
FRTEKVDGKWSLVDPQGNLFFATGVDNIRMDDTVTITGHDFADKDKRSGKEVASEVRRSMFTWLPEDDDV
LAENYDYANWVHSGALKKGEVFSFYGANLQRKYGGTFSEAEKVWKDITIDRMVDWGFTTLGNWADPMFYD
NKKVAYVANGWIFGDHARISTGNDYWGPIHDPFDPEFVNSVKAMTKKLMTEVDKNDPWMMGVFVDNEISW
GNTKNDANHYGLVVNALSYNVKKSPAKAAFTEHLKEKYWAIEDLNTSWGVKVASWAEFEKSFDHRSRLSK
NMKKDYAEMLEMLSAKYFSTVRAELKKVLPNHLYLGARFADWGVTPEIAKGAAPYVDVMSYNLYAEDLNS
KGDWSKLAELDKPSIIGEFHFGSTDSGLFHGGIVSAASQQDRAKKYTNYMNSIADNPYFVGAHWFQYIDS
PTTGRAWDGENYNVGFVSITDTPYVPLVEAAKKFNQDVYMLRYKK

>BAD99519.1
MKIKFLSAAIAASLALPLSAATLVTSFEEADYSSSENNTEFLEVSGDATSEVSTEQATDGNQSIKASFDA
AFKPMVVWNWGSWNWGAEDVMSVDVVNPNDTDVTFAIKLIDSDILPDWVDESQTSLDYFTVSANTTQTFS
FNLNGGNEFQTHGENFSKDKVIGVQFMLSENDPQVLYFDNIMVDGETVTPPPSDGAVNTQTAPVATLAQI
EDFETIPDYLRPDGGVNVSTTTEIVTKGAAAMAAEFTAGWNGLVFAGTWNWAELGEHTAVAVDVSNTSDS
NIWLYSRIEDVNSQGETATRGVLVKAGESKTIYTSLNDNPSLLTQDERVSALGLRDIPADPMSAQNGWGD
FVALDKSQITAIRYFIGELASGETSQTLVFDNMRVIKDLNHESAYAEMTDAMGQNNLVTYAGKVASKEEL
AKLSDPEMAALGELTNRNMYGGNPDSSPATDCVLATPASFNACKDADGNWQLVDPAGNAFFSTGVDNIRL
QDTYTMTGVSSDAESESALRQSMFTEIPSDYVNENYGPVHSGPVSQGQAVSFYANNLITRHASEDVWRDI
TVKRMKDWGFNTLGNWTDPALYANGDVPYVANGWSTSGADRLPVKQIGSGYWGPLPDPWDANFATNAATM
AAEIKAQVEGNEEYLVGIFVDNEMSWGNVTDVEGSRYAQTLAVFNTDGTDATTSPAKNSFIWFLENQRYT
GGIADLNAAWGTDYASWDAMRPAQELAYVAGMEADMQFLAWQFAFQYFNTVNTALKAELPNHLYLGSRFA
DWGRTPDVVSAAAAVVDVMSYNIYKDSIAAADWDADALSQIEAIDKPVIIGEFHFGALDSGSFAEGVVNA
TSQQDRADKMVSFYESVNAHKNFVGAHWFQYIDSPLTGRAWDGENYNVGFVSNTDTPYTLMTDAAREFNC

GMYGTDCSSLSNATEAASRAGELYTGTNIGVSHSGPEAPDPGEPVDPPIDPPTPPTGGVTGGGGSAGWLS
LLGLAGVFLLRRRKV

>SEP20511.1
MTEISRTRWGGLADRKAKATGFFRVEPIDGVWWFVDPDGGRFLSKGVTAVNFDHDNIKDTERHPYREACL
RKYGSRDAWRGAAARRLASWGFNTLGAWSEPEIAHAGPTPLASAAGVVYLATAYSDGRGWPQSDLFDSAF
ETFAQQRAREICAPRRDDPTVLGWFIDNELQWGPDWRGENELLPVILRDGAAPCSRRVAVELLRGRYSSV
DEFNAVWGNTASSWDALETEPVTPPPFTRNFFTNDRAQERHPLRARYFADCDAFAGLLAERYMAVSAAAI
RAAAPYHLVLGSRFAYVPQPDVVAATARHVDVISINCYDALPDAVIDAYAAAGRPCLIGEFSFRSDDAGM
PNTQGAGPRVATQAERAAGFSRYATAALRCPNLIGYHWFLHADQPAEGRWDGENSNYGVVTIADEVYAEL
TQAMTALNADAERLHDNATMVARIERHAVG

>SES97693.1
MSYQQITTNKKIIATLLVLLSISGCENAGTQTPKQSEPSVSQTQDVFENEVVLTQLVNTQSADYLEWVAS
NTSHFSHDDKSFTIGFLANENISKMTIKPNKPWDLTAYSPYNLAFDIVNESTESVHLYLSLENKDGDIQS
RSISLPREFSGTVYFPLTGKEAETETGFWGDMPPWNTQDQLMIWRSWRAAQVDLSQIYALNFFTIGLLDD
KQVKISDIRLRENPASDPQWMKNVLDQYGQNARVHTPLHISDDETLLQNTKQELAALDSAPADETRSIYG
GYKNGPKLEATGYFRTQKIEGKWWMVDPEGYIFFSHGPANVRMANLSTLTGVDFKDDSVRHRSADELTPE
DSMGVVTVSEKVRESRYIASTLRHDLFSWLPEYDAPLAKHYSYRRSTHKGAMPHGETYNFYRANLERKYG
ESFMDKWYDVTLQRMKNWGFTSFGNWVDPNFYDRKQVPYFANGWIIGDFKTLSGKVNHWGLMPDAYDPVF
AERAKATIEAIAKDIKNSPWCAGIFIDNEKSWGEREGSVEARYGVILDALSKNAQNSPAKQAFTGYLKAK
YSSIDALNLAWQTDIENWQTFENGVEFESYTQAHVDDLSMLLTRLGEQYFTVVHDTLADILPNHLYMGAR
MANWGMPDEIIKASVTYSDVLSFNIYEDGMQDHYWQFLEQVDLPVVIGEFHIGTATDSGMFNPGIVHASD
QADRARKYKAYMQSVLSKPYMVGAHWFQYVDEPITGRAFDGENANIGFVNVTDTPYPEMIQAVKEVTSTM
YQNRLEK

>SES97569.1
MKHISAAKASGIIFSLVTTLFVSGCENGHQKRDAIAQNSNVIFDFESEHSLASVQLEHAAKSLMTNQDGK
GLSIQFDSKAHHQSSVSFQADSAWNWTQYKDFGVALDISNPNKSSAFVYISVTDIHGASHNRSAVIPAQS
QDTYLIELAGKDLQVESGIRSNPPSWPNKYQSIIWRYGNKNIDLANVAAITFKVIGVNEDKTLVFDNITL
VEAENIDTNYLTGLVDKYGQNAKRDFENKISSDQELLAISQKEQALLTNKNFADRGKYNGWKDGPKLNAT
GFYRVEKYQGKWSLVDPEGYLFFSNGLANVRMANTSTITGFDFDQNYIVQREPGDLTPEDSIGLNRAPKK
AWPTRYKSSPLRADMFNWLPNYEDPMGQHYGYRREVHSGAVERGETYSFYQANLARKYQTDDVDLAQQKW
RETTVKRMQSWGFTSFGNWIDPSYYQMENYPYFANGWIIGDFKTVSSGNDYWAPLPDPFDSEFEQRARVT
VKQIAKEVNNSPWCIGVFIDNEKSWGQMGSIRSQYAIPLNALTLDANSSPTKAHFTALLKQKYVDISKLN
KAWDSQIADWDALDKGISIEQINEAMVSDLSTMLYEFGLQYFQVVDRAMQTYMPNHLYMGVRFADWGMTP
ELRQAAAEVADVVSYNYYKEVINDDFWQFLEALDKPSIIGEFHNGAVDSGLLNPGLVHASDQQDRGKKYA
EYVNSAIDNPYLVGTHWFQYIDSPLTGRALDGENYNVGFVSVTDIPYQPLVDAAKDVNKNIYSRRYTNE

>SET37963.1
MKCVNNTAKLLASSILLTLVACGGSSSTETPPPPPPVQKDTTPDAFAFTAHTNAELETDVESNEITVSGI
DAAATISINNGAYSINNGAYIASNSSVENGDTVRLKLKSSSEANQQVDATLNIGGVKATFSVTTKEAPKT
PDNVQVSLNFDTRHSVGGVDSFDRQKFITIHADVTENGWNDNDVHSRNAPNEDPNLLDNFANDYDVYFGR
NTGSISWNLSRVAEDGSKPGFASEDDLTTLGNGAKWGYLNYSGSRWDAVRKNQHRALDMIVGAQQHPFWP
EGTLTNQGKWALSQTDTVDEPLGTATGHYMGQFINKFFDSNRNDSVTDGQIRPTLIEVMNEPLYDLTTIR
EGQANYVEPADIFAFHNTVADEIRKLNDDVLIGGYTVAFPNFDWDNFERWEQRDKLFIDIAGENMDFYSI
HLYDFPAWNNREQYRKGSNMEATMDMLEQYSLIKFGDTRPLVISEYGAAIHSMFNQGWNPERNTLQMRAA
NSMLMQFMERPDMVLKTIPFFVVKAEWGRTDVPYGPRLMIQKFERDGAGAGDQWVYSDLVMFYQLWAEVK
GTRIETHATDLDIQVDGYVDDATAYIILNSLEFEDTDIDLNAFGINSDSVTEVEIKHLTTLPGAEQASTL
DVETTSDLPSTITLGAESTMVIKITFNESVTIDQQVEETKYYAGEYKKAITAGSEITLSINDIAVPAQGE
SVLRLGVGRAHNKSLSPTILVNGVSLEVPADFRGYDQKQGKVNPGRDGFYGVLEIPVPISALRENNQISV
TFADDGGFIASSALQVLASSQPLSR

>SET37905.1
MHNKMSKLWFASAISCLAILGCSQQASNDNNGVIDELVNFSSQTTLDQNIALYNASTEYNAETRSLKVNF
NTSEHAYSSISFVSEAGWDWSDLNDFNIAFDIANEGEHSTQVYLDITDGNGDNYTRSVSIPVGATKTYYA
KMAGHDLQTPDGDEDVELNFMSGLRSNPDTWQSDEVNFISLWGKKNLDVSNIKRIAFSVQSALYDKTITF

SKVQIRQNPAMDETFLTGIVDQYGQNAKQEFVGKIHNDQELLAARDEELSALKNGYDAETRTKFGGWKNG
PRQEATGYFRTEKINGKWSLVDPLGYPYFATGLDIIRLSNTSTMTGYDFDQGLINQRKASDLTPEDSQKL
NRVSNEAAKTRYVASDLRKDLFTWLPSYDEPLGKHYGYRRSAHSGPLSHGETFSFYSANLERKYADINPD
FMEVWKDVTIRRMQTWGFTSFGNWTDPMFYQNDRVPYFANGWIIGNYKKVSSGNDFWAPLPDVFDPLFEE
RAIVTVKQVAAEVQNNPWCVGVFIDNEMSFGRPDSVASHYGIVLNTLARDGKDVPTKAEFTRVMKEKYQD
IAALNKAWGTDIASWDAFNQGVEGNVESEGQLADFSILLTTYADKYFAIVNKALKEHMPNHLYLGARFPD
WGMPIEVVKASAKHVDVISFNVYKEGLIKSKWDFLKEIDMPSIVGEWHIGASDSGLFHPGLIHAADQADR
AKMYKDYMHSVIDNDYFVGAHWFQYMDSPITGRAYDGENYNVGFISVTDQPYKPIVQAASEVNEAMYNRR
FKAQ

>BAL76845.1
MRRTKWGGVVDDGLSGSGFFRVAERDGVFWLVDPDGGRFLSKGVNTVRFDQDRIGRTERVPYAEACRAKY
GSLQAWRAAAADRLASWKFNTVACWSDEGVASAGAQLLAITPIRELGASFRLHRRDQIFPDVFDPEFSAH
IRTSARERCTLRRNDPGLLGTFIDNELYWSTDARGPDELLTLFLNLPSHRPGRLAAISRLQAHYREFSQF
NAVWRTPARSWEELGRIDRVAPPFVRLPPGGLNDSLETKANLADPAREAFAADCDAFVAVVADKYFEVCV
SAIKAVDQHHLVIGSRFGWQPPRGVIAAAGRHLDVISFNCYEFDPGPIIDAYSAAGKPCLISEFSFRGDD
AGLPNSRGGGPRVATQIERARAFERYVTAALSKPNVVGYHWFEHADQPVEGRFDGEDSNFGTVTIEDEVY
DELTTTMTRLNAAAERIHAAALPAVI

>WP_069321504.1
MRWRASVKRGSFAILALLSSSAGILDNSAFGEPRKAAAPAQDMLFDFEDGMVPSLLQAANATLSIEKSGR
ETVLRAALHSRENLYTSLNFRPEKPWDWRGGEVVGLAMEIGNPGRESVQINLDIVDGQGRTATRSTVIPA
GGSGTYYAPLKGSDLERDTGLRDDPDYWRMAGRKFTWMWGTKQLDLSSIREFKIGSISLATDRTITIDKV
RIVRNPVIDPNYLKGIVDRYGQAAKIDFAGKVKSDADLRAAARAEVAQLRGAALPDRSRYGGWKNGPKLK
ATGFFRTEKVDGKWAMVDPEGYLYFATGIDNIRMANLTTMTGYDFRPGSVKPRDADDVTPEDSAGLYRVP
DSALAGRFVASQLRRDMFQWIPEYNDPLGDHFDYRREGHSGPLDKGEAYSFYRANLERRYGQTSPASYMK
AWRDVTTKRMIDWGFTSFGNWLDPSYYATAQLPYFANGWIIGNFKTVSSGDDYWAPLPDPFDPLFAERAR
ATARSLASEVRGSPWCAGIFIDNEKSWGRVGTPQGQYGIVINTLSRSASDSPTKAVFVKLLRKKYGTVAA
LNTAWGADIASWGALAKGVVLKEHGAARQADYAMLLKAYAREYFRVVDGALNEVLPKHLYMGVRFATWGM
TPEVIEAASEFVDIMSYNEYREIPHEGAWSFLAKIDKPSLIGEFHMGASDAGLYHPGLILASDQKDRAAQ
YERYMDAVIANPWFVGAHWFQYVDSPLTGRSYDGENYNVGFVSVADVPYPEMVAAAKRMNGRLYPVRFGK
KALK

>WP_069319437.1
MRGQAIRMMLFASGAIAVLAQSALAQDTVIRTIEDFESSRSIAPQTENASARIVPATNGPGKSLRVDFQP
RAEDDVVLKPATPWDFTGKGDVNLAFDIANPAKISTQIFLIITDANGASQTTMAVVPAGKSVTAYAVLSG
FEAMVQSGMREVPPGWASDETKLFWRTGSKKIDLSRIVSITIRTQAMTTPRSLIFDNFRLRRNPPTDPFF
ITDIVDPFGQAAKVEYPIKIHSEAELKAAAQKELAQLAASNGPPDRSRFGGWASGPKVRGTGYFRTEKVD
GKWWLVDPEGHLFFSSGIANVRMANLETVTGYDFVDSSVRKIDPEELTPEDSRDFAPVAPEVRGTRFLAS
PIRRQMFQWLPSYDEPLGKHYGYRRTFHQGALKHGEIYSFYGANLERRYGDNYMAKWRQVTLDRMKDWGM
TSFGNWIDPMYYDNQKMPYFANGWIIGDFKTLSSGFDYWSPLPDVYDPEFKRRARLTIEQIAREVKGSPW
CVGVFVDNEKSWGRVDTNRNRYAAVINALAKSAADSPAKARFVEMLRGRYPTIAALNAAWKSQYASWDAF
GAGASLPDVEAAVPDLARLFADYADTYFRTVRDEIKRVMPNHMYMGVRMAEWGMPEEVTQAAIKYSDVLS
YNVYREDFHEDTWGFLKKVDRPTIIGEFHIGSTSDTGLYHPGLVIATDQTDRGRIYEQYMNSILANPMMV
GAHWFQYVDDPVTGRAYDGENYNVGWVSNTDMPYPELVAAGKRFNYDLYRRRYGN

>WP_068375637.1
MNIPPAKGYLLCSCLTLLLCTVLGCNQQAGTNMRQNSEPFLSTVDSFAKQQSLLTFYDPSDLQHQHELIL
SGASSEVISTNGDAKLAVEFSPLHNISELRIQPAQPWNLSPFKQANLALDVENTGATSIQMYLSIENNQG
QSRRYSVSLASDFKGTVYVPVSGKEADTDSGLWGDAPPWTTEDKMLVWRSWRSAGVELETIAALNFFTIG
ILEPKSVQIDNIRLRQNPPTDPNWLVNIIDQYGQNARLDTPLKVHSDEQLQQLAQQELRQLQESSGMPHR
SKFGGYTNAPRREATGYFRTEKVNGKWWMVDPEGYLFFSHGPANVRMANMSTITGIDYRDPSVRVRHVDE
LTPEDSMGIVAVSDQVRATRYVSSELRHNMFEWLPEYSDPLADHYSYRRSTHKGPVPHGETYSFYQANLE
RRYGETEAKSYLKKWQEVTMQRMHDWGFTSFGNWVDPAFYAADKVPYFANGWIIGDYQTLSGETNHWGLM
PDPFDPVFAQRAKSTIDAIAKDVKASPWCVGIFIDNEKSWGEREGSVNARYGVILDALSKNSQTSPAKAA
FSAYLKQKYSTIEQLNSAWNSDLKDWQALDNGVVMTDYSAQHVQDLSTMLAMLGEQYFQVVHGTLAEVLP
KHLYMGARMANWGMPDEIITASLKYSDVLSFNIYEEGMQEHFWQFLEKADLPVVIGEFHIGTATDSGMFN

PGIVHAANQSDRARMYKDYMQSVLSKPYMVGAHWFQYIDEPISGRAFDGENANIGFVTAADIPYPELIQA
VKEVTSTMYQNRVAEQGP

>WP_068375608.1
MKNAPSLSLLIMLSGLCACQPAENVQSPAKQSIAQASNTIMTLAALDGSMPNNIKLSNAKGQITDQGLEI
DFQSKQHPYASVTLVPDEPYNWSEFSDFNLAFDIANKGKHSVQLNLDVSDIDGNNYTRSVNVAVSGKHTY
YGKMSGHDLATPKGDENIELNFNSGLRSNPATWEGDDIQFISMWGKKNLNLNGITKISLSVQNVLHDKSI
TLSNIRLRKNQAKNPEFLTAIVDQYGQNAKLDFPTKIHSDEQLQLITANELATFKDTTRPDRSKFNGWKQ
GPKLNATGYYRSEKVDGKWWLVDPEGYLYFATGLDIIRLSNSTTITGYDFDQSHIAQKAANDLTPEDSQG
LNKVSDKAVTTRFVSSDLRKNMFAWLPSYEEPLGKHFGYRRSVHSGPVKHGETFSFYSANLERKYGEQFM
DKWRSVTIQRMLEWGFTSFGNWTDPSFYQNGQMPYFANGWITGDYKTVSSGNDFWAPMPDVFDPEFAVRA
NMTASVIAQEVKDSPWCVGVFIDNEKSFGRPDSTTAHYGIVLHTLRRDGAEVPTKAEFTRLMQQKYGDIA
ALNKVWDKEIADWASFNQGINSDINNEQQIADYGILLGAYAEQYFKTVHDAVAKHLPNHMYLGSRFPDWG
MPIEVVKAAAKYADVVSYNSYKEGLRPDKWAFLKELDKPSIIGEFHMGASDSGLFHPGLIHAANQEDRAQ
MFADYMQSVIDNPYFVGAHWFQYMDSPITGRAYDGENYNVGFVNVTDTPYPEMVEAAKDVNSKIYQARFE
Q

>WP_068375604.1
MNNVFMPTLITTHQLNRLLILSLSFAVIACGGSGGSATPPSTPPPVTTPVTDTTPNSFSFSFSSIDHVPL
DTLVSSDAISISGINAATAISISGGEFKVNEGEFTASSTTLNNNDKVTVRITSAADYATSSELNVTIGGV
VGSFKVTTVPAPPLGKVVDVNLDIKHSVGGIERFDREKFITIHANHTENDWYAVGENASADLITEFAEGY
DVYFGRDAGGIGWNLNNLPQDPAKAGFVDPAATTERANGVRWNYTNLTTPRAITQRNLEYRNKNLIVSGQ
QHPYWPDGQLTGNLSAASWAFSQIDSANEPFGTATGDYMARYVSQFFKQGETDPYGQTKPAFVEVMNEPL
YDLYDAATNPVELSQVFAFHKTVANTIRNLEINGEKPNQNLKVGGYTAAFPDFDTDNFQEWEERDKLFID
MAGADMDFISLHLYDMPRFRNTVQLRKGSNMEATFDLLEHYTNLSFGAPKPFIISEYGSQVHTMLNSPWS
PERDWLFINSMNAQLMNFLERPNLIEMTIPFIVVKAEWGRISDTVPYSHRLMRQQKEAAGETGDLWVYTD
LVKFYQLWANVNGQRAESKANDLDLQVDAYVDGNKAYIIVNSLEMEATTFSLNTYGLTDNDINQLIKREL
RQENGVAVLDEQTLTSIPNTMEIGAEATIIFEITYQNAIAMAHSATETKYYASTYKQAIVAATTHSFTIA
DVILTDEGEAVLRLGLGRDHGLSLLPTVTFNDVTLSVPEDFRGYDQFYNGNGRENFFGVIEIPVPLSALQ
ATNTINVTFDDAGGFISSLSLQVLNSDKALKRAE

>WP_068375578.1
MTFNKMFKKSAIALLLSLGACSADKQANLANTPLAKDDNVLQLLENFDGEQPALAFSLNGVESQIKTHVG
STNNALALSFNPQEQQAKISFKPAQAWDWSSYKEINFAFDASNPGTESIQLYISIANEAGEVSHQSINIA
AGEQGTYYLLLDGEALDTDMGFKKSGMPAWQSTDEMAFFRYGSPKIDLHKVSEIGLYIKGNLSEKSLELD
NLRLRANPSYDISYRQAYVDRFGQNDKMEFPIKIHSEAELKQEADKELAQLNQSGLMPDRSRFGGWKDGP
RSKATGYFRTEKVNGKWWMVDPDGYLFFSHGLANVRMANLTTLTGVDFKDDSVRYIDPEAVTPEDSMGIV
QVSDEVRKSRYIASDVRHDMFTWLPDYKDELAEHYSYRRSVLFGPVSSGETYSFYRANLERRYGQQAPES
YVKKWEEVTLARFQDWGFTSMGNWVDPAFYTNEKVPYFANGWIIGDYKTLASKHDVWAPMPDAFDPEFVR
RAQITIDTIANEVHNSPWCVGVFVDNEKSWGLREGSVEHRYGLILDAMAKNAQQSPAKAAFTKQLQSKYQ
TIAALNQAWQTQFASWQTLANGVSLSDFPQAMISDLSHMLEMLSEQYFKVVHDALAKAMPQHLYMGARMA
NWGMPEETIKASVKYSDVLSFNIYEEGIQPKAWAFLQDIDLPTVIGEYHIGASRETGLYHPGLVQADGQA
DRAQMYLDYMQSVLASPNMVGAHWFQYVDSPISGRAFDGENYNVGFVSTTDIPYPEMVEAAKKFNATVYP
RRYNDNK

>WP_067157817.1
MLLWDFENGKQPKELKLQNATAEVIQRGSGKALSIRLLSEDNHTSAFSFEPEGTWDWSAMGNFAFALDIS
NAESSSVHLYVKATDNEGRTHSRSFVVPEQSSATYFFEMKGPDLAVDTGIRSNPPSWMSEYQDMIYRWGD
KSLNVSEIDSIEFSVAGVLENKYVVVDNVRLIQPESLDEMYLVNLVDEFGQNNKRDFTNKVHSLEELREA
SERELAQLRKTPLEGRSRFGGWALGPRLKGTGYFRTEKVDGKWSLVDPDGYLFFSTGIANVRLANTSTIT
GYDFDHALIPARKPGDLTPEDSLGLNRVPDVAVPTRYVSSSLRADMFTWLPAYDEPLGKNFGYRREVHTG
AIERGETFSFYRANLQRKYGIDDDKKLMEKWREVTVDRMLSWGFTSFGNWIDPGYYQMDRFPYFANGWII
GNFKTVSSGNDYWSPLPDPFDPLFKERAYITAEQIAKEVKGSPWCVGVFVDNEKSWGQEGSVETQYGLVI
NTLTRDAKESPTKAQFVRLMREKYGDIQKLNKSWGVSIDSWNDFASGISLNIFNDSVIKDLSIMLQHYAD
QYFRIVREAVKHYMPNHMYMGARFADWGMTPEIRAAAANYADVVSYNYYKEGVSDNFWHFLKELDRPSII
GEFHNGALDSGLLNPGLIHASSQADRGKKYAEYMNSVIDNPYLVGAHWFQYIDSPLTGRAYDGENYNVGF
VSVTDIPYSPLVESAKAVNKNLYQRRFGSM

>WP_067155675.1
MKFFASALTALAISSPTIAADWDGLSVPADAGAGNIWQLQDNVSDDFNYSAPANGKSQAFYDRWAEGFIN
AWQGPGLTDYHNPNSRVENGQLVIQATRKPNTDQVYTGAIHSNDSVQYPVYIETRVKIMDQVLANAVWML
SSDSTEEIDIVEAYGSSRPDQTWFAERMHLAHHVFIRDPFQDYQPQDMGAWYSDGRLWREQYSRVGVYWR
DPWHLEYYIDGQLVRTVSGTDMIDPYGYTNGTGLSKPMQIIIDAEDQDWRSDNGILATDAELADSSKNQF
YVDWVRVYKPVPADDNNDDSDVTVSFDFDSFYATGKEGDAVAGDTVTGFNPSGNGNINFNTLGDWAEYSF
NIPEAGDYRLELDTASTVTSGIGADLQIDDIYIGQIRMQATGGWENYQTFSLSNNLTIGAGAHILRVQSS
GSAPWQWNGDKIRLIKVGDSTGNNGGNDGGNDNGQQTQVITLEAENFTSTGGTYDGFQTYTQNGVTAINY
NQRGDYGEYTLSVPSGGNYIFSAVVATPETGAAMALSLNGNALLNLDVPSTGSWSHFSTISAANAVTLPA
GTHTLRITSAGNTSNTWEWNADSFEFSPQ

>WP_067154090.1
MVCPSLTGRIALAFLSLVLTACSSDGDRADTKQPPPSEDKVQQDSQPDAFTFGAVSDAEPGSQQVSNAIT
ITGINTSTDISVNGGEFSINDGPFQNSTGTVNSGDSVVLRLIASTDYGATVTATLTVGGVSAEFRVTTKA
ESTNNEDTVKVDVNLNMRHTVGGESVFDRRKFINIHASNTEQDWVGNNSQSAGAPNEDPDLMTNFLEGYD
VYLGRDTGGMKWQLSLLPEDPSRPGFIDETAAKSNGGGARWNYTTGTSTGAQLARYHEHRATDMVVGGQQ
HPYWPNGDDTGMGWSFSTADSEEEPFGTAVGHYMANFLYEYFNRGAGDTYGQPKPVFLEVMNEPLYDLVD
FPSDKDKGTTPEEVFKFHNAVADAVRAFRDPWDQAVHDNVLIGGYTVAFPDFEKNDFNRWEERDKLFIDI
AGEKMDFLSLHFYDFPDIEGKRKLRRGSNLEATFDMLEQYTLMATGERMPFLISEFGATVHSMMNQPWSP
ERDGYKLRAFNGLMMNILERPDQILKSIPFITVKAEWGRQSETVPYTNRLMRQKKEADTESGDSWVYTEL
VKFYQLWAEVKGTRVDSWASDLDVQVNAYVDGATAYIVLNNLEEETSILDLATLGTGTNPLQEVTVKELS
YNDDGVPVLSETTLEQLPEKITLKPEATLVLQLNYKENITLDEENSETKYYADKYKQPIEAKRKLQFAIS
NVEVSDEGEAILRLGIGRDHGKSLTPSVTINGHKVSVPEDYQGYDQYYDGKGRDQFFGVIEIPVAIEYLT
KDNAVEIEFPDDGGYVSTTTLQVFSTSSAINRGVR

>WP_067154022.1
MSLHTFAPLAVSRSSRSFVFAALFSLTACGGASDPSVPKVQAASTSGQLSEDLVLEDFDKTAIPATVKVN
NGAVSLVDSARGDKALLVKLDLKENNSAALVIAPEEPWDWSEFSGFNLAFDVANKGTESVQIDVTMTDKN
GDSYTRGMVVPADGVWRTYYAKLHGHDQQDPDSAAKNEFNFSSGLRSNPPTWQSDDVMLHSFWGKKLLDL
SGITKISFGSDGSLSNRQYTIDNIRLRANPEMDKNFLTGLLDKYGQNAKVDYEGKIHSDEELRAVVAEEL
AGLSGKLNEGRSKYSGWKDGPRFKSTGYFRTQKVNGKWALIDPEGYLYFSTGIDIIRLSNSSTITGYDYD
QALIPARSDDEVIAEDDQPLNRVNEKAWATRHLVSETRAKMFTWLPDYNDPLGNHYGYRRETQSGPLKHG
ETFSFYSANLERRYGESYPESYLDTWRKVTVDRMLDWGFTSLGNWADASFYQEERIPFVAFADIIGDFGT
LSSGFDFWHPVPDPFDPRFYQRALVAAESVNQQINGSPWCMGVFFDNEQSFGRLESDELHYGIVINALSR
DAADTPAKSAFTEILKKKYGSIEALNKAWNKQVASWEAFAAGMDSSISTDAQLEDYASLLFAYGDQYFGT
INRAMKSIMPNHLYLGSRLPSWGMPDEIVKAAAKNVDIISYNLYEEGLVPSKWEFLAEIDKPSLIGEFSF
GADDQGHFHPGIVISADQKDRGRMFRNYMYSFIDNPYFVGVHMFQYMDSPITGRAYDGENYANGFVSVTD
VPYIELVKAAKEVNGSLYKRRYGDVQLRARSASERGSD

>WP_066968083.1
MRSVLSLAITAAALSGLAACSGERKGSEPSVSAVSSADSNLLAQDFVLENFDSGGIPGSVQVNNGTANLV
DDGAGGKALQVKLNLADNNGAGLVIKPAEAWDWSEFSDFNLAFDVANHGEESVQIDVTMGDKNGDFYTRG
LVVPADGTSRTYYAKLHGHDQEDPKAAAQNEFNFASGLRSNPPTWQSDDIMLHSFWGKKLLDLSGITQIA
FGSDGSLSNRQYTIDNLRLRANPEMDKNFLTGLLDKYGQNAKVDYQGKIHSDEELKKVVEEELASLSGKP
NADRSKFSGWKSGPQLEATGYFRTEKVNGKWAIVDPEGYLYFSTGIDIIRLSNSSTITGYDYDQALIPKR
SADEVIAEDDQPLNRVNEAAWATRELISETRANMFNWLPGYDDELGNHYGYRRETQSGPLKHGETFSFYS
ANLERRYGETYPESYLDTWQRVTVDRMLDWGFTSLGNWAADPFYEQQRIPFVAFADIIGEFSTLSSGFDF
WHPVPDPYDPRFYQRSVVAAKAVSEQIQASPWCMGIFFDNEQSFGRLESDELHYGIVINALSRDAADTPA
KGAFTKVVKEKYGTIEALNKAWNKNVASWEAFEKGMDSTLTTDAQREDYATLLFEYGNQYFGTINKAMKS
VLPNHLYLGSRLPSWGMPPEIVKAAGKNVDIISYNLYEEGLVPSKWDFLAEIDKPSLIGEFSFGSDDQGH
FHPGIVISADQKDRGRMFKNYMHSFIDNPWFVGVHMFQYMDSPITGRAYDGENYANGFVSVADVPYAELV
KAAKEVHEGLYERRFGDVKLEKE

>WP_066965765.1
MKYPYSRRPLALAQSVLAAGLAFSTAATAADYRIEAESFSSVGGTYADGQPQKISVYSVNGVNAINYVNR
GDYAEYSLQVAEAGTYNLQYLIGTSVSSGAEIDFQIGGGSSWTSLVKKAVPAGNWDNFQPLDAGNISLPA

GTVNLRVTGSGSNDWQWNLDALELTLVSASGGSSSSSSSGGSGSSSSSSGGGSTDFTVEAESFTQVGGTY
ADGQPQKISVYSVNGATAINYVNKADYAEYTISVPQAGNYDLTYFAGTAINGGRIDFQHNNGGSWQTLAQ
TSVPNVGWDNFQALAGGSVYLPAGTQQIRVYGGGTNDWQWNLDRIELSYDSAGSGSSGGSSSSSSSSSSS
SSSSSSSSSSSGGSSSSSSSSSSSSSSSSSSGGSSSGGSSPGNGSPVSGTFTLQAESAHVVGGEIETYAI
NGGTAVNYFNSGDYLEYNLSLDQSGLYQPKFYVGTGNTSGSAVGLMATDHEGELVIKNITDVQSQGDWDS
FYLLNASSEINLFAGDLTIRVYGAGSQDFQFNIDYAIFERVGDADLALDGDGDGTPDVSDQCPSTDPAET
ANSVGCAPSQLDTDEDGITDNLDQCPTTGADEFVNAVGCASAGGDDDDFDGVLNSADNCANTPYGQNVDP
SGCTGFADSDGDGVANSADNCPSTPAGEFANESGCSASQVGNSHSATVTVNANIKHSVNGVSDFGRNRHI
TAHTTIYENDWKGHADKLNYFLNTLDVTLGRDNGTATWKFQDTKEDPNKANWPDMDYMVTRGQELRESYE
ANAFYKRFSPESTELIAGTNPHPTYPTLSWYDNGKTWHGWQPMDIETSAAWMGQYLKHYYANSSNGNVGD
PMPKYWEVINEPDMEMKTGKFMVTNQEALWEYHNLVAQEIRSKLGNEAPMIGGMTWGQHDFYRRDGISRF
ADDNYDQWITDEDPEVQEAARAFFRNAMTTTVDDTRSQDWYQWDVMWKGFMDAAGHNMDFYAVHVYDWP
G
VSNDSTSTLRRGGHLPAMLDMMEWYDVHQNGAANRTPIVLSEYGAVQGGWDYLAHNSRYESEVVKSFNAM
LMQILDRPDYVIKSMPFTPAKPLWGYKPFGCGYEEVRTCTAPYHYSMMKESQLNNNDWHWSDYIQFFELW
ADVDGTRVDAVSSDADVQVQSYVDGNEVFVIINNLETVATTIDLNVAGLGSAVQNVEMRNMHFNSSFDTI
TDRHHMQQAPSKLTLAADGTVVLRYTLSNNVAINQSMNEKKYFGNSVSGGSVPHRITVAGGAKSLQVNNV
AVPSGYAEAQLRLTVALFPGEDDTPDSLLQIDSLTINGQTVETPLDWRGRKQNAAERYFNTLEIPVPAEL
IQANNTISVDFRHNGDLTVANLIIKDYSTTPVRN

>WP_066965761.1
MKPHSLPTRLALLTLTATLVACGGGGNGGSNNDQPPQDGGEAQQDTRPDAFSFTAISDAARDTAYTSNAV
TVTGINAMANISISGGEYSVNDGDFTSDAGTVENGDRVSVRVTTGSEYSTTVTASVTIGGVSADYSVTTM
VEPAQPAPPVDDTVKVDFNLNMRHTVGGASEFDRRKFITVHASNTENDWFGGNSQSAGAPNDDPDLMTNF
LEGYDVYLGRDTGGMKWQLSQLPEDGARPGFIDVEAAKTNGGSARWNYTQSNTDNAALARKHEHRATDMI
VGGQQHPYWPNGDDTGMGWSFSTADTQEEPFGTAVGHFMATFLYEYFNRGEDDAYGQPKPVFLEVMNEPL
YELVDYPKDVDEGTTPEDVFHFHNAVANELRAYRDQWGQASHENVLIGGYTVAFPDFEKSDFNRWEERDK
LFIDIAGANMDFLSVHFYDFPAFNGTRQLRRGSNVEATFDMLEQYSLIATGERKPFVISEIGATVHSMMN
DPWSPERDGYKLRALNGLTMNMLERPDQILKSIPFVTIKAEWGRQSDTVPYTNRLMRQRKEASGETGDAW
VYTEFVKFYQLWSDVKGTRVDSWASDLDIQVNAYVDGDTAYLVLNNLEQEDTELSLATLGVAGNSLQSVT
IKELHYGRDGKPVLDERDTTDLPEMYTLKSEATTVLQLTYADTIVIDDDLTETKYYADKYKQAIITDAKL
QFAINDVVVADNGEAVLRLGIGRDHGKSLTPMVTVNGNAVTVPEDYQGYDQYYDGKGRAQFFGVLEVPVD
LEYLNEDNSVEVVFDDDGGFVSTATLQVFNSSAALTRGTRD

>WP_066965750.1
MKTTQGALAALVFSTPLMAADWNGIPVPADAGPGNTWELHSLSDDFNYAAPASGKSASFFERWSEGFINP
WLGPGETEYHAPNSSVEDGNLVIKATRKPGTIKIHTGAIHSKESLTYPLYMEARVKITNLTLANAFWLLS
SDSTQEIDVLESYGSDRPSETWFDERLHLSHHVFIRSPFQDYQPKDAGSWYPNPNGGTWRDQWIRIGTYW
VDPWTLEYYVNGEHVRTVTGPEMIDPYGYTGGTGLSKPMQVIFDAEHQPWRDAQGTAPPTDAELADPSRN
RFLVDWVRFYKPVPADNGGGDPGNGGNPGNGGDGETITVELGNFTDTGKSGAAVAGDTVPGFNRNGADNI
NYNTLGDWGDYTVNFPEAGQYAVELVAASPTTSGIAADIQVDGSYVGTIPMSSTGAWELYNTFSLPDTIY
IASAGNHTIRVQSAGGAGWQWNGDEIRFTKTDGGTSNPPPATGASVTIEAEAFASVGGTFADGQAQPISV
YTANGNTAINYVNKGDFADYTVTIAEAGTYEIHYQAGSGVTGGSIEFLVNENGSWNSKTVTAVPNQGWDN
FQVLDGGSVYLEAGTHQVRLYGVGSNDWQWNLDKFVLSN

>WP_066965748.1
MNFANQWLTAFLICALCLTGCGRSDGNTLAAAETVSGDKLSSAEGLSGTESAEADPVVLWNFENKKLPES
IKLENANGRLVSTGAGQALRLQLQTKAHYAASFTFSADKSWDWSNLGNFAFALDIDNAQPSSVHLYVKAF
DGEGRLHSRSFVVPEQSQNTYYMELKGPDLSVVTGIRSNPPSWNSAYQDIIFRHGEKQLDVSAIERIEFS
VAGVLEDKTLTIDNVRLIKPETLDQNYLNGLVDQFGQNAKMEFANKVHSVEELRDYAGEELADLRHTPLE
GRSRFGGWAEGPKLEATGFFRTEKVDGKWALVDPEGYLFFSTGIANVRLANTSTITGYDFDKAKVPQRTP
GDLTPEDSLGLNPVPDAALPTRYISSPLRAEMFTWLPEYDEPLGQNFGYRREVHTGAIEHGETFSFYRAN
LQRKYDIADEEQLMAKWRDTTVDRMLSWGFTSFGNWIDSDYYQMDRIPYFANGWIIGNFKTVSSGNDYWS
PLPDPFDPLFKERAYITAEQIAKEVENSPWCVGVFVDNEKSWGQEGSTASQYGIVINTLSRSAKESPTKA
QFAQLMQDKYGEIAKLNSAWNIQLQDWDSFARGVALSGFNAAMVEDFSTMLEHFTGQYFKIVREAVKHYM
PNHMYLGARFATWGMTPEVRSAAAKYADVVSYNYYKEGVSDKFWHFLAEIDRPSIIGEFHNGALDSGLLN
PGLIHASSQADRGKKFAEYMNSVIDNPYFVGAHWFQYIDSPLTGRAYDGENYNVGFVSVTDIPYTPLVNA

VKEVNENLYQRRFGGTTDKNKKANR

>WP_066959277.1
MTLKSLAYAVAVSAALASIDSQQAGAAQVLAVPQAAELTPRQLPATIVDFASARERGWLVAEQAEIGAVD
KNTPQSINLRFPASVHTPVLNIVPDQPWDLSKVGDFNLAFDVANLAPVATHFYVELFDTAGNSQSRELSV
PKGYTGTVFFPLAGEKAAMDKGMWANPMPWPTDDMKMVWRSWHAELDLSKVTKIAFYTIGVLQDRALQVG
NIQLRPNPESGADWTNKLVDRFGQAAKKDTPLKVSSEAELKALADRELASLAQHTGPKDRARFGGYKNGP
KLEATGFFRTEKIDEKWWMVDPEGYLFFSHGPANVRMSNLTTLTGIDFKDPSVRVVHADEVTPEDSMGIV
PVSDKVRESRYVINDTRHDMFEWLPSYDDPLADHYSYRRSTHKGPIPHGETYSFYRANLERRYGETAPES
YVRKWEEVTLQRMHSWGFTSFGNWVDPAFYPNEKVPYFANGWIIGDYQTLSGDTNHWGLMPDFYDPVFAE
RARATIEVIARDVKASPWCIGVFIDNEKSWGEREGTVAERYGVILDALSKDAAESPAKLAFTERLKEKYG
NVKALNRAWKSQFDSWDAFAGDASLNHHSDAQVADLSLLLEALGEQYFKVVHGTLQEYLPDHLYMGARMA
NWGMPDEIIKASVKYSDVLSFNIYEEGMQTHQWDFLEEIDLPVVIGEFHIGATMGSDNYHPGIVSAANQK
DRARMYKAYMNSVLEKDYMVGAHWFQYVDEPVTGRAFDGENANIGFVTVTDIPYPEMVEAAKEITFDLYP
KRYGK

>WP_065676849.1
MSVLAKEAVMFSEHDTSISLFDFAEASIPSQFSFENIEAKIVSSGDGITASNHALKIHTHSKENFHTSIV
LEPSDGEVWDWSLLPAFSFAFDATNVGQRSTQLFINLFDHKGQMHSRCANIPSQTSDTYLVELKGEFLKG
KTNFDSGLRSNPASFETPFTYATWMWGLMNVDLTSIVKIELSIHGTLIDHDIVFDNFRLMLNPEPNPAFL
EGVIDQYGQNRDASYSEKVTSDEDLLVRTNNELQQLKEGAMPDRGKFGGYIDGQRLKATGFFRTEKIEGK
WSLVDPEGYPYFATGIDIIRLANAYTITGVDYDHNKVESRASDDVTPEDSKEKVTVPYAAKESAHIANKA
RRDIFQWLPSYEEPLGEHYAYMRELFEGPVERGETFSFYAANLQRKYGQNYLEKWREVTVDRMLNWGFTS
LGNWTNPEFYRNEKIPFFANGWIIGDFKTVSSGDDFWAALPDPFDPVFRERARATVTQVREEIKETPWCV
GIFIDNEKSWGRMGTIEGHHGIAIHTLGRDAVDCPTKLVFVDTLRAKYNTIEALNARWGTRIVSWDTLAT
GVKGLEHNEAQLEDYGMLLEVFASEYFRVVNEELKAQLPNHLYLGVRFADWGMTPDVVRAAAKHCDVISY
NYYKEGLHPEPWKFLPEIDMPSIIGEYHIGSKDVGFYHPGLVCAANQQERSEMYESYMHTVIDNPYFVGA
HWFQYIDSPIAGRSFDGENYNVGFVSTADVPYEPMVEAAQRLHSQMYKRRYQK

>WP_065108449.1
MLNQFDFEHSRGEIKKIDGKEQLVVHLDSKQHHSASFSVKPDKSFNWQSNEPIGFAVSLANPKSTSVFIH
VKVKDSAGLSHNRNIVLPAKSQDNYFMALSGKDLSIETGIRSNPNPWLTDYTPIIWRYGVKNIDLKNVAE
IEFSVHGVPEDKQILLSNLQLIKATKLNDQYLENLVDEFGQSTKIDFINKVKSTDELIAISAKEQASLRK
TVPEGRSTFNGWKAGPKLDATGYFRVEKYQGKWSLVDPEGYLFFSNGIANIRMANTSTITGYDFNTTHIT
PRAEGDFTPEDSIGLNRAPESAWDTRYVSSELRAKMFSWMPKYGEPLSEHFGYRREVHTGAVEKGETFSF
YQANLARKYQSNDADVFMPKWRDTTVDRMLSWGFTSFGNWVDPSFYQLNRIPYFANGWIIGDFKTVSSGN
DYWSPLPDPFDPEFVKRAHFTAKQIAAEVQNNPWCVGIFIDNEKSWGQMGSIESQYGLVINTLGIDASDS
PTKAEFVKVLKTKYTDIGELNRQWGTNFATWKRVETGIEIADFKDEVVTDLSLLLEHYTSEYFAVVSDAV
AQYLPNHLYLGARFASWGMTPEVRSAAAKHVDVMSYNYYKESVNDSFWSFLEGIDMPSIIGEFHNGSMDS
GLLNPGLIHASSQADRGAKYQEYVNSVLDNPYLVGSHWFQYIDSPLTGRAYDGENYNVGFVSVTDIPYQP
LVDAAKAVNQQIYTRRFGDVHVK

>WP_065108447.1
MSALLLGVLVALTACHSQEASVDKASKGQIVNTQNHQADDVFADEVVLKHLVDFNNDEQLSWLKLTDTQT
AVVHNSSNNSQSLKVDFPQTANIPKLTIKPDSPWNVAEFPRANLALNVTNTSDESIQLYVDITNPLGQYQ
SRSISLVAGYNGTIYFPLSGIEAQTDTGVWGDVRPWNTNDDMMVWRSWREKGASFTELSSLSFFTIGNLR
AKSVIIDDIRIRQNPPVKTGNPFDYLVGLIDRFGQNNKQPTALHINSETQLKALADNELSQLQASSGMPN
RSQYGGFTQGPKLNATGYFRTEKVDGKWWMVDPEGNLFFSHGPANVRMANMSTLTGIDYDEPQIRVRTSD
EITPEDSMGIVDVPKEVKEKRYVISSLRHDMFTWLPDYDAPLAKHYSYRRTTHKGPIPYGETYSFYRANL
ERRYNEMDIASYPAVFKPSNYLEENQHLEQKTQHSSQAKYLQAWQDVTAKRMHDWGFTSFGNWVDPGFYQ
SNQVPYFANGWIIGDFKTLSGKTNHWGLMPDPFDPVFAERAKITIDAIAKDIQSSPWCAGIFIDNEKSWG
EREGSVEARYGVILDALSKPASQSPAKKAFVKVLQEQYGAIDNINAAWGAQYTQWSDLNLGLEFADHTPE
LIADLSLLLEKLGEQYFNVVHNTLEKVLPNHLYMGARMANWGMPDEIIKASLKYSDVLSFNIYEEGLQTD
YWQFLEKVDLPVVVGEFHIGTATDSGMYSPGIVHAANQVDRARMYKAYMKSVLEKPYMVGAHWFQYIDEP
ITGRAFDGENANIGFVTVTDVPYPEMIQAVKQVTSTMYQQRYGQ

>WP_065108070.1

MKIKLDTRNALRYSFIATTILSTIACQHDTQNIGQNATPAESVPLVVNSKQAMDLINFNSQSNNIIKPVA
AQTSISDGKLHVAFNSKDNNYSGVSFVPEKAWDWSDLNDFNLAFDIVNTSNHSVQLYLDISDIDGFTYTR
TVNVPVGDNIQTYYAKMAGHDLGVMDSDHKVELNFSSGLRSNPDTWQSNEHQFTSMWGKKNLNTAGIAKI
SLSVQSNLHDKSIDISRVQIRQNPKFDPLFLTDIVDQYGQNKKQDFIGKVHNDLELEQQRVDEAKTFTGK
VAEDRSRYGGWLSGPKLKATGFFRTEKVNGKWSLVDPDGYLYLATGIDIIRLSNSSTLTGYDFKQAYIVQ
PPKDNVTPEDSQKLNRVSDEAIASRFIASNTRTNMFSWLPDYDAPLGKHFGYRRSAHSGPLKHGETFSFY
SANLERKYGQQAKGYMQAWEDTTVNRMLDWGFTSLGNWTDPRFYXSNKIPYFANGWIIGNYKTVSSGDDF
WAPMPDVFDPEFEKRAYATAKVIYDEVKGNPWCVGIFVDNEKSFGRSDSTESRYGIVVNTLTKNGAEVPT
KAEFTRLMQQKYVDINKLNQAWNKSITDWAEFDSGIDSSINNAEQINDYGLLLTAYADKYFSTVNKAVKH
YMPNHLYLGSRFPDWGMPREVVNASAKHVDVISFNSYKEGLTKKSWAFLQEIDMPSIIGEFHIGAKDSGL
YHPGLILASDQQDRAVMYKDYMKSVIDNPYFVGAHWFQYIDSPITGRAYDGENYNVGFVSVTDTPYPYMV
EAAKAINKTMYQQRFKEK

>OBT11990.1
MLNQFDFEHSRGEIKKIDGKEQLVVHLDSKQHHSASFSVKPDKSFNWQSNEPIGFAVSLANPKSTSVFIH
VKVKDSAGLSHNRNIVLPAKSQDNYFMALSGKDLSIETGIRSNPNPWLTDYTPIIWRYGVKNIDLKNVAE
IEFSVHGVPEDKQILLSNLQLIKATKLNDQYLENLVDEFGQSTKIDFINKVKSTDELIAISAKEQASLRK
TVPEGRSTFNGWKAGPKLDATGYFRVEKYQGKWSLVDPEGYLFFSNGIANIRMANTSTITGYDFNTTHIT
PRAEGDFTPEDSIGLNRAPESAWDTRYVSSELRAKMFSWMPKYGEPLSEHFGYRREVHTGAVEKGETFSF
YQANLARKYQSNDADVFMPKWRDTTVDRMLSWGFTSFGNWVDPSFYQLNRIPYFANGWIIGDFKTVSSGN
DYWSPLPDPFDPEFVKRAHFTAKQIAAEVQNNPWCVGIFIDNEKSWGQMGSIESQYGLVINTLGIDASDS
PTKAEFVKVLKTKYTDIGELNRQWGTNFATWKRVETGIEIADFKDEVVTDLSLLLEHYTSEYFAVVSDAV
AQYLPNHLYLGARFASWGMTPEVRSAAAKHVDVMSYNYYKESVNDSFWSFLEGIDMPSIIGEFHNGSMDS
GLLNPGLIHASSQADRGAKYQEYVNSVLDNPYLVGSHWFQYIDSPLTGRAYDGENYNVGFVSVTDIPYQP
LVDAAKAVNQQIYTRRFGDVHVK

>OBT11988.1
MSALLLGVLVALTACHSQEASVDKASKGQIVNTQNHQADDVFADEVVLKHLVDFNNDEQLSWLKLTDTQT
AVVHNSSNNSQSLKVDFPQTANIPKLTIKPDSPWNVAEFPRANLALNVTNTSDESIQLYVDITNPLGQYQ
SRSISLVAGYNGTIYFPLSGIEAQTDTGVWGDVRPWNTNDDMMVWRSWREKGASFTELSSLSFFTIGNLR
AKSVIIDDIRIRQNPPVKTGNPFDYLVGLIDRFGQNNKQPTALHINSETQLKALADNELSQLQASSGMPN
RSQYGGFTQGPKLNATGYFRTEKVDGKWWMVDPEGNLFFSHGPANVRMANMSTLTGIDYDEPQIRVRTSD
EITPEDSMGIVDVPKEVKEKRYVISSLRHDMFTWLPDYDAPLAKHYSYRRTTHKGPIPYGETYSFYRANL
ERRYNEMDIASYPAVFKPSNYLEENQHLEQKTQHSSQAKYLQAWQDVTAKRMHDWGFTSFGNWVDPGFYQ
SNQVPYFANGWIIGDFKTLSGKTNHWGLMPDPFDPVFAERAKITIDAIAKDIQSSPWCAGIFIDNEKSWG
EREGSVEARYGVILDALSKPASQSPAKKAFVKVLQEQYGAIDNINAAWGAQYTQWSDLNLGLEFADHTPE
LIADLSLLLEKLGEQYFNVVHNTLEKVLPNHLYMGARMANWGMPDEIIKASLKYSDVLSFNIYEEGLQTD
YWQFLEKVDLPVVVGEFHIGTATDSGMYSPGIVHAANQVDRARMYKAYMKSVLEKPYMVGAHWFQYIDEP
ITGRAFDGENANIGFVTVTDVPYPEMIQAVKQVTSTMYQQRYGQ

>OBT11464.1
MKIKLDTRNALRYSFIATTILSTIACQHDTQNIGQNATPAESVPLVVNSKQAMDLINFNSQSNNIIKPVA
AQTSISDGKLHVAFNSKDNNYSGVSFVPEKAWDWSDLNDFNLAFDIVNTSNHSVQLYLDISDIDGFTYTR
TVNVPVGDNIQTYYAKMAGHDLGVMDSDHKVELNFSSGLRSNPDTWQSNEHQFTSMWGKKNLNTAGIAKI
SLSVQSNLHDKSIDISRVQIRQNPKFDPLFLTDIVDQYGQNKKQDFIGKVHNDLELEQQRVDEAKTFTGK
VAEDRSRYGGWLSGPKLKATGFFRTEKVNGKWSLVDPDGYLYLATGIDIIRLSNSSTLTGYDFKQAYIVQ
PPKDNVTPEDSQKLNRVSDEAIASRFIASNTRTNMFSWLPDYDAPLGKHFGYRRSAHSGPLKHGETFSFY
SANLERKYGQQAKGYMQAWEDTTVNRMLDWGFTSLGNWTDPRFYXSNKIPYFANGWIIGNYKTVSSGDDF
WAPMPDVFDPEFEKRAYATAKVIYDEVKGNPWCVGIFVDNEKSFGRSDSTESRYGIVVNTLTKNGAEVPT
KAEFTRLMQQKYVDINKLNQAWNKSITDWAEFDSGIDSSINNAEQINDYGLLLTAYADKYFSTVNKAVKH
YMPNHLYLGSRFPDWGMPREVVNASAKHVDVISFNSYKEGLTKKSWAFLQEIDMPSIIGEFHIGAKDSGL
YHPGLILASDQQDRAVMYKDYMKSVIDNPYFVGAHWFQYIDSPITGRAYDGENYNVGFVSVTDTPYPYMV
EAAKAINKTMYQQRFKEK

>WP_016709240.1
MKYKKNLAVTALVLALTGCNQQTNNVSDPVAAKATSSSSTLLHLLDDAQHTADTAVKIKTSGAQVTNNDN
ILNIAFNSEQNLYSGVTFIPDTPWDWSQFKDFNIAFELANPGQHSVQVYLDISDIDGANYTRTVNVPIGD

FHTYYAKLDGHDLATPDGDESVELNFTSGLRSNPATWQSDETQFISMWGKKNLNLKGITKISLSVQSALH
DKALQIKSIDLRTNPEFDTQFLTHIVDKFGQNAKQDFAYKVHSVDELITDKQQEAKQLIAQRPADRSRFG
GWAQGPKLEGTGYFRTAKHNGKWSLVDPDGYLYLATGLDIIRLANSTTLTGYDFDQSLFKSANESGLTPE
DSKGLNRVNKEALPSRFVASQVRKDLFEWLPSYDEPMGKHYGYRKSAHSGPLAHGETYSFYSANLERKYG
QNNSDFMQKWREVTLQRMITWGFTSLGNWTDPSYYDNQQVPYFANGWIIGDFKTVSSGNDFWGAMPDVFD
PHFAERADITVENVAKEVKNSPWAVGVFIDNEKSFGRPDSVQSHYGIVINTLGRDAATVPTKAEFSRLMK
QKYTDINQLNQVWHLNLASWEEFDKGVKVDVKNNDQLADFSIMLTAYADKYFSVVDAAMDKHLPNHLYLG
ARFPDWGMPIEVVRASAKHVDVISFNAYKEGIKPKKWEFLSEFDKPTIIGEFHVGALDSGLFHPGLIHAA
DQQDRAKMYTDYMRSVFDNPYFIGAHWFQYIDSPITGRAYDGENYNVGFITVTDRPYTEMVEAAKTVNNE
MYERRFKK

>WP_064385062.1
MKKNLAVTALLLALTGCNQQTNSVSEPVASQATNTSKTLLQLLDDDQHTANTAVQIKTSGARVTNDAGVI
NVAFDSEQNLYSGITLTPDTPWDWSQFKDFNIAFELANPGQHSVQIYLDISDIDGANYTRTVNVPIGGFN
TYYAKLDGHDLATPEGEENVELNFTSGLRSNPATWQSDETQFISMWGKKNLNLTGITKISLSVQSALHDK
ALQIKSIDLRTNPEFDTEFLTHIVDKFGQNAKQDFAYKVDSEAELIADKKQESQQLLSQRPADRSRFGGW
AQGPKLEGTGYFRTAKHKGKWSLVDPDGYLYLATGLDIIRLANSTTLTGYDFDQSLFKSANESGLTPEDS
KGLNQVNKEALASRFVASKVRKDLFEWLPSYDEPMGKHYGYRKSAHSGPLAHGETYSFYSANLERKYGQN
NNDYMQKWREVTLKRMITWGFTSLGNWTDPSYYDNQQVPYFANGWIIGDFKTVSSGNDFWGAMPDVFDPH
FTERANITVENVAKEVKNSPWAVGVFIDNEKSFGRPDSVQSHYGIVINTLGRDAATVPTKAEFSRLMKQK
YTDINELNKVWHLNLASWQEFDKGVKVDVKNNDQLADFSIMLSAYADKYFSVVNAAMDKYLPNHLYLGAR
FPDWGMPIEVVRASAKHVDVISFNAYKEGIKPKKWEFLSEFDKPTIIGEFHVGASDSGLFHPGLIHASNQ
QDRAKMYTDYMRSVIDNPYFIGAHWFQYIDSPITGRAYDGENYNVGFITVTDRPYTEMVEAAKTINNEMY
ERRFKK

>AND89921.1
MQRTKWGGVVDDDLRGSGFFRVSERNGVFWLIDPDGGRFLSKGVNTVRFDQDRIGRTERVPYAETCRAKY
GSLQSWRAAASTRLASWNFNTVGCWSDEGVASAGSQLLAITPIRELGASFRLHRRDQIFPDVFDPEFAAH
ICASAKERCTLRRNDPGLLGTFIDNELYWSTDARGPDELLTLFLNLPSHRPGRVAAISRLQAHYRDISEF
NAVWRTPARSWEELGRIGHVVAPFVRMPPGGLNDALETKANLADRAREAFSADCDAFVAVVADKYFEVCV
SAIKTADPHHLVIGSRFGWQPPRGVIAAAGRHLDVISFNCYEFDPGPVIDAYAATGKPCLISEFSFRGDD
AGLPNSKGAGPRVATQTERARAFQGYVVAALGKPNVVGYHWFEHADQPVQGRFDGEDSNFGTVTVDDRVY
DELTKTMTRVNAAAERIHAAAVPAVI

>ANB26030.1
MKIHSISMAVAVACLSLAACNTQKNQTSLPSSSSQNVIDNLYSFDVPLNGKIETEFASATVQPMSPNSGK
ALHVSFEGDVAEASVKLIPAQNWDWSMHKEINLAFEANNPEEESVQLYISVMTESGNQASHSVIIPPGST
STYYFVLNGQVLDTDLGYKRSRMEAWQSNEQMAHFRIGSIKLDLSSVEAIRLYTRGNMVSKRLTIDNLRL
RENPDYGDEYRQNTVDKFGQNAKRDFPLKVHSEAELKEKANEELTRLKASGPLPDRSRFGGWKAGPKLEA
TGYFRTHKMQGKWWLVDPDGYLFFSNGLANVRMANLTTLTGVDFKDESVRYIDPEAVTPEDSMGIVNVSD
AVRDTRYIASEVRHEMFSWLPPYDDALADHYSYRRSVHAGPLTSGETFSFYRANLERRYGESSPGAYEEK
WQEVTLDRFQDWGFTSMGNWVDPAFYPNEQVPYFANGWIIGDFKTLSSVHDVWDSMPDSFDPEFVRRAQV
TINQIAKEIQSSPWCIGIFVDNEKSWGRTEGTLEQRYGLILDALSHSIDESPAKKAFVDALKQKYSSLNS
LNEGWGTSFTSWQALNNAWQPQQPTAALEKDLSMMLEMLSEQYFKVVHDALETALPHHLYMGARMASWGM
PDETISAATRYSDVLSFNIYKEGVQPSQWRFIEDIDLPSIIGEFHIGTNTDSGLFHPGLVAAADQEDRAK
MYQQYMQSVVDHPNMVGAHWFQYVDSPISGRAFDGENYNVGFVSVTDIPYTEMVNAAREFNTTLYPARFN
QR

>ANB26027.1
MNKTAIALALSLSLLGCQKSEEAPTFTASKSDSIEASASLSKEQPLIQLTKEVIDSQVELVNAKASFTSR
GMKVTLLAKDNPNSGINIKPSEPWDLSEFDDFNLAMDIENPGPHSVQLFLNITDIDGATYTRSVAVPVGE
KATYYAKMRGHDLATPDGDVNQELNFLSGLRSNPETWESGDVQFISMWGKKNLNLKGITEISLSVQSALF
DKHIELSNIRLRPNPEMNTDFLTKIVDKYGQNATVEFPGKIHSQAELIQARETEAKQLDNKLMPDRSRFG
GYKEGPKLAATGYFRTEKIDGKWAMVDPEGYLYFATGLDIIRLSNTSTMTGYGFDDGLVDLGGDGVTPED
SKGLNRVNDEAIPSRHIVSDVRANMFNWLPSYDEPLGKWFGYRGSAHSGPVKKGETFSFYASNLERKYGE
QDPLEAWEQVTLKRMKNWGFSSLGNWTDPRFYQNNEVPYFANGWIIGDFKTVSSGNDFWSPLPDVFDPEF
ARRADVTASNVAEQVKNSPWCVGVFIDNEKSFGRSESNEARYGIVINTLTRDGKDVPTKAAFTGLMKKKY

GSISALNAAWGTKIASWGAFNAGIDSSIRNEVQLADYSEMLFHYGEKYFSVVNAALDKHMPNHMYLGARF
PSWGKPMEIVEAAAKHVDVMSYNVYKEGIHPKSWEFLQDIDMPSIIGEFHMGARDNGLFHPGLIQAATQE
DRAQMYIDYMHSVIDNPYFVGAHWFQYMDSPITGRAYDGENYNVGFVNVADTPYAPMIKAAKEVNSKMYP
RRFK

>ANB20076.1
MNKTAIALALSLSLLGCQKSEEAPTFTASKSDSIEASASLSKEQPLIQLTKEVIDSQVELVNAKASFTSR
GMKVTLLAKDNPNSGVNIKPSEPWDLSEFDDFNLAMDIENPGPHSVQLFLNITDIDGATYTRSVAVPVGE
KATYYAKMRGHDLATPDGDVNQELNFLSGLRSNPETWESGDVQFISMWGKKNLNLKGITEISLSVQSALF
DKHIELSNIRLRPNPEMNTDFLTKIVDKYGQNATVEFPGKIHSQEELIQARETEAKQLDNKLMPDRSRFG
GYKEGPKLAATGYFRTEKIDGKWAMVDPEGYLYFATGLDIIRLSNTSTMTGYGFDDGLVDLGGDGVTPED
SKGLNRVNDEAIPSRHIVSDVRANMFNWLPSYDEPLGKWFGYRGSAHSGPVKKGETFSFYASNLERKYGE
QDPLEAWEQVTLKRMKNWGFSSLGNWTDPRFYQNNEVPYFANGWIIGDFKTVSSGNDFWSPLPDVFDPEF
ARRADVTASNVAEQVKNSPWCVGVFIDNEKSFGRSESNEARYGIVINTLTRDGKDVPTKAAFTGLMKKKY
GSISALNAAWGTKIASWGAFNAGIDSSIRNEVQLADYSEMLFHYGEKYFSVVNAALDKHMPNHMYLGARF
PSWGKPMEIVEAAAKHVDVMSYNVYKEGIHPKSWEFLQDIDMPSIIGEFHMGARDNGLFHPGLIQAATQE
DRAQMYIDYMHSVIDNPYFVGAHWFQYMDSPITGRAYDGENYNVGFVNVADTPYAPMIKAAKEVNSKMYP
RRFK

>ANB20073.1
MKIHSISMAVAVACLSLAACNTQKNQTSLPSSSSQNVIDNLYSFDVPLNGKIETEFASATVQPMSPNSGK
ALHVSFEGDVAEASVKLIPAQNWDWSMHKEINLAFEATNPEEESVQLYISVMTESGNQASHSVIIPPGST
STYYFVLNGQVLDTDLGYKRSRMEAWQSNEQMAHFRIGSIKLDLSSVEAIRLYTRGNMVSKRLTIDNLRL
RENPDYGDEYRQNTVDKFGQNAKRDFPLKVHSEAELKEKANEELTRLNASGPLPDRSRFGGWKAGPKLEA
TGYFRTHKMQGKWWLVDPDGYLFFSNGLANVRMANLTTLTGVDFKDESVRYIDPEAVTPEDSMGIVNVSD
AVRDTRYIASEVRHEMFSWLPPYDDALADHYSYRRSVHAGPLTSGETFSFYRANLERRYGESSPGAYEEK
WQEVTLDRFQDWGFTSMGNWVDPAFYPNEQVPYFANGWIIGDFKTLSSVHDVWDSMPDSFDPEFVRRAQV
TINQIAKEIQSSPWCIGIFVDNEKSWGRTEGTLEQRYGLILDALSHSIDESPAKKAFVDALKQKYSSLNS
LNEGWGTSFTSWQALNNAWQPQQPTAALEKDLSMMLEMLSEQYFKVVHDALETALPHHLYMGARMASWGM
PDETISAATRYSDVLSFNIYKEGVQPSQWRFIEDIDLPSIIGEFHIGTNTDSGLFHPGLVAAADQEDRAK
MYQQYMQSVVDHPNMVGAHWFQYVDSPISGRAFDGENYNVGFVSVTDIPYTEMVNAAREFNTTLYPARFN
QR

>WP_062085905.1
MNKTAIALALSLSLLGCQKSEEAPTFTASKSDSIEASASLSKEQPLIQLTKEVIDSQVELVNAKASFTSR
GMKVTLLAKDNPNSGINIKPSEPWDLSEFDDFNLAMDIENPGPHSVQLFLNITDIDGATYTRSVAVPVGE
KATYYAKMRGHDLATPDGDVNQELNFLSGLRSNPETWESGDVQFISMWGKKNLNLKGITEISLSVQSALF
DKHIELSNIRLRPNPEMNTDFLTKIVDKYGQNATVEFPGKIHSQAELIQARETEAKQLDNKLMPDRSRFG
GYKEGPKLAATGYFRTEKIDGKWAMVDPEGYLYFATGLDIIRLSNTSTMTGYGFDDGLVDLGGDGVTPED
SKGLNRVNDEAIPSRHIVSDVRANMFNWLPSYDEPLGKWFGYRGSAHSGPVKKGETFSFYASNLERKYGE
QDPLEAWEQVTLKRMKNWGFSSLGNWTDPRFYQNNEVPYFANGWIIGDFKTVSSGNDFWSPLPDVFDPEF
ARRADVTASNVAEQVKNSPWCVGVFIDNEKSFGRSESNEARYGIVINTLTRDGKDVPTKAAFTGLMKKKY
GSISALNAAWGTKIASWGAFNAGIDSSIRNEVQLADYSEMLFHYGEKYFSVVNAALDKHMPNHMYLGARF
PSWGKPMEIVEAAAKHVDVMSYNVYKEGIHPKSWEFLQDIDMPSIIGEFHMGARDNGLFHPGLIQAATQE
DRAQMYIDYMHSVIDNPYFVGAHWFQYMDSPITGRAYDGENYNVGFVNVADTPYAPMIKAAKEVNSKMYP
RRFK

>AMX04183.1
MLLWDFENGKQPKELKLQNATAEVIQRGSGKALSIRLLSEDNHTSAFSFEPEGTWDWSAMGNFAFALDIS
NAESSSVHLYVKATDNEGRTHSRSFVVPEQSSATYFFEMKGPDLAVDTGIRSNPPSWMSEYQDMIYRWGD
KSLNVSEIDSIEFSVAGVLENKYVVVDNVRLIQPESLDEMYLVNLVDEFGQNNKRDFTNKVHSLEELREA
SERELAQLRKTPLEGRSRFGGWALGPRLKGTGYFRTEKVDGKWSLVDPDGYLFFSTGIANVRLANTSTIT
GYDFDHALIPARKPGDLTPEDSLGLNRVPDVAVPTRYVSSSLRADMFTWLPAYDEPLGKNFGYRREVHTG
AIERGETFSFYRANLQRKYGIDDDKKLMEKWREVTVDRMLSWGFTSFGNWIDPGYYQMDRFPYFANGWII
GNFKTVSSGNDYWSPLPDPFDPLFKERAYITAEQIAKEVKGSPWCVGVFVDNEKSWGQEGSVETQYGLVI
NTLTRDAKESPTKAQFVRLMREKYGDIQKLNKSWGVSIDSWNDFASGISLNIFNDSVIKDLSIMLQHYAD
QYFRIVREAVKHYMPNHMYMGARFADWGMTPEIRAAAANYADVVSYNYYKEGVSDNFWHFLKELDRPSII

GEFHNGALDSGLLNPGLIHASSQADRGKKYAEYMNSVIDNPYLVGAHWFQYIDSPLTGRAYDGENYNVGF
VSVTDIPYSPLVESAKAVNKNLYQRRFGSM

>AMX02903.1
MVCPSLTGRIALAFLSLVLTACSSDGDRADTKQPPPSEDKVQQDSQPDAFTFGAVSDAEPGSQQVSNAIT
ITGINTSTDISVNGGEFSINDGPFQNSTGTVNSGDSVVLRLIASTDYGATVTATLTVGGVSAEFRVTTKA
ESTNNEDTVKVDVNLNMRHTVGGESVFDRRKFINIHASNTEQDWVGNNSQSAGAPNEDPDLMTNFLEGYD
VYLGRDTGGMKWQLSLLPEDPSRPGFIDETAAKSNGGGARWNYTTGTSTGAQLARYHEHRATDMVVGGQQ
HPYWPNGDDTGMGWSFSTADSEEEPFGTAVGHYMANFLYEYFNRGAGDTYGQPKPVFLEVMNEPLYDLVD
FPSDKDKGTTPEEVFKFHNAVADAVRAFRDPWDQAVHDNVLIGGYTVAFPDFEKNDFNRWEERDKLFIDI
AGEKMDFLSLHFYDFPDIEGKRKLRRGSNLEATFDMLEQYTLMATGERMPFLISEFGATVHSMMNQPWSP
ERDGYKLRAFNGLMMNILERPDQILKSIPFITVKAEWGRQSETVPYTNRLMRQKKEADTESGDSWVYTEL
VKFYQLWAEVKGTRVDSWASDLDVQVNAYVDGATAYIVLNNLEEETSILDLATLGTGTNPLQEVTVKELS
YNDDGVPVLSETTLEQLPEKITLKPEATLVLQLNYKENITLDEENSETKYYADKYKQPIEAKRKLQFAIS
NVEVSDEGEAILRLGIGRDHGKSLTPSVTINGHKVSVPEDYQGYDQYYDGKGRDQFFGVIEIPVAIEYLT
KDNAVEIEFPDDGGYVSTTTLQVFSTSSAINRGVR

>AMX02879.1
MSLHTFAPLAVSRSSRSFVFAALFSLTACGGASDPSVPKVQAASTSGQLSEDLVLEDFDKTAIPATVKVN
NGAVSLVDSARGDKALLVKLDLKENNSAALVIAPEEPWDWSEFSGFNLAFDVANKGTESVQIDVTMTDKN
GDSYTRGMVVPADGVWRTYYAKLHGHDQQDPDSAAKNEFNFSSGLRSNPPTWQSDDVMLHSFWGKKLLDL
SGITKISFGSDGSLSNRQYTIDNIRLRANPEMDKNFLTGLLDKYGQNAKVDYEGKIHSDEELRAVVAEEL
AGLSGKLNEGRSKYSGWKDGPRFKSTGYFRTQKVNGKWALIDPEGYLYFSTGIDIIRLSNSSTITGYDYD
QALIPARSDDEVIAEDDQPLNRVNEKAWATRHLVSETRAKMFTWLPDYNDPLGNHYGYRRETQSGPLKHG
ETFSFYSANLERRYGESYPESYLDTWRKVTVDRMLDWGFTSLGNWADASFYQEERIPFVAFADIIGDFGT
LSSGFDFWHPVPDPFDPRFYQRALVAAESVNQQINGSPWCMGVFFDNEQSFGRLESDELHYGIVINALSR
DAADTPAKSAFTEILKKKYGSIEALNKAWNKQVASWEAFAAGMDSSISTDAQLEDYASLLFAYGDQYFGT
INRAMKSIMPNHLYLGSRLPSWGMPDEIVKAAAKNVDIISYNLYEEGLVPSKWEFLAEIDKPSLIGEFSF
GADDQGHFHPGIVISADQKDRGRMFRNYMYSFIDNPYFVGVHMFQYMDSPITGRAYDGENYANGFVSVTD
VPYIELVKAAKEVNGSLYKRRYGDVQLRARSASERGSD

>ALM91910.1
MNKTAIALALSLSLLGCQKSEEAPTFTASKSDSIEASASLSKEQPLIQLTKEVIDSQVELVNAKASFTSR
GMKVTLLAKDNPNSGVNIKPSEPWDLSEFDDFNLAMDIENPGPHSVQLFLNITDIDGATYTRSVAVPVGE
KATYYAKMRGHDLATPDGDVNQELNFLSGLRSNPETWESGDVQFISMWGKKNLNLKGITEISLSVQSALF
DKHIELSNIRLRPNPEMNTDFLTKIVDKYGQNATVEFPGKIHSQEELIQARETEAKQLDNKLMPDRSRFG
GYKEGPKLAATGYFRTEKIDGKWAMVDPEGYLYFATGLDIIRLSNTSTMTGYGFDDGLVDLGGDGVTPED
SKGLNRVNDEAIPSRHIVSDVRANMFNWLPSYDEPLGKWFGYRGSAHSGPVKKGETFSFYASNLERKYGE
QDPLEAWEQVTLKRMKNWGFSSLGNWTDPRFYQNNEVPYFANGWIIGDFKTVSSGNDFWSPLPDVFDPEF
ARRADVTASNVAEQVKNSPWCVGVFIDNEKSFGRSESNEARYGIVINTLTRDGKDVPTKAAFTGLMKKKY
GSISALNAAWGTKIASWGAFNAGIDSSIRNEVQLADYSEMLFHYGEKYFSVVNAALDKHMPNHMYLGARF
PSWGKPMEIVEAAAKHVDVMSYNVYKEGIHPKSWEFLQDIDMPSIIGEFHMGARDNGLFHPGLIQAATQE
DRAQMYIDYMHSVIDNPYFVGAHWFQYMDSPITGRAYDGENYNVGFVNVADTPYAPMIKAAKEVNSKMYP
RRFK

>ALM91905.1
MKIHSISMAVAVACLSLAACNTQKNQTSLPSSSSQNVIDNLYSFDVPLNGKIETEFASATVQPMSPNSGK
ALHVSFEGDVAEASVKLIPAQNWDWSMHKEINLAFEATNPEEESVQLYISVMTESGNQASHSVIIPPGST
STYYFVLNGQVLDTDLGYKRSRMEAWQSNEQMAHFRIGSIKLDLSSVEAIRLYTRGNMVSKRLTIDNLRL
RENPDYGDEYRQNTVDKFGQNAKRDFPLKVHSEAELKEKANEELTRLNASGPLPDRSRFGGWKAGPKLEA
TGYFRTHKMQGKWWLVDPDGYLFFSNGLANVRMANLTTLTGVDFKDESVRYIDPEAVTPEDSMGIVNVSD
AVRDTRYIASEVRHEMFSWLPPYDDALADHYSYRRSVHAGPLTSGETFSFYRANLERRYGESSPGAYEEK
WQEVTLDRFQDWGFTSMGNWVDPAFYPNEQVPYFANGWIIGDFKTLSSVHDVWDSMPDSFDPEFVRRAQV
TINQIAKEIQSSPWCIGIFVDNEKSWGRTEGTLEQRYGLILDALSHSIDESPAKKAFVDALKQKYSSLNS
LNEGWGTSFTSWQALNNAWQPQQPTAALEKDLSMMLEMLSEQYFKVVHDALETALPHHLYMGARMASWGM
PDETISAATRYSDVLSFNIYKEGVQPSQWRFIEDIDLPSIIGEFHIGTNTDSGLFHPGLVAAADQEDRAK
MYQQYMQSVVDHPNMVGAHWFQYVDSPISGRAFDGENYNVGFVSVTDIPYTEMVNAAREFNTTLYPARFN

QR

>WP_057794026.1
MNKTAIALALSLSLLGCQKSEEAPTFTASKSDSIEASASLSKEQPLIQLTKEVIDSQVELVNAKASFTSR
GMKVTLLAKDNPNSGVNIKPSEPWDLSEFDDFNLAMDIENPGPHSVQLFLNITDIDGATYTRSVAVPVGE
KATYYAKMRGHDLATPDGDVNQELNFLSGLRSNPETWESGDVQFISMWGKKNLNLKGITEISLSVQSALF
DKHIELSNIRLRPNPEMNTDFLTKIVDKYGQNATVEFPGKIHSQEELIQARETEAKQLDNKLMPDRSRFG
GYKEGPKLAATGYFRTEKIDGKWAMVDPEGYLYFATGLDIIRLSNTSTMTGYGFDDGLVDLGGDGVTPED
SKGLNRVNDEAIPSRHIVSDVRANMFNWLPSYDEPLGKWFGYRGSAHSGPVKKGETFSFYASNLERKYGE
QDPLEAWEQVTLKRMKNWGFSSLGNWTDPRFYQNNEVPYFANGWIIGDFKTVSSGNDFWSPLPDVFDPEF
ARRADVTASNVAEQVKNSPWCVGVFIDNEKSFGRSESNEARYGIVINTLTRDGKDVPTKAAFTGLMKKKY
GSISALNAAWGTKIASWGAFNAGIDSSIRNEVQLADYSEMLFHYGEKYFSVVNAALDKHMPNHMYLGARF
PSWGKPMEIVEAAAKHVDVMSYNVYKEGIHPKSWEFLQDIDMPSIIGEFHMGARDNGLFHPGLIQAATQE
DRAQMYIDYMHSVIDNPYFVGAHWFQYMDSPITGRAYDGENYNVGFVNVADTPYAPMIKAAKEVNSKMYP
RRFK

>WP_062065002.1
MCSSYKLDRPITLFLKTSVLSVAALFLIACQDKAVDKDPAGTPAQAQTAAAEQVLFDFEGDQLPAEISFF
NAQGSLVKSSASDTSASQALKVKYNSVDHEYTSLVIQPKSDTWNWSDIGDASLAFDIANDGEHSVQLFLD
VSDAKGNNFTRSVSVPVGKSRVYYSKLSGHDMVSANPDSKVELNAASGLRGNPPTWSGDDVQFIWMWGVM
NLDLSAVKRISLSVQYALHDKEITLDNIRVIKSPAMNKDFLVNLVDKFGQPAKVDFAGKIHSEAELQAAT
QSELKELNNGAPLADRSTFGGWKNGPKQPATGYFYPKKVDGKWWLVDPEGYLYFATGLDIIRLANAYTMT
GYDYDASTIEQRSADDLTPEDSKGKILISEEAQKTRHLVSKTRADMFEWLPKHTDPLGNHYDYNRDAHSG
PLLKGEAFSFYSANLERKYGETEPDSYLRQWEKVTVDRMLNWGFTSLGNWTDPKFYNNQRIPYFANGWII
GNFKTVSSGNDFWGGLPDPFDPVFKERALATAKAIAAETKNSPWCVGVFIDNEKSWGRSESKESEYGIVL
NTLTRDGADSPTKNKFTQLMKEKYVDIAALNTAWGTSVGSWDAFQKGVKTGINNDVQLQDFSLLFTQYAE
EYFKIVEGALTQYMPNHLYLGVRFADWGMPKDVVKAAAKYADVVSYNFYKEGLTKNKWTFLAELDKPSII
GEFHIGTTESGLFHPGLVHAANQEDRAKMYKEYMETVVDNPYFIGAHWFQYMDSPVTGRSYDGENYNVGF
VSVTDTPYAPMVKAAKELHGEMYTRRAKK

>WP_062064965.1
MKFTQLISIATYTLILGGCGGGGGGGGTSTPPASSKASVSPTKSSTAVSSLVASSSTSLSSTPNSSVAST
DSRPKISFNRDIKHIVGGIDSFDRRKFITIHSSNTEADWFGSNAQSLGAPNASPDLITEVMDGYDVYFGR
DTGGITWQLGELNQDPARPGFVSESHAQTKGGDARWVYSNNANSTKIRQFENRLTDMIIGAQQHPFWPDG
KTTRKGWALSQTDTPSEPFGTATGHYMGQYLAKYFNKGAGDLYGQPKPLYVEVMNEPLYDLVDASASPVP
VEKVFQFHNTVAAEIRKTNSDVLIGGYTVAFPDYDKNDFQQWLNRDKVFIDLAGANMDFYSIHLYDFPAH
NNREKYRRGSNVEATLDLLEQYDTIKFGKIKPLVISEYGASIHSMFSDPWTPQRDGLRLIAFNGLLMSFL
ERPNNIAKTIPFLPIKAEWGRVNGIPYNDRLMRQRKEAPGETGDEWVFTDMIKFYQLWSDVKGTRIDITS
SDPDLVVNAYADNKRVYLIINNLEFNAQEFTLNDYSLNNPAFANATIKHLHLDSQNAPVLSETVSDKFPD
SLSIGASGTMIISMNYESAVVIDQTKVEQKTYATNYLQAIKTNSPLNFAINGITPAAFGEATLRLGIGRD
HGKSLQPKVIINGTEVTVPKDYRGYDQYHQGKGRENFFGVIEIPVPYSTLKANNQIEVTFADDGGHISSA
ALRAFHYSRNFTH

>WP_062064950.1
MKTNQLRLIKQMLAGTLAGLSLGCVANQNSPLGSIENFDSFSLNKAVENNVKASLIEGQEKKYGKALKLV
FEPREESTIELPTPAQGYWNWDYAGDLNLALDVTNPGSQSFQFWLTLMDAKGRKQERSAVIDAGQSARFY
APLTGRVANAQTGMRETPPAWQTSEEKLAWRSGDRDFDFTQVTKIIFKAYAQFETNTLIVDNLQLRVNPA
QDPEYLVGIVDKFGQAAKKNYPTKIHSEKELKAAADAELAALANAKQPEDRSRFGGWSKGPKLEGTGYFR
TAKVDGRWWMVDPEGYLFFSSAIANVRMANLETITGYDFNDASVRKIDPEELTPEDSRDIIPVGKKSLNS
RFLASPLRREMFEWLPPYDHELGEHYGYRRTVHQGVLEQGEVYSFYKANLERRYGQSSPNSYIKTWRDVT
LDRMVDWGFTSFGNWIDPMFYDNQRMPFFANGWIIGDFKVIYSGQDYWSPLPDVYDPEFKRRAHLTIKQI
GREVKNTPWCVGVFVDNEKGWGSMKNDRAHFGAVYYALSRSADESPAKKQFTQLLTKKYGDIAALNQAWG
TKIDSWSSFATGITMDKLNDASRADFSMLYADYAETYFRIVSSEIKDALPNHMYMGVRIAAEWGMPVEVV
AAAKKYSDVLSFNNYREGMHPDTWAFLKDLDFPTIIGEYHIGSTSDTDFYHPGLVIAANQTDRAKMYENY
MNSVIDNPYMVGAHWFQYIDDPVTGRAYDGENYNVGWVSNTDIPYQPMVDAAKRVNKSLYKRRSKIAPIQ

>KYL34743.1

MKKNLAVTALLLALTGCNQQTNSVSEPVASQATNTSKTLLQLLDDDQHTANTAVQIKTSGARVTNDAGVI
NVAFDSEQNLYSGITLTPDTPWDWSQFKDFNIAFELANPGQHSVQIYLDISDIDGANYTRTVNVPIGGFN
TYYAKLDGHDLATPEGEENVELNFTSGLRSNPATWQSDETQFISMWGKKNLNLTGITKISLSVQSALHDK
ALQIKSIDLRTNPEFDTEFLTHIVDKFGQNAKQDFAYKVDSEAELIADKKQESQQLLSQRPADRSRFGGW
AQGPKLEGTGYFRTAKHKGKWSLVDPDGYLYLATGLDIIRLANSTTLTGYDFDQSLFKSANESGLTPEDS
KGLNQVNKEALASRFVASKVRKDLFEWLPSYDEPMGKHYGYRKSAHSGPLAHGETYSFYSANLERKYGQN
NNDYMQKWREVTLKRMITWGFTSLGNWTDPSYYDNQQVPYFANGWIIGDFKTVSSGNDFWGAMPDVFDPH
FTERANITVENVAKEVKNSPWAVGVFIDNEKSFGRPDSVQSHYGIVINTLGRDAATVPTKAEFSRLMKQK
YTDINELNKVWHLNLASWQEFDKGVKVDVKNNDQLADFSIMLSAYADKYFSVVNAAMDKYLPNHLYLGAR
FPDWGMPIEVVRASAKHVDVISFNAYKEGIKPKKWEFLSEFDKPTIIGEFHVGASDSGLFHPGLIHASNQ
QDRAKMYTDYMRSVIDNPYFIGAHWFQYIDSPITGRAYDGENYNVGFITVTDRPYTEMVEAAKTINNEMY
ERRFKK

>KXI28840.1
MNIPPAKGYLLCSCLTLLLCTVLGCNQQAGTNMRQNSEPFLSTVDSFAKQQSLLTFYDPSDLQHQHELIL
SGASSEVISTNGDAKLAVEFSPLHNISELRIQPAQPWNLSPFKQANLALDVENTGATSIQMYLSIENNQG
QSRRYSVSLASDFKGTVYVPVSGKEADTDSGLWGDAPPWTTEDKMLVWRSWRSAGVELETIAALNFFTIG
ILEPKSVQIDNIRLRQNPPTDPNWLVNIIDQYGQNARLDTPLKVHSDEQLQQLAQQELRQLQESSGMPHR
SKFGGYTNAPRREATGYFRTEKVNGKWWMVDPEGYLFFSHGPANVRMANMSTITGIDYRDPSVRVRHVDE
LTPEDSMGIVAVSDQVRATRYVSSELRHNMFEWLPEYSDPLADHYSYRRSTHKGPVPHGETYSFYQANLE
RRYGETEAKSYLKKWQEVTMQRMHDWGFTSFGNWVDPAFYAADKVPYFANGWIIGDYQTLSGETNHWGLM
PDPFDPVFAQRAKSTIDAIAKDVKASPWCVGIFIDNEKSWGEREGSVNARYGVILDALSKNSQTSPAKAA
FSAYLKQKYSTIEQLNSAWNSDLKDWQALDNGVVMTDYSAQHVQDLSTMLAMLGEQYFQVVHGTLAEVLP
KHLYMGARMANWGMPDEIITASLKYSDVLSFNIYEEGMQEHFWQFLEKADLPVVIGEFHIGTATDSGMFN
PGIVHAANQSDRARMYKDYMQSVLSKPYMVGAHWFQYIDEPISGRAFDGENANIGFVTAADIPYPELIQA
VKEVTSTMYQNRVAEQGP

>KXI28825.1
MKNAPSLSLLIMLSGLCACQPAENVQSPAKQSIAQASNTIMTLAALDGSMPNNIKLSNAKGQITDQGLEI
DFQSKQHPYASVTLVPDEPYNWSEFSDFNLAFDIANKGKHSVQLNLDVSDIDGNNYTRSVNVAVSGKHTY
YGKMSGHDLATPKGDENIELNFNSGLRSNPATWEGDDIQFISMWGKKNLNLNGITKISLSVQNVLHDKSI
TLSNIRLRKNQAKNPEFLTAIVDQYGQNAKLDFPTKIHSDEQLQLITANELATFKDTTRPDRSKFNGWKQ
GPKLNATGYYRSEKVDGKWWLVDPEGYLYFATGLDIIRLSNSTTITGYDFDQSHIAQKAANDLTPEDSQG
LNKVSDKAVTTRFVSSDLRKNMFAWLPSYEEPLGKHFGYRRSVHSGPVKHGETFSFYSANLERKYGEQFM
DKWRSVTIQRMLEWGFTSFGNWTDPSFYQNGQMPYFANGWITGDYKTVSSGNDFWAPMPDVFDPEFAVRA
NMTASVIAQEVKDSPWCVGVFIDNEKSFGRPDSTTAHYGIVLHTLRRDGAEVPTKAEFTRLMQQKYGDIA
ALNKVWDKEIADWASFNQGINSDINNEQQIADYGILLGAYAEQYFKTVHDAVAKHLPNHMYLGSRFPDWG
MPIEVVKAAAKYADVVSYNSYKEGLRPDKWAFLKELDKPSIIGEFHMGASDSGLFHPGLIHAANQEDRAQ
MFADYMQSVIDNPYFVGAHWFQYMDSPITGRAYDGENYNVGFVNVTDTPYPEMVEAAKDVNSKIYQARFE
Q

>KXI28823.1
MPTLITTHQLNRLLILSLSFAVIACGGSGGSATPPSTPPPVTTPVTDTTPNSFSFSFSSIDHVPLDTLVS
SDAISISGINAATAISISGGEFKVNEGEFTASSTTLNNNDKVTVRITSAADYATSSELNVTIGGVVGSFK
VTTVPAPPLGKVVDVNLDIKHSVGGIERFDREKFITIHANHTENDWYAVGENASADLITEFAEGYDVYFG
RDAGGIGWNLNNLPQDPAKAGFVDPAATTERANGVRWNYTNLTTPRAITQRNLEYRNKNLIVSGQQHPYW
PDGQLTGNLSAASWAFSQIDSANEPFGTATGDYMARYVSQFFKQGETDPYGQTKPAFVEVMNEPLYDLYD
AATNPVELSQVFAFHKTVANTIRNLEINGEKPNQNLKVGGYTAAFPDFDTDNFQEWEERDKLFIDMAGAD
MDFISLHLYDMPRFRNTVQLRKGSNMEATFDLLEHYTNLSFGAPKPFIISEYGSQVHTMLNSPWSPERDW
LFINSMNAQLMNFLERPNLIEMTIPFIVVKAEWGRISDTVPYSHRLMRQQKEAAGETGDLWVYTDLVKFY
QLWANVNGQRAESKANDLDLQVDAYVDGNKAYIIVNSLEMEATTFSLNTYGLTDNDINQLIKRELRQENG
VAVLDEQTLTSIPNTMEIGAEATIIFEITYQNAIAMAHSATETKYYASTYKQAIVAATTHSFTIADVILT
DEGEAVLRLGLGRDHGLSLLPTVTFNDVTLSVPEDFRGYDQFYNGNGRENFFGVIEIPVPLSALQATNTI
NVTFDDAGGFISSLSLQVLNSDKALKRAE

>KXI28812.1
MTFNKMFKKSAIALLLSLGACSADKQANLANTPLAKDDNVLQLLENFDGEQPALAFSLNGVESQIKTHVG

STNNALALSFNPQEQQAKISFKPAQAWDWSSYKEINFAFDASNPGTESIQLYISIANEAGEVSHQSINIA
AGEQGTYYLLLDGEALDTDMGFKKSGMPAWQSTDEMAFFRYGSPKIDLHKVSEIGLYIKGNLSEKSLELD
NLRLRANPSYDISYRQAYVDRFGQNDKMEFPIKIHSEAELKQEADKELAQLNQSGLMPDRSRFGGWKDGP
RSKATGYFRTEKVNGKWWMVDPDGYLFFSHGLANVRMANLTTLTGVDFKDDSVRYIDPEAVTPEDSMGIV
QVSDEVRKSRYIASDVRHDMFTWLPDYKDELAEHYSYRRSVLFGPVSSGETYSFYRANLERRYGQQAPES
YVKKWEEVTLARFQDWGFTSMGNWVDPAFYTNEKVPYFANGWIIGDYKTLASKHDVWAPMPDAFDPEFVR
RAQITIDTIANEVHNSPWCVGVFVDNEKSWGLREGSVEHRYGLILDAMAKNAQQSPAKAAFTKQLQSKYQ
TIAALNQAWQTQFASWQTLANGVSLSDFPQAMISDLSHMLEMLSEQYFKVVHDALAKAMPQHLYMGARMA
NWGMPEETIKASVKYSDVLSFNIYEEGIQPKAWAFLQDIDLPTVIGEYHIGASRETGLYHPGLVQADGQA
DRAQMYLDYMQSVLASPNMVGAHWFQYVDSPISGRAFDGENYNVGFVSTTDIPYPEMVEAAKKFNATVYP
RRYNDNK

>AMJ93382.1
MKIHSISMAVAVACLSLAACNTQKNQTSLPSSSFQSVIDNLYSFDVPLNGKIETEFASATVQPMSPNSGK
ALHVSFEGDVAEASVKLIPAQNWDWSMHKEINLAFEATNPEEESVQLYISVITESGNQASHSVIIPPGST
STYYFVLNGRVLDTDLGYKRSRMEAWQSNEQMAHFRIGSIKLDLSSVEAIRLYTRGNMVSKRLTIDNLRL
RENPDYGDEYRQNIVDKFGQNAKRDFPLKVHSEAELKEKANEELTRLNASGPLPDRSRFGGWKAGPKLEA
TGYFRTHKMQGKWWLVDPDGYLFFSNGLANVRMANLTTLTGVDFKDESVRYIDPEAVTPEDSMGIVNVSD
AVRDTRYIASEVRHEMFSWLPPYDDALADHYSYRRSVHAGPLTSGETFSFYRANLERRYGELSPGAYEEK
WQEVTLDRFQDWGFTSMGNWVDPAFYPNEQVPYFANGWIIGDFKTLSSVHDVWDSMPDSFDPEFVRRAQV
TINQIAKEIQSSPWCIGIFVDNEKSWGRTEGTLEQRYGLILDALSHSIDESPAKKAFVDALKQKYSSLNS
LNEGWGTSFTSWQALNNAWQPQQPTAALEKDLSMMLEMLSEQYFKVVHDALETALPHHLYMGARMASWGM
PDETISAATRYSDVLSFNIYKEGVQPSQWRFIEDIDLPSIIGEFHIGTNTDSGLFHPGLVAAADQEDRAK
MYQQYMQSVVDHPNMVGAHWFQYVDSPISGRAFDGENYNVGFVSVTDIPYTEMVNAAREFNTTLYPARFN
QR

>AMJ93379.1
MNKTAIALALSLSLLGCQKSEEAPTFTASKSDSIEASASLSKEQPLIQLTKEVIDSQVELVNAKASFTSR
GMKVTLLAKDNPNSGINIKPSEPWDLSEFDDFNLAMDIENPGPHSVQLFLNITDIDGATYTRSVAVPVGE
KATYYAKMRGHDLATPDGDVNQELNFLSGLRSNPETWESGDVQFISMWGKKNLNLKGITEISLSVQSALF
DKHIELSNIRLRPNPEMNTDFLTKIVDKYGQNATVEFPGKIHSQAELIQARETEAKQLDNKLMPDRSRFG
GYKEGPKLAATGYFRTEKIDGKWAMVDPEGYLYFATGLDIIRLSNTSTMTGYGFDDGLVDLGGDGVTPED
SKGLNRVNDEAIPSRHIVSDVRANMFNWLPSYDEPLGKWFGYRGSAHSGPVKKGETFSFYASNLERKYGE
QDPLEAWEQVTLKRMKNWGFSSLGNWTDPRFYQNNEVPYFANGWIIGDFKTVSSGNDFWSPLPDVFDPEF
ARRADVTASNVAEQVKNSPWCVGVFIDNEKSFGRSESNEARYGIVINTLTRDGKDVPTKAAFTGLMKKKY
GSISALNAAWGTKIASWGAFNAGIDSSIRNEVQLADYSEMLFHYGEKYFSVVNAALDKHMPNHMYLGARF
PSWGKPMEIVEAAAKHVDVMSYNVYKEGIHPKSWEFLQDIDMPSIIGEFHMGARDNGLFHPGLIQAATQE
DRAQMYIDYMHSVIDNPYFVGAHWFQYMDSPITGRAYDGENYNVGFVNVADTPYAPMIKAAKEVNSKMYP
RRFK

>AMJ89565.1
MKIHSISMAVAVACLSLAACNTQKNQTSLPSSSSQNVIDNLYSFDVPLNGKIETEFASATVQPMSPNSGK
ALHVSFEGDVAEASVKLIPAQNWDWSMHKEINLAFEATNPEEESVQLYISVMTESGNQASHSVIIPPGST
STYYFVLNGQVLDTDLGYKRSRMEAWQSNEQMAHFRIGSIKLDLSSVEAIRLYTRGNMVSKRLTIDNLRL
RENPDYGDEYRQNTVDKFGQNAKRDFPLKVHSEAELKEKANEELTRLNASGPLPDRSRFGGWKAGPKLEA
TGYFRTHKMQGKWWLVDPDGYLFFSNGLANVRMANLTTLTGVDFKDESVRYIDPEAVTPEDSMGIVNVSD
AVRDTRYIASEVRHEMFSWLPPYDDALADHYSYRRSVHAGPLTSGETFSFYRANLERRYGESSPGAYEEK
WQEVTLDRFQDWGFTSMGNWVDPAFYPNEQVPYFANGWIIGDFKTLSSVHDVWDSMPDSFDPEFVRRAQV
TINQIAKEIQSSPWCIGIFVDNEKSWGRTEGTLEQRYGLILDALSHSIDESPAKKAFVDALKQKYSSLNS
LNEGWGTSFTSWQALNNAWQPQQPTAALEKDLSMMLEMLSEQYFKVVHDALETALPHHLYMGARMASWGM
PDETISAATRYSDVLSFNIYKEGVQPSQWRFIEDIDLPSIIGEFHIGTNTDSGLFHPGLVAAADQEDRAK
MYQQYMQSVVDHPNMVGAHWFQYVDSPISGRAFDGENYNVGFVSVTDIPYTEMVNAAREFNTTLYPARFN
QR

>AMJ89562.1
MNKTAIALALSLSLLGCQKSEEAPTFTASKSDSIEASASLSKEQPLIQLTKEVIDSQVELVNAKASFTSR
GMKVTLLAKDNPNSGVNIKPSEPWDLSEFDDFNLAMDIENPGPHSVQLFLNITDIDGATYTRSVAVPVGE

KATYYAKMRGHDLATPDGDVNQELNFLSGLRSNPETWESGDVQFISMWGKKNLNLKGITEISLSVQSALF
DKHIELSNIRLRPNPEMNTDFLTKIVDKYGQNATVEFPGKIHSQEELIQARETEAKQLDNKLMPDRSRFG
GYKEGPKLAATGYFRTEKIDGKWAMVDPEGYLYFATGLDIIRLSNTSTMTGYGFDDGLVDLGGDGVTPED
SKGLNRVNDEAIPSRHIVSDVRANMFNWLPSYDEPLGKWFGYRGSAHSGPVKKGETFSFYASNLERKYGE
QDPLEAWEQVTLKRMKNWGFSSLGNWTDPRFYQNNEVPYFANGWIIGDFKTVSSGNDFWSPLPDVFDPEF
ARRADVTASNVAEQVKNSPWCVGVFIDNEKSFGRSESNEARYGIVINTLTRDGKDVPTKAAFTGLMKKKY
GSISALNAAWGTKIASWGAFNAGIDSSIRNEVQLADYSEMLFHYGEKYFSVVNAALDKHMPNHMYLGARF
PSWGKPMEIVEAAAKHVDVMSYNVYKEGIHPKSWEFLQDIDMPSIIGEFHMGARDNGLFHPGLIQAATQE
DRAQMYIDYMHSVIDNPYFVGAHWFQYMDSPITGRAYDGENYNVGFVNVADTPYAPMIKAAKEVNSKMYP
RRFK

>AMJ85705.1
MKIHSISMAVAVACLSLAACNTQKNQTSLPSSSSQNVIDNLYSFDVPLNGKIETEFASATVQPMSPNSGK
ALHVSFEGDVAEASVKLIPAQNWDWSMHKEINLAFEATNPEEESVQLYISVMTESGNQASHSVIIPPGST
STYYFVLNGQVLDTDLGYKRSRMEAWQSNEQMAHFRIGSIKLDLSSVEAIRLYTRGNMVSKRLTIDNLRL
RENPDYGDEYRQNTVDKFGQNAKRDFPLKVHSEAELKEKANEELTRLNASGPLPDRSRFGGWKAGPKLEA
TGYFRTHKMQGKWWLVDPDGYLFFSNGLANVRMANLTTLTGVDFKDESVRYIDPEAVTPEDSMGIVNVSD
AVRDTRYIASEVRHEMFSWLPPYDDALADHYSYRRSVHAGPLTSGETFSFYRANLERRYGESSPGAYEEK
WQEVTLDRFQDWGFTSMGNWVDPAFYPNEQVPYFANGWIIGDFKTLSSVHDVWDSMPDSFDPEFVRRAQV
TINQIAKEIQSSPWCIGIFVDNEKSWGRTEGTLEQRYGLILDALSHSIDESPAKKAFVDALKQKYSSLNS
LNEGWGTSFTSWQALNNAWQPQQPTAALEKDLSMMLEMLSEQYFKVVHDALETALPHHLYMGARMASWGM
PDETISAATRYSDVLSFNIYKEGVQPSQWRFIEDIDLPSIIGEFHIGTNTDSGLFHPGLVAAADQEDRAK
MYQQYMQSVVDHPNMVGAHWFQYVDSPISGRAFDGENYNVGFVSVTDIPYTEMVNAAREFNTTLYPARFN
QR

>AMJ85702.1
MNKTAIALALSLSLLGCQKSEEAPTFTASKSDSIEASASLSKEQPLIQLTKEVIDSQVELVNAKASFTSR
GMKVTLLAKDNPNSGVNIKPSEPWDLSEFDDFNLAMDIENPGPHSVQLFLNITDIDGATYTRSVAVPVGE
KATYYAKMRGHDLATPDGDVNQELNFLSGLRSNPETWESGDVQFISMWGKKNLNLKGITEISLSVQSALF
DKHIELSNIRLRPNPEMNTDFLTKIVDKYGQNATVEFPGKIHSQEELIQARETEAKQLDNKLMPDRSRFG
GYKEGPKLAATGYFRTEKIDGKWAMVDPEGYLYFATGLDIIRLSNTSTMTGYGFDDGLVDLGGDGVTPED
SKGLNRVNDEAIPSRHIVSDVRANMFNWLPSYDEPLGKWFGYRGSAHSGPVKKGETFSFYASNLERKYGE
QDPLEAWEQVTLKRMKNWGFSSLGNWTDPRFYQNNEVPYFANGWIIGDFKTVSSGNDFWSPLPDVFDPEF
ARRADVTASNVAEQVKNSPWCVGVFIDNEKSFGRSESNEARYGIVINTLTRDGKDVPTKAAFTGLMKKKY
GSISALNAAWGTKIASWGAFNAGIDSSIRNEVQLADYSEMLFHYGEKYFSVVNAALDKHMPNHMYLGARF
PSWGKPMEIVEAAAKHVDVMSYNVYKEGIHPKSWEFLQDIDMPSIIGEFHMGARDNGLFHPGLIQAATQE
DRAQMYIDYMHSVIDNPYFVGAHWFQYMDSPITGRAYDGENYNVGFVNVADTPYAPMIKAAKEVNSKMYP
RRFK

>AMJ73263.1
MKIHSISMAVAVACLSLAACNTQKNQTSLPSSSSQNVIDNLYSFDVPLNGKIETEFASATVQPMSPNSGK
ALHVSFEGDVAEASVKLIPAQNWDWSMHKEINLAFEATNPEEESVQLYISVMTESGNQASHSVIIPPGST
STYYFVLNGQVLDTDLGYKRSRMEAWQSNEQMAHFRIGSIKLDLSSVEAIRLYTRGNMVSKRLTIDNLRL
RENPDYGDEYRQNTVDKFGQNAKRDFPLKVHSEAELKEKANEELTRLNASGPLPDRSRFGGWKAGPKLEA
TGYFRTHKMQGKWWLVDPDGYLFFSNGLANVRMANLTTLTGVDFKDESVRYIDPEAVTPEDSMGIVNVSD
AVRDTRYIASEVRHEMFSWLPPYDDALADHYSYRRSVHAGPLTSGETFSFYRANLERRYGESSPGAYEEK
WQEVTLDRFQDWGFTSMGNWVDPAFYPNEQVPYFANGWIIGDFKTLSSVHDVWDSMPDSFDPEFVRRAQV
TINQIAKEIQSSPWCIGIFVDNEKSWGRTEGTLEQRYGLILDALSHSIDESPAKKAFVDALKQKYSSLNS
LNEGWGTSFTSWQALNNAWQPQQPTAALEKDLSMMLEMLSEQYFKVVHDALETALPHHLYMGARMASWGM
PDETISAATRYSDVLSFNIYKEGVQPSQWRFIEDIDLPSIIGEFHIGTNTDSGLFHPGLVAAADQEDRAK
MYQQYMQSVVDHPNMVGAHWFQYVDSPISGRAFDGENYNVGFVSVTDIPYTEMVNAAREFNTTLYPARFN
QR

>AMJ73260.1
MNKTAIALALSLSLLGCQKSEEAPTFTASKSDSIEASASLSKEQPLIQLTKEVIDSQVELVNAKASFTSR
GMKVTLLAKDNPNSGVNIKPSEPWDLSEFDDFNLAMDIENPGPHSVQLFLNITDIDGATYTRSVAVPVGE
KATYYAKMRGHDLATPDGDVNQELNFLSGLRSNPETWESGDVQFISMWGKKNLNLKGITEISLSVQSALF

DKHIELSNIRLRPNPEMNTDFLTKIVDKYGQNATVEFPGKIHSQEELIQARETEAKQLDNKLMPDRSRFG
GYKEGPKLAATGYFRTEKIDGKWAMVDPEGYLYFATGLDIIRLSNTSTMTGYGFDDGLVDLGGDGVTPED
SKGLNRVNDEAIPSRHIVSDVRANMFNWLPSYDEPLGKWFGYRGSAHSGPVKKGETFSFYASNLERKYGE
QDPLEAWEQVTLKRMKNWGFSSLGNWTDPRFYQNNEVPYFANGWIIGDFKTVSSGNDFWSPLPDVFDPEF
ARRADVTASNVAEQVKNSPWCVGVFIDNEKSFGRSESNEARYGIVINTLTRDGKDVPTKAAFTGLMKKKY
GSISALNAAWGTKIASWGAFNAGIDSSIRNEVQLADYSEMLFHYGEKYFSVVNAALDKHMPNHMYLGARF
PSWGKPMEIVEAAAKHVDVMSYNVYKEGIHPKSWEFLQDIDMPSIIGEFHMGARDNGLFHPGLIQAATQE
DRAQMYIDYMHSVIDNPYFVGAHWFQYMDSPITGRAYDGENYNVGFVNVADTPYAPMIKAAKEVNSKMYP
RRFK

>WP_059746353.1
MTFTFSPKYSTLSCSILLSLTVISGCSESSTSPNVVSEASNANATESLSIQAVNTDLAMGNVDVVKVLYD
FEHINDDSFANVDLTAANSIITDSIVTKNATTSIISTPKSNALEVKFDTEKSHIASVDFIASTAWDWSGH
TDLAIAVDITNPSSASTPIYVKAADDMHQQSRSTVIPAASSNTYYIVLSGDELDQNTGIRSNPKYWLTDF
EPIEWRGGEKQLDLSKMTSIHFELNGSLVDKALVFDNIRLITPKAVNSEYLVGLVDQFGQRISQDFNGKV
SSVEQLQRDSDNEIAALALHPQMDDRSKFNGWLNGPKLKASGYYRVDKYQGKWTLVDPEGYVFFSNGIAN
IRLANTSTITGYDFDPGLINIKLATDFTPEDSLGLNQVDKQALPSRKLMSPLRANMFTSLPNYDEPLGQN
FGYRRSVHSGALTAGETFSFYRANLQRKFGLDDPEQLFAKWQDNTINRMRSWGFTSFGNWVDPQYYQMNR
YPYFANGWIIGDFKTVSSGNDYWSPLPDPFDPVFTERARATIAQIAKEVDNNPWCVGVFIDNEKSWGMMD
SLQNQYGIVLNTMTRPIADSPAKVAFVSALKQKYSSIDNLNTAWALTTSWDEIANGITLDKINDAVKADL
SMLLTLYAEQYFKTVNATLKEHMPNHLYMGARFASWGMTPEVRAAAAKYADVVSYNYYKEGLDKGFWTFL
DEVDRPSIIGEFHNGSLDSGLLHPGLVPAQSQADRGKMYQQYMKSVINNQYFVGAHWFQYIDSPLTGRSY
DGENYNVGFVSVTDKPYKELVDAAKEVNRTLYINRFKEEKPANK

>WP_059746346.1
MNTGIVSLKTKKMILAAAIAIVCTGCQLDTPKKAQNSSAIVELSYTGDIVNVTNATAMKTDQGLSVYFNS
KDNAYTSVSFMPKTAYNWSSYDNVNLAFSISNPGKHSVQLYLDISDIDGNNYTRTVSIPVGSKPQVYYAK
LAGHDLATPDGDEKVELNFTSGLRSNPDTWESDEHQFTSLWGKKNLNLAGISKINLSVQSAMHDKKIVIN
NMQIRQNPAYDPTFLTGIVDQFGQNAKQDFVGKINSVDELITQRQQEALTLTGKRDENRSQYGGWSNGPK
LKATGFFRTEKVDGKWSLVDPDGYLYLATGIDIIRLANSSTMTGYDVDAKFIVQPTANDVTPEDSQRLNR
INSAAINSRFVASDVRKNMFTWLPSYDEPLGKHYDYRRSAHSGPLSKGETYSFYAANLERKYQTDTQEYM
QAWQDTTINRMINWGFTSLGNWTDPAYYNNDKIPYFANGWIIGDYKTISSGDDFWGAMPDVFDPKFEERA
MATAKVIYEEVKGNPWCVGIFVDNEKSFGRSDSDNSHYGIVINTLTKDAAEVPTKAAFSNTIKQKYHTID
KLNLAWGKKLTDWSEFDKGFDSSLQNQQQLEDYALLLTVYADKYFSTVDKALQHYLPNHLYLGSRFPDWG
MPIEVVKSSAKYVDVISFNSYKEGLPKSAWAFLEDIDMPSIIGEFHIGAKDSGLYHPGLILASDQQDRGE
MYKDYMNSVIDNPYFVGAHWFQYIDSPITGRAYDGENYNVGFITVTDTPYPHMVEAAKEINSNMYQRRFK
QQE

>KVX01292.1
MTFTFSPKYSTLSCSILLSLTVISGCSESSTSPNVVSEASNANATESLSIQAVNTDLAMGNVDVVKVLYD
FEHINDDSFANVDLTAANSIITDSIVTKNATTSIISTPKSNALEVKFDTEKSHIASVDFIASTAWDWSGH
TDLAIAVDITNPSSASTPIYVKAADDMHQQSRSTVIPAASSNTYYIVLSGDELDQNTGIRSNPKYWLTDF
EPIEWRGGEKQLDLSKMTSIHFELNGSLVDKALVFDNIRLITPKAVNSEYLVGLVDQFGQRISQDFNGKV
SSVEQLQRDSDNEIAALALHPQMDDRSKFNGWLNGPKLKASGYYRVDKYQGKWTLVDPEGYVFFSNGIAN
IRLANTSTITGYDFDPGLINIKLATDFTPEDSLGLNQVDKQALPSRKLMSPLRANMFTSLPNYDEPLGQN
FGYRRSVHSGALTAGETFSFYRANLQRKFGLDDPEQLFAKWQDNTINRMRSWGFTSFGNWVDPQYYQMNR
YPYFANGWIIGDFKTVSSGNDYWSPLPDPFDPVFTERARATIAQIAKEVDNNPWCVGVFIDNEKSWGMMD
SLQNQYGIVLNTMTRPIADSPAKVAFVSALKQKYSSIDNLNTAWALTTSWDEIANGITLDKINDAVKADL
SMLLTLYAEQYFKTVNATLKEHMPNHLYMGARFASWGMTPEVRAAAAKYADVVSYNYYKEGLDKGFWTFL
DEVDRPSIIGEFHNGSLDSGLLHPGLVPAQSQADRGKMYQQYMKSVINNQYFVGAHWFQYIDSPLTGRSY
DGENYNVGFVSVTDKPYKELVDAAKEVNRTLYINRFKEEKPANK

>KVX01285.1
MNTGIVSLKTKKMILAAAIAIVCTGCQLDTPKKAQNSSAIVELSYTGDIVNVTNATAMKTDQGLSVYFNS
KDNAYTSVSFMPKTAYNWSSYDNVNLAFSISNPGKHSVQLYLDISDIDGNNYTRTVSIPVGSKPQVYYAK
LAGHDLATPDGDEKVELNFTSGLRSNPDTWESDEHQFTSLWGKKNLNLAGISKINLSVQSAMHDKKIVIN
NMQIRQNPAYDPTFLTGIVDQFGQNAKQDFVGKINSVDELITQRQQEALTLTGKRDENRSQYGGWSNGPK

LKATGFFRTEKVDGKWSLVDPDGYLYLATGIDIIRLANSSTMTGYDVDAKFIVQPTANDVTPEDSQRLNR
INSAAINSRFVASDVRKNMFTWLPSYDEPLGKHYDYRRSAHSGPLSKGETYSFYAANLERKYQTDTQEYM
QAWQDTTINRMINWGFTSLGNWTDPAYYNNDKIPYFANGWIIGDYKTISSGDDFWGAMPDVFDPKFEERA
MATAKVIYEEVKGNPWCVGIFVDNEKSFGRSDSDNSHYGIVINTLTKDAAEVPTKAAFSNTIKQKYHTID
KLNLAWGKKLTDWSEFDKGFDSSLQNQQQLEDYALLLTVYADKYFSTVDKALQHYLPNHLYLGSRFPDWG
MPIEVVKSSAKYVDVISFNSYKEGLPKSAWAFLEDIDMPSIIGEFHIGAKDSGLYHPGLILASDQQDRGE
MYKDYMNSVIDNPYFVGAHWFQYIDSPITGRAYDGENYNVGFITVTDTPYPHMVEAAKEINSNMYQRRFK
QQE

>KVX01280.1
MKLFYQFTYLTIVIALSGCNDSSTVNTNNNQQDTSMLEPVSSIAQKPVSQPNTNIVLLDLVDFSSVEQQA
WVKNTASQTEVTNNKLKILFSAQHNISTTTIKPEKPWDLSALSRYNLAFNVTNSQDKSVHFYLSVENNAA
EYQSRSISIAPNYKGTVYFPLDGVEANSETGLWGDAPPWKTDDLLMVWRSWRADKVDLSQIAALNFFTIG
LLESQAIEISDIRIRQNPELDPQWMQNIVDKYGQNSQVDYALKVKSDSQLAQQTEQELAQLDASTGMSDR
SAYGGYTKGPKLKATGYFRTEKINGKWWMVDPDGNVFFSHGPANVRMANMSTLTGVDYKDNKVRYRSPDE
TTPEDSMGTVQIPQAIKDTHYVSSTLRHKMFEWLPKYSDPLAKHYSYRRSTHKGAMKHGETFSFYQANLE
RKYGNDTPGAYLKKWHQVTLDRMKDWGFTSFGNWVDPAFYQAKQVPYFANGWIIGDFKTLSGSVNHWGLM
PDPYDLEFAKRAKITIDEIADSVDSSPWCVGIFIDNEKSWGEREGSVEQRYGIILDALSKNVIESPAKKA
FSDHLKQQYKDIKQLNTAWDSDIEDWHTFDSGVRFTHFSTLQTSDLSKLLELLGEQYFTIVHNTLAEKMP
NHLYMGARMANWGMPDEIIKASIKYSDVLSFNIYEEGMQTNFWQFLEQVDLPVVIGEFHIGSNEGSGMFN
PGIVHASGQQDRADKYKKYMQSVLEKPYMIGAHWFQYIDEPITGRAFDGENANIGFVTVTDTPYPALINA
VKEVTSTMYQQRLDNK

>WP_058230569.1
MQHDNKTILIDDFEHASNSYKLTNKNIKTTQVIKNKNKALELSFSTKHKFSGITLKPNKLWDLSVLGNSA
LFFDVSNVRDFPVMLSVNITGKDKQVQRRTIGLTTNEHATLYFELNSQTLNVDTGLRDTPGSFKTAARKM
ILRGAKLNVDFSQVESIAIYTETQINPTAVTVDNLRFETIPDAKPDFLTNIVDKFGQSTQVNYPLKVSSE
QQLRAIANKELNDLSKVTPRADRSKFGGWKQGPKLKATGFFRTEKVNGKWALVDPEGYLFFSSGIANARM
ANTTTFTGVDYRDDAVRARDPDDVTPEDSKGLNSNLAKYQKSAYIAYPDRRAMFNWLPQYNDKLANHYSY
KRLSHLGPIQHGEVFSFYQANLERRYAQQYPDSYIDKWREVTLKRMQDWGFTSFGNWTDASFYNNQQVPY
FANGWIIGDFKRLSSGFDYWGAMPDPFDPEFVKRANITTQVIAQEVQNNPWCIGVFIDNEMSWGGEGSTT
LRYGIVLDALSKTTGNSPTKSVFSDMLKQKYKTISQLNQAWNRNIKSWKVFDNTGVNYKKDSNFNNAMIA
DLSWLLIRFSDEYFKVVNHSLKSVLPDHLYMGARFTSWGTSPEARWSAKRYADVISYNYYREGLDPMTWD
MLKELDMPTIIGEFHIGSGDTGQPNPGIIHAANQRDRADMYKTYMKTVIDNPYLIGAHWFQYIDSPITGR
AYDGENYNVGFVTTTDIPYPELVEAAKQVHKSLYQQRYGDVKIK

>ALQ09764.1
MLCCLVGLAGCNNGKISEKKPNNTVVDFKKQLVIFDFEKNNYSDNINTINASTKLVEQSGNHKLQVNLYS
KSHTESDFEFVNPQGWDWQAIGNFALAIDIQNPNSASTHIYVKTTDKSGKSQTRSVVVPGHSENTYYIEL
KGANLNINSGIRSNPPSWHSGYTPIIYRGGQKNIDVSSIVKVSLGVKGLLEDKRFLIDNLRLIKPTNFDV
NYLKGLVDEFGQNAKLNFINKVTSTEQLLVISQKEQAQLQAQPLQGRSKFSGWKNGPQLKATGYFRTEKY
KGKWSLVDPEGYLYFSTGIDNVRMANTSTITGYDFDRSYIKQREAGDLTPEDSLGLNPAPVAAWPTRYQN
SKLRAEMFNWLPKQDDPLADNYGYRREVHSGAVKKGETFSFYRANLERKYQTHNNNELMKQWQKTTVNRM
LSWGFTSFGNWIEEQYYHTKKLPYFANAWIIGNFKTVSSGNDYWSPLPDPFDPLFVERADVTLAKVAQQV
KNSPWCVGVFIDNEKSWGMMNSDTTRYGIAINTLKNNAKNSPTKAEFVLLMKNKYSKISKLNKAWNTTLS
SWDEFAEGVTLTQFNDHVNTDLSAMLFHYANQYFAVVDEAITKHLPNQLNMGARFADWGMTPEIRAASAA
HVDVMSYNYYREGLNQEFWEFLSDIDMPSIIGEFHNGALDSGLLNPGLIHTQSQQERGSKYQNYMNSVID
NPYFVGAHWFQYIDSPLTGRAYDGENYNVGFVNVADIPYEPLVNAAKNVNKSLYERRYRNDKN

>ALQ09758.1
MQHDNKTILIDDFEHASNSYKLTNKNIKTTQVIKNKNKALELSFSTKHKFSGITLKPNKLWDLSVLGNSA
LFFDVSNVRDFPVMLSVNITGKDKQVQRRTIGLTTNEHATLYFELNSQTLNVDTGLRDTPGSFKTAARKM
ILRGAKLNVDFSQVESIAIYTETQINPTAVTVDNLRFETIPDAKPDFLTNIVDKFGQSTQVNYPLKVSSE
QQLRAIANKELNDLSKVTPRADRSKFGGWKQGPKLKATGFFRTEKVNGKWALVDPEGYLFFSSGIANARM
ANTTTFTGVDYRDDAVRARDPDDVTPEDSKGLNSNLAKYQKSAYIAYPDRRAMFNWLPQYNDKLANHYSY
KRLSHLGPIQHGEVFSFYQANLERRYAQQYPDSYIDKWREVTLKRMQDWGFTSFGNWTDASFYNNQQVPY
FANGWIIGDFKRLSSGFDYWGAMPDPFDPEFVKRANITTQVIAQEVQNNPWCIGVFIDNEMSWGGEGSTT

LRYGIVLDALSKTTGNSPTKSVFSDMLKQKYKTISQLNQAWNRNIKSWKVFDNTGVNYKKDSNFNNAMIA
DLSWLLIRFSDEYFKVVNHSLKSVLPDHLYMGARFTSWGTSPEARWSAKRYADVISYNYYREGLDPMTWD
MLKELDMPTIIGEFHIGSGDTGQPNPGIIHAANQRDRADMYKTYMKTVIDNPYLIGAHWFQYIDSPITGR
AYDGENYNVGFVTTTDIPYPELVEAAKQVHKSLYQQRYGDVKIK

>ALQ08241.1
MTYKKNILLTSLLLALSACNQEDVKVDRPTTDAVQIKSTNTLMYLFADDTHHTATSVDYQSNSAIVKNEN
SVLNVQFQSKKNSYASIVFSPEKPWDWSEFNDFNLAFELANPGTHSVQIYLDISDIDGANYTRSVNVPVG
GYNTYYAKLDGHDLATPDGKENVELNFTSGLRSNPDTWESDEVQFISMWGKKNLNLKGIAKIAISVQSTL
HDKELAIKSISLRKNPQFNTAFLTKIVDEFGQNAKQEFAGKVHSEAELLSDKKQEATQLLSKRPTNRSRF
GGWAEGPKLEATGYFRTAKYNDKWSLVDPDGYLYLATGIDIIRLANSTTLTGYDFDQALLAKPADAGVTP
EDSKGLNQVNKEALKSRFVASQVRKNLFEWLPDYSDPLGKHFGYRKSAHSGPLEHGETYSFYAANLERKY
GQNNADYMQKWREVTLDRMITWGFSSLGNWTDPSYYDNQKVPYFANGWIIGDFKTVSSGNDFWGAMPDVF
DPEFTVRANETVSVVAKEVKNSPWAVGVFIDNEKSFGRPDSVKSHYGIVINTLGRDAKTVPTKAEFSRLM
KEKYTDVAKLNKVWHLNLASWVEFDKGVTVDIKNEEQLVDFSILLTAYADKYFSVVNAAMDKYLPNHMYL
GARFPDWGMPIEVVKASAKYVDVISFNAYKEGLRDDKWAFLSQFDKPAIIGEFHVGSSDSGLFHPGLIHA
ANQQDRANMYTDYMNSVIENPYFIGAHWFQYIDSPITGRAYDGENYNVGFISVTDRPYIEMIEAAKAMNE
SMYERRFKK

>WP_055024526.1
MNIKLDTRNALRYSFIATTILSTIACQHDTQNIGQNTTPTESVPLVVNSKQAMDLINFNSQSNNIIKPVA
AQTSISDGKLHVAFNSKDNNYSGVSFVPEKAWDWSDLNDFNLAFDIVNTSNHSVQLYLDISDIDGFTYTR
TVNVPVGDNIQTYYAKMAGHDLGVMDSDHKVELNFTSGLRSNPDTWQSNEHQFTSMWGKKNLNTAGIAKI
SLSVQSNLHDKSIDISRVQIRQNPKFDPLFLTDIVDQYGQNKKQDFIGKVHNDLELEQQRVDEAKTFTGK
VAEDRSRYGGWLSGPKLTATGFFRTEKVNGKWSLVDPDGYLYLATGIDIIRLSNSSTLTGYDFKQAYIVQ
PPKDNVTPEDSQKLNRVSDEAIASRFIASDTRTNMFSWLPDYDAPLGKHFGYRRSAHSGPLKHGETFSFY
SANLERKYGQQAEGYMQAWEDTTVNRMLDWGFTSLGNWTDPRYYDNNKIPYFANGWIIGNYKTVSSGDDF
WAPMPDVFDPEFEKRAYATAKVIYDEVKGNPWCVGIFVDNEKSFGRSDSTESRYGIVVNTLTKNGAEVPT
KAEFTRLMKQKYVDINKLNQAWNKSITDWAEFDSGIDSSINNSEQINDYGLLLTAYADKYFSTVNKAVKH
YMPNHLYLGSRFPDWGMPREVVNASAKHVDVISFNSYKEGLTKKSWAFLQEIDMPSIIGEFHIGAKDSGL
YHPGLILASDQQDRAVMYKDYMKSVIDNPYFVGAHWFQYIDSPITGRAYDGENYNVGFVSVTDTPYPYMV
EAAKVINKAMYQQRFKEK

>WP_055014349.1
MTTTNTSLTVNLIALSLACIFANGCSDTDKTPVQERAANTPVDPFYQAETLTKLVDFSNQQQQSWFFDKY
TSHQFIESANSNKLALNFSAKGNISELKIAPPSPWDLSSFNNYNVAFDVENTSASSVHVYLSIENPEGQL
QSRSVSLPANYSGTVYFPLDGKEAQTNTGMWGDAPPWQTNDRLMVWRSWRDDGGDFTKISAMNLFTIGVL
EDKSILIGDIKLRKNPPSDPNWMVNILDKYGQYTKQTNALTITSDEQLKRLADIELAELAKSKGMSDRSR
FGGYAKGPKLEATGYFRTEKVDGKWWMVDPEGYLFFSHGPANVRMANLTTITGVDFKDDSVRNRSSDEIT
PEDSMGIIKVSDDARQSRFITSELRHNMFQWLPDYNEPLSEHYSYRRSTHKGPVAHGETYSFYRANLQRR
YGETSPKSYLDKWHEVTLDRMKDWGFTSFGNWVDPAFYDKESVPYFANGWIIGDFKTLSGHTNHWGLMPD
FYDPEFKKRAEITIDAIAKSIKSSPWCIGIFIDNEKSWGEREGSVEKRYGIILDALSKDAQQSPAKHAFA
THLKQKYTSINALNKAWQSDITSWDELNNGVTFTTYTDAQIADISKMLEMLGEQYFKVVNGTLAKKLPNH
LYMGARMANWGMPDEIIKASVKYSDVLSFNIYEEGMQDHYWQFLEDVDLPVVIGEFHIGTATDSGLFNPG
IVHAANQTDRAAMYKNYMQSVLEKPYMVGAHWFQYVDEPVSGRAFDGENANIGFVTTTDIPYPEMIQAVK
DVTSTMYENRYDK

>WP_055014338.1
MKYKKNLAVTALVLTLTGCNQQTNNVSDPVAAKATSSSTLLQLLDDAQHTADTAVKIKTSGAQVTNNDNI
LNIAFNSEQNLYSGVTFTPDTPWDWSQFKDFNIAFELANPGQHSVQVYLDISDIDGANYTRTVNVPIGDF
HTYYAKLDGHDLATPDGDESVELNFTSGLRSNPATWQSDETQFISMWGKKNLNLKGITKISLSVQSALHD
KALQIKSIDLRTNPEFDTEFLTHIVDKFGQNAKQDFAYKVDSEAELIEDKKQEARQLLSHRPAERSRFGG
WAQGPKLEGTGYFRTAKYNGKWSLVDPDGYLYLATGLDIIRLANSTTLTGYDFDQSLFKSTNESGVTPED
SKGLNRVNKEALPSRFVASQVRKDLFEWLPSYDEPMGKHYGYRKSAHSGPLAHGETYSFYSANLERKYGQ
NNSDYMQKWREVTLKRMITWGFTSLGNWTDPSYYDNQQVPYFANGWIIGDFKTVSSGNDFWGAMPDVFDP
HFAERANVTVENVAKEVKNSPWAVGVFIDNEKSFGRPDSVQSHYGIVINTLGRDAATVPTKAEFSRLMKQ
KYTDINQLNKVWHLNLASWAEFDKGVKVDVKNNDQLADFSIMLTAYADKYFSVVDAAMDKHLPNHLYLGA

RFPDWGMPIEVVRASAKHVDVISFNAYKEGIKPKKWEFLSEFDKPTIIGEFHVGALDSGLFHPGLIHASN
QQDRAKMYTDYMRSVFDNPYFIGAHWFQYIDSPITGRAYDGENYNVGFITVTDRPYTEMVEAAKTVNNEM
YERRFKK

>KPG00157.1
MTDIPRTRWGGLATRKVQATGFFRVAQIDGVWWFIDPDGGRFLSKGVVSVQFDHDNIKGTERRPYREACL
HKYGSHDAWRGAAADRLRGWGFNTLGAWSEPELARAGGAPLASAAGVAYLATAYGEGRGWPQCDLFDPAF
ESFAQQRAQQICGPSRDDAGVLGWFIDNELQWGPDWRGENELLPTILGGIAAPYSRGVAVALLRDRYKDI
AAFNAAWRSALISWDELAGTPIAAPPFKRNFLTHDHAQERDPSRARYFADCDAFAGVLAERYFAVSTAAI
RAAAPHHLVLGSRFAYVPPPQVITAAGRYCDVISVNCYEALPGAVIEAYAATGRPCLIGEFSFRGDDAGL
PNTQGAGPRVETQADRAAGFARYVGAALRHPNLIGYHWFVHADQPAEGRWDGENSNYGVVTIHDEIYPEL
TEAMRVVNDDAEWLHEAAAAAARRVATPPAA

>GAA78452.1
MTYKKNILLTSLLLALSACNQEDVKVDRPTTDAVQIKSTNTLMYLFADDTHHTATSVDYQSNSAIVKNEN
SVLNVQFQSKKNSYASIVFSPEKPWDWSEFNDFNLAFELANPGTHSVQIYLDISDIDGANYTRSVNVPG

>GAA79964.1
MKKRSLYCAVSSALLLSSTFNVHAVKVQFNIDTKHAVGQFDSFDRRKFITLHSSNTESDWFGNNAQSLNA
PNAVPDLMTHFLEDYDVYFGRDTGGMKYQLTQLPEDSAKPGYASAATATTNGGGVKWNYTNITTAQAKTM
RKHEGRNSDLIVAAQQHPYYPDGTKIGNQNWSFSQRDTAAEPLGTAMGDYMAQFLQKYFKSGPSDSLGQK
RPTYVEVMNEPLFELHDFPHTGYDKESLYDIFRLHNSVADVINANPALNDVKVGGFTVAFPDYEKGSQFF
DNWKQRDKAFLDIAGNKMDFISMHLYDFPNFPGGPGGQHQIQYRKGSNMEATLDMVEQYMAYKWGSIKPL
VISEYGSQLQGSFGTKWTPQRDWLCLKAMSSMLMSFMERPNY

>GAA79963.1
MKGTRIDSWASDMDIQADAYVDGKDVYLVLNSLEFSPTNIDLSVLGKGSNNVTGVNIKHLYPNAQGKPVL
DNSNRTTLPSSVVLGSESTMIIKVSHQKNVVINNINQETKHYASAVVKNIAANATQFFTINNVDKGANGE
ATLRLGVGRPHGKSLTPVVTVNGNRVAIPTDFRGYDQKNGGLGRERFFGVIEIPVPYNNIKKTNNIEVTF
PDSGGAVSSLTLQNFKMSKHITR

>GAD03451.1
MLFKKSNLAILVSVVLAGVSTSNVIADDTKQSSENAATSGDMTSAATPLDFTEPAVLEKITNSHSQFSVL
KKTAEQSKDGLKMNFDAISEAEAQSKWPNVKIHSKAGPWDWNTKGGLKVALENPGSEDVRIEMKVSDNIG
IMGSADNQVDLPIILPAGKTTTVDFLFNGTQMNIDGYRGGAKLNLKSIAEIQFYSVGPIAAQEVVIRDIN
FIERTGDFVKSEAREAEVIAAPIPTLLALSDFDDGSKGIVSKTHGTTITSVKRDEGKGLKIDYSADASYP
SVTFSADKPWNWSEHGDFTLALDIENIGDAGAQLFIRVDDDVNEKQGGSANGVIHSRTGYVQLPAGEAGT
YYFTLEELAKTLDSGMRGEPPKKSYQAQAINFGWGEQKLDLSNIVSFQLYMQDLQKDLSLVIDNIRLVPN
LSADTSRYEGLLDEFGQFTNEDWAEKIHSAEELQAHAKADVKLIDSAKPMDDRTPYGGWKNGPKLEATGY
FRTEKVDGKWSLVDPSGYLYFATGLDNIRMDDTYTTTGVGFTDLVLSEDLSLRPSQISQDSYVDNQDARS
VASQLRNSMFTWLPSYQDALAQNYQYSTMIHTGPLEHGEVYSFYSANLQRKYAPDSRDEAIAVWRDVTLA
RMLDWGFTSLGNWADPSFYGNQKVAYVANGWIVGDHQRINTGNDYWGPMHDPYDPEFVESVKTMAKQVAA
EVEQDPWCIGTFVDNEMSWGNTEFDANHYALAIAALRADAKDSFAKAAFVGLLEAKYAQDIQALNKAWGS
ELKSWDELAKGYVHQGDLNDALKADYSMFLADHSDRYFAIVQQQMKQVLPNHLYLGARFTEWGITPEAAN
SAAQYVDVMSYNLYGNDMSKGDWSHLAELDMPSIIGEFHFGATDSGMFHPGLVAADTQQGRAEKYAHYMD
SVIANPYFVGAHWFQYLDSPTTGRAWDGENYNNGFVTVADSPYEKLVAGAAEVNRKLYPQRYPELVK

>GAD03444.1
MKFTKNKIAALLSLTLLGVYGCGSTPSSSDAEGAVEDVGGTIPDFESAAFFKKVKKDHAKAEVVSDQGVT
SGSSALKVNFDSVSEANKFKYWPNVKVHPDSGFWNWNAKGSLSLDITNPTDSPANIILKLADNVGVMGSG
DNQLNYAVNVPAGETVPVEMLFNGTKRKLDGYWGGEKINLRNIVEFQIFVQGPMDAQTVIIDNFNLVDAT
GDFIEASGQEVKVSGPIPTVASVTSFDEGQPTFVAFDRSAAATVTELKTDMGGLLAVKLAATNAYPNITF
KAPQPWDWSEYGDFSLAFDLESKTDEPLQLFVRVDDAENENWGGTANGVVDSMSSYVTLAPGDDGTFYLP
LGQTGSQIVSGMRAEPPKKSYNAQAISYGWGEKSLDTSNIVSFQLYLQNPTKDAEFNIKSVRLIPNIDAD
ATRYEGLIDQYGQFTGSEWPKKISEDEELETMGKLAKMSLKSTSQMPGRSIYGGWADGPKLKGTGFFRTE
KVDGKWSLVDPQGNLFFATGVDNIRMDDTVTITGHDFADKDKRSGKEVASEVRRSMFTWLPEDDDVLAEN
YDYANWVHSGALKKGEVFSFYGANLQRKYGGTFSEAEKVWKDITIDRMVDWGFTTLGNWADPMFYDNKKV

AYVANGWIFGDHARISTGNDYWGPIHDPFDPEFVNSVKAMTKKLMTEVDKNDPWMMGVFVDNEISWGNTK
NDANHYGLVVNALSYDMKKSPAKAAFTEHLKEKYWAIEDLNTSWGVKVASWAEFEKSFDHRSRLSKNMKK
DYAEMLEMLSAKYFSTVRAELKKVLPNHLYLGARFADWGVTPEIAKGAAPYVDVMSYNLYAEDLNSKGDW
SKLAELDKPSIIGEFHFGSTDSGLFHGGIVSAASQQDRAKKYTNYMNSIADNPYFVGAHWFQYIDSPTTG
RAWDGENYNVGFVSITDTPYVPLVEAAKKFNQDVYMLRYKK

>GAD03438.1
METRLRADSAKTISEHEQSILIYDFAEQIPKAFSFSNVDAELVSEGNGITTGSQALKVTTHSKENFYTSI
FIEPEQPFDWSALPNFSFAFDVTNLGRRSTQIFINIFDKQGQMHSRSINVAGGSCKTYLNELKGEFLKGG
LNYESGFRSNPAAWDTPFHYATWMWGEMNIDLSAVAKIELSIHGTLIDHQLVLDNFRVIFTPECNPDFLK
GCLDKFGQNALVETAEKVHSEEELLAVTAKELKALEQGAMPQRSRFSGYTGGPQLEATGFFRTEKIDGKW
SLVDPDGYPYFATGLDIIRLANSFTITGIDYDHSKVAARSDDDVTPEDSKEKLEISQEAFDSAYVANQTR
RDFFDWLPSYDDPLAEHYSYMRELFEGPVDRGEIFSFYSANLQRKYGQDGADYMGKWREVTMDRMLNWGF
SCLGNWTAPEFYSNDKIPYFANGWIIGDFKTVTSGDDFWAPLPDPFDPVFRERAEATVSQVKAEMQGSPW
CVGIFIDNEKSWGRMGTINGHYGITIHTLGRSDEESPTKAVFTQALKDKYGTVEQLNQAWGTNIDSWQAV
AGGVSDLAHNEAQLSDYSMLLELYASEYFKVVNESLKAQLPNHLYLGARFADWGCNPEVVRAAAKHVDVV
SYNYYKEGLHPEPWKFLAEVDMPSIIGEFHIGVKEGFFHAGLVTANDQTERGEMFEDYLNSVIDNPYFVG
AHWFQYIDSPITGRSFDGENYNVGFVGITDVPYQPMVDAAKRVNGGMYQRRFKNAKK

>WP_033029506.1
MLCCLIGLAGCNNGKISEKKPNNTVVDFKKQLVIFDFEKNNYSDNINTINASTKLVEQSGNHKLQVNLYS
KSHTESDFEFVNPQGWDWQAIGNFALAIDIQNPNSASTHIYVKTTDKSGKSQTRSVVVPGHSENTYYIEL
KGANLNINSGIRSNPPSWHSGYTPIIYRGGQKNIDVSSIVKVSLGVKGLLEDKRFLIDNLRLIKPTNFDV
NYLKGLVDEFGQNAKLNFINKVTSTEQLLVISQKEQAQLQAQPLQGRSKFSGWKNGPQLKATGYFRTEKY
KGKWSLVDPEGYLYFSTGIDNVRMANTSTITGYDFDQSYIKQREAGDLTPEDSLGLNPAPVAAWPTRYQS
SKLRAEMFNWLPKQDDPLADNYGYRREVHSGAVKKGETFSFYRANLERKYQTHNNNELMKQWQKTTVNRM
LSWGFTSFGNWIEEQYYHTKKLPYFANAWIIGNFKTVSSGNDYWSPLPDPFDPLFVERADITLAKVAQQV
KNSPWCVGVFIDNEKSWGMMNSDTTRYGIAINTLKNNAKNSPTKAEFVLLMKNKYSKISKLNKAWNTTLS
SWDEFAEGVTLTQFNDHVNTDLSAMLFHYANQYFAVVDEAITKHLPNQLNMGARFADWGMTPEIRAASAA
HVDVMSYNYYREGLNQEFWEFLSDIDMPSIIGEFHNGALDSGLLNPGLIHTQSQQERGSKYQNYMNSVID
NPYFVGAHWFQYIDSPLTGRAYDGENYNVGFVNVADIPYEPLVNAAKNVNKSLYERRYRNDKN

>WP_053084491.1
MIKNKLRKSLIILASSYMALGCSENSQQKTESSQSQLNQTYLYQFDLATELNDFLEENTQLSLIPETAAI
ENATSKRLSVTFLAKENYKSSLTIQPKKPWNWQQDKAFYLALDIENPTQDSTHIFGQVIDNKKQTHTRSV
VIPKQSKHTFYIELKGEDLNQETGIRSNPASWQSKDKPFIWRWGVKNLDLTQIVKVKFSVTSLLKDKTII
LDNIRLVDSPEMDPNYLADLVDKYGQSTRVDYPQKVETDDVLQKVSQAELEQLDGKLMPDRSKFGGWKFG
PKLEATGYFRAEKLGNTWTMVDPEGYLFYSHGIANVRMANTTTITGRDYKKPIPQTAASDVTPEDSKGVI
RASDDVLKTVYVASELRHKMFQWLPEYDDSLAKHFGYRRSVHTGALEKGETFSFYQANLERKYGDDFIPK
WRDVTVDRMINWGFTSFGNWIDPMFYQLDRFPYFANGWIIGDFKKVSSGADYWSPLPDPFDPEFSKRAKA
TVDVIAKEVQQNPWCVGIFIDNEKSWGSTSSLQAQYGIVIHTLNRTATESPTKAEFVKLMQTKYQTIEQL
NQSWNTKISSWNSFATGFKTQDLESSANMIADYSAMLSHYAQAYFDVVNKALKQQLPNHMYMGARFADWG
MTAEVVEAAAKYADVVSYNFYKEGLQDKHWAFLEQIDKPSIIGEFHMGATDTGLLNPGLVHAQSQQDRAD
MYTEYMQSVLKNNYFVGAHWFQYTDSPLTGRAYDGENYNVGYVSVTDTPYKEMVEATKKITRDLYSNKYG
HLK

>WP_052260855.1
MSTSIIEPTQKVPSLKEHRLLNFSRESLPNELMFLHTDAFITPVGENQYGVRITSRAKDNFYTATNLVPE
MPWDWSQLPNFSFAFDASNLANHSTQVFINIFNTQGEMHSRSITIPACRNPRTYICELKGDYLQGKTNHY
SGLRSNPAPFDTPYEYATWMWGAINIDLTSICKIELSIHGSLLDHELVLSNFRLIQSPPTNPDYLTGIID
KFGQNANVDYVQKVHSESQLLALKDAELETLKAGKMPQRSTFSGYTGAKQFEATGYFRTLKIGGKWSLID
PEGYPYFATGIDIIRLANAYTMTGVDYDHSKIEQRQADDLTPEDSIEKVTVSSEAKQTAFIASDMRRNCF
NWLPSYDDPLAEHYGYMRELWEGPTAQGETFSFYAANLQRKYGENYRQIWSDVTIDRMLNWGFTCLGNWA
APEFYRNEKIPFFANGWIIGDFKTVSSGDDFWTALPDPFDPQFEQRAIATVQQVKAEIQGSPWCVGIFID
NEKSWGRMGTIQGQYGIPIHTLSRDAVESPTKAVFMTVLQEKYQTIEALNQAWSTQFTSWEIVAKGVCDL
AHNEAQCEDYALLLETYASEYFRIVSQAVKAELPNHLYLGARFADWGMTPEVVRACAKHADVVSYNYYKE
GLHPEPWTFLEEIDMPSIIGEFHFGARDTGFFHPGLVSAESQDERGEMYERYVQSVVDNPYFIGCHYFQY

IDSPITGRSFDGENYNIGFVSVTDVPYDGMVNAAKTINSSLYQQRFNQLTK

>WP_052480967.1
MKLNKRASLIAKASFCLTAMLISACSHVSEDSPKQKRLIDFSEKVDLKQIVPLSASVSYTDLDGGAAQID
LHAASNHIAGFTIQPDKPWNFSDFKALALTLDIANPTDESVSMYVSTSDQSGDFQMRSVVVPANSKQRYS
IDIDVPELAVETGVRSNPESWVNSYQALIWRGGEKQLDTSAITAIRFDVRGALHDKTLIVDNVTAIEPLE
FKDDYLKSLVDSFGQNAKMDFAQKIDSVEQLRSVSDAEQQQLQSGPLTDRSRYHGWRDGPKLEATGFYRT
EKYQGQWSLVDPEGYLFFSNGIANVRMANTSTMTGYDFDHKLIHERSSNDFTPEDSLGLNRVPDQALSSR
HVSSKLRADMFTWLPDYQDPLGKHFGYRREVHSGAMERGETYSFYRANLARKYRTHDTATLMQQWRDTTV
KRMENWGFTSFGNWVDPAFYEMKNYPYFANGWIIGDFKTVSSGNDYWGALPDPFDPAFTERAYATVKKIS
EEVANSPWCVGVFIDNEKSWGIMGSVESQYGVVLNTLTRKASDSPTKAAFAAYLKQQYGSIDELNNAWGT
QVASWKILEEGIVLNRYTDAALADMAAMLELYTAEYFRIVDAAMDELMPNHLYLGARFADWAMTPEVRQA
AAPYVDVMSYNYYREAVSDVFWDFLAELDMPSIIGEFHNGALDSGLLNPGLIHAESQYDRGVKYQEYVNS
VIDNPYFVGVHWFQYIDSPLTGRAYDGENYNVGFVSVTDIPYKPLVDAARAVNRSLYKRRYD

>WP_051479578.1
MATFEQGLPNTLKTLNAKAQLANTSELEGNKALKIEFDATHLKSELSFKAAKPWDWSQYGDINLAADISN
LSTESIQIYIEIKDATGWPHIRSVSIPAKYTGTYYALLKGPQLELDSGLREDPKSWQSDDHKMFWMRGAK
KLQLDKITSVRFFVESIKNNKTLLIDNLRVRQNPRFNQDYLKDLTDAYGQSYKFDYPTKVTSDEQLKALA
EAEIAQLEQQGTMADRSKFGGWASGPKLEATGYFRTEKVDGKWAIVDPEGHLFFSSALANIRIANTTTFT
GVDFKDDSVRYVDPEDVTPEDSLGIRPVSNQAQQTRYIRSDMRHKMFTWLPGYDHELANHYSYRRSSHKG
PMAHGETYSFYQANLERRYGEEYPDSYLDTWRDVTIKRFKNWGFTSTGNWTDASFYQMNRIPYFANGWII
GDFKTVSSGQDVWSRMPDPFDPEFKRRAIITAQVIGEEIKNNPWCIGIFVDNEKSWGNDSSLQRRYGIVL
NTLTREDSDSPLKAKLTEMMQTKYQSIQALNQAWQTDIDSWQTFSKGVKVTDLNDTVVADLSDMSFTYAN
EYFKIVHDALADVAPNHMYMGVRMAAWGLTTEAANAAAQYADIMSYNFYREYAHPKAWEFLERLDKPSLI
GEFHIGATSDTGLYHPGLIHAADQQDRAQMYKDYMYSVIDNPYMVGAHWFQYLDSPLTGRAYDGENYNTG
FVTTTDVPYQQMVEAAKEVNANLYQRKFGNTVKK

>WP_051235400.1
MSTTSISSSSAAASEGATQVYVDSDVTRAVGDADTFHREAFITLHASHTEQDWWLGENADSVGGDNLMAE
FVTEYDVYFGRDTGGMAWQLSQVPEDPEKPGYADDEAIESRGNDATGWYSARQDVRGPIQRAQEHRNQQM
IVAAQQHPYWPNGTPTGQGWTFSQTDTPEEPLGTATGQYMSQFLKHFFRQSEGEPGQPKPLYVEVMNEPL
WELVTVAEDPVDLTTIFEFHNTVAKEVRALNPDVLLGGYTVAFPDFEKDNFNRWEERDKHFLDVAGSNMD
FISLHLYDFPDFTDNGVPVKRYRKGANMEATLDMLNHYTDLSFGKEMPLIISEYGAQVHRLRNEPWFPYR
DWLTINSINSMMMAFMERPHQVEKALPFITVKAEWGRISPTVPYNFRLMRQQFEAEGESGEQWVYTELVK
LYELWKDVGGTRLDTATDDPDLRVDAYRSDTETFIIVNNLDFETQEFALNFRDGAAPAKIQTKQLFLGDQ
ELPLHDRTPMIENQTYSELPQTLTLESEATMVIRYSNTGNAPLGELQETNYYANTYKQPIESARPITFEV
DDINTSAEGFAILRVGLGREHGLSLQPEITVNGEPVTVPTDWRGGDQYLDGKGRESFFGVIEVPLPVELI
DVNNTVRIKFPDSGGYVSSLAIRYGASTTALLRPKTAF

>WP_049797727.1
MLADFEEAALPSWIQRDHIQAQITKAAGITSGNQAIELTFDSSSEYSTLSLQPAKPWDFSVLGDMNLAFD
ATNTSDVSVHLYLRVNDTHQMQTRSISIPAHSSATYYADLSGPQISLDSGLRADPPAWSSDEFRMPYMTG
NKLLDTSEIKEVSFYISSNIQDKQVILDNVRARSNPAIDPQYLVGLVDAFGQPAKSNFKDKVKSDSHLKQ
LAEEELADLAANPVLADRSKYGGWKNGPKLEATGFFRTEKVNGVWSLVDPEGYLFFSSGIANIRIANTTT
LTGVDFKDDQVRYIDPEDVTPEDSLGIRPVSARAQKTRYISSELRHNMFNWLPDYDDELANHYSYRRSTH
KGPLAHGETFSFYQANLERRYGERYPESFIDDWEDVTIKRMRSWGMTSFGNWVDPEFYHKNQFPYFANGW
IIGDFKTVTADGHGWSPMPDPYDPEFARRAKVTTQVIADEVKNNPWCIGVFIDNEKSWGNPASIESHYRI
PIQNFKMDASQSPAKAEFTKLLQDKYGDIATLNRAWETNIPSWDSFAKKFDVEQINDAMIEDMSVILETY
TSQYFEVVNSALREVMPNHLYMGVRMAAWSINPEGVRAAKKYVDVMSYNYYREGMHDSTWDILPQIDMPS
IIGEYHFGAMDTGLYHPGLIHSSDQKDRARGYQAYMRKVIDNPYMVGAHWFQYTDSPVTGRAYDGENYNV
GFVTNADVPYKEMVEAAREINQELYPRKFKDAVK

>WP_049721026.1
MTLKPLALAVAISAALGSACTSNNSVSLSDADRLINEQLEPQQLPTTLVDFSSAQQQGWLTAQQAELKPT
ASISTTALNIRFPSSVHMPKLNIEPNTPWDLSGLDNYNLAFEVNNLSPVSTHFYVELFDANGTSQSRELS
IPKGYQGKVFFPLTGEKAATDKGMWANPMPWPTDEMKMVWRSWHQTLDMSQITKISFYTIGVLQDRTLEI

GDIVLRPNPDAGAEWASNLVDRFGQAAKKNSPLKVLSEAELKAIAERELQQLTEQPGPSDRSRFGGYKDG
PKLEATGYFRTEKVDGKWWMVDPEGYLFFSHGPANVRMSNLTTLTGVDFKDPSVRVVQADEVTPEDSMGI
VTVSDEVRETRYVINETRHDMFEWLPSYDDSLADHYSYRRSTHKGPVPHGETYSFYRANLERRYGETEPE
SYVRKWEEVTLDRMNSWGFTSFGNWVDPAFYPNQQVPYFANGWIIGDYQTLSGHTNHWGLMPDFFDPVFA
ERARATIEVIAKDVQASPWCVGIFIDNEKSWGEREGSVEQRYGVILNALSKNANQSPAKQAFTERLQAQY
SSIKALNRAWNTDFSSWSEFAENASTEQHSDAQVVDLSKLLEALGEQYFKVVHGTLKEYLPHHLYMGARM
ANWGMPDEIIKASVKYSDVLSFNIYEEGMQTHQWDFLTEVDLPVVIGEFHIGATTDSDNYHPGIVSAASQ
KDRARMYKDYMQSVLEKDYMVGAHWFQYVDEPVTGRAFDGENANIGFVTVTDIPYPEMIKAAKEVNYNLY
PQRYGQ

>WP_049721010.1
MKLTVSLCLLLLSSLMLSCGQVKKDLPRERSLMTFSDPDSIDQIEPQQASISYSSLDGGAARVDMHAASH
HISGFTIKPSVPWDLSDFNNLALVFDIANPTDESVSMYVSTTDIHNDFQMRSFVVPAQSSQTYSIDIVVP
ELSQETGIRSNPESWNNPYQALIWRGGKKQIDTSAIAGVRFDVRGALSDKTLIVDNVRAVEPLEFNPDYL
KGLVDTFGQSAKVEFSHKIDSVDELRNVSDAEQKQLQHNTITDRSSFHGWRNGPKLAATGFYRADQYKGK
WTLVDPEGYLFFSNGIANVRMANTSTMTGYDFDHTLIPARNLGGFTPEDSLGLNPAPNQALSSRQISSPL
RAGMFNWLPSYDEPLGEHFGYRREVHSGALNRGETYSFYRANLARKYRTDDNQKLMQAWRETTVKRMIDW
GFTSFGNWVDPEFYEMKNYPYFANGWIIGDFKTVSSGNDYWGALPDPFDPKFRERAFVTVKKISDEVANS
PWCVGVFIDNEKSWGIMGSVESQYGIVINTLTRSVDDSPSKAAFVDYLKKTYSSINAFNSAWGTSLRDWE
MLENGLALTNYTGAALKDLAAMLELYSAEYFRIVDSTMDELMPNHLYMGARFADWAMTPEVRTAAARYVD
VMSYNYYREAVSDVFWDFLADIDKPSIIGEFHNGALDSGLLNPGLIHAESQQDRGVKYQEYVNSVIDNPY
FVGVHWFQYIDSPLTGRAYDGENYNVGFVSVTDIPYKPLVDSARSVNRALYPRRYGN

>WP_049720988.1
MKIKSLPAILSLIILSACSPQVTHTAFSQASSDDSLNTINHLYDFEHSTPPLALSTNNAQLALIPGPDGQ
QLKVTFLSHDHHEASIDFAPEKPWQWHKYPRFAFAIDIENPSENSAHLYAKAKDSSGKQHNRSFVVPAQS
RNTYYMELRGSDLTTDTGLRANPKEWITDAEPMIWRHADKVIDVSQISSVSFTVRGLLEDKTIHLDNVRL
VTPKALDPEYLTGLLDEFGQNDKIDTPYKVKTLVQMRDQAQAEAKELSQGAPADRSLFGGWLNGPKLEAT
GYFRTTKYQGKWSLVDPDGYLFYSNGIANVRMSNTSTLTGYDFDHTLLDERAGNDLTPEDSKGLNRVSDN
ALPSRQVVSPLRAGMFTSLPKYNDPLGAHFGYRRSVHSGPVARGETYSFYRANLERKYQTDTAPDYMATW
REVTVDRMLQWGFTSFGNWIDPEFYQMDRIPYFANGWIIGDFNTVSSGNDYWSPLPDPFDPIFAERAAAT
IERIAQEVDNNRWCVGVFIDNEKSWGAMGSIESQYGIVLNTLSRADTDSPTKAAFSQWLQQQYNDIEAFN
RAWGTNLTDWQALHNGITINVYSPAQLADFSQLLQLYAEQYFKVVHNTLERYMPDHLYMGARFADWGMTP
EIRRAAAKYADVVSYNYYKEGINQPYFDFLAELDKPSIIGEFHNGAADSGVFNPGLIHSESQTDRGEMYT
NYVTSAIENPYLVGTHWFQYLDSPITGRAYDGENYNVGFVSVADVPYSPLVNAAKRINNKLYTLRFK

>WP_049720970.1
MNVYKTITPFVLLFSLFLSACNTEEAPTVAASNEAAQEKADTDQPLAVLYNFDDALDSTIKSASANLDLV
GYGSDKAIKIDFLSKENGYSGITFKPEKPWDWSEFDSFSLSMDLANDGDQSTQIYLNLKDGNGNVATRSV
VVPRGDFKTYYAKLAGHDIEATGDDDATELNFSSGLRSNPPTWSGEDSHFIWMWGVKSLDVSQITEISLS
VQGAVSNKTISVDNVRLTPNPKMDEEFLVGIVDEFGQNAKYNYADKIDSLEELHQVRDAELAELDGKAMA
DRSKFSGWKSGPKLEATGYFRTEKVDGKWALVDPEGYLYFATGLDIIRLSNSTTMTGYDYDQDKINQRDE
HDLTPEDSIGMLKVSDEAKKTRFVASKTRADMFSWLPEMDGELANHYSYRRAAHSGPLKHGETFSFYQAN
LERKYGEEHPNSFLDQWEDVTKKRMLNWGFTSLGNWTDPHFYSNKQIPFFANGWIIGDFKKVSSGNDFWG
PLPDVFDPKFSERANVTAKQVAAEVQNTPWCVGVFIDNEMSFGRSESDQLRYGIVINTLGRDASTVPTKA
QFSQLMKAKYESIGAFNQAWNLKLTDWSEFDNGFAPGEITKAQRVDYADMLEHYATQYYRVVHDAVQEHL
PNHLYLGSRLPDWGMPIEVVRAAAKYADVVSYNVYKEGLNKKKWEFLAEIDKPSIIGEFHMGAMDRGLYH
PGLIHAEDQDDRAQMYLRYMDTVVENPYFVGAHWFQYMDSPLTGRAHDGENYNVGFVDVTDTPYPEMVEA
AKELGRNLYDERYGDK

>WP_049632135.1
MILCIISLFLIASCAEPKHKKGKNDKINLLEVKERILLDFENESDIKAIELQNARSKLIVNEGNHGLEIN
FDSLNNHDSSVYINPSNTWNFNDYESAALVLDIENSTKSSTHIYIYTYDKSGAFQLSNVAVPAESNNSYL
IELKVPSFLLNTGIRNDPPSWPHNYISTIWRGGTKTLDTSAITSIRLLISGVLENKQLIIDNLRVVKPNN
FDPEYLTGLVDEYGQNAKQDFAGKVYSADQLRSFSQQEQENLTDQQFPSRSTFNGWINGPKLKSTGYFRA
EKYQGKWSLVDPEGYLFFSNGIANIRMANTSTITGYDFDAALIKQRDAGDYTPEDSIGLNTAPKQAWPSR
HISSQLRADMFTWLPSYSEAAGANYGYRRSVHSGSLKKGETFSFYRANLARKYATQNEEALMQDWRDTTI

KRMHNWGFTSFGNWVDASFYQMNRLPYFANGWIIGDFKTVNSGNDYWGAMPDPFDPVFTQRTNKAIKKIA
DEVKNNPWCVGVFIDNEKSWGSMGSPSLQYGIVINGLKRNADDSPLKQEFINHLKNKYQNIENLNTAWDL
KHTSWAQLSQPVELTSYNAQMLGDFSELLYLYADAYFSRVDKALTKYMPNHLYMGPRFAHWAMTPEVLKA
AAKYVDVMSYNYYREGIDQPYWDFLAELDKPSIIGEFHNGAIDSGLLNPGLIHAESQFDRGEKYKSYLNS
VIENPYFVGAHWFQYIDSPLTGRAYDGENYNVGFVSVADIPYTPLVKAAQEVNKSLYIKRFGK

>WP_049629447.1
MKKNQLRLIKQMLAGTLAGLSLGCVANQNSPLGSIENFDSFSLNRAVANNVKASLITGQEEKYGKALKLV
FEPREESTIELPTPAQGYWNWEYAGDLNLALDVTNPGSQSFQFWLTLMDAKGRKQERSAVIDAGQSARFY
APLTGRVANAQTGMRETPPAWQTSEEKLAWRSGDRDFDFTQVTKIIFKAYAQFETNILIVDNLQLRVNPA
QDPEYLVGIVDKFGQAAKKDYPTKIHSEKQLKAAADAELASLANAKQPEDRSRFGGWSKGPKLEGTGYFR
TAKVDGRWWMVDPEGYLFFSSAIANVRMANLETITGYDFNDASVRKIDPEELTPEDSRDIIPVGKKSLNS
RFLASPLRREMFEWLPPYDHELGEHYGYRRTVHQGVIEHGEVYSFYKANLERRYGQSSPNSYIKTWRDVT
LDRMVDWGFTSFGNWIDPMFYDNQRMPFFANGWIIGDFKVIYSGQDYWSPLPDVYDPEFKRRAHLTIKQI
GREVKNTPWCVGVFVDNEKGWGSMKNDRAHFGAVYYALSRTSDESPAKKQFTQLLTKKYGDIATLNQAWG
TKIDSWSSFATGITMDKLNDASRADFSMLYADYAETYFRIVSSEIKDALPNHMYMGVRIAAEWGMPVEVV
AAAKKYSDVLSFNNYREGMHPDTWAFLKDLDFPTIIGEYHIGSTSDTDFYHPGLVIAADQTDRAKMYENY
MNSVIDNPYMVGAHWFQYIDDPVTGRAYDGENYNVGWVSNTDIPYQPMVDAAKRVNKSLYQRRSKIPPIQ

>WP_049629422.1
MSSSYKLDRSITFFLKTSVLSVAALFLIACQEEAADKGTAVSQVKSTPADKVLFDFEGEQLPAEISFFNA
EGSLVTTSPNNSSASQALKVKFHSLEHEYASLAIQPKSNTWNWSDIGDASLAFDIANDGEHSVQLFLDVS
DAKGNNFTRSVSVPVGKSRVYYSKLSGHDMVSANPDSKVELNAASGLRGNPPTWSGDDVQFIWMWGVMNL
DLSAIQRISLSVQYALHDKEITLDNIRIIKSPAMNKDFLVNLVDKFGQPAKVDFAGKIHSEAELQAATQS
ELKELNNGAPLADRSTFGGWKNGPKQPATGYFYPKKVDGKWWLVDPEGYLYFATGLDIIRLANAYTMTGY
DYDASTIEQRSADDLTPEDSKGKILISEEAQKTRHLVSKTRADMFEWLPKHTDPLGNHYDYNRDAHSGPL
LKGEAFSFYSANLERKYGETEPNSYLRQWEKVTVDRMLNWGFTSLGNWTDPKFYNNQRIPYFANGWIIGN
FKKVSSGNDFWGGLPDPFDPVFKERALATAKAIAEETKNSPWCVGVFIDNEKSWGRSESKESEYGIVLNT
LTRDGADSPTKNKFTQLMKEKYVDIAALNTAWGTSVESWDAFQKGVKTGINNDVQMQDFSLLFTQYAEEY
FKIVEGALTQYMPNHLYLGVRFADWGMPKDVVKAAAKYADVVSYNFYKEGLTKNKWTFLAELDKPSIIGE
FHIGTTESGLFHPGLVHAANQEDRAKMYKEYMETVVDNPYFIGAHWFQYMDSPVTGRSYDGENYNVGFVS
VTDTPYAPMVKAAKELHGEMYTRRAKK

>WP_049629440.1
MKLTQLISIATYTLILSGCGGGGGGGGGGGTSSPPASSKASVSSIQSSIAVSSSVASSNTSISSTLNSSAA
SADSRPKISFNRDIKHIVGDIDSFDRRKFITIHSSNTEADWFGSNAQSLGAPNASPDLITEVMEGYDVYF
GRDTGGITWQLGELNQDPARPGFISESHAQTKGGDARWVYSNNANSAKIRQFENRLTDMIIGAQQHPFWP
DGKLTRKGWALSQTDTPSEPFGTATGHYMGQYLAEYFNKGAGDLYGQPKPLYVEVMNEPLYDLVDASASP
VPVEKVFQFHNTVAAEIRKTNSDVLIGGYTVAFPDYDKNNFQQWLNRDKVFIDMAGANMDFYSIHLYDFP
AHNNREKYRRGSNVEATLDLLEQYDTIKFGKIKPLVISEYGASVHTMFSDPWTPQRDGLRLIALNGLLMS
FIERPEHIAKTIPFIPIKAEWGRLNGIPYNDRLMRQRKEAPGETGDEWVFTDMIKFYQLWSDVKGTRIDI
SSSDPDLVVNAYADNQQVHLIINNLEFNAQEFTLNDYSLNNPAFTHATIKHFHLDSQNVPVLSETVSDKF
PSALSVGANGTMIITMHYESAVVIDQTKVEQKTYATNYLQAIKTNSPLNFAISGITPAAFGEATLRLGIG
RDHGKSLQPKVTINGTEVTVPKDYRGYDQYHQGKGRENFFGVIEIPVPYSILKANNQIEVTFADDGGHIS
SAAIRAFHYSRNFTH

>WP_048688993.1
MKLNSIKAIAPIILTLAISACSNPSTSQHSHLSLIDFEDETDKHIILLENADAKMIQSSQGNALEIKLES
KNNHDASFTVQPKTPWNFSQYQNAAIALDIKNPSATSTHVYIYTYDANNAFQLRNVVIPANSNNSYLIEL
KSLGLQVNSGIRNNPPSWQHDYVQTIWRGGVKQPDTSAISKIRFLISGVLEDKTLEIDNIKVVEPKQYDE
NFLTGIVDKFGQNAKQKFVRKVHSVAELQKFSQQEQQSLVDHPLPSRSKFNGWADGPKLKATGFYRTEKY
QGKWSIVDPEGYLFFSNGIANIRMANTSTITGYDFDKKLIKQRDAKDYTPEDSIGLNRAPDNAISSRYVS
SETRANMFTWLPSYQSDEAASFGYRREVHSGAIKHGETYSFYRANLARKYETNDEALLMEKWRDTTIKRM
HTWGFTSFGNWVDPSYYQLNRLPYFANGWIIGDFKTVSSGNDYWGAMPDVFDPVFATRAEVTVKQIADEV
QNNPWCVGVFIDNEKSWGADWGATAQFGIAINAFTKQASQSPIKQNFVATLKAKYQNIEALNQAWNSQFA
SWQTLEAPYTINKITEPMKQDLSTLSYNYADKYFSVVAELLEQYMPNHMYMGPRFAHWAMTPESRKAAAK
WLDVVSYNYYREGIDQPYWQILEELDKPSIIGEFHNGAMDSGLFNPGLIHATNQTDRGKKYQEYMYSVID

NPYFVGAHWFQYIDSPLTGRAYDGENYNVGFVNVADIPYQPLVDAAKELNENIYQRRYGL

>WP_048688578.1
MKLKKRYLASLVAVLTSQLSGCNNLNNQEKESQSSQVKMAEVVLFDFEKGQVPDGFEFWNAASSVTDKTS
AIDGNHSLLVKVNTLDNNSAGMRIKPEKAFDWSEYQDFNLAFDLTNAGTESLQIDLTISDENGGFYTRGL
VVPVGKTQTFYAKMDGHDQQDPPWATQTEFNFASGLRSNPVTWQSDDKQVYSFWGKKRLDISAITAINFS
ANGLLSDRQFTVDNIRLRANPKMDENFLVGLIDEFGQNAKVDYPNKVKSDAHLKQMAAEELASLNAELMD
DRTKFHGWKSGPRFAATGYFRTEKIEGKWALIDPEGYLYFSSGIDIIRLSNSSTLTGYDFDQNLVEKRTA
DMTVAEDDQPLNPVSKQAQATRYVANQTRKDLFNWLPDYNDSLGNHYGYRRETQSGPLKHGETFSFYSAN
LERRYGETYPESYLDTWRQVTLRRMLDWGYTSLGNWSESSYYGNNQIPFVAFADIIGDFGTLSSGFDFWH
GVPDAYDPKFKQRAVAAAKHVAAQINATPWCMGVFLDNEQSFGRGGSDESRYGIVLNTLTRDAKEVHAKA
AFTQSLKNKYKTISALNKAWQKDIASWQAFAAGIDASFNTEQQKQDYSELLYQYGVQYFGTVNAALKSVL
PNHLYLGSRLPVWGMPLEIVKAAAENSDVITYNLYEEGLVKGQWDFLAEVDAPSLIGEFSFGADDAGHVH
PGIVISADQKDRAEQMKKYMHSIIDNPYFVGVHMFQYADGPITGRAYDGENYNTGIVRVTDVPYEHMVEA
AKDVHQNLYQRRYGYLLKD

>KMT67009.1
MALGCSENSQQKTESSQSQLNQTYLYQFDLATELNDFLEENTQLSLIPETAAIENATSKRLSVTFLAKEN
YKSSLTIQPKKPWNWQQDKAFYLALDIENPTQDSTHIFGQVIDNKKQTHTRSVVIPKQSKHTFYIELKGE
DLNQETGIRSNPASWQSKDKPFIWRWGVKNLDLTQIVKVKFSVTSLLKDKTIILDNIRLVDSPEMDPNYL
ADLVDKYGQSTRVDYPQKVETDDVLQKVSQAELEQLDGKLMPDRSKFGGWKFGPKLEATGYFRAEKLGNT
WTMVDPEGYLFYSHGIANVRMANTTTITGRDYKKPIPQTAASDVTPEDSKGVIRASDDVLKTVYVASELR
HKMFQWLPEYDDSLAKHFGYRRSVHTGALEKGETFSFYQANLERKYGDDFIPKWRDVTVDRMINWGFTSF
GNWIDPMFYQLDRFPYFANGWIIGDFKKVSSGADYWSPLPDPFDPEFSKRAKATVDVIAKEVQQNPWCVG
IFIDNEKSWGSTSSLQAQYGIVIHTLNRTATESPTKAEFVKLMQTKYQTIEQLNQSWNTKISSWNSFATG
FKTQDLESSANMIADYSAMLSHYAQAYFDVVNKALKQQLPNHMYMGARFADWGMTAEVVEAAAKYADVVS
YNFYKEGLQDKHWAFLEQIDKPSIIGEFHMGATDTGLLNPGLVHAQSQQDRADMYTEYMQSVLKNNYFVG
AHWFQYTDSPLTGRAYDGENYNVGYVSVTDTPYKEMVEATKKITRDLYSNKYGHLK

>KMT66960.1
MKLNSIKAIAPIILTLAISACSNPSTSQHSHLSLIDFEDETDKHIILLENADAKMIQSSQGNALEIKLES
KNNHDASFTVQPKTPWNFSQYQNAAIALDIKNPSATSTHVYIYTYDANNAFQLRNVVIPANSNNSYLIEL
KSLGLQVNSGIRNNPPSWQHDYVQTIWRGGVKQPDTSAISKIRFLISGVLEDKTLEIDNIKVVEPKQYDE
NFLTGIVDKFGQNAKQKFVRKVHSVAELQKFSQQEQQSLVDHPLPSRSKFNGWADGPKLKATGFYRTEKY
QGKWSIVDPEGYLFFSNGIANIRMANTSTITGYDFDKKLIKQRDAKDYTPEDSIGLNRAPDNAISSRYVS
SETRANMFTWLPSYQSDEAASFGYRREVHSGAIKHGETYSFYRANLARKYETNDEALLMEKWRDTTIKRM
HTWGFTSFGNWVDPSYYQLNRLPYFANGWIIGDFKTVSSGNDYWGAMPDVFDPVFATRAEVTVKQIADEV
QNNPWCVGVFIDNEKSWGADWGATAQFGIAINAFTKQASQSPIKQNFVATLKAKYQNIEALNQAWNSQFA
SWQTLEAPYTINKITEPMKQDLSTLSYNYADKYFSVVAELLEQYMPNHMYMGPRFAHWAMTPESRKAAAK
WLDVVSYNYYREGIDQPYWQILEELDKPSIIGEFHNGAMDSGLFNPGLIHATNQTDRGKKYQEYMYSVID
NPYFVGAHWFQYIDSPLTGRAYDGENYNVGFVNVADIPYQPLVDAAKELNENIYQRRYGL

>KMT66938.1
MRSKMRKLPIAAVLSAILLTACDNNSSQTQNPALTTDTPLAVIWNGSKSSGVEIANWGAQTQLEDKSVKV
SYQSDHYRPSVKFHPEKPWDWSKFDEFNLAVDIKNLSNESIQVYLSLTDEAGVKEDMEITGSRARSLNRT
ANLAAGEQGTYFSIIKGKFNDTNAGVREKPYPWKTDEEMFVTRFGKKLDLSRITEVALFVRGNLHDKQIE
VSNLRLRKNPEYDDSYLLGLADEFGQNAKQDFPIKVSSVEELKQVANKELAELAQGGLMANRSKYGGWKD
GPKLKATGYFRTEKIDGRWWMVDPEGHIFFSHGPANVRMANLTTLTGVDFKDDSVRYIDPSRVTPEDSMG
IVEVSDEVRKTRYIASDLRNDMFNWLPSYDNPMAKHYSYRREVHKGPLTSGETFSFYRANLERRYGENFE
ETWQQVTLDRMNNWGFTSFGNWVDPAFYPNEQVPYFANGWIIGDFKTLKSEHDVWAPLPDPFDPEFVRRA
KITIDVIAEEIKGSPWCAGIFVDNEKSWGLPEGTVEQKYGVILATLTVNNADSPAKTFFTDYLKGKYGDI
KKLNSAWNFKAENWASIEQGVHLEAPFSTEAVADLSYMLEALSDRYFKVVHDTLAEALPNHLYMGARMAN
FGMPKETIKASVKYSDVLSFNIYEEGVQPEEWGFLTEFDLPVAIGEFHIGSTTDTGVFHAGLVQAHDHKD
RAQMYLDYITSVAAHPNMVGAHWFQYIDSPLTGRAFDGEPYNVGFVSSTDIPYPEMVDAIKSFMSTVYEK
RFKGEFDAPAYKK

>KMT66819.1

MKLKKRYLASLVAVLTSQLSGCNNLNNQEKESQSSQVKMAEVVLFDFEKGQVPDGFEFWNAASSVTDKTS
AIDGNHSLLVKVNTLDNNSAGMRIKPEKAFDWSEYQDFNLAFDLTNAGTESLQIDLTISDENGGFYTRGL
VVPVGKTQTFYAKMDGHDQQDPPWATQTEFNFASGLRSNPVTWQSDDKQVYSFWGKKRLDISAITAINFS
ANGLLSDRQFTVDNIRLRANPKMDENFLVGLIDEFGQNAKVDYPNKVKSDAHLKQMAAEELASLNAELMD
DRTKFHGWKSGPRFAATGYFRTEKIEGKWALIDPEGYLYFSSGIDIIRLSNSSTLTGYDFDQNLVEKRTA
DMTVAEDDQPLNPVSKQAQATRYVANQTRKDLFNWLPDYNDSLGNHYGYRRETQSGPLKHGETFSFYSAN
LERRYGETYPESYLDTWRQVTLRRMLDWGYTSLGNWSESSYYGNNQIPFVAFADIIGDFGTLSSGFDFWH
GVPDAYDPKFKQRAVAAAKHVAAQINATPWCMGVFLDNEQSFGRGGSDESRYGIVLNTLTRDAKEVHAKA
AFTQSLKNKYKTISALNKAWQKDIASWQAFAAGIDASFNTEQQKQDYSELLYQYGVQYFGTVNAALKSVL
PNHLYLGSRLPVWGMPLEIVKAAAENSDVITYNLYEEGLVKGQWDFLAEVDAPSLIGEFSFGADDAGHVH
PGIVISADQKDRAEQMKKYMHSIIDNPYFVGVHMFQYADGPITGRAYDGENYNTGIVRVTDVPYEHMVEA
AKDVHQNLYQRRYGYLLKD

>KMT63824.1
MKGPRCNLAKLRQVIQISAFSILSTALVACQSSETTAKVEKEIKKEQILFDFENTSIPLQVKTANAKTEL
TDQNGSKALNVKFNSAKHEWTSVDFVPENGVWDWSKYKSFSIAFDISNQGQYSTQLFLNVYDKNGQVYTR
SVNVPVGDAKTYYSKMAGHDLGSPNNAHSEKVELNLSSGLRSNPETWDTDDVQFIWMWGNKNLDTSGIKR
ISLSVQYNLHDKEVTIDNVRLMPHPKMNPDYLKHIVDKFGQPTRLEYEEKIHSMDELLAVKDKEIKRLNN
GEKIADRSKFSGWKSGPKLEATGYFRTEKVGEKWSLVDPEGYLYFTTGIDNVRMSNATTLTGYDFDQAAI
NKRTSSDVTPEDSQGLNPPPKSATGTRHLVSETRADMFEWLPEDYDHPLANNFGYRRSAHSGPLQKGEVF
SFYMANLERKYGETTPYSYLNDWSKVTVDRMLSWGFTSFGNWIDPMYYDNDRIPYFANGWIIGDFKTVSS
GNDFWGGLPDVFDPLFAERAEVTIKQIAKEVDNSPWCVGVFIDNEKSWGRPESKVTELGIVIHTLTRDGK
QSPTKNMFTEKMKAKYGDISKLNQAWNLSIPSWDIFQKGGFNSKLDTNNKAQIADYSQLLYEYASEYFRI
VDTTLEKYLPNHLYMGVRFADWGMPMPVVQASKKYVDVVSFNLYKEGLTPKKWKFLEELDMPTVIGEFHM
GTTASGFFHPGLIHAATQEDRARMYKDYMHSIIDNNYFIGAHWFQYLDSPITGRAYDGENYNVGFVSVTD
IPYNAMVKAARELHTELYERRFGDLKQEK

>KMT63726.1
MNKILTSTLIAASLLSACGEKNHTHVQQESSEKSWSISNFEQGLPTEFDAGNTKSSLVNKNAPEGKQALR
IDFSSDELRSDFSFKAKKPWDWSQYGNINLAADIHNLSDESIQIYLEIKDATGWPHIRSVSIPAGYTGTY
YALLKGPQLELDSGLREDPKTWQSNDHKMFWMRGAKQLKLDQISSINFFVESMKNDKSLLIDNLRVRQNP
DVDANYLKDLTDAYGQSYKFEYPTKVKSDEQLKALADAEIAQLNQQGTMADRSQFGGWASGPKLKATGYF
RTEKVNGKWAIVDPEGHLFFSSALANIRIANTTTFTGVDFKDDSVRYIDPEDVTPEDSLGIRPVSKEAQK
TRYVSSEMRHKMFTWLPDYDHDLANHYSYRRSSHKGPLSHGETYSFYQANLERRYGEDYPDSYLDAWRDI
TIKRFKNWGFTSTGNWTDASFYQMNQVPYFANGWIIGDFKTVSSGQDVWSRMPDPFDPEFKRRAIITAKV
IGEEIKNNPWCIGIFVDNEKSWGNNSSLQKRYGIVLNTLTREDSDSPLKAKISQMMQAKYPSIKALNTIW
KTDIESWASFSKGISITDLNAGVVADLADMSFAYANEYFKIVHDALADVAPNHMYMGVRMAAWGLTTEAA
NAAAQYADIMSYNFYREYAHPKAWEFLADLDKPSLIGEFHMGATSDTGLYHPGLILAADQQDRAQMYKDY
MYSIIDNPYMVGAHWFQYLDSPLTGRAHDGENYNTGFVTITDVPYQEMVNAAKEVNANLYQRKFGNGQQN

>KJZ28757.1
MLCCLIGLAGCNNGKISEKKPNNTVVDFKKQLVIFDFEKNNYSDNINTINASTKLVEQSGNHKLQVNLYS
KSHTESDFEFVNPQGWDWQAIGNFALAIDIQNPNSASTHIYVKTTDKSGKSQTRSVVVPGHSENTYYIEL
KGANLNINSGIRSNPPSWHSGYTPIIYRGGQKNIDVSSIVKVSLGVKGLLEDKRFLIDNLRLIKPTNFDV
NYLKGLVDEFGQNAKLNFINKVTSTEQLLVISQKEQAQLQAQPLQGRSKFSGWKNGPQLKATGYFRTEKY
KGKWSLVDPEGYLYFSTGIDNVRMANTSTITGYDFDQSYIKQREAGDLTPEDSLGLNPAPVAAWPTRYQS
SKLRAEMFNWLPKQDDPLADNYGYRREVHSGAVKKGETFSFYRANLERKYQTHNNNELMKQWQKTTVNRM
LSWGFTSFGNWIEEQYYHTKKLPYFANAWIIGNFKTVSSGNDYWSPLPDPFDPLFVERADITLAKVAQQV
KNSPWCVGVFIDNEKSWGMMNSDTTRYGIAINTLKNNAKNSPTKAEFVLLMKNKYSKISKLNKAWNTTLS
SWDEFAEGVTLTQFNDHVNTDLSAMLFHYANQYFAVVDEAITKHLPNQLNMGARFADWGMTPEIRAASAA
HVDVMSYNYYREGLNQEFWEFLSDIDMPSIIGEFHNGALDSGLLNPGLIHTQSQQERGSKYQNYMNSVID
NPYFVGAHWFQYIDSPLTGRAYDGENYNVGFVNVADIPYEPLVNAAKNVNKSLYERRYRNDKN

>KJZ28647.1
MKKRSLYCTVSSALLLSSTFNVHAVKVQFNIDTKHAVGQFDSFDRRKFITLHSSNTESDWFGNNAQSLNA
PNADPDLMTHFLEDYDVYFGRDTGGMKYQLTQLPEDSAKPGYASAATATTNGGGVKWNYTNITTAQAKTM
RKHEGRNSDLIVAAQQHPYYPDGTKIGNQNWSFSQRDTAAEPLGTAMGDYMAQFLQKYFKSGPSDSLGQK

RPTYVEVMNEPLFELHDFPHTGYDKESLYDIFRLHNSVADVINANPALNDVKVGGFTVAFPDYEKGSQFF
DNWKQRDKAFLDIAGNKMDFISMHLYDFPNFPGGPGGQHQIQYRKGSNMEATLDMVEQYMAYKWGSIKPL
VISEYGSQLQGSFGTKWTPQRDWLCLKAMSSMLMSFMERPNRIDKAIPFIPLKAEWGRISDTIPYYWRLL
RQAKEGQGETGEQWVYTEMVKFYQLWSDIKGTRIDSWASDMDIQADAYVDGKDVYLVLNSLEFSPTNIDL
SVLGKGSNNVTGVNIKHLYPNAQGKPVLDNSNRTTLPSSVVLGSESTMIIKVSHQKNVVINNINQETKHY
ASAVVKNIAANATQFFTINNVDKGANGEATLRLGVGRPHGKSLTPVVTVNGNRVAIPTDFRGYDQKNGGL
GRERFFGVIEIPVPYNNIKKTNNIEVTFPDSGGAVSSLTLQNFKMSKRITR

>KJZ28311.1
MTYKKNILLTSLLLALSACNQEDVKVDRPTTDAVQIKSTNTLMYLFADDTHHTATSVDYQSNSAIVKNEN
SVLNVQFQSKKNSYASIVFSPEKPWDWSEFNDFNLAFELANPGTHSVQIYLDISDIDGANYTRSVNVPVG
GYNTYYAKLDGHDLATPDGKENVELNFTSGLRSNPDTWESDEVQFISMWGKKNLNLKGIAKIAISVQSTL
HDKELAIKSISLRKNPQFNTAFLTKIVDEFGQNAKQEFAGKVHSEAELLSDKKQEATQLLSKRPTNRSRF
GGWAKGPKLEATGYFRTAKYNDKWSLVDPDGYLYLATGIDIIRLANSTTLTGYDFDQALLAKPADAGVTP
EDSKGLNQVNKEALKSRFVASQVRKNLFEWLPDYSDPLGKHFGYRKSAHSGPLEHGETYSFYAANLERKY
GQNNADYMQKWREVTLDRMITWGFSSLGNWTDPSYYDNQKVPYFANGWIIGDFKTVSSGNDFWGAMPDVF
DPEFTVRANETVSVVAKEVKNSPWAVGVFIDNEKSFGRPDSVKSHYGIVINTLGRDAKTVPTKAEFSRLM
KEKYTDVAKLNKVWHLNLASWVEFDKGVTVDIKNEEQLVDFSILLTAYADKYFSVVNAAMDKYLPNHMYL
GARFPDWGMPIEVVKASAKYVDVISFNAYKEGLRDDKWAFLSQFDKPAIIGEFHVGSSDSGLFHPGLIHA
ANQQDRANMYTDYMNSVIDNPYFIGAHWFQYIDSPITGRAYDGENYNVGFISVTDRPYIEMIEAAKAMNE
SMYERRFKK

>WP_044620689.1
MSTSIIEPTQKVPYLKEHRLLNFSRESLPNELMFLHTDAFITPVGENQYGVRITSRAKDNFYTATNLVPE
MPWDWSQLPNFSFAFDASNLANHSTQVFINIFNTQDEMHSRSITIPACRNPRTYICELKGDYLQGKTNHY
SGLRSNPAPFDTPYEYATWMWGAINIDLTSICKIELSIHGSLLDHELVLSNFRLIQSPPTNPDYLTGIID
KFGQNANVDYVQKVHSESQLLALKDAELETLKAGKMPQRSTFSGYTGAKQFEATGYFRTLKIDGKWSLID
PEGYPYFATGIDIIRLANAYTMTGVDYDHSKIEQRQADDLTPEDSIEKVTVSSEAKQTAFIASDMRRNCF
NWLPSYDDPLAEHYGYMRELWEGPTAQGETFSFYAANLQRKYGENYRQIWSDVTIDRMLNWGFTCLGNWA
APEFYRNEKIPFFANGWIIGDFKTVSSGDDFWTALPDPFDPQFEQRAIATVQQVKAEIQGSPWCVGIFID
NEKSWGRMGTIQGQYGIPIHTLSRDAVESPTKAVFMTVLQEKYQTIEALNQAWSTQFTSWEIVAKGVCDL
AHNEAQCEDYALLLETYASEYFRIVSQAVKAELPNHLYLGARFADWGMTPEVVRACAKHADVVSYNYYKE
GLHPEPWTFLEEIDMPSIIGEFHFGARDTGFFHPGLVSAESQDERGEMYERYVQSVVDNPYFIGCHYFQY
IDSPITGRSFDGENYNIGFVSVTDVPYDGMVNAAKTINSSLYQQRFNQLTK

>WP_043317017.1
MKLPIKQCSTAIFLASLISGCGNDVSSTAVTPNTVNIDAPAAASSDTQSLWTFDQGQLPSAIQLENADAR
VIAGESGKALELQLRTKSHYSANITFAAEKPWDWSGLGNFAFALDITNPKQSSVHLYVKAADKHGKVQSR
SFAVPENSSGTYYMELKGPDLTVDTGIRSNPPSWDSEFQDMIYRGGVKQIDVSAVKSVALSVIGVLEDKT
LVIDNVRLIQPKSLDESYLKDLVDEFGQNNKLDFASKVDSLEELRAISEEEQSQLRKTPMDGRSRFGGWA
EGPKLEATGYFRTEKVDGKWALVDPEGHLFFSTGIANVRLANTSTITGYDFDKARIPQRTPGDLTPEDSL
GLNRVPDTAIPTRHISSPLRADMFTWLPEYNEPLGQNFGYRREVHTGVIEHGETFSFYRANLQRKYDIAD
EERLMAKWRETTIDRMLSWGFTSFGNWIDPAYYQMNRIPYFANGWIIGNFKTVSSGNDYWSPLPDPFDPL
FKERAYITAEQIGKEVANNPWCVGVFVDNEKSWGQEGSTASQYGIVINTLGRAAGESPTKAQFVQLMQDK
YGEIGKLNTAWNIQLADWDAFANGVALTEFSDAMIEDFSTMLEHYTGQYFKIVREAIKHFMPNHMYLGAR
FADWGMTPEVRRAAAKYADVVSYNYYKEGVSNKFWSFLEEIDRPSIIGEFHNGSLDSGLLSPGLIHASSQ
ADRGKKFAEYMNSVIDNPYFVGAHWFQYIDSPLTGRAYDGENYNVGFVSVTDIPYQPLVDAVKEVNENLY
QRRFGEAKLAVAPE

>WP_043316972.1
MDTKRFSKLLMRSALSLAITATTLGGLAACSGEQKGIKPKADETAASVAAASDSNLLAQDFLLEGFDQGG
IPASVQVNNGTANLVDDGAGGKALQVKLNLANNNSAGLIIQPAEAWDWSEYSDFNLAFDVANHGEESVQI
DVTMGDKNGDFYTRGLVVPADGLSRTYYAKLHGHDQEDPEAAAQNEFNFASGLRSNPPTWQSDDIMLHSF
WGKKLLDLSGITQISFGTDGSLSDRQYTIDNLRLRANPPMDENFLTGLLDKYGQNAKVDYEGKIHSDEEL
KKVVEEELASLSGKPNADRSKFSGWKSGPQLEATGYFRTEKFNGKWAIVDPEGYLYFSTGIDIIRLSNSS
TITGYDYDQKLIPKRSADEVIAEDDQPLNRVDKAAWATRTLVSETRAKMFNWLPGYDDELGNHYGYRRET
QSGPLKHGETFSFYSANLERRYGETYPESYLDTWQKVTVDRMLDWGFTSLGNWAAEPFYEQERIPYVAFA

DIIGEFGTLSSGFDFWHPVPDPYDPRFYQRSVVAAKSVSEQVENSPWCMGVFFDNEQSFGRLESDELHYG
IVINTLTRDAADTPAKGAFTKVLREKYGTIEALNKAWNKEVKSWEAFEKGMDSSLTTDAQREDYATLLFE
YGNQYFGTIRKAMKSVMPNHLYLGSRLPSWGMPPEIVKAAGKNVDIISYNLYEEGLVPSKWEFLAEIDKP
SLIGEFSFGSDDQGHFHPGIVISADQKDRGRMFKNYMHSFIDNPWFVGVHMFQYMDSPITGRAYDGENYA
NGFVSVADVPYVELVKAAKEVHEDLYERRFGDVKPVE

>WP_041522724.1
MTLKPFALAVAISAALGSACTSHNASTSNPAQQASAQLAAQQLPATLVDVTRAQQQGWLTAKQAELKLTS
DPVLNIRFPASVHTPAFNIEPETPWDLSNLDNYNLAFEVNNLSPVSTHFYVELFDTSGSSQSRELSIPKG
YRGKVFFPLAGEKAATDKGMWANPMPWPSDEMKMVWRSWHQTLDMSQISKISVYTIGVLQDRTLEIGNIV
LRPNPDAGADWTTHLVDRFGQAAKKHSPLKVESEAELKALAQRELKQLAEQPGPADRSRFGGYKDGPKLE
ATGYFRTEKVDGKWWMVDPDGYLFFSHGPANVRMSNLTTLTGVDFKDPSVRVVHADEVTPEDSMGIIKVS
DEVRESRYVINETRHDMFEWLPGYDDPLADHYSYRRSTHKGPVPHGETYSFYRANLERRYGETAPESYVK
KWEEVTLDRMNSWGFTSFGNWVDPAFYPNQQVPYFANGWIIGDYQTLSGHTNHWGLMPDFFDPVFAERAR
ATIEVIAKDVQASPWCVGIFIDNEKSWGEREGSVAERYGVILDALSKNANQSPAKQAFTQRLQAQYSSIN
ALNRAWNTDFSSWSEFAENASTEQHSEAQVADLSTLLEALGEQYFEVVHGTLKEYLPNHLYMGARMANWG
MPDEIIKASVKYTDVLSFNIYEEGMQTHQWAFLEDIDLPVVIGEFHIGATTDSDNYHPGIVSAASQKDRA
RMYKAYMESVLEKDYMVGAHWFQYVDEPVTGRAFDGENANIGFVTVTDIPYPEMIKAAKDINYNLYPKRY
GQ

>WP_041522690.1
MTIKTLPAIISFMLLSACSQQATQPPGDDSLTTLYSLYDFEQSSAPASLLSENAQTNLIAGPDGRQLKVT
FLSQQHHEASIEFSPTTPWQWGQYPAFGFAMDIENPSDESIHFYAKTKDGDGKQHNRSFAVPANSRNTYY
MELRGADLTTDTGLRANPKEWVTDAEPMIWRHSDKIIDVDNVTSISFTVRGLLQDKSVNIDNVRLVAPKS
LDADYLTGLLDEFGQNDKIDTPYKVESTEQMRRQAKAEAAQLSQGAPTDRSRFGGWLEGPKLEATGHFRT
TKHKGKWSLVDPDGYLFFSNGIANVRMSNTSTLTGYDFDHTLVDERSSNDLTPEDSKGLNRVSDNALPTR
HVVSPVRADMFTWLPGYEEPLGQHYGYRRSVHSGPVERGETYSFYRANLERKYKTDAAPDYMETWRNVTV
DRMLQWGFTSFGNWIDPAFYQMDRIPYFANGWIIGDFKTVSSGNDYWSPLPDPFDPFFAERAEATVKHVA
QEVNNNPWCVGVFIDNEKSWGAMGSVESQYGIVLNTLTRRDTDSPTKAAFSRWLKQRYGTVAALNQAWAT
DLANWQSVSDGITTTEYTDAQLTDFSELLQLYAEKYFKVVHDTLEQHMPDYLYMGARFADWGMTPEIRRA
AAKHADVVSYNYYKEGINEPFFGFLAELDKPSIIGEFHNGATDSGVFNPGLIHSQSQADRGEMYVDYVIS
AVENPYLVGTHWFQYLDSPITGRAYDGENYNVGFVSVADVPYSPLVKAAQRVNKTLYTRRFK

>WP_041522670.1
MNVYKTITPSVLLFSLFLSACNTEETPAAAASTEAAVEKSPGADQPLAMLYNFDDALDSTVKSASANLTL
VGDGSDKSVKIDFLSKENGYSGITFKPESPWDWSKFDSFSLSMDLANDGEQSTQIYLNLKDGKGNVATRS
VVVPRGDFKTYYAKLAGHDIEAAGDDDATELNFSSGLRSNPPTWNSADTHFIWMWGVKSLDVSQITEISL
SVQGAVSNKTISIDNVRLTSNPKMDEEFLVGIVDEFGQNAKYDYADKIDSLEELHQVRDSELAELDGKSM
ADRSRFSGWKDGPKLEATGYFRTEKVDGKWALVDPEGYLYFATGLDIIRLSNSTTMTGYDYDQKKINQRD
ENDLTPEDSIGMLKVSDAAKKTRFVASKTRADMFSWLPEMDGELANHYSYRRAAHSGPLEHGETFSFYQA
NLERKYGEEYPNSFLDKWEDVTKKRMLNWGFTSLGNWTDPRFYSNEQIPFFANGWIIGDFKKVSSGNDFW
GPLPDVFDPKFAERANVTAKQVADEVKNTPWCVGVFIDNEMSFGRPESDQLRYGIVINTLGRDASNVPTK
AQFSQLMKAKYESIGAFNQAWDLQLTDWAEFDKGFAPGEITEAQRVDYAAMLEHYATQYYRVVHDAVQEH
LPNHLYLGSRLPDWGMPIEVVRAAAKYADVVSYNVYKEGLNKKKWAFLAEIDKPSIIGEFHMGAMDRGLY
HPGLIHAEDQDDRAQMYLRYMDTVVENPYFVGAHWFQYMDSPLTGRAHDGENYNVGFVDVTDTPYPEMVE
AAKELGRDLYDERYGDE

>WP_039995382.1
MNNIRRYRLAATIAVTVATYLAGCSVSEVSKEQIEKIQYKEHEVLEMLYSFDGDTIPDDINFVDATGTLV
SDGSDNSNALRIKMNSSESKYTSVLIQPEKPWDWSNYNDFSIAMDISNQGNVSTQVYFDVSDMDGGNYTR
SVAIPVSELSDGKTYYGKMRGHDLGTPEGDINVELNFESGLRGNPPTWQGYNDTQFISMWGSKNLNTKAI
TQFSLSVQSALRAKEITIDNIRLIKNPPKQQDFLVGLIDKFGQNSTVEFPDKVHDYAELIDQRDAELKSL
QNGKKLNSRSKYSGWIDGPKLKASGYFRTEKVNGKWSLVTPDGYLFWSSGIANIRLANTTTLTGYDFDQN
YIVQREPGDLTPEDSIGMNRASDQAVKTRFVQSKLRADMFLDLPEYDEPMGAHYGYRREAHSGPLERGEV
YSFYRANLDRKYSEMGDFEEMWHKVTVDRMLNWGFTSFGNWIAPALYDNNRIPYFANGWIIGNFKTVSSG
NDFWRPLPDVFDPEFKERAEITVKQVAAEVKGNPWCVGVFIDNEMSFGRPDRIETRLGIMLHTLRRDGSE
VPTKAKFTNMMKDKYGTIDALNNAWDKSIASWEEFDKGINSDLNNDIQIADYSDMLHAYANQYFAVVSNA

VDKFMPNHMYLGSRFPDWGMPIEVVRAAAKHADVVSYNSYKEGLTKKTWEFLAEIDMPSIIGEYHIGSTD
SGLYHPGLIHGADQEDRGRMFKDYIYSAIDNPYFVGAHWFQYLDSPITGRAHDGENYNVGFVRTTDVPYK
PLVKAAQEVHKELYERRFGKYKE

>WP_039987978.1
MKNPQLTKKVSFALSAIAFAIALNGCSHDQQGLAKTSSTTKNTPTKNSLTDAFAKEQRIASLIDFDDQQQ
LSWLHESDISLTQITSSDSLVNKQVAVEFAKTGNISTLRVEPPKPWDLSQYENYNIAFDVENTSAESIHL
YLSLENPNGEVQSHSISLAKGYKGTVYFPLDGIEAETDSGMWGDVPPWTSKDDLMVWRSWRNAEQNYDVV
SALNFFTIGILENKSVILDDVRLRANPAHDPNVMVGLIDKYGQNAKQSTPLDVHSDAQLKQQAQEELAEL
AKSSGMPDRSRFGGYTKAPKREATGFFRTEKVDGKWWMVDPEGYLFFSHGPANVRMANMSTLTGIDYDQP
SIRERTSDEITPEDSMGIVSVPQSAKEKRYVISKARNDMFEWLPSYDDELSEHYSYRRSTHKGPIPYGET
YSFYRANLERRYGDDGVSAGSDPSYVKKWHDVTAQRMHDWGFTSFGNWVDPAFYQSEQVPYFANGWIIGD
YQTLSGHTNHWGLMPDPFDPVFAQRAKITIDAIAENIQASPWCAGIFIDNEKSWGEREGTVSQRYGVILD
ALSKNTQQSPAKKAFTAHLQQKYQTIEGLNSAWSSDFNNWQTFEQNASVSNHTETQIADLSKMLEMLGEQ
YFKVVHNTLEAALPEHLYMGARMANWGMPDEIITASLKYSDVLSFNIYEEGVQEDYWKFLEDVDLPVVIG
EFHIGSTTDSGMYNPGIVHGANQQDRAQMYKDYMQSVLDKPYMVGAHWFQYIDEPISGRAFDGENANIGF
VTVTDIPYPHMIQAVKDVTSTMYQKRLAD

>AFV00513.2
MNRIPGLTLSAIALSAFLAACGGGGGSAPTPTPTPTPTPTPTPTPTPTPTPTPTPTPAPTPTPTPTPT
PTPTASVKVDVNLDLRHTVGGVDSFDRRKFITLHASNLENDWTLGTNEDPDLMESFLVGLDVYFGRDTGN
LTYSATQLPQDPNRAGFIDEAAAENNGGSARWNYTMGTEAKWTLGRAYADRATDMVVGAQQHPFWPDGTE
VGQQGWSFSQTDTADEPFGTAVGHYMANFLYWYFNRGENDVESPGNPWGSAKPLYLEVMNEPLFELVDYP
SGTPTTPEKVFEFHNNVANEVRAFRDQWGLASHDNVKIGGYTVAFPDFERNDFNHWETRDKLFIDIAGTN
MDFISMHLYDFPVFNGKYQLRRGSNVEATFDMIEQYMHMTLGEVKPFVISEFGASTHALFNDPWTPYRDG
LKLKALNGLMMSLIERPDQIAKAIPFIPVKAEWGRTSVPYNDRLMRQKNEAVGGAGEEWVYTDLVKFYEL
WSDVKGTRVDTWADDPDFQVDAYVDGETLHLIINSLEMADTTIDLALLGANGNTVTQVNTKHLYYDADGN
PVLDTNTTAQLPTALEVKDQATIIVSVRFEQALSQTRASEEAKYYASSYKQAISSNSTISQSIDGVTLGT
AGEAVLRLGIGRAHGKSLLPIVTMNGETLTVPTDFRGYDQYHNGQGRDSFYGVIEIPVPYSALRENNAVE
VSFPDDGGWLASTALQVFSMPSSPNRGQR

>AFV00535.1
MEKFTRGLLCAALATTLVACSNSTTAPEAHASKTIMLADFNSPTDGLQISTEGGAEFTRLESGELTVTFS
PVEKFSKLILQPSTPWDLSGRPELNLAMDVQNMSEESIQLYIGFKNAQGRATNHSVNVAAGSTKTVYVLL
AGHAAKVDLGFKHSRMPAWESDDALAFFRYGDDLLDLSALSEISLSVRGNLTPKHIRVDNIRARTNPDYP
SDFHKGYVDAWGQNAKVDFPGKIQSDEQLKAAADAELAALSKSGLMPDRSRFGGWKDGPRLEATGYFRRE
KVDGKWWLVDPDGYLFFSHGLANVRLANLSTTTGIDFKDDSVRYVDPDAVTPEDSMGMVKVSDAVRATRY
VASELRHNAFTWLPGYDDPLADHYSYRRSVFLGPVKSGETYSFYRANLERRYGETEPESYMRTWEQVTLD
RFNDWGFTSMGNWVDPAFYTNEQVPYFANGWIIGNFKTLTSEVSYWADMPDAFDPEFVRRAQVTIDVIAE
EIQSSPWCVGIFVDNEKSWGLREGTISQRYGLILDALSKTTADSPAKAAFVTALKQKYPTIEALNEAWGT
ELSGWAALSESQTFDAFPEAFVADLSMMLEMLSDQYFRVVHDALEKTLPNHLYMGVRMASWGMPDETIKA
SVKYSDVLSFNVYEEGLQPKLWSFLDEIDLPAVIGEFHIGATSDTGLYHPGLVQAANQQDRAQMYLAYME
SILANKNLVGAHWFQYVDSPISGRAFDGENYNVGFVSATDIPYPDMVKATKAFNASVYPKRYKLGRK

>KHT62341.1
MPSLKEHRLLNFSRESLPNELMFLHTDAFITPVGENQYGVRITSRAKDNFYTATNLVPEMPWDWSQLPNF
SFAFDASNLANHSTQVFINIFNTQGEMHSRSITIPACRNPRTYICELKGDYLQGKTNHYSGLRSNPAPFD
TPYEYATWMWGAINIDLTSICKIELSIHGSLLDHELVLSNFRLIQSPPTNPDYLTGIIDKFGQNANVDYV
QKVHSESQLLALKDAELETLKAGKMPQRSTFSGYTGAKQFEATGYFRTLKIGGKWSLIDPEGYPYFATGI
DIIRLANAYTMTGVDYDHSKIEQRQADDLTPEDSIEKVTVSSEAKQTAFIASDMRRNCFNWLPSYDDPLA
EHYGYMRELWEGPTAQGETFSFYAANLQRKYGENYRQIWSDVTIDRMLNWGFTCLGNWAAPEFYRNEKIP
FFANGWIIGDFKTVSSGDDFWTALPDPFDPQFEQRAIATVQQVKAEIQGSPWCVGIFIDNEKSWGRMGTI
QGQYGIPIHTLSRDAVESPTKAVFMTVLQEKYQTIEALNQAWSTQFTSWEIVAKGVCDLAHNEAQCEDYA
LLLETYASEYFRIVSQAVKAELPNHLYLGARFADWGMTPEVVRACAKHADVVSYNYYKEGLHPEPWTFLE
EIDMPSIIGEFHFGARDTGFFHPGLVSAESQDERGEMYERYVQSVVDNPYFIGCHYFQYIDSPITGRSFD
GENYNIGFVSVTDVPYDGMVNAAKTINSSLYQQRFNQLTK

>WP_036188492.1
MPSKASIGTALALGLTLGVGTGCAPTQDTADDTQRPFTPLQSIADFNATPPSALYEDSEGTRSELHSTDS
GEQQLHIRFSPKVYKAAVTLRPENLWDWSDFNDFHLAMDAHNPGNESVQLWLGITDASGAVRRQSVNLAA
GESGTYYVVLNGPFNQLDSGLRENPPPWDADEMFYWRHGTKDLDLSQIQKITLFVEGNLTEKVITVDDIR
LRQNPAYSSSHIQQFVDRFGQNATVDYPIKIHSESELKRAAKRELEALESNGPMADRSRFGGWKDGPRYE
ATGYFRTKKVDGKWWLVDPEGYLFFSNGLANVRMANLTTLTGVDFRDPSVREVDPDEVTPEDSIGIVELS
DEVRQTRYVASSLRHDMFNWLPEYDHPLSDHYSYRRSVHKGPMPSGETFSFYRANLERRYGEQASESYVR
QWEDVTLDRMQSWGFTSMGNWVDPAFYPNEQVPYFANGWIIGDFATLSSKHDVWAPMPDPFDPEFVRRAK
VTIDVIAEETQGSPWCIGVFIDNEKSWGRPEGSVSERYGIILDALSQPAAQSPAKQAFTRHLKKTYKSIA
QLNERWGTSLASWDALAEKSYAPSTHTDAAVEDYSTLLALLSEEYFRVVHNTLEAALPNHLYMGVRMASW
GMPDETVEASIKYSDVLSFNIYDEGVQPHAWDFLNEIDLPSIIGEFHIGATRGSGLLHPGLVMADDLADR
AEMYKRYMESVAAHDTMVGAHWFQYVDSPITGRAFDGENYNVGFVSVTDIPYPNMVEAARSFNKTLYPKR
FNDQ

>WP_035480747.1
MNAKFSPEQVVAVSDQEQSILIYDFSGDALPSAFSFNNIDAELVSQGAGITCGEQALKIHCNSAENMYTS
VYIEPETPFDWSKLADFSFAFDTTNTGSRCTQIFINIFDHKGQMHSRSVNVAAGVTQTNLVELKGEFLRN
TTSCESGLRSDPASFDTPFEYATWMWGLMNIDLTAIAKIELSIHGTLIDHDMVLDNFRLIMSPEHNRDFL
KASIDRFGQDANTEFAEKVQSEEDLLKRMDDELSVLEEGAMADRSRFGGYTAGERYEATGFFRTQKLGDT
WALIDPDGYPYFATGIDILRLANSYTITGVDYDHDLVTQRTSEDLTPEDSMEKLVVSKAAMASGKVINQW
RRDFFQWLPDIDDPLAEHYSYAREVWEGPVEQGQIFSFYGANLQRKYGKDYMETWRKVTIDRMVNWGFTS
LGNWTAPEFYENETVPFFANGWIIGDFKTVSSGDDFWAALPDPFDPLFRTRAEATVAQVREEIKDTPWCV
GIFIDNEKSWGRMGTIEGQYGISIHTLGRDAAECPTKAVFVELMKNKYSSIDALNASWGTAIASWDAFAK
GVKDLEHNDAQLVDYAICLEAYASEYFRVVNESLKAVLPNHLYLGSRFADWGMTPEVVRACAKHVDVVSY
NYYKEGLHPQPWKFLAEIDMPSIIGEYHFGVKGEGLHHAGLVTASCQEERGKMYEAYMHSVIDNPYFIGA
HWFQYIDSPVTGRSYDGENYNVGFVTNADIPYQPMVDAAKRIHSSMYQRRFGGKK

>WP_035016017.1
MKIRSKIAAIPLLGIGLAACQSSDGNTQGQVETKPDKVLFDFENSAVPVEVKVANATASIVKQGATSGDQ
ALNVKFNSKAHEWTSIEFTPAQPWDWSEYESFSIAFDISNVGQYSTQLFLNVFDKNGQVYTRSVNVPVGE
DAKTYYSKMAGHDLVSPKNKHGENVELNLSSGLRSNPPTWNEGDDQVQFIWMWGNKNLDTSGITKISLSV
QYNLHNKEVTIDNVRVMPNPKMDPDYLVDIVDKFGQPSRMEYEEKIHSEAELLAVKEKELAELQGGKKIS
DRTKFSGWKDGPRLEATGYFRTEKVGGKWSLVDPEGYLYFTTGIDNVRMSNATTLTGYDFDQTAINQRTA
DEVTPEDSQGLNPPPPSAIPTRHLVSETRKNMFEWLPDYDDPLASSYGYRRGAHSGPLERGEVFSFYMAN
LERKYGETTPYSYLKDWSKVTVDRMLSWGFTSFGNWIDPMYYQNNRIPYFANGWIIGDFKQVSSGNDFWG
GLPDPFDPLFAERADITVKTIANEVKGSPWCVGVFIDNEKSWGRPESKVSELGIVIHTLTRDGNDSPTKN
MFTDTMKAKYGSISKLNKAWGTNVASWDAFQQGKINSKLDVNNATQIADYEQLLYNYAAEYFRIVDEALA
KYMPNHMYMGVRFADWGMPKPVVKAAAKYVDVVSFNLYKEGLTQNKWKFLEEMDMPTVIGEFHMGTTASG
FFHPGLIHAANQEDRARMYKDYMRSIIDNDYFIGAHWFQYLDSPITGRAYDGENYNVGFVSVTDIPYNAM
VKAARELHTELYERRYGHLKQNK

>WP_035014943.1
MYTTRTQHTKLASLIILALSLSACGGSSESDDGNDSPTTPVEQTDTTPNTFNFSAQNNVALSTQITSASI
TITGINTKTPISISGGEYAIDNGAFTSAQGEIENNQSIKVRLTSAEHYNKTTSATVTIGGVTSKFDVTTE
QFGGVQVDINLDTKHSLNGFDSFDRQKYITIHATQTEPDWGENDGHSANAANKDPDLMLNFVNNYDVYYG
RETGGNKWHLRNVQEDTSKPGFVDEASMAKRGSDTKWGYSNFSKKINQDARAAEDRGLDLIIGAQQHPYW
PEGTLVNAIGSNDWAFSTTDTSAEPLGTATGHYLAHYLAKFFKQENDTQGQPKPKYFEIMNEPLYDLTTV
RSGSDRVDPATIFEFHNTVAAEIKKLPENSDILVGGYTVAFPDFDKDNFQRWFDRDKQFIDIAGANMDFY
SIHLYDFPCFRNSERYRKGANAEATMDMMEHYSRIATGTMKPYVISEYGAAIHCLNKAGWSPERNTYQLR
AVNSLLMQFLERPDVIAKTIPFIVVKAEWGRTDYPYGPRLMIQEFEKTGDPTQTDWVYSDLVKFYQLWSN
VNGTRVDSKSSDLDIQVDAYVEGKKAYVILNNLEFDDANIKLNTLGLNGNQVASVKIKHLETLAGAEMQS
NLTTLDSQTLPESVTLGKEATMIIEIAYTQDVQISEEVEEKKYYATEYLKTIEADTQIEYTINDVAIDTN
GEAVLRIAVGRDHGLSLTPSVKVNGTQVEIPNDFRGYNQQQGSTMTGRDNFFGVLEIPVDYALLQASNKI
SLTFADDGGRVASASLQVFNSSKQLTRSF

>WP_035013115.1
MNFRRKTMLHLVWPIALASLAACQAKQEHKTKDTLIFGFESSQVPAEIKLSNASAELVSKTEGTTEGAQA

LRVKFSSKHNEHAAIHIEPAKPFDWSELSDFNLAFDMANQGKHSTYLWLKVTDVDGKGFSRAINVPVGES
RTYYAKLDGHDLGSPNNDKGEQVELNLSSGLRSNPPTWQSDDEQFTWMWGAKHLNLSGIKRISLSVQYNM
HDKEVTIDNIRLRANPAMDKNFLVNIVDKYGQNAKAEFAEKIHSDKELQAKTQQELAELKANQLMDDRSD
YSGWKKGPKLRATGYFRTEKVDGKWWLVDPQGYLYLATGIDIIRLANSTTMTGYDFDQALMKKRDTENVT
PEDSQGLNTVDKNIAKTRQLVSSTRANMFEWLPESYDDPLADHFGYRKSAHSGPLKHGEVFSFYSANLER
KYGESSNNSFLTDWKNVTVDRMRNWGFTSLGNWTDPMFYDNAKIPFFANGWVIGDYKTVSSGNDFWSPMP
DVFDPEFARRAEVTVKQIAAEVNNSPWCVGVFIDNEKSWGRSNSRNSELGIVIHTLTRDGKDSPTKAKFT
QVMRDKYADISKLNQAWGTDIQSWLAFDQGQFDSSLTNEVQDADYGHLLYVYAQQYFKVVSGKLKQYMPN
HLYFGVRFASWGMPKEVIKASIPYTDVVSYNHYKQGVTDKKWQFLQEIDKPSMIGEFHFGAKTSGFFHPG
LIQAADQQDRAAMYKDYMRSVFNNDYFIGAHWFQYMDSPITGRAYDGENYNVGFVTVADIPYEEMVKAAK
ELHSEMYQKRFERKANN

>WP_033186821.1
MLLADFEEAALPSWIQRDHIQAKITKAAGITSGNQAIELTFDSSSEYSTLSLQPAKPWDLSVLGDMNLAF
DATNKSDVSVHLYLRVNDTHQMQTRSISIPAHSSATYYADLSGPQISLDSGLRADPPAWSSDEFRMPYMT
GNKLLDISEIKEVSFYISSNIEDKQVILDNVRARSNPAIDPEYLVGLVDEFGQPAKSNFKDKVKSDSHLK
QLAEEELADLAANPVLADRSKYGGWKKGPKLEATGFFRTEKVNGVWSLVDPEGYLFFSSGIANIRIANTT
TLTGVDFKDDQVRYIDPEDVTPEDSLGIRPVSAKAQKTRYISSELRHNMFNWLPDYDDELANHYSYRRST
HKGPLAHGETFSFYQANLERRYGERYPESFIDDWEDVTIKRMRSWGMTSFGNWVDPEFYHKNQFPYFANG
WIIGDFKTVTADGHGWSPMPDPYDPEFARRAKVTTQVIADEVKNNPWCIGVFIDNEKSWGNPASIESHYR
IPIQNFKMDASQSPAKAEFTKLLQDKYGDIATLNRAWETNIPSWDSFAKKFDVEKINDAMIEDLSVILET
YTSQYFDVVNSALREVMPNHLYMGVRMAAWSINPEGVRAAKKYVDVMSYNYYREGMHDSTWDILPKIDMP
SIIGEYHFGAMDTGLYHPGLIHSSDQKDRARGYQAYMRKVIDNPYMVGAHWFQYTDSPVTGRAYDGENYN
VGFVTNADVPYKEMVEAAREINQELYPRKFKDAVK

>WP_033186681.1
MKNPQLTKKVSFALSAIAFAIALNGCSHDQQGLAKTSSTTKNTPTKNSLTDAFAKEQRIASLIDFDDQQQ
LSWLHESDISLTQITSSDSLVNKQVAVEFAKTGNISTLRVEPPKPWDLSQYENYNIAFDVENTSAESIHL
YLSLENPNGEVQSHSISLAKGYKGTVYFPLDGIEAETDSGMWGDVPPWTSKDDLMVWRSWRNAEQNYDVV
SALNFFTIGILENKSVILDDVRLRANPAHDPNVMVGLIDKYGQNAKQSTPLDVHSDAQLKQQAEEELAEL
AKSSGMPDRSRFGGYTKAPKREATGFFRTEKVDGKWWMVDPEGYLFFSHGPANVRMANMSTLTGIDYDQP
SIRERTSDEITPEDSMGIVSVPQSAKEKRYVISKARNDMFEWLPSYDDELSEHYSYRRSTHKGPIPYGET
YSFYRANLERRYGDDGVSAGSDPSYVKRWHDVTAQRMHDWGFTSFGNWVDPAFYQSEQVPYFANGWIIGD
YQTLSGHTNHWGLMPDPFDPVFAQRAKITIDAIAENIQGSPWCAGIFIDNEKSWGEREGTVSQRYGVILD
ALSKNSQQSPAKKAFTAHLQQKYQTIEGLNSAWSSDFNNWKTFEQNASVSNHTETQIADLSKMLEMLGEQ
YFKVVHNTLEAALPEHLYMGARMANWGMPDEIITASLKYSDVLSFNIYEEGVQEDYWKFLEDVDLPVVIG
EFHIGSTTDSGMYNPGIVHGANQQDRAQMYKDYMQSVLDKPYMVGAHWFQYIDEPISGRAFDGENANIGF
VTVTDIPYPHMIQAVKDVTSTMYQKRLAD

>WP_033186679.1
MKPSPKSPGFILSAIALAVTALAGCNNNQASNNSVATSSDSGAKHAQHKPLPWDFAGMTDLQNVTLTAAG
ARVINTNIDGDGEQKLAIDLHSKEHKSAGFSFIPDTPWDWSQEGQFAFAIDIENPSSASVHLYVSAKDAE
GQSHNRSFAVPGNSSDTYFMALNDPDLSIETGIRSNPNNWETEFTPMIWRYGTKQIDLSQVKSIEFDVRG
VPEDKHLIVDNLRLIKPQKLDPNYLVGLVDEFGQNDKLEFTNKIDTVEQLQALNAKEQSAFTHEVPQGRS
KFNGWANGPKLAATGYFRTEKYQGKWTLVDPEGYLFFSNGIANVRMSNTSTITGYDFDSQFIKQRAPGDF
TPEDSIGLNRAPKAAWPSRHVSSELRANMFTWLPSVDEPLAEHFGYRREVHTGVVDKGETYSFYSANLAR
KYASNDPKVFMTKWRDTTVDRMLDWGFTSFGNWIDPSFYQLNRIPYFANGWIIGDYKTVSSGNDYWSPLP
DPFDPVFKQRAMVTATKIAQEVQNNPWCVGVFIDNEKSWGQEGSIEGQYGIVIHTLEVDAKDSPTKAQFV
TYLKNKYSDIAELNGKWNTKVRTWDDVATGITLTKFNDPIIEDLSAMLSLYAEKYFAVVHDAVEQTMPNH
MYMGARFADWGMTDEIRNAAAKYADVVSYNYYKEAITEQAWGFLAQIDKPSIIGEFHNGALDSGLLNPGL
IHGASQADRGKKYQEYINSVIDNPYFVGAHWFQYIDSPLTGRAYDGENYNVGFVSVTDTPYQPLVDAAKA
VNAHIYTRRFGDAQPK

>WP_033185649.1
MNASKKLLCVAIVTALAGCSASETKDNVSHSSAAKAQAVGHVEQQEKTLQTLFIATNSNSLKQVKYTNAS
GTVVDSAGNGLKVLFYGKENINSAVEFIPDVAWDWSDLDDFNIAFDIGNEGDHSVQLFLNISDTNGDTYT
RSVSVPVGPQSTYYAKMAGHDLAKSISDDKNEFNFTSGLRSNPDTWHSDDKQFISLWGKKNLKLSGISKI

SLSVQNNLFDKQITINDIRLRQNPPIDKLYLTGVVDQFGQNAKREFGGKVHSLAELKDARDRELQTLDGK
WNAPRSKWGGWLNGPKLEGTGNFRTAKYNGKWSLVDPDGYLYFATGIDIIRLANSSTMTGYDFPPEVLVK
ADNADVTPSDSQGLNRVADSAVPARFVASELRKDLFTWLPSYDEPMGQHYGYRTGVHSGPLKQGETYSFY
SANLDRKYSEMTSDYMQKWHDVTLDRMRNWGFTSLGNWTDPAFYDNQQVPFFANGWIIGDYKTVSSGDDF
WGNMPDVFDPKFKERALHTVSVIAEEVKNTPWCVGVFIDNEKSFGRSETPQSRYGIVFNTLKRDGSEVPT
KAAFTKMAKNKYTSIEALNAAWGKDISSWSAFDKGIDSVLATDETQAKQQLADYSDMLYAYADKYFSTVD
AAMQTYLPNHLYLGSRFADWGMPMEVAKAATQYVDVMSYNIYKEGLHPKGWGFLKDFDMPSIVGEFHIGA
TDSGLFHPGLVHAANQQDRADMYQDYMGTIIDNPYFIGAHWFQYMDSPITGRAFDGENYNVGFVNVTDTP
YTPMVNAAKALHKGMYERRFGSK

>GAL10871.1
MAEDWREIPIPAPLEDGQSWELQEAYSDSFNYTGKGDSFRSKWNDTYFHGWTGPGLTYWQSDESWVDDGN
LIISASRRHGTEMVNAGVVTSKTKVKYPIFLEANIKVSGLELSSNFWLLSENDQREIDVLEVYGGAEDEW
FAKNMSTNFHVFFRNEDNSIRSDFNDQTHNEPQWGTYWRDGFHRFAVYWKSPTEVTFYINGIETPKGSWE
DVVMKDKDYTGAILDKSTYNMDQEMFIILDTEDHSWRSELE

>GAL11211.1
MDRMLNWGFTSLGNWTAPEFYANEKVPFFANGWIIGEFKTVSSGDDFWSPLPDPFDPLFKERAEATVKQV
REEIKDTPWCVGIFIDNEKSWGRMGTIEGQHGIAIHTLSRDANECPTKAEFINVLRDKYGDIESLNARWG
TEIASWEALSQGVKGLANNEAQLEDYGILLEAYASQYFKIVREALKAELPNHLYLGCRFADWGMTPDVVR
AAAKYCDVISYNYYKEGLHPQPWSFLSEMDMPSIIGEFHIGSKDTGLYHPGLVTAGNQQERGEMYEAYMH
SVIDNPYFVGVHWFQYIDSPITGRSYDGENYNVGFVSIADTPYEPMVEAAKRLHSSMYKRRYNS

>GAL11210.1
MPEFCFAFDAHNLRSRSTQVFINIFDSKGQMHSRCVNVTGDTDTSYLVELKGEYLKGNTNYYSGFRSNPA
PWDSPFVYATWMWGLMNIDLSDIVQVELSIHGTLIDHELELSNFRLMLSPDINPSYLSNIIDCYGQNAGF
EYPEKVHTDQELTEFTERELQMLKEGAMPDRSRFGGYKEGKRYQATGFYRTEKIDGKWSLVDPEGYPYFA
TGIDIIRLANSYTQTGVDYDHSKVEQRSPDDLTPEDSIEKFEVSMEAKQTAFVGSDVRRNCFQWLPDYEE
ELGEHYAICVRILKVPSIKAKLSVSTPPTYNENMASNTWISGEKSPWIACLTGASPL

>GAL11999.1
MKKTKIALILSSLLIGAYGCQSTTGSQPEAATESEEQTSVAIPDFESDSFFNNAQASHAKAEKVSGVGVT
SGDSALKVTFDSVSEANKFKYWPNVKFFPEGGMWNWNNKGSLKVDVTNPTDKPATFIFKIVDNVGQMGAA
TNQLNYAVTIPANSTEKVEMLFNGGKRGLDGYWDGQNINLRKLAELQVFVQGPMDKQTIILDNLELVDAT
GDFISAEAQVVEAGPIPTLKSITSFESGEKHYVSDRSVSTSIEKVKSDGDLGLQVKFKADNAYPNVTFMP
NRPWDWSNEGNFNLAFDIENPTNDPIQMFVRVDQAENKNWGGTADGVQDSMSNYVTLLPNEKSTYYMTLS
QLEGHIVSGMRSEPPKKAYSAQAISYGWGETSLDLSNIVSIQLYLQNPTKDATIVVDSIRLVPNLDADAT
RYEGLVDQYGQFTGSDWTEKVHNDEELRESGKAELAELGNVKPMSDRSKFGGWNAGPRLEATGFFRAEKL
EGKWTLVDPEGYPFFMTGLANIRMDDTVTITGADYANPKTKEGRLIASPLRESMFTWLPDYDDELAINYD
YAGYVHSGALKKGEVFSFYRANLMRKYDTNQQEALEIWRDVTLDRMQDWGFTTLGNWIDPMFFGTERVAY
EAHGWIAGDHKRISTGNDYWGPIHDPFDPEFQQSTRAMAEGLAKQVDKNDPWLLGIFVDNEISWGNVMNE
ANHFGLIVNALSYDAKESPAKAAFSQHLKEKYTTINALNKAWGTTQVSSWEEFDQSFDYRSSLNPGMKRD
YSDMLTLLADKYFATVDAELERVLPNHMYLGARFADWGVTPEIAKGAAKYVDVMSYNLYANDLNDSKKAH
FRKWLAELDKPSIIGEFHFGSTDTGLFHGGIVNVANQSERAKMYTHYMQSVVDNPYFVGAHWFQYLDSPA
TGRAWDGENYNIGFVTIADEPYVELIEAAKQFNANLYNNRFK

>GAL13779.1
MVVHGIDFIERTGDFVVSEARQGQVIEAQIPTLLTVTDFEQGIDQIVERHTGSNVDIVETSTGSGIKVHY
TTDDDYPTIKFSAGTNGEAWDWSEFGDVALAFDAKNLGDSGMQLFVRVDDALDEKLGGTATGAVNSRTGY
VQVPANSEDKYYFTFKDLAEGLDSGMRGEHLRNLSLQAKLFLAGVKPSLTYLISSVFSFT

>GAL13778.1
MMNPQEEATLVIDNLSLIPNLSTDTTRYANLLDEFGQYQEESWPEKVTDVAQLKEQGKTDKKLLKKAALM
DDRSKFGGWAKGPKLEGTGYFRTEKVDGTWALVDPEGYLYFATGVDNIRMDDTYTVTGMDFADAVEADTK
GMRPSQVATARYVDDKRERVEASTLRRGMFDWLPDFNDPLADNYSYTQMVHTGPLKHGELFSFYSANLQR
KYDTDTAQEAIDIWKDVTLARMQDWGFTSLGNWTDPMYRKNGKVPYTAHGWITGNHQRVSTGNDYWWAMH
DPFDPQFRVSVATMAKALGEEVDNDPWCIGYFVDNELSWGNTVNDTNHYALAVSGLRESAESSSTKAAFD

ALLKEKYGSVEKFNQAWGTDVASWNEFAKGFNYQGEYTDAVKADLSILLDAFADEFFAVVSEEMEKVLPN
HLYMGVRFSDWGITPEAATAAARYVDVMSYNLYATDLNAKGDWSRLPELDKPSIIGEFHFGSTDSGLFHP
GIISSDDQKGRAESYAKYMESVIDNPYFVGAHWFQYMDSPVTGRAWDGENYNVGFVTVTDTPYELLVESA
KEINRNLYNRRFGSLN

>WP_033039584.1
MQHDNKTILIDDFEHASNSYKLTNKNIKTTQVIKNKNKALELSFSTKHKFSGITLKPNKLWDLSVLGNSA
LFFDVSNVSDFPVMLSVNITGKDKQVQRRTIGLTTNEHATLYFELNSQTLNVDTGLRDTPGSFKTAARKM
ILRGAKLNVDFSQVESIAIYTETQINPTAVTVDNLRFETIPDAKPDFLTNIVDKFGQSTQVNYPLKVSSE
QQLRAIANKELNDLSKVTPRADRSKFGGWKQGPKLKATGFFRTEKVNGKWALVDPEGYLFFSSGIANARM
ANTTTFTGVDYRDDAVRARDPDDVTPEDSKGLNSNLAKYQKSAYIAYPDRRAMFNWLPQYNDKLANHYSY
KRSSHLGPIQHGEVFSFYQANLERRYAQQYPDSYIDKWREVTLKRMQDWGFTSFGNWTDASFYNNQQVPY
FANGWIIGDFKRLSSGFDYWGAMPDPFDPEFVKRANITTQVIAQEVQNNPWCIGVFIDNEMSWGGEGSTT
LRYGIVLDALSKTTGNSPTKSVFGDMLKQKYKTISQLNKAWNRNIKSWEVFNNTGVNYKKDSNFNNAMIA
DLSWLLTRFSDEYFKVVNHSLKSVLPDHLYMGARFTSWGTSPEARWSAKKYADVISYNYYREGLDPMTWD
MLKELDMPTIIGEFHIGSGDTGQPNPGIIHAANQRDRADMYKTYMKTVIDNPYLIGAHWFQYIDSPITGR
AYDGENYNVGFVTTTDIPYPELVEAAKQVHKSLYQQRYGDVEIK

>WP_033039368.1
MLCCLIGLAGCNNGKISEKKPNNTVVDFKKQLVIFDFEKNNYSDNINTINASTKLVEQSGNHKLQVNLYS
KSHTESDFEFVNPQGWDWQAIGNFALAIDIQNPNSASTHIYVKTTDKSGKSQTRSVVVPGHSENTYYIEL
KGANLNINSGIRSNPPSWHSGYTPIIYRGGQKNIDVSSIVKVSLGVKGLLEDKRFLIDNLRLIKPTNFDV
NYLKGLVDEFGQNAKLNFINKVTSTEQLLVISQKEQAQLQAQPLQGRSKFSGWKNGPQLKATGYFRTEKY
KGKWSLVDPEGYLYFSTGIDNVRMANTSTITGYDFDQSYIKQREAGDLTPEDSLGLNPAPVAAWPTRYQS
SKLRAEMFNWLPKQDDPLADNYGYRREVHSGAVKKGETFSFYRANLERKYQTHNNNELMKQWQKTTVNRM
LSWGFTSFGNWIEEQYYHTKKLPYFANAWIIGNFKTVSSGNDYWSPLPDPFDPLFVERADVTLAKVAQQV
KNSPWCVGVFIDNEKSWGMMNSDTTRYGIAINTLKNNAKNSPTKAEFVLLMKNKYSKISKLNKAWNTTLS
SWDEFAEGVTLTQFNDHVNIDLSAMLFHYANQYFAVVDEAITKHLPNQLNMGARFADWGMTPEIRAASAA
HVDVMSYNYYREGLNQEFWEFLSDIDMPSIIGEFHNGALDSGLLNPGLIHTQSQQERGSKYQNYMNSVID
NPYFVGAHWFQYIDSPLTGRAYDGENYNVGFVNVADIPYEPLVNAAKNVNKSLYERRYRNDKN

>WP_029250032.1
MRYRILLPVLTCLAGALIAACSERLDGQQQTSAAQLDKRPIGESLWDFEDTAVPPEIKLHNAVATLVGGT
TDKNGQALNIKLQTENHYTAGFSFSPQSPWDWSELGTFAFALDIANQQPSSVHFYVKAHDQQGKTHNRSF
VVPERSDNTYFFELKGPDLGVETGIRSNPPSWDSKYQDIIFRYGAKDLDVSAIARIEFNVVGVLEDKEIR
IDNVRLIQPQSLDQDYLKGLVDAFGQSAKREFAGKVDSVATLRQQAEEEGSILRKQPLEGRSRFGGWTQG
PKLDATGFFRTQKVDGKWTLVDPEGYPFFSTGIANVRLANTSTITGYDFDRGAVPQRAPGDLTPEDSLGL
NPVPDAALPSRHISSSLRAEMFTWLPDYGDPLGENFGYRREVHTGAIPRGETFSFYRANLQRKYQITDDE
QLMAKWREVTIDRMLSWGFTSFGNWIDPDYYQMDRIPYFANGWIIGNFKTVSSGKDYWSPLPDPFDPLFK
ERAFITAEQVAREVQGSPWCVGVFIDNEKSWGQEGSTETRFGIVINTLSRDAVDSPTKAEFVRLMQEKYA
SIEQLNRAWDTQISSWEQFSGGVALASFNPAMVEDFSALLEHYTGQYFKVVRAAVKHFMPNHLYLGARFA
DWGMTPEVRRAAAKHADVVSYNYYKEGVSDKFWHFLETLDKPSIIGEFHNGALDSGLLNPGLIHASSQVD
RGKKYTEYMHSVIDNPWFVGAHWFQYIDSPLTGRAYDGENYNVGFVSVTDTPYEPLVDAVTRLNKSLYQR
RFTSSEQAAAQGDTP

>WP_027330223.1
MTLKPLVYAVAVTAALTSACSQHTHNPGVHGAGESPAAQLTPRQLPATIVDFAIASERGWLEPEQAHISA
ANNGRTQALNIRFPASVHTPVLSIAPDQPWDFSRLDDYNLAFDVTNLSSVSTHFYVELFDAAGNSQTREL
SVPKGYTGTVFFPLAGEKAATDKGMWANPMPWPTDELKMVWRSWHDELDLGQITKMSFYTIGVLQDRELQ
VGNIQLRPNPDAGKDWTQNLVDRFGQAAKKDTPLKVKSEAQLKALAEQELKTLAQNTGPKDRSRFGGYKD
GPQLEATGYFRTEKVDGKWWLVDPEGYLFFSHGPANVRMSNLTTLTGVDFKDPSVRVVHEDEVTPEDSMG
IVNVSDEVRESRYIINDIRHNMFEWLPGYDHPLADHYSYRRSTHKGPIPHGETYSFYRANLERRYGETAP
ESYVRKWEDVTLQRMNSWGFTSFGNWVDPAFYPNEKVPYFANGWIIGDYQTLSGHTNHWGLMPDFFDPVF
AERARATIEVIAEDVKASPWCVGVFIDNEKSWGEREGTVAERYGVILDALSKDASKSPAKKAFTDRLKEK
YGTVEALNRAWDSQFDNWDVFAADASVSQHTGAQVADLSLLLEALGEQYFKVVHGTLKEYLPHHLYMGAR
MANWGMPDEIIKASVKYSDVLSFNIYEEGMQPHQWDFLEDINLPVVIGEFHIGATTDSDNYHPGIVSAAS
QEDRARMYKAYMESVLEKDYMVGAHWFQYVDEPVTGRAFDGENANIGFVTVTDIPYPEMVEAAKEINFNL

YRERYGK

>WP_027330171.1
MRLLTINKSISILICSLALAACDSDTKERESDPKPQAQLLEVLYDFEEGVKSSVKPSSANLSLQPGPEEG
NVLSVEFKATESSYSGVTFKPEAPWDWSQYESFNLRMDMKSIGEHSTQIYLNVEDADGNVFTRSVNVPVG
DFKTYYAKMSGHDIEGTYDGDDTELNFSSGLRSNPPTWDSEDEMFVWMWGTQQLNTAKITKISLSVQGAL
FDKKVLIDDIRLESNPPMKKDFLVGIVDRFGQNAKVDYPGKVQSEDELIQRRKEETASLKEGMMADRSKF
GGWKNGPQLEATGYFRTEKYNGKWSLVDPEGYLYFATGLDIIRLSNTTTMTGYDYDQDLIKKRDKDELTP
EDSIGMISVSDEAKASRFVASETRANMFKWLPGFDHELANHYSYRRDAHSGPLDHGETFSFYQANLERKY
GEETPSSFLKDWERVTIARMLNWGFTSLGNWTDPQFYDNETIPYFANGWIIGDFKTVSSGNDFWGPLPDV
FDPKFTERANATAAKVAREVQGSPWAVGVFIDNEMSFGRPESDQLRYGIVINTLGRDAKSVPTKREFTRA
MRERYESIEALNAAWDIDLASWEAFAEGFDPKAITPAQREDYSSMLELYASEYYRIVDQALEKHMPNHLY
LGSRLPDWGMPIEVVRSAAKYVDVVSYNAYKEGLTKIKWDFLKDIDMPSIIGEWHIGATDRGLFHPGLIH
ASSQQDRARMYKDYMKSLIDNPYFVGGHWFQYMDSPITGRAYDGENYNVGFVDVTDTPYPEMVEAAKEIG
ESLYQRRFDAK

>WP_026973021.1
MPSLSTKSSLATLVSLALCSGAQAATALQHSSGLCLRTADNSSSPINGTAVVLSGDCSGDAAAFSWNPGN
SIQHQPSGLCIHPGGAAIPDNGQALVLWQGCDFADRVRFDATSANSLQQASSAKCVHPEGGSASPADGTA
LVMWEGCNEQRLAFSVVDQDGGSSASTAVGINLDTKHQVGTIDSFERKKYINVHASHVENDWFGGNAQSL
FAENARPNLMGSFLEDFNVFMGRDTGGMKYELEQLPEDPQKPGYASAAAASTNGGGAKWIYSNTDNAQWQ
AIRRHAGRDDELIVGAQQRPYWPNGTQVGNQGWSFSQTDNAAEPLGTATADYLSQFVEKYFQAGPSDIGQ
PKPKYLEIMNEPLFELVEYPHPGENVPTIQQIFEFHRDAAREIRKNSANDDVLIGGYTVAFPDYEKGDTP
FDQWKERDQLFLDIAGDDMDFVSIHLYDFPHIPYPDGTRRMQNRKGSNMEATLDMLEQYMAYKWGYIKPI
VVSEYGSQLQGAFNEPWSPQRDWLVLNSMNAMTMSLMERPDRIAKAIPFVPIKAEWGRNTDGGRHSYEWR
LMRQKLEEQDHHYDSDDDYFDSIQANAAPSEWVYTELVRFYQLWSEVAGTRVDSYSTNPDVQVDAYVDGN
DVYLILTSLVLGSQEVDLSVFGNNGASVSEVNIRHLYPDGQGRPKYDAYQLSSLPSSVTLGAEASMIVKI
SYDNVISIDHISQETKHYAAALVTNISANVTQSYSINNVDKGSYGEAVLRLGVGRDHYVPNSSPAVRSSL
QPEVLINGVRLNVPDNYRGYDQYHGGLGRDRFFGVLEIPVPYSALNTNNTIQITFPDNGGAVSSVTLQHF
KHSKQLSR

>WP_026959682.1
MNAKFSPEQVVAVSDQEQSILIYDFSGDALPSAFSFNNIDAELVSQGAGITCGEQALKIHCNSAENMYTS
VYIEPETPFDWSKLADFSFAFDTTNTGSRCTQIFINIFDHKGQMHSRSVNVAAGVTQTNLVELKGEFLRN
TTSCESGLRSDPASFETPFEYATWMWGLMNIDLTAIAKIELSIHGSLIDHDMVLDNFRLIMSPEHNRDFL
KASIDRFGQDANTEFAEKVQSEEDLLKRMDDELSVLEEGAMADRSRFGGYTAGERYEATGFFRTQKLGDT
WALIDPEGYPYFATGIDILRLANSYTITGVDYDHSLVKQRTSDDLTPEDSMEKLVVSKAAMASGKVINQW
RRDFFQWLPDIDDPLAEHYSYAREVWEGPVEQGQIFSFYGANLQRKYGKDYMETWRKVTIDRMVNWGFTS
LGNWTAPEFYENETVPFFANGWIIGDFKTVSSGDDFWAALPDPFDPLFRTRAEATVAQVREEIKDTPWCV
GIFIDNEKSWGRMGTIEGQYGISIHTLGRDAAECPTKAVFVELMKDKYSSIDALNASWGTTIASWDAFAK
GVKDLEHNDAQLEDYAICLEAYASEYFRVVNESLKAVLPNHLYLGSRFADWGMTPEVVRACAKHVDVVSY
NYYKEGLHPQPWKFLAEIDMPSIIGEYHFGVKGEGLHHAGLVTASCQEERGKMYEAYMHSVIDNPYFIGA
HWFQYIDSPVTGRSYDGENYNVGFVTNADIPYQPMVDAAKRIHSSMYQRRFGGKK

>WP_025254065.1
MKKTAIALALSVILMGCQKSEENHTNNVNTSGSIEAKSERNSEHPLIQLSKKDAASQIDLVNANVDFVGN
SLNVTFLAKENPHSGINLRPSTPWDLSEFDDFNLAMDIKNPGPHSVQLFLNITDIDGATYTRSVAVPVGE
KTTYYAKMRGHDLATPDGNVNQELNFLSGLRSNPETWESNEVQFISMWGKKNLNLEGITQISLSIDSALF
DKTIELSNIRLRPNPEMDTEFLTNIVDKYGQNATVEFPGKIHSQEELIAAKDTEAEKLDNKLMPDRSRFG
GYKNGPKLEATGYFRTQKVDGKWALVDPEGYLYFATGLDIIRLSNTSTMTGYGFDDALVSLGGDGVTPED
SKGLNRVNDAAVPSRHVVSDVRVNMFNWLPSYDESLGKWFGYRGSAHSGPVKKGETFSFYAANLERKYGA
EDPLEAWEQVTLKRMKHWGFSSLGNWTDPRFYQNNQVPYFANGWIIGDFKTVSSGNDFWSPLPDVFDPEF
ARRADITASNVAEQVKGNPWCVGVFIDNEKSFGRSETNEARYGIVINTLTRDGSEVPTKAAFTNLMKKKY
ESITALNKAWDTDIASWDAFDEGIDSSIRNDVQLADYSDMLYLYGEKYFSIVNAALDKHMPNHMYLGARF
PSWGKPKEIVKAAAEHVDVMSYNVYKEGIHPKSWAFLEDIDMPSIIGEFHMGARDNGLFHPGLIQAATQK
DRAKMYKDYMYSVIDNPYFVGAHWFQYMDSPITGRAYDGENYNVGFVNVTDTPYAPMVKAAQEINSEMYP
RRFK

>KDC54398.1
MNHIPKDVRLTLMLCCLIGLAGCNNGKISEKKPNNTVVDFKKQLVIFDFEKNNYSDNINTINASTKLVEQ
SGNHKLQVNLYSKSHTESDFEFVNPQGWDWQAIGNFALAIDIQNPNSASTHIYVKTTDKSGKSQTRSVVV
PGHSENTYYIELKGANLNINSGIRSNPPSWHSGYTPIIYRGGQKNIDVSSIVKVSLGVKGLLEDKRFLID
NLRLIKPTNFDVNYLKGLVDEFGQNAKLNFINKVTSTEQLLVISQKEQAQLQAQPLQGRSKFSGWKNGPQ
LKATGYFRTEKYKGKWSLVDPEGYLYFSTGIDNVRMANTSTITGYDFDQSYIKQREAGDLTPEDSLGLNP
APVAAWPTRYQSSKLRAEMFNWLPKQDDPLADNYGYRREVHSGAVKKGETFSFYRANLERKYQTHNNNEL
MKQWQKTTVNRMLSWGFTSFGNWIEEQYYHTKKLPYFANAWIIGNFKTVSSGNDYWSPLPDPFDPLFVER
ADVTLAKVAQQVKNSPWCVGVFIDNEKSWGMMNSDTTRYGIAINTLKNNAKNSPTKAEFVLLMKNKYSKI
SKLNKAWNTTLSSWDEFAEGVTLTQFNDHVNIDLSAMLFHYANQYFAVVDEAITKHLPNQLNMGARFADW
GMTPEIRAASAAHVDVMSYNYYREGLNQEFWEFLSDIDMPSIIGEFHNGALDSGLLNPGLIHTQSQQERG
SKYQNYMNSVIDNPYFVGAHWFQYIDSPLTGRAYDGENYNVGFVNVADIPYEPLVNAAKNVNKSLYERRY
RNDKN

>KDC54386.1
MKKRSLYCAVSSALLLSSTFNVHAVKVQFNIDTKHAVGQFDSFDRRKFITLHSSNTESDWFGNNAQSLNA
PNADPDLMTHFLEDYDVYFGRDTGGMKYQLTQLPEDSAKPGYASAATATTNGGGVKWNYTNITTAQAKTM
RKHEGRNSDLIVAAQQHPYYPDGTKIGNQNWSFSQRDTAAEPLGTAMGDYMAQFLQKYFKSGPSDSLGQK
RPTYVEVMNEPLFELHDFPHTGYDKESLYDIFRLHNSVADVINANPALSDVKVGGFTVAFPDYEKGSQFF
DNWKQRDKAFLDIAGNKMDFISMHLYDFPNFPGGPGGQHQIQYRKGSNMEATLDMVEQYMAYKWGSIKPL
VISEYGSQLQGSFGTKWTPQRDWLCLKAMSSMLMSFMERPNRIDKAIPFIPLKAEWGRISDTIPYYWRLL
RQAKEGQGETGEQWVYTEMVKFYQLWSDIKGTRIDSWASDMDIQADAYVDGKDVYLVLNSLEFSPTNIDL
SVLGKGSNNVTGVNIKHLYPNAQGKPVLDNSNRTTLPSSVVLGSESTMIIKVSHQKNVVINNINQETKHY
ASAVVKNIAANATQFFTINNVDKGANGEATLRLGVGRPHGKSLTPVVTVNGNRVAIPTDFRGYDQKNGGL
GRERFFGVIEIPVPYNNIKKTNNIEVTFPDSGGAVSSLTLQNFKMSKRITR

>KDC54219.1
MNKIIRCLSIFVYSTLVLSGEGVAREFDLQHDNKTILIDDFEHASNSYKLTNKNIKTTQVIKNKNKALEL
SFSTKHKFSGITLKPNKLWDLSVLGNSALFFDVSNVSDFPVMLSVNITGKDKQVQRRTIGLTTNEHATLY
FELNSQTLNVDTGLRDTPGSFKTAARKMILRGAKLNVDFSQVESIAIYTETQINPTAVTVDNLRFETIPD
AKPDFLTNIVDKFGQSTQVNYPLKVSSEQQLRAIANKELNDLSKVTPRADRSKFGGWKQGPKLKATGFFR
TEKVNGKWALVDPEGYLFFSSGIANARMANTTTFTGVDYRDDAVRARDPDDVTPEDSKGLNSNLAKYQKS
AYIAYPDRRAMFNWLPQYNDKLANHYSYKRSSHLGPIQHGEVFSFYQANLERRYAQQYPDSYIDKWREVT
LKRMQDWGFTSFGNWTDASFYNNQQVPYFANGWIIGDFKRLSSGFDYWGAMPDPFDPEFVKRANITTQVI
AQEVQNNPWCIGVFIDNEMSWGGEGSTTLRYGIVLDALSKTTGNSPTKSVFGDMLKQKYKTISQLNKAWN
RNIKSWEVFNNTGVNYKKDSNFNNAMIADLSWLLTRFSDEYFKVVNHSLKSVLPDHLYMGARFTSWGTSP
EARWSAKKYADVISYNYYREGLDPMTWDMLKELDMPTIIGEFHIGSGDTGQPNPGIIHAANQRDRADMYK
TYMKTVIDNPYLIGAHWFQYIDSPITGRAYDGENYNVGFVTTTDIPYPELVEAAKQVHKSLYQQRYGDVE
IK

>KDC54204.1
MTYKKNILLTSLLLALSACNQEDVKVDRPTTDAVQIKSTNTLMYLFADDTHHTATSVDYQSNSAIVKNEN
SVLNVQFQSKKNSYASIVFSPEKPWDWSEFNDFNLAFELANPGTHSVQIYLDISDIDGANYTRSVNVPVG
GYNTYYAKLDGHDLATPDGKENVELNFTSGLRSNPDTWESDEVQFISMWGKKNLNLKGIAKIAISVQSTL
HDKELAIKSISLRKNPQFNTAFLTKIVDEFGQNAKQEFAGKVHSEAELLSDKKQEATQLLSKRPTNRSRF
GGWAKGPKLEATGYFRTAKYNDKWSLVDPDGYLYLATGIDIIRLANSTTLTGYDFDQALLAKPADAGVTP
EDSKGLNQVNKEALKSRFVASQVRKNLFEWLPDYSDPLGKHFGYRKSAHSGPLEHGETYSFYAANLERKY
GQNNADYMQKWREVTLDRMITWGFSSLGNWTDPSYYDNQKVPYFANGWIIGDFKTVSSGNDFWGAMPDVF
DPEFTVRANETVSVVAKEVKNSPWAVGVFIDNEKSFGRPDSVKSHYGIVINTLGRDAKTVPTKAEFSRLM
KEKYTDVAKLNKVWHLNLASWVEFDKGVTVDIKNEEQLVDFSILLTAYADKYFSVVNAAMDKYLPNHMYL
GARFPDWGMPIEVVKASAKYVDVISFNAYKEGLRDDKWAFLSQFDKPAIIGEFHVGSSDSGLFHPGLIHA
ANQQDRANMYTDYMNSVIDNPYFIGAHWFQYIDSPITGRAYDGENYNVGFIGVTDRPYIEMIEAAKAMNE
SMYERRFKK

>KDC51314.1
MNHIPKDVRLTLMLCCLIGLAGCNNGKISEKKPNNTVVDFKKQLVIFDFEKNNYSDNINTINASTKLVEQ

SGNHKLQVNLYSKSHTESDFEFVNPQGWDWQAIGNFALAIDIQNPNSASTHIYVKTTDKSGKSQTRSVVV
PGHSENTYYIELKGANLNINSGIRSNPPSWHSGYTPIIYRGGQKNIDVSSIVKVSLGVKGLLEDKRFLID
NLRLIKPTNFDVNYLKGLVDEFGQNAKLNFINKVTSTEQLLVISQKEQAQLQAQPLQGRSKFSGWKNGPQ
LKATGYFRTEKYKGKWSLVDPEGYLYFSTGIDNVRMANTSTITGYDFDQSYIKQREAGDLTPEDSLGLNP
APVAAWPTRYQSSKLRAEMFNWLPKQDDPLADNYGYRREVHSGAVKKGETFSFYRANLERKYQTHNNNEL
MKQWQKTTVNRMLSWGFTSFGNWIEEQYYHTKKLPYFANAWIIGNFKTVSSGNDYWSPLPDPFDPLFVER
ADITLAKVAQQVKNSPWCVGVFIDNEKSWGMMNSDTTRYGIAINTLKNNAKNSPTKAEFVLLMKNKYSKI
SKLNKAWNTTLSSWDEFAEGVTLTQFNDHVNTDLSAMLFHYANQYFAVVDEAITKHLPNQLNMGARFADW
GMTPEIRAASAAHVDVMSYNYYREGLNQEFWEFLSDIDMPSIIGEFHNGALDSGLLNPGLIHTQSQQERG
SKYQNYMNSVIDNPYFVGAHWFQYIDSPLTGRAYDGENYNVGFVNVADIPYEPLVNAAKNVNKSLYERRY
RNDKN

>KDC51303.1
MKKRSLYCTVSSALLLSSTFNVHAVKVQFNIDTKHAVGQFDSFDRRKFITLHSSNTESDWFGNNAQSLNA
PNADPDLMTHFLEDYDVYFGRDTGGMKYQLTQLPEDSAKPGYASAATATTNGGGVKWNYTNITTAQAKTM
RKHEGRNSDLIVAAQQHPYYPDGTKIGNQNWSFSQRDTAAEPLGTAMGDYMAQFLQKYFKSGPSDSLGQK
RPTYVEVMNEPLFELHDFPHTGYDKESLYDIFRLHNSVADVINANPALNDVKVGGFTVAFPDYEKGSQFF
DNWKQRDKAFLDIAGNKMDFISMHLYDFPNFPGGPGGQHQIQYRKGSNMEATLDMVEQYMAYKWGSIKPL
VISEYGSQLQGSFGTKWTPQRDWLCLKAMSSMLMSFMERPNRIDKAIPFIPLKAEWGRISDTIPYYWRLL
RQAKEGQGETGEQWVYTEMVKFYQLWSDIKGTRIDSWASDMDIQADAYVDGKDVYLVLNSLEFSPTNIDL
SVLGKGSNNVTGVNIKHLYPNAQGKPVLDNSNRTTLPSSVVLGSESTMIIKVSHQKNVVINNINQETKHY
ASAVVKNIAANATQFFTINNVDKGANGEATLRLGVGRPHGKSLTPVVTVNGNRVAIPTDFRGYDQKNGGL
GRERFFGVIEIPVPYNNIKKTNNIEVTFPDSGGAVSSLTLQNFKMSKRITR

>KDC49080.1
MTYKKNILLTSLLLALSACNQEDVKVDRPTTDAVQIKSTNTLMYLFADDTHHTATSVDYQSNSAIVKNEN
SVLNVQFQSKKNSYASIVFSPEKPWDWSEFNDFNLAFELANPGTHSVQIYLDISDIDGANYTRSVNVPVG
GYNTYYAKLDGHDLATPDGKENVELNFTSGLRSNPDTWESDEVQFISMWGKKNLNLKGIAKIAISVQSTL
HDKELAIKSISLRKNPQFNTAFLTKIVDEFGQNAKQEFAGKVHSEAELLSDKKQEATQLLSKRPTNRSRF
GGWAKGPKLEATGYFRTAKYNDKWSLVDPDGYLYLATGIDIIRLANSTTLTGYDFDQALLAKPADAGVTP
EDSKGLNQVNKEALKSRFVASQVRKNLFEWLPDYSDPLGKHFGYRKSAHSGPLEHGETYSFYAANLERKY
GQNNADYMQKWREVTLDRMITWGFSSLGNWTDPSYYDNQKVPYFANGWIIGDFKTVSSGNDFWGAMPDVF
DPEFTVRANETVSVVAKEVKNSPWAVGVFIDNEKSFGRPDSVKSHYGIVINTLGRDAKTVPTKAEFSRLM
KEKYTDVAKLNKVWHLNLASWVEFDKGVTVDIKNEEQLVDFSILLTAYADKYFSVVNAAMDKYLPNHMYL
GARFPDWGMPIEVVKASAKYVDVISFNAYKEGLRDDKWAFLSQFDKPAIIGEFHVGSSDSGLFHPGLIHA
ANQQDRANMYTDYMNSVIDNPYFIGAHWFQYIDSPITGRAYDGENYNVGFISVTDRPYIEMIEAAKAMNE
SMYERRFKK

>EWH10720.1
MTYYRTKALAITTGLLLSMSAETFAQTQTDINLNVQHSVGGVAEFDRKKFITLHATLSESGWTGEEEKLQ
YMMDELNVYFGRDNGSIMWNLNQATEDPERPGYVDPTYMATKGKQARINAYGKQLADRHQFDDHNDVMVG
GQTSSFWLGRSTKPCCGKEGWEIAGADAIGEYVGHFLNEFYRNDGEDPSMGQARPKLFEVLNEPLYELID
GEADSHVTPLEVFEYHNDVATAVRKFNDSTLIGGYTTAFPIFEERDFARWDERMKLFMDTSGEYMDFFSI
HIYDFNNLGNDGKVNFKGGRLEATFDMMEHYSALRLGEVKPLVISEYAGRDHKLEKNYWSSYRDWQSIKA
ISPLLLSQMDRPNLILKSIPFINVKAIWGTADGIPYNWRLLRQENENADGLSGDWVFSDVVKFYELWSDV
EGTRVESRTTNPDVLIDTYVNDNKAYVILSNLNYSPETVYLNLFGENDNNISQIKVKHLHLSGGKVVLDE
TTQSTSLESFTLDTEATAIIEYTFDSALAINQTAEENKYYAQSYNQQIIANQSIQFNINDVVTSQYGDAT
LRLGIGRDLDKSRQPIVKVNGVQIDVPTNFSGDDQQNRPAFFSLLEIPVPYNLLTEENTLEVKFNDDGGR
VSSAVLQVMNFSADIRAKTSAVTGVAISHHGHFLATGETVQIEGTALPFFANNTQVTFSVDNPAVASISS
DGLLTALNPGQVIVTATSVEGGLTAQSTITVEQPAEASFSFDDINKYRTAEYTAGETLDVTTHFEAGTGS
TVSDKFGGVQYLLRELTSSWQVVKDIAVTDYSAVGQQRGTSSVNIPLHGVTATENLPEGNFYFLFIRFAN
TEGVTKTVQLARLNIKPDPNVVPAMLSLDDPSIYQTSSYVANSTLPVTVNFEAGTGETVTNQFGGVQFFL
RLIDTSTGSWQVLQDVTISSADAIGQQNGQATANLSLAGLTPTADLPTSQFYFLWARFNSSDGNNYSIGG
VSPIEIIEPSIAPSVTFDDPTKYSSTTYLTSGSMDLTTNIEAGSGNVISEKLGGVKVFLRELRPNWTVVK
DVIVYDSSVIGEQTVSSTVSIPLGGLTPTHQLPAGNFYFLYVQFASSNGQTYNNGIARLNIDSDFDNDGI
GDLLDTDDDNDGTPDATDTFPFDAVYGVLGDFDGDQDIDRKDISFFVRALRNPSLIRPEFDFNGDGKVHQ
SDVSRLRNLCTRPRCAE

>EWH09658.1
MQKVNKNILYALGMSGILTACSQTSNHASSESMLWDFEQAEHLKHIQTENAKLTSVKQGDNSAVKISFDS
KNNHVSGFEFKSDNGWDWSNDSSFAFALDIQNPKNTSVHMYVTAKDKAGKTHNRSFVVPANSAETYFIEL
KGLDLNQETGIRSNPPSWQSDYVPIIWRHGSKNIDISQITSIKFNIRGVLEDKQLVIDNARLIYPKALDK
NYLTKLVDKFGQSTRIDFPNKVNSTAELIERHQQEQAELTGKNFADRSKYNGWKHGPKLEATGYYRTEKY
QGKWTLVDPEGYLFFSNGIANVRLANTSTITGYDFDHSKIKARAPGDLTPEDSLGLNPAPQQAWPTRHVS
SELRANMFSWLPEYGSELADNFGYRREVHTGAIERGETFSFYRANLQRKYQINDDAELMQKWRETTVKRM
QSWGFTSFGNWIDSKYYDMQQYPYFANGWIIGNFKTVSSGNDYWSPLPDPFDPLFKERTDITIKQIADEV
NNSPWCVGVFVDNEKSWGMMGSVASQYGIVINTLSRPAIESPTKQVFVDELTKKYRTIEQLNIAWHTNIS
SWDELTQGVKLTQYPAGMLADFSHLLFVYAEQYFKIVSQSVERYMPNHMYMGARFADWGMTPEIRAAAAK
HADVVSYNYYKEGINDDFWNFLAEIDKPSIIGEFHNGALDSGLLNPGLIHAESQADRGRKYQEYLYSVID
NPYLVGAHWFQYIDSPLTGRAYDGENYNVGFVSVTDIPYQPLVDAVKEVNKNIYPRRFSQDIYQIHAPEA
LNYLDVNYGYEELADGFKWAEGPVWVDDSSSKNGGYLIFSDIPNNKIIKYQAGEGISTYLAPSGYSNGLA
IGNNKQLLIAQSRSRQLAIMNAPTNKAKAQYSALATHYQGKRLNSPNDLTVHTNGTVFFTDPPYGLPKQM
DDPAKELDFQGVYKLSPQGELKLIDKSLTAPNGIALSPDNQFLYVAVSDPNEPAWYQYKLNSQGDLVSKK
LFYKPKATGTNAHGAPDGLKVHSSGTVFATGPHGIWLFSPSGKVLAQINIPYFSANLAFDSNEDYLYITA
HEKLLRVKLK

>EWH09648.1
MRKLPVAAVISAILLSGCDSGKPDTPTTQVQSEQPLAVIWNGSDNSKVDIESWGAETQLLDKSVKVSYQS
DHYRPSVKFHPEKPWDWSKFDEFNLAVDIKNLSDESIQVYLSLTDEAGVKEDMEITGSRARSLNRAANLA
KGEQGTYFLIIKGKFVDTNAGVREKPYPWKTDEEMFVTRFGKKLDLSRITEVAIFVRGNLHDKQIEVSNL
RIRKNPEYDNSYLTALADEFGQNAKQDFPIKVKSVEELKAAADKELAELAKGGLMANRSKYGGWKDGPKL
KATGYFRTEKIDGRWWMVDPEGYIFFSHGPANVRMANLTTLTGVDFKDDSVRYIDPTRVTPEDSMGIVRV
SDEVRKTRYIASELRNNMFTWLPSYDDPMAKHYSYRREVHKGPLTSGETFSFYRANLERRYGENFEETWQ
QVTLDRMNNWGFTSFGNWVDPAFYPNEQVPYFANGWIIGDFKTLKSEHDVWAPLPDPFDPEFVRRAKITI
DVIAEEIKGSPWCAGIFVDNEKSWGLPEGTVEQKYGVILSTLTAKDADSPAKTYFTEYLKGKYGSIAKLN
QAWNFNADNWASIEQGVHLHEPYSADAVADLSYMLEAVSDRYFKVVHDTLAEALPNHLYMGARMANFGMP
KETIKASVKYSDVLSFNIYEEGVQPEEWGFLTEFDLPVAIGEFHIGSTTDTGVFHAGLVQAHDHKDRAKM
YLDYITSVAAHPNMVGAHWFQYIDSPLTGRAFDGEPYNVGFVSGTDIPYPEMVDAIKSFMSTVYEKRFAG
EFDDERYQMKKD

>EWH09595.1
MYTTRTQHTKLASLIILALSLSACGGSSESDDGNDSPTTPVEQTDTTPNTFNFSAQNNVALSTQITSASI
TITGINTKTPISISGGEYAIDNGAFTSAQGEIENNQSIKVRLTSAEHYNKTTSATVTIGGVTSKFDVTTE
QFGGVQVDINLDTKHSLNGFDSFDRQKYITIHATQTEPDWGENDGHSANAANKDPDLMLNFVNNYDVYYG
RETGGNKWHLRNVQEDTSKPGFVDEASMAKRGSDTKWGYSNFSKKINQDARAAEDRGLDLIIGAQQHPYW
PEGTLVNAIGSNDWAFSTTDTSAEPLGTATGHYLAHYLAKFFKQENDTQGQPKPKYFEIMNEPLYDLTTV
RSGSDRVDPATIFEFHNTVAAEIKKLPENSDILVGGYTVAFPDFDKDNFQRWFDRDKQFIDIAGANMDFY
SIHLYDFPCFRNSERYRKGANAEATMDMMEHYSRIATGTMKPYVISEYGAAIHCLNKAGWSPERNTYQLR
AVNSLLMQFLERPDVIAKTIPFIVVKAEWGRTDYPYGPRLMIQEFEKTGDPTQTDWVYSDLVKFYQLWSN
VNGTRVDSKSSDLDIQVDAYVEGKKAYVILNNLEFDDANIKLNTLGLNGNQVASVKIKHLETLAGAEMQS
NLTTLDSQTLPESVTLGKEATMIIEIAYTQDVQISEEVEEKKYYATEYLKTIEADTQIEYTINDVAIDTN
GEAVLRIAVGRDHGLSLTPSVKVNGTQVEIPNDFRGYNQQQGSTMTGRDNFFGVLEIPVDYALLQASNKI
SLTFADDGGRVASASLQVFNSSKQLTRSF

>ACO78156.1
MIRPLVPLLFACCLVPLSARAADEQTLFNFVRPTDVVQVKGEGAFLPELTAETTAGGDVLRRVTFNPQER
PSLRLTPQQGNWDWSSAGAVSLRIQNAMDWALTLQVSIESADGKVLRSQVALPAGPAQTLVLPLRVSSPR
AHGMRAAPPMPWTHENQRLLVATTLEGEIDPRQVQAVSLSLERPDVQQSILLGRFGVREDLEPAAYRGIV
DAYGQYSRGDWPEKVNSDKQLKTAAEQERAQLDRWLVGRPELDRFGGWLKGPQLEATGFFRVARHEGRWY
LVTPDGHPFFSLGVNTVSSGNSRTYVEGREEMFLALPGKEEPLGAFYGAGDSRQATGANDGRQFAHGRWY
DFYRANLYRTYGQSCKPPVEQAAEPERVLPAAPMEEEPDNVQVAPEGGTPAPGQPAPAKQPTAAPPCVVQ
FFDALRWRGHTLDRLQAWGFNTLGNWSDLSLGAMHRIPYTIPLLIRGDYATISTGHDWWGGMPDPFDPRF
AMAVERAIAIATRDHRNDPWVIGYFADNELSWAAPGTDPKARYALAYGTLRQTTDMPAKRAFLKLLRDRY
RNQQGLSAAWGIELPAWELMEDPGFEAPLPSPEHPAIEEDLQRFQQLFADTYFKTIAESLKWHAPDHLLL

GGRFAISTPEAVEACAKYCDVLSFNFYTREPQHGYDFEALRKLDKPMLVSEFHFGSRDRGPFWGGVAEVY
KEEERGPAYAHFLERALAEPFIVGMHWFQYLDQPATGRLLDGENGHIGLVGVTDRPFAGFVEALRKANLK
VGKAFEPVATPAAGQLKTEGGAPAAAPARTQ

>AEX22367.1
MTPTINDVVRHSEHDSSISLFDFSTQDIPAEFRFNNIDASMTLESKLKIHCHSAENMYTSVLIEPVDGDK
WDWSQMPNFCFAFEAKNLGSRSTQVFINIFDSKGQMHSRCVNVTGNTSNSYLIELKGDYLKGNTNHYSGF
RSPPAPWDSPFVYATWMWGVMNIDLSDIVQIELSIHGTLIDHKLELSNFRLMLSPEYNPNYLSQIIDCYG
QNANCEYPEKVYNDEELKLFTERELQDLKQGAMAGRSRFGGYIQGKRYEATGFYRTEKVDGKWSLIDPEG
YLYFATGIDVIRLANSYTQTGIDYDHSKVEQRSPDDTTPEDSIEKFEVSMEAKKTAFVGSTVRRNCFQWL
PSYQEDLGEHYAYMRENFEGALEQGETFSFYAANLQRKYGKDYMQKWREVTLDRMLNWGFTSLGNWTAPE
FYSNEKVPFFANGWIIGEFKTVSSGDDFWSPLPDPFDPIFKERAIATVKQVRAEIKDTPWCVGIFIDNEK
SWGRMGTIQGQHGIAIHTLSRDAQESPTKAEFMKVLKKKYLSIEALNICWNTDIASWDALSSGVNGLKHN
EAQLEDYCLLLETYASQYFKVVREALKSELPNHLYLGCRFADWGMTPNVVRAAAKYCDVISYNYYKEGLH
PQAWQFLAEVDMPSIIGEFHIGSKDTGLYHPGLVTAGNQQERGEMFEAYLHSVIDNPYFVGAHWFQYVDS
PITGRSYDGENYNVGFVSIADIPYKPMIKAAKRLHSCMYKRRYK

>AEX22317.1
MNKTKIALILSSLLFGAYGCQSTTGSQPETSGESEEQASIAIPDFESDSFFNNVQASHAKSEKVTGVGVT
SGNSALKVTFDAVSEANKFKYWPNVKFFPEGGMWNWNNRGSLKVDVTNPSDTPATFIFKIVDNVGQMGAA
TNQLNYAVTIPANSTENVEMLFNGGKRGLDGYWDGQNINLRKLAELQVFVQGPMDEQTIILDNLQLVDAT
GDFISAEEQVVEAGPIPTLKSITNFESGEKHFISDRSVSTTIEKVKSDGGLGLQVKFKADNDYPNITFMP
NRPWDWSKEGNFNLAFDIENPTNDPIQMFVRVDQAENKNWGGTADGVKDSMSNYVTLLPNETSTYYMSLS
QLEGHIVSGMRSEPPKKAYSAQAISYGWGETSLDLSNIVSIQLYLQNPTKDATIVVDSIRLVPNLDADTT
RYEGLVDQYGQFTGSDWAEKVHNDEELRASGKAALEHLGKAELMSDRSKFGGWNAGPRMEATGFFRTEKL
DGKWTLVDPEGYPFFMTGLANIRMDDTVTITGADFDNPETKQGRQIASQLRESMFTWLPEYDDQLAVNYD
YAGYVHSGALKKGEVFSFYRANLMRKYETSQQEAMEIWRDVTLDRMQDWGFTTLGNWIDPMFFGTERVAY
EAHGWIMGEHKRISTGNDYWGPIHDPFDPEFKQSTRVMAEGLAKQVEKDDPWLLGIFVDNEISWGNVMNE
ANHYGLIVNALSYDAKESPAKAAFSTYLKEKYTTINALNQAWGTTQVNSWEEFDKSFDYRSSLKPGMKRD
YSDMLTMLADKYFSTVDAELERVLPNHMYLGARFADWGVTPEIAKGAAKYVDVMSYNLYANDLNDSKKAH
FRKWLAELDKPSIIGEFHFGSTDTGLFHGGIVNVANQTERAKMYTNYMQSVVDNPYFVGAHWFQYLDSPA
TGRAWDGENYNIGFVTIADEPYVELIEAAKQFNANLYNNRFK

>AEX22316.1
MNVNKTLTALLIVGMAAPTLASTLVTSFESSDYSAGENNPEWLQIVGDQSSSYSLVSDGVTDGEQALKSD
FSAEFEPVIIWNWGTWNWSQHNVMTVDVTNSESTDTVFAIKLVDNDIQNDWGDSTQTSLDYFTAPAGETT
TFTFNLDGGNEYQDHGVNFDKDKVLAIQFMLSNNESKSLHFDNVRVSDGTTEPTPPPTDPEDMSGSVSSA
PVKTLLQLETFEELTDSIEATGADIELIDEGVSIGETALKATFNNGWSSVKLNGNWDLSALGEHLAIAVD
VTNPTNTGLFLYSRIEDNSGNEASGIARGKYIPAKSTQTVYISVKDTPAIGDIVNTLGLRELPAKSLDEG
WGDWLELNLAAIQSVTFFIPDLAEGTTDHEFIFDNARVIKDLNDVSAYEELVDSLGQNNQHDFYAKLESR
EKLDSLGARETQLLGKLLNRSQYGGAPAGSSIVADQDCKLANPATFNVCKTADGKWYLVDPDGNAFISTG
LANIRMTDTYTFTGESSTMPSDVRKSMFTEIPTNHRKEMGPVHSGPVKQGEGVSFYANNIDARHGGEEAW
QDITIKRMQDWGFTSLGNWTDSAFYAKAAAANMPYVANGWVLHHETSENPVNRIGAGYWGPIADPFDPNF
ALAAEKMAAKIKEEVTGHEATLMGIFVDNEISWGNCLNGDDAACYGQTLAAMNTDASTSPAKAAFIWFLE
NGFGQSKTIEAFNTAWGTSFASWSELGGAQSFEYSAGMLDDLKSLNWQFANKYFEVVKQAIKAEFPNNLY
LGARFADWGRTPETVDAAKVHADVVSFNIYKDSITSEDWQSDVLEQIEDLDFPAIIGEFHFGALDSGNFA
TGIVSANSQKDRGDKYITYMESVLANDNFVGAHWFQYLDSPVTGRAWDGENYNVGFVNVTDTPYSHLTDA
ARLINCELYGDDCSALEANTETRSARDVGSLYNGRNIGITSGQLNGLETIEGIDPEEPVDPPTDPDEPVD
PPTDPDEPQLRTGGSLGAFFVSIIAVLGWVRRKYVS

>AEX22315.1
MRFNKNTIALAIIASTLAAGSIAATKTPDTSNNETTQQDMSSSVQPVVMTDDGFLSKTANTHTSYNVAGE
NLEIVFDAISESEANSKWPNMKFRPESGSYDWNTKGGLQLTLENPGDREVRIEMKVADNIGIMGATTHQL
DLPIYLPAGKTTTVDFLFNGAEMNIEGYRGGSELDLRNIAEFQFYAVGPIGEQKVVVHDIDFIERTGDFV
LSEARQGQVIEAQIPTILTVTDFESGVEGIVEHHSGSNVDIVKSDTGSAIKVHYTTDDDYPTIKFSAGKD
GQAWDWSKYGDVALAFDAKNIGDSGMQLFVRVDDALDEKLGGTATGAVNSRTGYVQIPANSEDKYYFTFK
DLAEGLDSGMRGEPPKKSFSASQVVFGWGESELDLSNIVSVQLYMMNPQEEATLVIDNLSLIPNLSTDTT

RYANLLDEFGQYQEETWPEKITDLDQLKAQAKVDKKLLKNASLMSDRSKFGGWAKGPKLEATGYFRTEKV
NDKWALVDPEGYLYFATGVDNIRMDDSYTVTGMDFADAVEADTKGMRPSQVAAERYIDDKRERVEASTLR
RGMFDWLPDFNDPLADNYSYTQMVHKGPLKHGEVFSFYSANLQRKYDTDSAQEAIEIWKDVTLARMQDWG
FTSLGNWSDPMYRKNGKVPYTAHGWITGDHQRVSTGNDYWGAMHDPFDTNFRVSVAIMAKELGEEVDNDP
WCIGYFVDNELSWGNTVNNTNHYALAISGLRASTKESSAKAAFDALLKAKYGSIKKFNAAWGVDVASWDA
FAKGFNYQGEYTETVKADLSVLLDAFADKYFAVVSEEMEKALPNHLYMGVRFSDWGITPEAATAAARYVD
VMSYNLYATDLNAKGDWSRLPELDKPSIIGEFHFGSTDSGLFHPGIISSDNQQGRAQSYAKYMESVIDNP
YFVGAHWFQYMDSPVTGRAWDGENYNVGFVTVTDTPYEPLVKSAKEINRNLYNRRFGSINK

>ABJ08540.1
MRRTRWGGLADGQFEASGFFRVDQQDGIHWLVDPEGGRFLSKGVNNVRFDQDHIRNTDKIPYAEACLAKY
GSRNNWRAAAADRLAGWHVNTIGCWSDEVVAGAGRVPLAMTPIVDLGASFWLHRRGQRFPDVFDAGFEDH
IQHRAKDLCTPRRNAPQLLGTFIDNELYWSPDWRGTDELLTTFLNFPVRRAGRVTAITALQQHYREFEQF
NAVWRTPARSWEALHMLRQIEPPFVRTPPGPLYEAEEARANGLDRRRAAFAADCEAFAAVVADRYFETCV
AAIKAADPNHLVLGSRFGALPHEGVVAAAGRHLDVISFNCYAFDPSPLIDAYAVTGKPCMITEFAFRGDD
VGLPNSMGGGPRVADQIERAARFRHYARAAVSKPTLVGYHWFEHADQPREGRFDGEDCNYGTVNIEDEVY
AELTDAISEVNAAAEWLHAGWDDAPPLG

>ABG41155.1
MNTSPKSAGFILSAIALAVTALAGCNNNQATNNSVATPSDAPTRQTQQNVLPWDFAGVTDMQDVTLTAAD
MHVVDVDGEQKLAIDLHAKEHKSAGFSFIPDSPWDWSHEGQFAFAIEIENPSQSSTHLYVSVKDATGQSH
NRSFAVPGHSQDTYFMALNDPDLSIETGIRSNPNNWQSEFTPMIWRYGTKKIDLSQVKSIEFDVRGVPED
KHLIVDNPRLIKPQKIDQNYLVGLVDEFGQNDKLEFTNKIDTVEQLRALNAKEQSAFTHDVPKGRSKFNG
WADGPKLAATGYFRTEKYQGKWTLVDPQGYLFFSNGIANVRMSNTSTITGYDFDSQFIKQRAQGDFTPED
SIGLNRAPKAAWPSRHVTSELRANMFTWLPSVDEPLAEHFGYRREVHTGVVDKGETYSFYSANLARKYAS
NDPKVFMPKWRDTTVDRMLDWGFTSFGNWIDPSFYQLNRIPYFANGWIIGDYKTVSSGNDYWSPLPDPFD
PVFKQRAMVTANKIAQEVQNNPWCVGVFIDNEKSWGQEGSIERQYGIAIHTLEVDAKDSPTKAQFVTYLK
NKYSDIAELNGKWNTQIRTWDDVATGITLTRFNDPIIEDLSAMLSLYAEKYFAVVHDAVEQAMPNHMYMG
VRFADWGMTDEIRNAAAKYADVVSYNYYKEAITDQAWGFLAKIDKPSIIGEFHNGALDSGLLNPGLIHSA
SQADRGKKYQEYINSVIDNPYFVGAHWFQYIDSPLTGRAYDGENYNVGFVSVTDTPYQPLVDAAKAVNAN
IYTRRFGDAQPK

>ABG41153.1
MKYPQFITKVAFRASAIALAITLTGCSHDQHEIAQAQGVPQDLAGNGSPLGSAEDAFTNEQKIVSLIDFD
NQEQLNWLQQSDTSFAQITSSESLIDKQLAVEFAKTGNISTLRVEPPKPWDLREYENYNIAFDVQNTSPD
SVHLYLSLENVNGQIQSHSISLAPNYKGTVYFPLDGIEAETDSGMWGDVPHWQTQDDLMVWRSWRKAEQD
YSQLTALNFFTIGILENKSVILDDVRLRANPAHDPNVSVGLIDKYGQNAKQSTPLDVHSDAQLKQQADEE
LAKLAKSSGMPDRSRFGGYTKAPKREATGFFRTEKVDGKWWMVDPDGYLFFSHGPANVRMANMSTLTGID
YDQPSIRVRTSDEITPEDSMGIVSVPDSAKEKRYVISKARNDMFQWLPSYDDELSEHYSYRRSTHKGPIP
YGETYSFYRANLERRYGDDDLRAGEGEKAGENQGSDPSYVKKWHDVTAQRMHDWGFTSFGNWVDPAFYQS
EQVPYFANGWIIGDYQTLSGHTNHWGLMPDPFDPVFAQRAKVTIDAIAENIQASPWCAGIFIDNEKSWGE
REGTVSQRYGVILDALSKNIEKSPAKTAFAAHLQQKYQTIDALNSAWSSHFDSWQMFEDDASVSTHTDAQ
IADLSKMLEMLGEQYFKVVHNTLEAALPEHLYMGARMANWGMPDEIITASLKYSDVLSFNIYEEGVQEDY
WKFLEEVDLPVVIGEFHIGSTTDSGMYNPGIVHSANQQDRAQMYKDYMQSVLDKPYMVGAHWFQYIDEPI
SGRAFDGENANIGFVTVTDIPYPHMIQAVKEVTSTMYQKRLAD

>ABG40489.1
MNASKKLLFVAIVTALTGCSASETSDEVSHSSAAEVQPVDHIEQQVKTLKTLFTAKDSDSLKQVKYTNAS
GTVVGTSGDSLKVVFHGKENINSAVEFIPDVAWDWSDLDDFNIAFDIGNEGEHSVQLFLNISDTNGDTYT
RSVSVPVGPQSTYYAKMAGHDLAKSISDDKNEFNFTSGLRSNPDTWHSDDKQFISLWGKKNLKLSGISKI
SLSVQNNLFDKQITINDIRLRQNPPMDTLYLTGVVDQFGQNAKREFDGKVHSLEELYSARDKELKTLDGK
WNAPRSKWGGWLNGPKLEGTGNFRTAKYQGKWSLVDPDGYLYFATGVDIIRLANSSTMTGYDFPPEVLVK
ADNADVTPSDSQGLNRVADSAVPSRFVASELRKDLFTWLPSYEEPMGQHYGYRTGVHSGPLKQGETYSFY
AANLDRKYSEMTPDYMQKWHDVTLDRMRNWGFTSLGNWTDPAFYDNQKVPFFANGWIIGDYKTVSSGDDF
WGNMPDVFDPTFKERALHTVSVIAQEVKNTPWCVGVFIDNEKSFGRSETPQSRYGIVFNTLKRDGSEVPT
KAAFTTMLKDKYASIEALNAAWGKNISSWSEFNKGIDSVLATDETHAEQQLADYSDMLYAYAEKYFSTVD
AAMQTYLPNHLYLGSRFADWGMPMEVAKAATQYVDVMSYNVYKEGLHPKGWGFLKEFDMPSIVGEFHIGA

TDSGLFHPGLVHAANQQDRADMYQDYMGTIIDNPYFIGAHWFQYMDSPITGRAFDGENYNVGFVNVTDTP
YAPMVNAAKDLHKGMYERRFTSN

>ABE39649.1
MTEISRTRWGGLADRKAKATGFFRVEPIDGVWWFVDPDGGRFLSKGVTAVNFDHDNIKDTERHPYREACL
RKYGSRDAWRGAAAGRLASWGFNTLGAWSEPEIAHAGPTPLASAAGVVYLATAYSDGRGWPQSDLFDPAF
ETFAQQRAREICAPRRDDPTVLGWFIDNELQWGPDWRGENELLPVILRDGAAPCSRRVAVELLRGRYSSV
DEFNAVWSSAASSWDALETGPVTPPPFTRNFFTNDRAQERHPLRARYFADCDAFAGRLAERYMAVSAAAI
RAAAPYHLVLGSRFAYVPQPDVVAATARHVDVISINCYDALPDAVIDAYAAAGRPCLIGEFSFRSDDAGM
PNTQGAGPRVATQAERAAGFSRYATAALRCPNLIGYHWFLHADQPAEGRWDGENSNYGVVTIDDEVYAEL
TQTMTALNAQAEHLHDNATMVVRIERHAVG

>ABD07731.1
MTDIPRTRWGGLAEDKAAATGFFRVAQIDGVWWFIDPDGGRFLSKGVTAVNFDHDSIKGTERHPYREASL
HKYGSRNAWRSAVADRLHRWGFNTIGAWSEPEVASAGCAPLASAAGVVYLATAYSDGRGWPQSDPFAPAF
ETFAQQRAREICAPRRDDPSVLGWFIDNELQWGPDWRGENELLPVILRDNAAPHSRQVAVDLLRRRYASV
AEFNTAWRCSASSWDALATVPIAAPPFTRNFFTHDHAQERDPLRGRYFADCDAFAGLLAERYMAVSAAAI
RAAAPHHLVLGSRFAYAPQPQVIAAAGRHCDVISINCYDALPDAVIDAYAECGRPCLIGEFSFRGDDAGL
PNTQGAGPRVETQADRAAGFARYVGAGLRHPNLIGYHWFLHADQPAEGRWDGENSNYGVVTIDDEVYVEL
TEAMTVVNDDAEWLHAGAAQVRRHIATPSAA

>AGK17502.1
MIRPLVPLLFACCLVPLSARAADEQTLFNFVRPTDVVQVKGEGAFLPELTAETTAGGDVLRRVTFNPQER
PSLRLTPQQGNWDWSSAGAVSLRIQNAMDWALTLQVSIESADGKVLRSQVALPAGPAQTLVLPLRVSSPR
AHGMRAAPPMPWTHENQRLLVATTLEGEIDPRQVQAVSLSLERPDVQQSILLGRFGVREDLEPAAYRGIV
DAYGQYSRGDWPEKVNSDKQLKTAAEQERAQLDRWLVGRPELDRFGGWLKGPQLEATGFFRVARHEGRWY
LVTPDGHPFFSLGVNTVSSGNSRTYVEGREEMFLALPGKEEPLGAFYGAGDSRQATGANDGRQFAHGRWY
DFYRANLYRTYGQSCKPPVEQAAEPERVLPAAPMEEEPDNVQVAPEGGTPAPGQPAPAKQPTAAPPCVVQ
FFDALRWRGHTLDRLQAWGFNTLGNWSDLSLGAMHRIPYTIPLLIRGDYATISTGHDWWGGMPDPFDPRF
AMAVERAIAIATRDHRNDPWVIGYFADNELSWAAPGTDPKARYALAYGTLRQTTDMPAKRAFLKLLRDRY
RNQQGLSAAWGIELPAWELMEDPGFEAPLPSPEHPAIEEDLQRFQQLFADTYFKTIAESLKWHAPDHLLL
GGRFAISTPEAVEACAKYCDVLSFNFYTREPQHGYDFEALRKLDKPMLVSEFHFGSRDRGPFWGGVAEVY
KEEERGPAYAHFLERALAEPFIVGMHWFQYLDQPATGRLLDGENGHIGLVGVTDRPFAGFVEALRKANLK
VGKAFEPVATPAAGQLKTEGGAPAAAPARTQ

>AGK13218.1
MIRPLVPLLFACCLVPLSARAADEQTLFNFVRPTDVVQVKGEGAFLPELTAETTAGGDVLRRVTFNPQER
PSLRLTPQQGNWDWSSAGAVSLRIQNAMDWALTLQVSIESADGKVLRSQVALPAGPAQTLVLPLRVSSPR
AHGMRAAPPMPWTHENQRLLVATTLEGEIDPRQVQAVSLSLERPDVQQSILLGRFGVREDLEPAAYRGIV
DAYGQYSRGDWPEKVNSDKQLKTAAEQERAQLDRWLVGRPELDRFGGWLKGPQLEATGFFRVARHEGRWY
LVTPDGHPFFSLGVNTVSSGNSRTYVEGREEMFLALPGKEEPLGAFYGAGDSRQATGANDGRQFAHGRWY
DFYRANLYRTYGQSCKPPVEQAAEPERVLPAAPMEEEPDNVQVAPEGGTPAPGQPAPAKQPTAAPPCVVQ
FFDALRWRGHTLDRLQAWGFNTLGNWSDLSLGAMHRIPYTIPLLIRGDYATISTGHDWWGGMPDPFDPRF
AMAVERAIAIATRDHRNDPWVIGYFADNELSWAAPGTDPKARYALAYGTLRQTTDMPAKRAFLKLLRDRY
RNQQGLSAAWGIELPAWELMEDPGFEAPLPSPEHPAIEEDLQRFQQLFADTYFKTIAESLKWHAPDHLLL
GGRFAISTPEAVEACAKYCDVLSFNFYTREPQHGYDFEALRKLDKPMLVSEFHFGSRDRGPFWGGVAEVY
KEEERGPAYAHFLERALAEPFIVGMHWFQYLDQPATGRLLDGENGHIGLVGVTDRPFAGFVEALRKANLK
VGKAFEPVATPAAGQLKTEGGAPAAAPARTQ

>ERG08970.1
MKKSSLYCAVSSALLLSSVFNAHAGTVIKQTSSQMCFRTAGNAVNGTQVILNNNCSGDAARFSWTSGGSI
KHDKSGLCIHPGGLVNPADGQQLVLWSGCDFDNRVKFTKTSANAIKHVSGGKCVQPTNISNGSNLVVRSQ
CGANQNKFVVEQQASSGASVSNDKVKVQFNIDTKHAVGQFDSFDRRKFITLHSSNTESDWFGNNAQSLNA
PNADPDLMTHFLEDYDVYFGRDTGNMKYQLTQLPEDSAKPGYASAATATTNGGGVKWNYTNITTAQAKTM
RKHEGRNSDLIVAAQQHPYYPDGTKIGNQNWSFSQTDTAGEPLGTALGDYMAQFLQKYFKAGTSDSLGQK
RPTYVEVMNEPLFELHDFPHNGYDKESLYDIFRLHNSVADVINANPSLNDVKVGGFTVAFPDYEKGSQFF
DNWKQRDKAFLDIAGNKMDFISMHLYDFPNFPGGPGGQHQIQYRKGSNMEATLDMVEQYMAYKWGSIKPL

VISEYGSQLQGSFGTKWTPQRDWLCLKAMSSMLMSFMERPNRIDKAIPFIPLKAEWGRISDTIPYYWRLL
RQAKEGEGETGEQWVYTEMVKFYQLWSDIKGTRIDSWASDMDIQADAYVDGKDVYLILNSLEFSPTDIDL
SVLGKGSNAVTSVNIKHLYPNAQGKPILDNSNRTTLPSSVILGSESTMIIKVSHQNNVAINNINQESKHY
ASAVVKNIAANATQFFTINNVDKGANGEATLRLGVGRPHGKSLTPVVTVNGNRVAIPTDFRGYDQKNGGL
GRERFFGVIEIPVPYNNIKKTNNIEVTFPDSGGAVSSLTLQNFKMSKRITR

>ERG08956.1
MFLNPYSVKLASSFFCLSVLAGCHNAKASDQDSTKGATPLKKEHIIFDFEQDNYSQYINTINGFSTLIKE
GGNHKLAVTLKSKSNVESDFEFINPKGWDWQATGNFALALDIQNPDEVSTHLYIKTIDKSGQFQTRSVVV
PAQSQNTYYIELKGVNLNVNSGIRSNPPSWKSSYTPIVYRGGQKNIDVSSIVKVSFGVKGLLEDKRFLID
NVRLIKPANFDTHYLKGLVDKFGQNAKLDFTNKVSSTEQLQVISQKEQINLQNSPMKGRSKYSGWKNGPQ
LKATGYFRTEKYKGKWSLVDPEGYLFFSTGIDNVRMANTSTITGYDFDQSYIKQREAGDLTPEDSLGLNP
APESAWPTRYKSSALRADMFNWLPEQNNPLADNYGYRREVHSGAIKRGETFSFYRANLQRKYQTNNNNNL
MKQWQNTTVKRMLSWGFTSFGNWIEEDYYHTNKLPYFANAWIIGNFKTVSSGNDYWSPLPDPFDSLFVER
ADVTLAKVAQQVKNSPWCVGVFIDNEKSWGMMNSDAARYGIAINTLKNNAQNSPTKAQFVTLMKNKYSKI
SKLNEAWNIRLASWDEFAAGVALTQFNEQTNEDLSTMLFHYANQYFAVVDAAIAKHLPNQLNMGARFADW
GMTPEIRAAAAAHVDVMSYNYYREGLNEDFWAFLSDINMPSIIGEFHNGALDSGLLNPGLIHTQSQQERG
TKYKKYMNSVIDNPYFVGAHWFQYIDSPLTGRAYDGENYNVGFVNVADIPYKPLVDAAKSMNKSLYERRY
NNAKN

>WP_010133188.1
METKKAAAKASAAVSHSTPLIRSVLALAITAATLGGLAACSAEQGDSAQRSGAQKDGAVSGADSNLLAED
FVLEGFDKDGVPASVQVNNGTAALVDDGAGGKALQVKLKLAEHNNAGLVIQPAEAWDWSEFSDFNLAFDV
SNDGEESVQIDVTMADKNGDFYTRGLVVPADGVARTYYAKLHGHDQEDPKAAAQNEFNFASGLRSNPPTW
QSDDIMLHSFWGKKLLDLSGITQISFGSDGSLSDRQYTIDNLRLRANPEMDKNFLSGLLDQYGQNAKVDY
VGKIHSDEQLQQVVEAELASLSGEPNADRSKFSGWKNGPKLEATGYFRTEKVNGKWALVDPEGYLYFSTG
IDIIRLSNSSTITGYDYDQALIPKRSAEDVIAEDDQPLNRVDEKAWATRELISQTRADMFEWLPGYDDPL
GNHYGYRRETQSGPLKHGETFSFYSANLERRYGETYPESYLDTWQKVTIDRMLDWGFTSLGNWAADSYYK
EERIPFVAFADIIGEFSTLSSGFDFWHPVPDPYDPRFYARAVVAAESVSEQINASPWCMGIFFDNEQSFG
RLESNELHYGIVINALSRDAADTPAKVAFSKVLKEKYGNIDALNKAWDKQVASWEAFDKGMDSSVSTDAQ
LEDYATLLFEYGNQYFGTINKAMKSVLPNHLYLGSRLPSWGMPPEIVKAAGKNVDIISYNLYEEGLVPSK
WAFLAEIDKPSLIGEFSFGSDDQGHFHPGIVISSDQKDRGRMFKNYMHSFIDNPWFVGVHMFQYMDSPIT
GRAYDGENYANGFVSVADVPYVEMVKAAKEVHEGLYERRFGGARSED

>WP_007640794.1
MKTNQLRLIKQMLAGTLAGLSLSCAASQNSPLGSVENFDSFSLNKAMENNVKASLIEGQEKKYGKALKLV
FEPREESTIELPTPAQGYWNWDYTGDLNLALDVSNPGSQSFQFWLTLMDANGRKQERSAVIDAGQSARFY
ALLTGRVANAQTGMRETPPAWQTSEEKLAWRSGDRDFDFTKVTKIIFKAYAQFETNTLIVDNLELRVNPA
QDPEYLVGIVDKFGQAAKKNYPTKIHSEKQLKAAADAELASLANAKQPEDRSRFGGWSKGPKLKGTGYFR
TAKVDGRWWMVDPEGYLFFSSAIANVRMANLETITGYDFNDASVRKIDPEELTPEDSRDIIPVGKKSLNS
RFLASPLRREMFEWLPPYDHALGEHYGYRRTVHQGVIEHGEVYSFYKANLERRYGQSSPNSYIKTWRDVT
LDRMVDWGFTSFGNWIDPMFYDNQRMPFFANGWIIGDFKVIYSGQDYWSPLPDVYDPEFKRRAHLTIKQI
GREVKNTPWCVGVFVDNEKGWGSMKNDRAHFGAVYYALSRTVDESPAKKQFTQLLTKKYSDITALNNAWG
TQIESWSSFAAGITMDELNDASRADFSMLYADYAETYFRIVSSEIKRALPNHMYMGVRIAAEWGMPVEVV
AAAKKYSDVLSFNNYREGMHPDTWEFLKDLDFPTIIGEYHIGSTSDTDFYHPGLVIAADQTDRAKMYENY
MNSVIDNPYMVGAHWFQYIDDPVTGRAYDGENYNVGWVSNTDIPYQPMVDAAKRVNKSLYKRRSKIAPIQ

>WP_007640756.1
MLACQDKVQEPKADSQVMAPAANTDAKLLFDFEQPELPAEISFYNATGTLVNSQSAAGAESSALKVKFNS
AAHEYTSVLIKPQAPSWDWSDLGEISVAFDITNPGEHSVQLFLDAQDTHGTSFTRSVSVPVGTSRVYYAK
LSGHDMVSANPDSKVELNAASGLRGNPPTWEGDDVQFIWMWGVMNLDVASIQQISLSVQYALHDKEIILD
NIRVIKNPPMNKNFLTKIVDKFGQPAKVDFPGKIHSEAELAQVTEQELKDLKNGQPLADRSTFGGWKNGP
KQPATGYFYPKKVDGKWWLVDPEGYLYFATGLDIIRLANAYTMTGYDYDASTIEQRSADDLTPEDSKGKI
LISEEAQKTRHLVSKTRADMFEWLPKHTDPLGNHYDYNRDAHSGPLLKGEAFSFYSANLERKYGETEPDS
YLRQWEKVTVDRMLNWGFTSLGNWTDPKFYSNQRIPYFANGWIIGNFKTVSSGNDFWGGLPDPFDPVFKE
RALATAKAIAEETKNSPWCVGVFIDNEKSWGRSESKESEYGIVLNTLTRDGADSPTKNKFTQLMKEKYVD
IAALNTAWGTSVESWDAFQKGVKTGINNDVQLQDFSLLFTQYAEEYFKIVEGALTQYMPNHLYLGVRFAD

WGMPKDVVKAAAKYADVVSYNFYKEGLTKNKWTFLAELDKPSIIGEFHVGTTESGLFHPGLVHAANQEDR
AKMYKEYMETVVDNPYFIGAHWFQYMDSPVTGRSYDGENYNVGFVSVTDTPYAPMVKAAKELHGEMYTRR
AKK

>WP_011469127.1
MITRPYTLLTLTLALLVSLSGCGGGGKSSTTDPDPTTPEPQPDTTPASFSFTATTGASLNSTTTSNTITV
SGINTATTVSVVNGQYAIDNGSFTSAEGTINNGQTLQVQVTNSANYQTTTTATVTIGGISASYSATTMAE
PTQGVAIDVNPYIRHSVGGVDSFDRRKFITIHASNTENDWFGGNDASLGFANESDDLITEFLEGYDVYFG
RDTGGISWHLSQTLEDPAKPGFADEANMTSRGNDTKGWYTVNPSEIAIKQRQHEHRNTDMIIGSQQHPFW
PDGKLTGQGWALSQTETEAEPFGTATGHYMANFLAKFYKQSESDPNGQPKPVYVEVMNEPLYDLVDAATN
PTTPEKVFLFHNTVADEIRKLNDDVLIGGYTVAFPDFDSNNFERWENRDKAFIDIAGEKMDFISIHLYDF
PNFQNTQRYRKGSNVEATFDMLDHYTTLTLGAPLPLIVSEYGAPDHALFKAPWTPYRDGLKLKALNSLLM
SMLERPDTLLKTIPFIPVKAEWGRDGVPYNDRLMRQKFEAEGETGNEWVYTDLVKFYQLWADVNGTRVDS
YAADMDILVDSYVDGSTLYLILNNLEFNDETLTLTDLGLNNNSFVSGTMRHLHTVDGNPVLSESALANIP
TNLTIGGEATIVLALNFENDIAISETSEETKYYATTYKQAITANTDISFAINNVALGDQGEAILRLGIGR
DHGLSLQPSVSVNGVDVEVPSDYRGYDQFHNGTGRPNFYGVIEIPVPYSALQTSNTVVVNFPDSTGFVTT
AALQVFNTSTSITRPMQ

>WP_016403218.1
MLFKKSNLAILVSVVLAGVSTSNVIADDTKQSSENAATSGDMTSAATPLDFTEPAVLEKITNSHSQFSVL
KKTAEQSKDGLKMNFDAISEAEAQSKWPNVKIHSKAGPWDWNTKGGLKVALENPGSEDVRIEMKVSDNIG
IMGSADNQVDLPIILPAGKTTTVDFLFNGTQMNIDGYRGGAKLNLKSIAEIQFYSVGPIAAQEVVIRDIN
FIERTGDFVKSEAREAEVIAAPIPTLLALSDFDDGSKGIVSKTHGTTITSVKRDEGKGLKIDYSADASYP
SVTFSADKPWNWSEHGDFTLALDIENIGDAGAQLFIRVDDDVNEKQGGSANGVIHSRTGYVQLPAGEAGT
YYFTLEELAKTLDSGMRGEPPKKSYQAQAINFGWGEQKLDLSNIVSFQLYMQDLQKDLSLVIDNIRLVPN
LSADTSRYEGLLDEFGQFTNEDWAEKIHSAEELQAHAKADVKLIDSAKPMDDRTPYGGWKNGPKLEATGY
FRTEKVDGKWSLVDPSGYLYFATGLDNIRMDDTYTTTGVGFTDLVLSEDLSLRPSQISQDSYVDNQDARS
VASQLRNSMFTWLPSYQDALAQNYQYSTMIHTGPLEHGEVYSFYSANLQRKYAPDSRDEAIAVWRDVTLA
RMLDWGFTSLGNWADPSFYGNQKVAYVANGWIVGDHQRINTGNDYWGPMHDPYDPEFVESVKTMAKQVAA
EVEQDPWCIGTFVDNEMSWGNTEFDANHYALAIAALRADAKDSFAKAAFVGLLEAKYAQDIQALNKAWGS
ELKSWDELAKGYVHQGDLNDALKADYSMFLADHSDRYFAIVQQQMKQVLPNHLYLGARFTEWGITPEAAN
SAAQYVDVMSYNLYGNDMSKGDWSHLAELDMPSIIGEFHFGATDSGMFHPGLVAADTQQGRAEKYAHYMD
SVIANPYFVGAHWFQYLDSPTTGRAWDGENYNNGFVTVADSPYEKLVAGAAEVNRKLYPQRYPELVK

>WP_016403211.1
MKFTKNKIAALLSLTLLGVYGCGSTPSSSDAEGAVEDVGGTIPDFESAAFFKKVKKDHAKAEVVSDQGVT
SGSSALKVNFDSVSEANKFKYWPNVKVHPDSGFWNWNAKGSLSLDITNPTDSPANIILKLADNVGVMGSG
DNQLNYAVNVPAGETVPVEMLFNGTKRKLDGYWGGEKINLRNIVEFQIFVQGPMDAQTVIIDNFNLVDAT
GDFIEASGQEVKVSGPIPTVASVTSFDEGQPTFVAFDRSAAATVTELKTDMGGLLAVKLAATNAYPNITF
KAPQPWDWSEYGDFSLAFDLESKTDEPLQLFVRVDDAENENWGGTANGVVDSMSSYVTLAPGDDGTFYLP
LGQTGSQIVSGMRAEPPKKSYNAQAISYGWGEKSLDTSNIVSFQLYLQNPTKDAEFNIKSVRLIPNIDAD
ATRYEGLIDQYGQFTGSEWPKKISEDEELETMGKLAKMSLKSTSQMPGRSIYGGWADGPKLKGTGFFRTE
KVDGKWSLVDPQGNLFFATGVDNIRMDDTVTITGHDFADKDKRSGKEVASEVRRSMFTWLPEDDDVLAEN
YDYANWVHSGALKKGEVFSFYGANLQRKYGGTFSEAEKVWKDITIDRMVDWGFTTLGNWADPMFYDNKKV
AYVANGWIFGDHARISTGNDYWGPIHDPFDPEFVNSVKAMTKKLMTEVDKNDPWMMGVFVDNEISWGNTK
NDANHYGLVVNALSYDMKKSPAKAAFTEHLKEKYWAIEDLNTSWGVKVASWAEFEKSFDHRSRLSKNMKK
DYAEMLEMLSAKYFSTVRAELKKVLPNHLYLGARFADWGVTPEIAKGAAPYVDVMSYNLYAEDLNSKGDW
SKLAELDKPSIIGEFHFGSTDSGLFHGGIVSAASQQDRAKKYTNYMNSIADNPYFVGAHWFQYIDSPTTG
RAWDGENYNVGFVSITDTPYVPLVEAAKKFNQDVYMLRYKK

>WP_016403205.1
METRLRADSAKTISEHEQSILIYDFAEQIPKAFSFSNVDAELVSEGNGITTGSQALKVTTHSKENFYTSI
FIEPEQPFDWSALPNFSFAFDVTNLGRRSTQIFINIFDKQGQMHSRSINVAGGSCKTYLNELKGEFLKGG
LNYESGFRSNPAAWDTPFHYATWMWGEMNIDLSAVAKIELSIHGTLIDHQLVLDNFRVIFTPECNPDFLK
GCLDKFGQNALVETAEKVHSEEELLAVTAKELKALEQGAMPQRSRFSGYTGGPQLEATGFFRTEKIDGKW
SLVDPDGYPYFATGLDIIRLANSFTITGIDYDHSKVAARSDDDVTPEDSKEKLEISQEAFDSAYVANQTR
RDFFDWLPSYDDPLAEHYSYMRELFEGPVDRGEIFSFYSANLQRKYGQDGADYMGKWREVTMDRMLNWGF

SCLGNWTAPEFYSNDKIPYFANGWIIGDFKTVTSGDDFWAPLPDPFDPVFRERAEATVSQVKAEMQGSPW
CVGIFIDNEKSWGRMGTINGHYGITIHTLGRSDEESPTKAVFTQALKDKYGTVEQLNQAWGTNIDSWQAV
AGGVSDLAHNEAQLSDYSMLLELYASEYFKVVNESLKAQLPNHLYLGARFADWGCNPEVVRAAAKHVDVV
SYNYYKEGLHPEPWKFLAEVDMPSIIGEFHIGVKEGFFHAGLVTANDQTERGEMFEDYLNSVIDNPYFVG
AHWFQYIDSPITGRSFDGENYNVGFVGITDVPYQPMVDAAKRVNGGMYQRRFKNAKK

>WP_013753882.1
MNASKTLLFSAIVSALAGCSTSETKDPRNGFSDTQVQQNQQGEQAEKTLETLFVASDDESLKEVKYTNAT
GTVSESPASGLTVVFHGKENINSAVDFVPQKPWDWSDLDNFNIAFDIGNQGEHSVQLFLNISDTNGDTYT
RSVSVPVGPQRTYYAKMAGHDLAKSISDDKNEFNFTSGLRSNPDTWTSEDTQFISLWGKKNLKLSGISKI
SLSVQNNLFDKQITINDIRLRQNPPMDTLYLTGVVDKFGQNAKREFDGKVHSLTELKDARDKELQTLDGK
WNAPRSKWGGWLEGPKLEATGNFRTAKYNGKWSLVDPDGYLYFATGIDIIRLANSSTMTGYDFPPEVLVK
ADNADVTPSDSQGLNRVDDSAVPARFVASELRKDLFTWLPSYDEPMGKHYGYRTGVHSGPLKQGETYSFY
SANLDRKYSEISNDYMQKWQDVTLDRMRNWGFTSLGNWTDPAYYDNQQVPFFANGWIIGDFKTVSSGDDF
WGNMPDVFDPKFKARAMHTVSVIADEVKNTPWCVGVFIDNEKSFGRSETPQSRYGIVFNTLKRDGSKVPT
KAAFTKLAKDKYITIEALNAAWGKNIESWSDFDQGIDSVLATNKAQEKQQLADYSEMLYAYADKYFSTVD
AAMQTYLPNHLYLGSRFADWGMPMEVAKAATQYVDVMSYNIYKEGLHPKGWGFLDDFDMPSIVGEFHIGA
TDSGLFHPGLVHAANQQDRADMYQDYMGTIIDNPYFIGAHWFQYMDSPITGRAYDGENYNVGFVNVTDTP
YKPMVNAAKNLHKDMYERRFSEK

>WP_013753439.1
MKYRQLTKKASFSLSAIALAIALTGCSNDQQEVAQSKRAQEEYTGKNSQQGAAQDVFANEQKIASLIDFD
NKEQLSWLHESDISLTQITSSDSVVNKQLAVEFAKTGNISTLRVEPPTPWDLSEYENYNIAFDVQNTSQS
SVHLYLSLENLEGQIQSHSISLTPGYTGTVYFPLDGIEAQTDSGMWGDVPHWQTQDDLMVWRSWRKADQD
YSQLKALNFFTIGILQNKSVILDDVRLRANPAHDPEVMVGLIDKYGQNAKQSTPLDVHSDAQLKQKADEE
LAELAKSTGMANRSRFGGYTKGPQREATGFFRTEKIDGKWWMVDPEGYPFFSHGPANVRMANMTTLTGID
YDQPSIRTRSSEEITPEDSMGIVYIPNVIKDKRYVISKARHDMFQWLPSYDDELAEHYSYRRSTHKGPIP
HGETFSFYRANLERRYGDQGEKNGVPSYVDTWHDVTAKRMHDWGFTSFGNWVDPAFYQSEQVPYFANGWI
IGDFKTLSGHTNHWGLMPDPFDPVFAERAQVTIDAIAQNIQSSPWCAGVFIDNEKSWGEREGSVSQRYGV
ILDALSKGVSESPAKQAFSRYLQEKYQSINALNTAWSTDFANWQTFDEGATFKDYSAEQVSDLSKMLEML
GEQYFKVVHNTLEAALPNHLYMGARMANWGMPDEIISASLKYSDVLSFNIYEEGVQEDYWKFLEEVDLPV
VIGEFHIGSTEESGMYNPGIVHAANQADRAKMYKEYMQSVLDKPYMVGAHWFQYIDEPITGRAFDGENAN
IGFVTVTDIPYPHMIEAVKEVTSTMYQQRLAK

>WP_013753437.1
MNTSLHSNGFLSAKGFFNANGFLSAKGFMLSAIALAFTTLSGCDQQAADNTAAPANTATEKLTAVTALPW
DFSDTKVLKSLTLTAADTRVIGENGEQKLAIALHSKDHKSAGFSFTPSKPWDWSQEGLFAFALDIENPDA
NSVHLYVSAKDAAGQSHNRSFVVPANSSDTYFMALSDPDLSIETGIRSNPNNWQSEYTPMIWRYGSKNID
VSQVKSIEFDVRGVAQDKHLIVDNLRLIKPQKLDDQYLVGLVDEFGQNDKRQFNNKIETVEQLRALDAKE
QATFSHTVPDGRSKFNGWADGPKLAATGYFHTQKYQGKWTLVDPQGYLFFSNGIANVRMSNTSTITGYDF
DQQFIEQRAAGDFTPEDSIGLNRAPEAAWPSRYVSSELRANMFTWLPSYDEPLGKHFGYRREVHTGAVDK
GETYSFYRANLARKYASDEPNVFMSKWRDTTVDRMLDWGFTSFGNWIDPSFYQLNRIPYFANGWIIGDFK
TVSSGNDYWSPLPDPFDPVFKERAMVTAKKIAQEVQDNPWCVGVFIDNEKSWGQEGSIEGKYGIVIHTLN
VDAKESPTKAQFVAYLKSHYSDIQQLNEKWNTGIRSWDALAKGVSIDKFNDPLIADLSAMLSLYAEKYFE
VVHDAVAQTLPNHMYMGARFADWGMTDEIRKAAAKYADVMSYNYYKEAITDQAWGFLAEIDKPSIIGEFH
NGALDSGLLNPGLIHAESQADRGKKYQEYVNSVIDNPYLIGAHWFQYIDSPLTGRAYDGENYNVGFVSVT
DTPYQPLVDAAKEVNQQIYTRRFGNAQSQ

>WP_010552869.1
MNKIIRCLSIFVYSTLVLSGEGVAREFDLQHDNKTILIDDFEHASNSYKLTNKNIKTTQVIKNKNKALEL
SFSTKHKFSGITLKPNKLWDLSVLGNSALFFDVSNVSDFPVMLSVNITGKDKQVQRRTIGLTTNEHATLY
FELNSQTLNVDTGLRDTPASFKTTARKMILRGAKLNVDFSQVESIAIYTETQINPTAVTVDNLRFETIPD
AKPDFLTNIVDKFGQSTQVNYPLKVSSEQQLRAIANKELNDLSKVTPRADRSKFGGWKQGPKLKATGFFR
TEKVNGKWALVDPEGYLFFSSGIANARMANTTTFTGVDYRDDAVRARDPDDVTPEDSKGLNSNLAKYQKS
AYIAYPDRRAMFNWLPQYNDKLANHYSYKRSSHLGPIQHGEVFSFYQANLERRYAQQYPDSYIDKWREVT
LKRMQDWGFTSFGNWTDVSFYNNQQVPYFANGWIIGDFKRLSSGFDYWGAMPDPFDPEFVKRANITTQVI
AQEVQNNPWCIGVFIDNEMSWGGEGSTTLRYGIVLDALSKTTANSPTKSVFSDMLKQKYKTISQLNKAWH

RNIKSWEVFNNTGVNYKKDSNFNNAMIADLSWLLTRFSDEYFKVVNHSLKSVLPDHLYMGARFTSWGTSP
EARWSAKKYADVISYNYYREGLDPMTWDMLKELDMPTIIGEFHIGSGDTGQPNPGIIHAANQRDRADMYK
TYMKTVIDNPYLIGAHWFQYIDSPITGRAYDGENYNVGFVTTTDIPYPELVEAAKQVHKSLYQQRYGDVE
IK

>WP_008136696.1
MLCCLVGLAGCNNGKISEKKPNNTVVDFKKQLVIFDFEKNNYSDNINTINASTKLVEQSGNHKLQVNLYS
KSHTESDFEFVNPQGWDWQAIGNFALAIDIQNPNSASTHIYVKTTDKSGKSQTRSVVVPGHSENTYYIEL
KGANLNINSGIRSNPPSWHSGYTPIIYRGGQKNIDVSSIVKVSLGVKGLLEDKRFLIDNLRLIKPTNFDV
NYLKGLVDEFGQNAKLNFINKVTSTEQLLVISQKEQAQLQAQPLQGRSKFSGWKNGPQLKATGYFRTEKY
KGKWSLVDPEGYLYFSTGIDNVRMANTSTITGYDFDQSYIKQREAGDLTPEDSLGLNPAPVAAWPTRYQS
SKLRAEMFNWLPKQDDPLADNYGYRREVHSGAVKKGETFSFYRANLERKYQTHNNNELMKQWQKTTVNRM
LSWGFTSFGNWIEEQYYHTKKLPYFANAWIIGNFKTVSSGNDYWSPLPDPFDPLFVDRADVTLAKVAQQV
KNSPWCVGVFIDNEKSWGMMNSDTTRYGIAINTLKNNAKNSPTKAEFVLLMKNKYSKISKLNKAWNTTLS
SWDEFAEGVTLTQFNDHVNTDLSAMLFHHANQYFAVVDEAITKHLPNQLNMGARFADWGMTPEIRAASAA
HVDVMSYNYYREGLNQEFWEFLSDIDMPSIIGEFHNGALDSGLLNPGLIHTQSQQERGSKYQNYMNSVID
NPYFVGAHWFQYIDSPLTGRAYDGENYNVGFVNVADIPYEPLVNAAKNVNKSLYERRYRNDKN

>WP_007583021.1
MQHDNKTILIDDFEHASNSYKLTNKNIKTTQVIKNKNKALELSFSTKHKFSGITLKPNKLWDLSVLGNSA
LFFDVSNVSDFPVMLSVNITGKDKQVQRRTIGLTTNEHATLYFELNSQTLNVDTGLRDTPASFKTAARKM
ILRGAKLNVDFSQVESIAIYTETQINPTAVTVDNLRFETIPDAKPDFLTNIVDKFGQSTQVNYPLKVSSE
QQLRAIANKELNDLSKVTPRADRSKFGGWKQGPKLKATGFFRTEKVNGKWALVDPEGYLFFSSGIANARM
ANTTTFTGVDYRDDAVRARDPDDVTPEDSKGLNSNLAKYQKSAYIAYPDRRAMFNWLPQYNDKLANHYSY
KRSSHLGPIQHGEVFSFYQANLERRYAQQYPDSYIDKWREVTLKRMQDWGFTSFGNWTDASFYNNQQVPY
FANGWIIGDFKRLSSGFDYWGAMPDPFDPEFVKRANITTQVIAQEVQNNPWCIGVFIDNEMSWGGEGSTT
LRYGIVLDALSKTTANSPTKSVFSDMLKQKYKTISQLNKAWNRNIKSWEVFNNTGVNYKKDSNFNNAMIA
DLSWLLTRFSDEYFKVVNHSLKSVLPDHLYMGARFTSWGTSPEARWSAEKYADVISYNYYREGLDPMTWD
MLKELDMPTIIGEFHIGSGDTGQPNPGIIHAANQRDRADMYKTYMKTVIDNPYLIGAHWFQYIDSPITGR
AYDGENYNVGFVTTTDIPYPELVEAAKQVHKSLYQQRYGDVEIK

>WP_011469121.1
MGAIGGLVKINISFIPLFVISASIFIGACNSSKLESGVDSNNISPVMLFDFENDQVPSNIHFLNARASIE
TYTGINGEPSKGLKLAMQSKQHSYTGLAIVPEQPWDWSEFTSASLYFDIVSVGDHSTQFYLDVTDQNGAV
FTRSIDIPVGKMQSYYAKLSGHDLEVPDSGDVNDLNLASGLRSNPPTWTSDDRQFVWMWGVKNLDLSGIA
KISLSVQSAMHDKTVIIDNIRIQPNPPQDENFLVGLVDEFGQNAKVDYKGKIHSLEELHAARDVELAELD
GKPMPSRSKFGGWLAGPKLKATGYFRTEKINGKWMLVDPEGYPYFATGLDIIRLSNSSTMTGYDYDQATV
AQRSADDVTPEDSKGLMAVSEKSFATRHLASPTRAAMFNWLPDYDHPLANHYNYRRSAHSGPLKRGEAYS
FYSANLERKYGETYPGSYLDKWREVTVDRMLNWGFTSLGNWTDPAYYDNNRIPFFANGWVIGDFKTVSSG
ADFWGAMPDVFDPEFKVRAMETARVVSEEIKNSPWCVGVFIDNEKSFGRPDSDKAQYGIPIHTLGRPSEG
VPTRQAFSKLLKAKYKTIAALNNAWGLKLSSWAEFDLGVDVKALPVTDTLRADYSMLLSAYADQYFKVVH
GAVEHYMPNHLYLGARFPDWGMPMEVVKAAAKYADVVSYNSYKEGLPKQKWAFLAELDKPSIIGEFHIGA
MDHGSYHPGLIHAASQADRGEMYKDYMQSVIDNPYFVGAHWFQYMDSPLTGRAYDGENYNVGFVDVTDTP
YQEMVDAAKEVNAKIYTERLGSK

>WP_015048687.1
MEKFTRGLLCAALATTLVACSNSTTAPEAHASKTIMLADFNSPTDGLQISTEGGAEFTRLESGELTVTFS
PVEKFSKLILQPSTPWDLSGRPELNLAMDVQNMSEESIQLYIGFKNAQGRATNHSVNVAAGSTKTVYVLL
AGHAAKVDLGFKHSRMPAWESDDALAFFRYGDDLLDLSALSEISLSVRGNLTPKHIRVDNIRARTNPDYP
SDFHKGYVDAWGQNAKVDFPGKIQSDEQLKAAADAELAALSKSGLMPDRSRFGGWKDGPRLEATGYFRRE
KVDGKWWLVDPDGYLFFSHGLANVRLANLSTTTGIDFKDDSVRYVDPDAVTPEDSMGMVKVSDAVRATRY
VASELRHNAFTWLPGYDDPLADHYSYRRSVFLGPVKSGETYSFYRANLERRYGETEPESYMRTWEQVTLD
RFNDWGFTSMGNWVDPAFYTNEQVPYFANGWIIGNFKTLTSEVSYWADMPDAFDPEFVRRAQVTIDVIAE
EIQSSPWCVGIFVDNEKSWGLREGTISQRYGLILDALSKTTADSPAKAAFVTALKQKYPTIEALNEAWGT
ELSGWAALSESQTFDAFPEAFVADLSMMLEMLSDQYFRVVHDALEKTLPNHLYMGVRMASWGMPDETIKA
SVKYSDVLSFNVYEEGLQPKLWSFLDEIDLPAVIGEFHIGATSDTGLYHPGLVQAANQQDRAQMYLAYME
SILANKNLVGAHWFQYVDSPISGRAFDGENYNVGFVSATDIPYPDMVKATKAFNASVYPKRYKLGRK

>WP_014232242.1
MTPTINDVVRHSEHDSSISLFDFSTQDIPAEFRFNNIDASMTLESKLKIHCHSAENMYTSVLIEPVDGDK
WDWSQMPNFCFAFEAKNLGSRSTQVFINIFDSKGQMHSRCVNVTGNTSNSYLIELKGDYLKGNTNHYSGF
RSPPAPWDSPFVYATWMWGVMNIDLSDIVQIELSIHGTLIDHKLELSNFRLMLSPEYNPNYLSQIIDCYG
QNANCEYPEKVYNDEELKLFTERELQDLKQGAMAGRSRFGGYIQGKRYEATGFYRTEKVDGKWSLIDPEG
YLYFATGIDVIRLANSYTQTGIDYDHSKVEQRSPDDTTPEDSIEKFEVSMEAKKTAFVGSTVRRNCFQWL
PSYQEDLGEHYAYMRENFEGALEQGETFSFYAANLQRKYGKDYMQKWREVTLDRMLNWGFTSLGNWTAPE
FYSNEKVPFFANGWIIGEFKTVSSGDDFWSPLPDPFDPIFKERAIATVKQVRAEIKDTPWCVGIFIDNEK
SWGRMGTIQGQHGIAIHTLSRDAQESPTKAEFMKVLKKKYLSIEALNICWNTDIASWDALSSGVNGLKHN
EAQLEDYCLLLETYASQYFKVVREALKSELPNHLYLGCRFADWGMTPNVVRAAAKYCDVISYNYYKEGLH
PQAWQFLAEVDMPSIIGEFHIGSKDTGLYHPGLVTAGNQQERGEMFEAYLHSVIDNPYFVGAHWFQYVDS
PITGRSYDGENYNVGFVSIADIPYKPMIKAAKRLHSCMYKRRYK

>WP_014232191.1
MNVNKTLTALLIVGMAAPTLASTLVTSFESSDYSAGENNPEWLQIVGDQSSSYSLVSDGVTDGEQALKSD
FSAEFEPVIIWNWGTWNWSQHNVMTVDVTNSESTDTVFAIKLVDNDIQNDWGDSTQTSLDYFTAPAGETT
TFTFNLDGGNEYQDHGVNFDKDKVLAIQFMLSNNESKSLHFDNVRVSDGTTEPTPPPTDPEDMSGSVSSA
PVKTLLQLETFEELTDSIEATGADIELIDEGVSIGETALKATFNNGWSSVKLNGNWDLSALGEHLAIAVD
VTNPTNTGLFLYSRIEDNSGNEASGIARGKYIPAKSTQTVYISVKDTPAIGDIVNTLGLRELPAKSLDEG
WGDWLELNLAAIQSVTFFIPDLAEGTTDHEFIFDNARVIKDLNDVSAYEELVDSLGQNNQHDFYAKLESR
EKLDSLGARETQLLGKLLNRSQYGGAPAGSSIVADQDCKLANPATFNVCKTADGKWYLVDPDGNAFISTG
LANIRMTDTYTFTGESSTMPSDVRKSMFTEIPTNHRKEMGPVHSGPVKQGEGVSFYANNIDARHGGEEAW
QDITIKRMQDWGFTSLGNWTDSAFYAKAAAANMPYVANGWVLHHETSENPVNRIGAGYWGPIADPFDPNF
ALAAEKMAAKIKEEVTGHEATLMGIFVDNEISWGNCLNGDDAACYGQTLAAMNTDASTSPAKAAFIWFLE
NGFGQSKTIEAFNTAWGTSFASWSELGGAQSFEYSAGMLDDLKSLNWQFANKYFEVVKQAIKAEFPNNLY
LGARFADWGRTPETVDAAKVHADVVSFNIYKDSITSEDWQSDVLEQIEDLDFPAIIGEFHFGALDSGNFA
TGIVSANSQKDRGDKYITYMESVLANDNFVGAHWFQYLDSPVTGRAWDGENYNVGFVNVTDTPYSHLTDA
ARLINCELYGDDCSALEANTETRSARDVGSLYNGRNIGITSGQLNGLETIEGIDPEEPVDPPTDPDEPVD
PPTDPDEPQLRTGGSLGAFFVSIIAVLGWVRRKYVS

>WP_011575418.1
MNTSPKSAGFILSAIALAVTALAGCNNNQATNNSVATPSDAPTRQTQQNVLPWDFAGVTDMQDVTLTAAD
MHVVDVDGEQKLAIDLHAKEHKSAGFSFIPDSPWDWSHEGQFAFAIEIENPSQSSTHLYVSVKDATGQSH
NRSFAVPGHSQDTYFMALNDPDLSIETGIRSNPNNWQSEFTPMIWRYGTKKIDLSQVKSIEFDVRGVPED
KHLIVDNPRLIKPQKIDQNYLVGLVDEFGQNDKLEFTNKIDTVEQLRALNAKEQSAFTHDVPKGRSKFNG
WADGPKLAATGYFRTEKYQGKWTLVDPQGYLFFSNGIANVRMSNTSTITGYDFDSQFIKQRAQGDFTPED
SIGLNRAPKAAWPSRHVTSELRANMFTWLPSVDEPLAEHFGYRREVHTGVVDKGETYSFYSANLARKYAS
NDPKVFMPKWRDTTVDRMLDWGFTSFGNWIDPSFYQLNRIPYFANGWIIGDYKTVSSGNDYWSPLPDPFD
PVFKQRAMVTANKIAQEVQNNPWCVGVFIDNEKSWGQEGSIERQYGIAIHTLEVDAKDSPTKAQFVTYLK
NKYSDIAELNGKWNTQIRTWDDVATGITLTRFNDPIIEDLSAMLSLYAEKYFAVVHDAVEQAMPNHMYMG
VRFADWGMTDEIRNAAAKYADVVSYNYYKEAITDQAWGFLAKIDKPSIIGEFHNGALDSGLLNPGLIHSA
SQADRGKKYQEYINSVIDNPYFVGAHWFQYIDSPLTGRAYDGENYNVGFVSVTDTPYQPLVDAAKAVNAN
IYTRRFGDAQPK

>WP_011575139.1
MLKVIPWLLVTSSLVAIPTYIHATTEVVVNLNVKHSVEGKSEFERKNHIKLHSTLNDNDWQGEEDKLKYM
MEELDVYFGRDNGGTVWNFNQAIEDPANIGYADPQNIIARGQAQRETNWGQNKSALHQYDGRGDLMIGGQ
PRAHYLGNTSPCCGGSAWQAKGGDAVGDFLGQYVNEFFRSAGDPVTKGHLAPVYFEVLNEPLYQVTDAPH
ELGLEQPIPPIDIFTFHNDVADAFRQHNTHIKIGGFTVAFPIFEQREFARWEERMKLFIDTSGSHMDVYS
THFYDLEDDNRFKGSRLEATLDMIDQYSLLALGETKPHVISEYGGRNRPMENAPWSALRDWWFLKTASPM
LMQFLSRPDSVLTSIPFVPIKALWGTAADGTPYNWRLLRQQKEAPNETGENWVFTEMVKFYQLWSDVKGT
RVDTFSTNSDFLIDSYVQNDKAYVLISNLTEQAEKIVVHKYGAPASSQPTTRIKHLYLKGAAPALDETSH
ASDIQEVTIAAEATMVIEYDYPSDIVINETSQEKKYFATEYLKPISANQISRFNINSVATSALGEGILRV
VVGRKLGKSLAPTIAVNGETLTASAQISGDIQNTRGDFFGVIEFPVPIDLLRTNNEIDVTFGDDGGHIAS
VNLKVFSFTSDVRPSAGPVKGITIEPTSAVVAVGSTLQLNPTITPYFATNQNYFLQSSAPEVATVTQTGL
VSALMQGEARITATTEEGSFIAQVDIEVELPSPTSITFDDQSIYASTVYTAGEAMHVTTEYDAGTGHTVT

AALGGVEYRLRHLTASFGLISDVAIVQDGHAVNTQRGTSSVELALPTNLQASADLPDGEFYFLFVRVVSS
NGETQSTSAFPVSIEAGNIDTSPSLTLDDARKYRDTIYKTDQQLTVTAHYQAGDGNTVTSEQGGVRFYLR
ELDANFGLINDIIIEDASAIGQQVGAATATFSLADLTPSAALPAGHFYYLFAVFNSTNGDKYNIPGVFPI
RIEQEVSELSLTFSEPNLYRSTDYEVGGSLAVSVDFDMGTGNAVSDELGGIRFFLRHLREDYSMVKDIIL
EDANAIGQQSGSASVTFSLANIAASDALPANDFYFLYVLVKSTDGATQDLAVQRINIVSPALVGDYDLDG
DVDINDIQSLIVAIHMRQSIDLSFDMNSDGTVNLLDTRLLMNACTRTRCAP

>WP_011574784.1
MNASKKLLFVAIVTALTGCSASETSDEVSHSSAAEVQPVDHIEQQVKTLKTLFTAKDSDSLKQVKYTNAS
GTVVGTSGDSLKVVFHGKENINSAVEFIPDVAWDWSDLDDFNIAFDIGNEGEHSVQLFLNISDTNGDTYT
RSVSVPVGPQSTYYAKMAGHDLAKSISDDKNEFNFTSGLRSNPDTWHSDDKQFISLWGKKNLKLSGISKI
SLSVQNNLFDKQITINDIRLRQNPPMDTLYLTGVVDQFGQNAKREFDGKVHSLEELYSARDKELKTLDGK
WNAPRSKWGGWLNGPKLEGTGNFRTAKYQGKWSLVDPDGYLYFATGVDIIRLANSSTMTGYDFPPEVLVK
ADNADVTPSDSQGLNRVADSAVPSRFVASELRKDLFTWLPSYEEPMGQHYGYRTGVHSGPLKQGETYSFY
AANLDRKYSEMTPDYMQKWHDVTLDRMRNWGFTSLGNWTDPAFYDNQKVPFFANGWIIGDYKTVSSGDDF
WGNMPDVFDPTFKERALHTVSVIAQEVKNTPWCVGVFIDNEKSFGRSETPQSRYGIVFNTLKRDGSEVPT
KAAFTTMLKDKYASIEALNAAWGKNISSWSEFNKGIDSVLATDETHAEQQLADYSDMLYAYAEKYFSTVD
AAMQTYLPNHLYLGSRFADWGMPMEVAKAATQYVDVMSYNVYKEGLHPKGWGFLKEFDMPSIVGEFHIGA
TDSGLFHPGLVHAANQQDRADMYQDYMGTIIDNPYFIGAHWFQYMDSPITGRAFDGENYNVGFVNVTDTP
YAPMVNAAKDLHKGMYERRFTSN

>ADY17920.1
MLKRHQASRKVSSSLVALLKPSAIVVLATVALASGANAANYTASNASQLSARLQDAANNGTGVDVITIQG
SIFTDQQIDIQTPVTIQGAAGFRVSRIIRTSDDGFQPLFNIQSSNVTIRNLLLIDEKGQNTNTQVASEAG
NDHSNARLINIPYEDAYQQIANITIENNTFENTAVGVASSGLIPRNLSITNNDFIKVNRSVELLRDVGRV
YNVWNVSANNVVLNGGTLNISNNRIRGNRVRLGISVDAGNDGVYVPPSFTNIPFFDAAARAQFSDKPVVF
ANGSQVNSNTVEGANEFGIALATVANVTVAGNTVSTTEDDINSSDDIENNFTAGINVEHNSRDIVVDSNT
ITVGASGNFATGINVLAFQDHHAPLNHAQASSNITLVRNIFKGTGENTILAFGFSNLVVEDNNASQFTTR
NPYQVTASFYNVPCGLSTSTARGTNNNIRYNQSSFNGTGNAPQYYDKNGNVVSGYTCQ

>ADY17918.1
MKIKFLSATIAASLALPLSAATLVTSFEEADYSSSENNAEFLEVSGDATSEVSTEQATDGNQSIKASFDA
AFKPMVVWNWGSWNWGAEDIMSVDVVNPNDTDVTFAIKLIDSDILPDWVDESQTSLDYFTVSANTTQTFS
FNLNGGNEFQTHGENFSKDKVIGVQFMLSENDPQVLYFDNIMVDGETVTPPPSDGAVNTQTAPVATLAQI
EDFETIPDYLRPDGGVNVSTTTEIVTKGAAAMAAEFTAGWNGLVFAGTWNWAELGEHTAVAVDVSNTSDS
NIWLYSRIEDVNSQGETATRGVLVKAGESKTIYTSLNDNPSLLTQDERVSALGLRDIPADPMSAQNGWGD
FVALDKSQITAIRYFIGELASGETSQTLVFDNMRVIKDLNHESAYAEMTDAMGQNNLVTYAGKVASKEEL
AKLSDPEMAALGELTNRNMYGGNPDSSPATDCVLATPASFNACKDADGNWQLVDPAGNAFFSTGVDNIRL
QDTYTMTGVSSDAESESALRQSMFTEIPSDYVNENYGPVHSGPVSQGQAVSFYANNLITRHASEDVWRDI
TVKRMKDWGFNTLGNWTDPALYANGSVPYVANGWSTSGADRLPVKQIGSGYWGPLPDPWDANFATNAATM
AAEIKAQVEGNEEYLVGIFVDNEMSWGNVTDVEGSRYAQTLAVFNTDGTDATTSPAKNSFIWFLENQRYT
GGIADLNAAWGTDYASWDAMRPAQELAYVAGMEADMQFLAWQFAFQYFNTVNTALKAELPNHLYLGSRFA
DWGRTPDVVSAAAAVVDVMSYNIYKDSIAAADWDADALSQIEAIDKPVIIGEFHFGALDSGSFAEGVVNA
TSQQDRADKMVSFYESVNAHKNFVGAHWFQYIDSPLTGRAWDGENYNVGFVSNTDTPYTLMTDAAREFNC
GMYGTDCSSLSNATEAASRAGELYTGTNIGVSHSGPEAPDPGEPVDPPIDPPTPPTGGVTGGGGSAGWLS
LLGLAGVFLLRRRKV

>EDM85054.1
MNIARLLSAVLLATLPLAAHTLPGPQLSSPTVISSVNPDELRIFTADIATSPDGRGILAWAEGGFVYLQR
LNADGSANGMRINANAVGTGEVRGVDVVMDNDANFALTYTKGQQQDAVVYFRRFLADGSPEAIEYDDQSM
QPYNLAGLQTCASQRKVATSNPAMDMDADGNVALVFDGATYCASTIERNQTIYRYLPKSASASAPLAVAS
VNVTDNIARTRLSAVDIQGSLVTIATTGLPSGRQQISLSRYNGTTQQSTVQVADFQSNVAGPPRFAVHRS
ASGTATVVWSASTFNAQTSSFSAPSVRMRSFDANNTPLTDTPVELISNSTLLGLSTEPDGDYAIYTRRQN
LDLWSIFRFSDAGVAGETRQGTNVYNNGYWQTAPISISPTLSGNNYVVLYNRSDSDDSTLLHVLRYGGPQ
GTLKLDMQLSRNPIIAGAGDQIALRWISNAIGPFSNGTCQASGNWGGFVAPSGVRDLGLFTVAGDRTYSL
LCGPAMEGLFQSVTLNVQNAEQELATPTINFTATPANLQGNATSTLSWSTTNATTCDATGEWSGSKAVNG
SETVGPFATAGSRTYTLTCRNQGGPFATQSVDVTVASDTTPDTFSFTAVTGQTPSTNTASNTVTIAGLNS

PAPISITGGTYSNNGSQFTSAAGTISNGQTLQLRTDSPAEPGETAVVTVTVGDFSTSFTVTTRMPDGAST
AMVDDVDNNPVEFMSSEGTLNNLRTVATPMGAPTTREYPNGFFAFNLDNVTANSTVTVTITLPADARPTS
YVKCNADGSSCAEFGGATFNNNVVVLTLTDNGAGDNDPREGFISDPGAPAVTPAAASGGSSVSSGGGGGG
SLNAMLLPLVGLLLWRVRRKAS

>ADD60418.1
MKQLKLLIGSTLFMSITSVQAADWSPFSIPAQAGAGKSWQLQSVSDEFNYIAQPNNKPAAFNNRWNASYI
NAWLGPGDTEFSAGHSYTTGGALGLQATEKAGTNKVLSGIISSKATFTYPLYLEAMVKPTNNTMANAVWM
LSADSTQEIDAMESYGSDRIGQEWFDQRMHVSHHVFIRDPFQDYQPKDAGSWVYNNGETYRNKFRRYGVH
WKDAWNLDYYIDGVLVRSVSGPNIIDPENYTNGTGLNKPMHIILDMEHQPWRDVKPNASELADPNKSIFW
VDWLRVYKAQ

>AFX00030.1
MLKRHQASRKVSSSLVALLKPSAIAVLATVALASGANAANYTASNASQLSARLQDAANNGTGVDVITIQG
SIFTDQQIDIQTPVTIQGAAGFRVSRIIRTSDDGFQPLFNIQSSNVTIRNLLLIDEKGQNTNTQVASEAG
NDHSNARLINIPYEDAYQQIANITIENNTFENTAVGVASSGLIPRNLSITNNDFIKVNRSVELLRDVGRV
YNVWNVSANNVVLNGGTLNISNNRIRGNRVRLGISVDAGNDGVYVPPSFTNIPFFDAAARAQFSDKPVVF
ANGSQVNSNTVEGANEFGIALATVANVTVAGNTVSTTEDDINSSDDIENNFTAGINVEHNSRDIVVDSNT
ITVGASGNFATGINVLAFQDHHAPLNHAQASSNITLVRNIFKGTGENTILAFGFSNLVVEDNNASQFTTR
NPYQVTASFYNVPCGLSTSTARGTNNNIRYNQSSFNGTGNAPQYYDKNGNVVSGYTCQ

>AFR90184.1
MKTTKCALAALFFSTPLMAADWDGIPVPADPGNGNTWELQSLSDDFNYVAPANGIPVPADPGNGNTWELQ
SLSDDFNYVAPANGKSTTFYSRWSEGFINAWLGPGQTEYYAPNSSVEGGNLVIKATRKPGTTQIYAGAIH
SKESVTYPLYMEARTKITNLTLANAFWLLSSDSTEEIDVLESYGSDRSTETWFDERLHLSHHVFIRQPFQ
DYQPKDAGSWYPNPDGGTWRDQFFRIGVYWIDPWTLEYYVNGELVRTVSGPEMIDPYGYTNGTGLSKPMQ
VIFDAEHQPWRDEQGTAPPTDAELADSSRNQFLVDWVRFYKPVANNNGGGDPGNGGNPDNGNGGNPDNGS
SGDTVVVEMANFSSTGKEGSAVAGDTFTGFNPSGANNINYNTLGDWADYTVNFPAAGNYTVNLIAASPVT
SGLGADILVDSSYVGTIPVSSTGAWEIYNTFSLPSSIYIASAGNHTIRVQSSGGSAWQWNGDELRFTQTD
ADTGTNPPSSTSITVEAESFNAVGGTFSDDQAQPASVYTVSGNTAINYVNQGDYADYTITVAQVGTYTIS
YQAGSGVTGGSIEFLVNENGSWSSKTVTAVPNQGWDNFQPLNGGSVYLSAGTHQVRLHGAGSNNWQWNLD
KFTLSN

>BAK08910.1
MRKITSILLTCVMGCTATYAADWDGVPVPANPGSGKTWELHPLSDDFNYEAPAAGKSTRFYERWKEGFIN
PWTGPGLTEWHPHYSYVSGGKLAITSGRKPGTNQVYLGSITSKAPLTYPVYMEARAKLSNMVLASDFWFL
SADSTEEIDVIEAYGSDRPGQEWYAERLHLSHHVFIRDPFQDYQPTDAGSWYADGKGTKWRDAFHRVGVY
WRDPWHLEYYVDGKLVRTVSGQDIIDPNGFTGGTGLSKPMYAIINMEDQNWRSDNGITPTDAELADPNRN
TYYVDWVRFYKPVPINGNATTVELGNFHNTGKDGANVTGDTVLGFNKNGNNINYNTKGDWADYTVNLPAA
GEYRVDLVIASPMSSGLGAELTFAGNAAKTVTLSNTGGWESYQTFTLPQTISVSSPGNYNFRLKSTGSSN
WQWNGDEIRFVKL

>BAF62129.1
MSSALALAFSSAPVQAIYTPWEGSFHQYPDSYYDVDGQLVEEAYEEPEQVPGNGLSPWDPADAIKRTSPE
HQNYPLSDLANQGGWTMVEELSDEFDSPQFNEYNLMGKENGKWLPNNHIWGGRAPAVFAPKNVKQEGGKL
HLSIANDESYTPHGFWNEWYYGWTSASVQNVNPIRYGYFEVKSKVGIGSSGFWLYAETLEDKEYLSQFNE
PGHVKSKLELDVYEQSGRRSEWAPYYNMNSWVFIHQGEEMSYVSEEGNEYTAYQNGGHWKADIDFADDFH
VYGFYWGPEEIIWYFDGKPIRSMKNRYYHTPLYLLLDTETMPDWFGMPSANELDFAHQIEYVRVWTNDET
EQNWRGRYFMDPTIKTGDGLNGKPGENRVAAEYGGTEAYREKWGNPVSGSPEVVTLQGIAAKQSHSEIQS
DIPYVLVPQFTPSNVTDKQMNWLSSDPSIADINRSGMVSAKAAGSVTFTGSHADLPNQTISVTADILQIG
KRFDLELVNYGATGKEGDEVENDNVYGWGVGNGVVTFNTRGDYGDFFDVDFGSGGTYMAGISAGTDIASG
IGATIFIDGVEVLSGDIDSSGNWNVNKRTDLAGSFEVEPGTHTVRIMSSGSSGWQWNGARAHFVQVLEGD
VDPGDGGTDPGDGGTDPGDGGTDPGDGGTDPGDGGTDPGDGGTDPGDGGTDPGDGGTDPGDGSTDPTAVT
LQAQSFVATGGANGGFEVYDIAGGQGINFNQTGDYADYTVNLAAGSYSVSLYAATPMNDAGVELILNGES
IAVSSISPTGGWDNFQSSVIAANLVVATGGEQALRLISVGADNAWQWNADKLVFTPNSGNGATNPDPTPG
EVVISVEAESFADTGGSYDGFQVYAINGGSAINFNQAGDYADYLVTIEQAGTYSMSIEAGTNLANTGIEV
WVDGESQASSAITNTGSWDVFASNLVSNNISLSEGQHTIRIMGVGEAGSWQWNADKFTLTKQ

>ALO78722.1
MKHTISTLTALLLCSSSFAADWDGLPVPADAGSGNTWQLQSNVSDDFNYSAPANGKSAAFYDRWSEGFIN
AWQGPGLTDYHNPNSRVENGELVIQATRKPGTNQVYTGAVHTNDSIQYPVYIETSSKIMDQVLANAVWML
SSDSTEEIDIVEAYGSSRPDQTWFAERMHLAHHVFIRDPFQDYQPKDAGAWYADGRLWRDQYSRVGVYWR
DPWHLEYYIDGQLVRTVSGVDMIDPYGYTNGNGLSKPMQIIVDAEDQDWRSDNGIIATDADLADSSKNQF
YVDWIRVYKPVPDANGGGDNGGDNGGDNGGDNGGDNDITSSVDFDSFFATGKDGSAVAGDSVNGFNPSGN
GNINYNTVGDWAEYSINLPEAGEYRLELDTASTVSTGLGADISIDDVFVGTVAISQTGGWESYQTFSLAN
TINIGAGTHTLRVQSAGSSPWQWNGNAIRMVKVGEGSSNNQTTTPTPSEMISLEAESFNSTGGPYDGFQT
YTQSGITATNYNQRGDYAEYTLSVPTAGNYNVSAIVATPESGAAMTLTLNGNALVSLDVPSTGGWNTFTE
VNASGAVALPAGTHTLRVTSSGNTANTWEWNADRFIFTPQ

>BAO79715.1
MKKHISCCIALMLTSVASLAADWDVYPVPASAGAGKVWQLQPQSDEFNYNFSATTSAATFGGKWTNFYHN
TWEGPGPTRWMRENTSVSDGQLQIKATRVAGETKTYDVDLNLDGINEQFTSPATRAGCITSTTRVKYPVF
VEARVKIANAVMASDVWMLSPDDTEEIDILEAYGGKAARNDWFAQRLHLSHHLFIRNPFTDYQPRDASTW
YAGDGVTYWADNWVRIGVNWVSPTRLEYYVNGQLVKVMDKLNTVNGIDGIDPWNITGGKGITKEMDIIIN
MEDQNWNAAQGRQPTDAEITNTSNHTFKVDWIRVYKPVTATTSSSSSSSAASSASSNTVQLIDFANYYDT
GKSTASVSGDNYIGFNKSGGGNINYNTVGDWGDYLVTLPSDGKYKFEIITASPMTSGLGAKLIIDGIYVG
TISVGSTGGWEVYSAFALANSISIGAGTHTVRIESTGSSTWQWNGDQIRITRVGSL

>ABD80437.1
MKTTKCALAALFFSTPLMAADWDGIPVPADPGNGNTWELQSLSDDFNYAAPANGKSTTFYSRWSEGFINA
WLGPGQTEFYGPNASVEGGHLIIKATRKPGTTQIYTGAIHSNESFTYPLYLEARTKITNLTLANAFWLLS
SDSTEEIDVLESYGSDRATETWFDERLHLSHHVFIRQPFQDYQPKDAGSWYPNPDGGTWRDQFFRIGVYW
IDPWTLEYYVNGELVRTVSGPEMIDPYGYTNGTGLSKPMQVIFDAEHQPWRDEQGTAPPTDAELADSSRN
QFLIDWVRFYKPVASNNGGGDPGNGGTPGNGGSGDTVVVEMANFSATGKEGSAVAGDTFTGFNPSGANNI
NYNTLGDWADYTVNFPAAGNYTVNLIAASPVTSGLGADILVDSSYAGTIPVSSTGAWEIYNTFSLPSSIY
IASAGNHTIRVQSSGGSAWQWNGDELRFTQTDADTGTNPPSTASIAVEAENFNAVGGTFSDGQAQPVSVY
TVNGNTAINYVNQGDYADYTIAVAQAGNYTISYQAGSGVTGGSIEFLVNENGSWASKTVTAVPNQGWDNF
QPLNGGSVYLSAGTHQVRLHGAGSNNWQWNLDKFTLSN

>BAK08903.1
MRLSKSQGILPLAHAVLAAAIAYSTAATAADYRLEAEDFTNVGGTYNDGQPQKISVYTVNGITAINYVNK
GDYAEYTLSVPQAGQYDLTYFAGTAIDGARIDFQVNNNGSWQTLARTDVPNAGWDNFQPLPAGSIHLSSG
SQQIRLFGGGDHDWQWNLDKMELAYIDDSSSSSGGGSTSSSSSGGSSSSSGSGSSSSSGGSPEEGGHVSGT
FKLEAESAHHVGGEIDTYAINGGVAVNYFNSGDYLEYNLHLDQSGLYRPKYYVSTAHSSGVAVGLMATDH
EGALVTKNTSEVQSQGGWDSFYLLNAASDINLFSGDLTIRIYGAGTQDFQFNIDYVIFERISDVDLDLDG
DSDGIADVNDSCPGTDPSETANSEGCAPSQLDTDKDGIADNRDQCPTTAPGDFVDSEGCASTGADDDDLD
GIANQEDQCPDTPFGENVAPSGCTGFEDSDSDGIANGTDQCPSTPAKEFTNESGCSPSQVANPHSVKVTV
NANIKHSVKGISDFGRNRHITAHTTIYEKDWEGHADKLNYLVNTLDVTLGRDNGTATWKFQDTKEDPNRE
NWPDLDYMVTRGKELRENYEANPFYKRFSADRTELIAGTNPHPTYPTLSWNANGSTWHDWQPMHIETSAA
WMGQYLKHYYANSSNGYIGDPMPKFWEVINEPDMEMKTGKFMVTNQEAIWEYHNLVAQEIRSKLGNEAPL
IGGMTWGQHDFYRRDGISRYADNAYDQWIVADDPAEEAAAEEFFRQAMATTVDDTRDQNWYQWDVMWKGF
MDAAGHNMDFYSVHVYDWPGVNSDAKSTLRRNGHLPAMLDMIEWYDVYQNGQANRKPIVISEYGAVQGGW
NTLAHQPRFESEVLKSFNAMLMQILERPDYVIKSMPFTPAKPLWGYYPGGCGYEEVRNCTAPYHYSLLIE
PVLNSDNWQWSDYIKFYELWADIDGTRVDSVSSDPDVQVQSYVNNNELFIIINNLETVDTTIDLTVAGLN
NAQLQNVELRNMHFDNNFDTQLERHHMKQMPTKVTLAADATLVLRYTLNSTIAINQSVDEKKYFGNSVSG
GSVPHRISVAGGAKNLQVNNVSVPSGYAESQLRLTVALYPSQDDTPDSLLQIDTLTINGHTIETPIDWRG
RKENSVERYFNTLEIPVPVDVLQKNNTISVDFRHNGELTVANLVIKEYTTTPVRH

>AAT67062.1
MRPSCAFFSTPLMAADWDGIPVPADPGNGNTWELQSLSDDFNYAAPANGKRTTFYSRWSEGFINAWLGPG
QTEFYGPNASVEGGHLIIKATRKPGTTQIYTGAIHSNESFTYPLYLEARTKITNLTLANAFWLLSSDSTE
EIDVLESYGSDRATETWFDERLHLSHHVFIRQPFQDYQPKDAGSWYPNPDGGTWRDQFFRIGVYWIDPWT
LEYYVNGELVRTVSGPEMIDPYGYTNGTGLSKPMQVIFDAEHQPWRDEQGTAPPTDAELADSSRNQFLID
WVRFYKPVASNNGGGDPGNGGTPGNGGSGDTVVVEMANFSATGKEGSAVAGDTFTGFNPSGANNINYNTL

GDWADYTVNFPAAGNYTVNLIAASPVTSGLGADILVDSSYAGTIPVSSTGAWEIYNTFSLPSSIYIASAG
NHTIRVQSSGGSAWQWNGDELRFTQTDADTGTNPPSTASIAVEAENFNAVGGTFSDGQAQPVSVYTVNGN
TAINYVNQGDYADYTIAVAQAGNYTISYQAGSGVTGGSIEFLVNENGSWASKTVTAVPNQGWDNFQPLNG
GSVYLSAGTHQVRLHGAGSNNWQWNLDKFTLSN

>AIW52355.1
MKNNLLLIGCVLTSTNLLANDWDAIPLPVTPDNGKVWQLQEAYSDSFNYTGKPAAFTSKWNDTYFNSWTG
PGLTYWQQDESWVSDGNLIISASRRAGTAQVNAGVITSKTKVTFPIFLEASIKVSNLELSSNFWLLSDND
EREIDVLEVYGGARDEWFAKNMSTNFHVFIRDQQTNQIISDYNDQTHNTPSWGTYWREGFHRFGVYWKSP
TDVTFYIDGQQTPDGSWAQVIMKDKDYTGATLNKNTHNMDQSAYIIIDTEDHDWRSEAGNIATDADLADD
SKNKMYVDWVRVYKPVNAANTNSVTSGAQIKAKHSQKCIDIKNGAMNNGSIYQQWNCNSNNENQAFELVE
LTNNEYAISSQLTGLCMQIANSSTSNGAGVEQWVCDHTKANQRFTLNNTGDGYFELRSSLSNKCIDIAGK
LQTNGASVVQWQCYNGDNQRFQLIE

>AGU13985.1
MYAADWDGVPIPAPAGQNKTWQIQSVSDDFNYTASANNKPNAFTSRWNDSYINAWLGPGDTEFSAGHSYT
NSGKLALQAAEKTGTDKVYAGIISSKQTFTYPLYIEARAKSTNNTMANAVWMLSADSTQELDAMEAYGSD
RPGQEWFDRRMHVSHHVFIREPFQDYQPKDEGSWIYNEQEPWRVSYHNYGMHWKDPWNVDYYIDGVLVRS
VSGPQMIDPNNFTNGTGINKPLHIIIDMEHQDWRDVKPTSAELADPAKSIFYVDWIRVYKPVDSGASAPT
PPTGATSLQARHSSKCLDLSAGNSADGTNMQQWGCSATNTNQDITFVAKGDGYYEMKTKHNKCIDVAGKE
TTNGANLVQWSCYNGTNPQFKLLDKGNGWFQLQAKHSGKCLEVVNSATTNGANVQQWACGNGNNQQWKFQ

>ABD81915.1
MRNLNKNKVHILRAAIAASMSVLPLAAGAADYVIEAENFVAQGGTYVDGQPNKVSVYSVNGATAINYVNR
ADYTDYQINVATHGYYNVQYAIGTSVASGAAIELLVQNGSSWESQGQTNVPVGHWDSFQPLNASHEVILP
AGTVNLRVYGAGSNDWQWNLDSISLTLESAINPQPDPDPDPSPQLVKTEAEAFNAQSGTFADGQPTPVSI
YTVNGKTAINFVNKGDAVEYNLVAPAAGSYALKYSIGTSVASGSEVEFFVLKNNVWVSQGKTPVPAVGWD
NFTSVASAQTVELAAGSNKVKLVGAGTNDWQWNLDFFELTLGNVEPEPEPEPEPEPEPEPEPEPEPEPEP
EPEPEPEPQPEPDGDPVPVSGSFKLEAEHFQKVGGEVQIYSLSPGNAVNYFNSGDYLEFYVDLDAGGLYE
ASFRVGTGVASDVAVGLMVTDHKGDLTLKSVTPVTDQGGWDAFYNLTAQSQLNIYSGINTIRITGAGSAD
FQFNIDSITLTRVGPINPALDGDNDGVPDTSDNCPSSPANETANAEGCVPSQLDTDEDGINDKIDQCDAT
PAGDFVDALGCTSTGGDDDDFDGVLNGADQCGNTPYGMNVNAQGCSVFSGSDADNDGVANSEDTCANTPA
LEFANEQGCSSSQVANTHVVNVSVNANFKRSVNGVFDFGRRRHMTAHTAIHEPDWVGHTDKLNYLFNTLD
VYMGRDNGSATWKFNDTTEDPNKPNWPNMDYMVERGKGLREAHDQNPLFKRFSAEKQLLIAGTNPHALYP
TLSWFPNAFTWSGWQPKNIETSAAWVGQYMEHYFANASNGYVGEQLPEYWEVVNEPDMKMKTGQFMVTNQ
EAIWEYHNLVAQEIRDHLGAEAPPIGGMTWGQHDFYRRDGISRFADDSYDQWITNDDQVLQAEARAFYRN
AMATTVDDTRDQDWYQWDVMWKGFMDAAGDNMDFYSVHIYDWPGENVGDTTVVRRGGHTSAMLEMME
WYD
VKRNGFNNRKPIVLSEYGSVNGAWDNRAHEERYDIASIKAFNGMLMQFLERPDYVIKSLPFTPAKPLWGY
LPGGCGYDDAVACTTRYHYAMLIEDELNSGNWEWSSYIKFYELWADIDGTRVDSKSSDVDVQVDSYVKGN
ELFVILNNLEAADTTVNLDVSGIASVQNVELRNMHFDIQETHLDRHHMSAAPKTVTLAADATVVLRYTLA
SSVAVNNTVVEKKYFGESVSGGIEPHRISVAGGAKTLYINNVSVPSGYSEAILRLTVSLYPDEDDKVGGH
LSLDSITVNGTAIEAPIDWKGPKANRAERFFGVLDIPVPVELLQSTNTIAVDFRHNGELTVANLIVSEFT
SEPNR

>AEE22769.1
MKSITLLSCACIMAITSAQGADWSSFSIPAQAGAGKTWELQSVSDEFNYIAPTNNKPSAFTSRWNDSYIN
SWLGPGDTEFSSEHSYTTGGVLGLQATEKTGTNKVLSGIVSSKATFTYPLYLEAMVKPTNNTMANAVWML
SADSTREIDAMESYGSDRPGQEWFDQRMHVSHHVFIREPFQDYQPKDGGSWIYNNGETYRNKFRRYGVHW
KDAWNVDYYIDGVLVRSVSGPNIIDPKNYTNGKGLNSPMHIILDMEYQPWRDVKPSSAELADSSKSIFWI
DWIRVYKAQ

>AFN94094.1
MADIGSNDWDTIPIPANPGTGYVWELQEAYSDSFNYSGKTNEFTSKWNDSYFKSWTGPGLTHWDSSESWV
ADGNLIVSASRRQGTNKVNAGVITSKTKVKYPIFLEASIKVSNLELSSNFWLLSENDEREIDILEVYGGA
ADTWYAKNMSTNFHVFIRDAASNQIISDFNDQTHNEPSWGTYWRDGFHRFAAYWKSPTEVTFYIDGQQTP
EGSWAQVVMKDKDYTGAVLDKSVYNLNQEAFIIIDTEDHSWRSEMGIIASDTDLADNSKNKMYVDWVRVY

KPVTDDSSGGENGNVSVPSTYTNLQLVHSDLCLDVASGATWNGSTYQQWICNTGNSNQRFQFSALGNGQY
AISSEVSQLCLELDQASHADGATVHQWVCNHSDSKQTWTLFDKGSSTFEIRNKVSGKCLEIANASSNNGA
PLQQWSCDGGNNQRFKFL

>AAF03246.1
MNILKLLSCSTCAILCTATHAADWDAYSIPASAGSGKTWQLQTVSDQFNYQAGTSNKPAAFTNRWNASYI
NAWLGPGDTEFSSGHSYTTGGALGLQATEKAGTNKVLSGIVSSKATFTYPLYLEAMVKPSNNTMANAVWM
LSSDSTQEIDAMESYGSDRVGQEWFDQRMHVSHHVFIREPFQDYQPKDAGAWVYNSGETYRNKFRRYGVH
WKDAWNLDYYIDGVLVRSVSGPNIIDPEGYTGGTGLNKPMHIILDMEHQPWRDVKPNSTELADSNKSIFW
IDWVRVYKAN

>AAA91888.1
MKQLKLLIGSTLFMSIISVQAADWSSFSIPAQAGAGKSWQLQSVSDEFNYIAQPNNKPAAFNNRWNASYI
NAWLGPGDTEFSAGHSYTTGGALGLQATEKAGTNKVLSGIISSKATFTYPLYLEAMVKPTNNTMANAVWM
LSADSTQEIDAMESYGSDRIGQEWFDQRMHVSHHIFIRDPFQDYQPKDAGSWVYNNGETYRNKFRRYGVH
WKDAWNLDYYIDGVLVRSVSGPNIIDPENYTNGTGLNKPMHIILDMEHQPWRDVKPNASELADPNKSIFW
VDWIRVYKAQ

>ADA72015.1
MNILKLLSCSTCAILCTATHAADWDAYSIPASAGSGKTWQLQTVSDQFNYQAGTSNKPAAFTNRWNASYI
NAWLGPGDTEFSSGHSYTTGGALGLQATEKAGTNKVLAGIVSSKATFTYPLYLEAMVKPSNNTMANGVWM
LSSDSTQEIDAMEAYGSDRVGQEWFDQRMHVSHHVFIREPFQDYQPKDAGSWVYNNGETYRNKFRRYGVH
WKDAWNLDYYIDGVLVRSVSGPNIIDPEGYTGGTGLSKPMHILLDMEHQPWRDVKPNSAELADSNKSIFW
IDWIRVYKAN

>ACM50513.1
MLPRHYKNLLLLTQGLSLSFASLAANDWDTIPIPANPGTGYVWELQEAYSDSFNYSGKTNEFTSKWNDSY
FKSWTGPGLTHWDSSESWVADGNLIVSASRRQGTDKVNAGVITSKTKVKYPIFLEASIKVSNLELSSNFW
LLSENDEREIDILEVYGGAADTWYAKNMSTNFHVFIRDAASNQIISDFNDQTHNEPSWGTYWRDGFHRFA
AYWKSPTEVTFYIDGQQTPEGSWAQVVMKDKDYTGAVLDKSVYNLNQEAFIIIDTEDHSWRSEMGIIASD
TDLADNSKNKMYVDWVRVYKPVTDDSSGGENGNVSVPSTYTNLQLVHSDLCLDVASGATWNGSTYQQWIC
NTGNSDQRFQFSALGNGQYAISSEVSQLCLELDQASHADGATVHQWVCNHSDSKQTWTLFDKGSSTFEIR
NKVSGKCLEIANASSNNGAPLQQWSCDGGNNQRFKFL

>ABW77762.1
MKGFTKHSILMACSIGLAINATAADWDNIPIPAELDAGQSWELQQNYSDSFNYSGKNSTFTGKWKDSYFH
SWTGPGLTHWSSDESWVGDGNLIISASRRQGTNKVNAGVITSKTKVKYPIFLEASIKVSNLELSSNFWLL
SENDQREIDVLEVYGGARQDWYAKNMSTNFHVFFRNNDNSIKNDYNDQTHFTPTWGNYWRDGFHRFGVYW
KSPTDVTFYIDGQKTTKGAWSQVVMKDKDYTGAILDKSRYNMDQEAFIIIDTEDHSWRSEAGHIATDADL
ADSDKNKMYVDWIRVYKPTGGSTTPPTGDITPPSGYTNLQLAHSNRCVDVINGALWNGSTYQQYSCNTGN
NNQRFKFTKIANNQYSINAKVSQLCMELASGSSANGAKVQQWICNHANSNQTWSLEDKGSNTFEIRNKQS
GKCLEVANSSNANGGQIRQWACTGATNQRFKFL

>AAR87712.1
MKQLKLLIGSTLFMSITSVQAADWSPFSIPAQAGAGKSWQLQSVSDEFNYIAQPNNKPAAFNNRWNASYI
NAWLGPGDTEFSAGHSYTTGGALGLQATEKAGTNKVLSGIISSKATFTYPLYLEAMVKPTNNTMANAVWM
LSADSTPEIDAMESYGSDRIGQEWFDQRMHVSPHVFIRDPFQDYQPKDAGSWVYNNGETYRNKFRRYGVH
WKDAWNLDYYIDGVLVRSVSGPNIIDPENYTNGTGLNKPMHIILDMEHQPWRDVKPNASELADPNKSIFW
VDWLRVYKAQ

>ACF40223.1
MKNNLLLIGCVLTSTNLMANDWDAIPLPVAPDNGKVWQLQEAYSDSFNYTGKPAAFTSKWNDTYFNSWTG
PGLTYWQRDESWVSDGNLIISASRRAGTDQVNAGVITSKTKVTFPIFLEASIKVSNLELSSNFWLLSDND
EREIDVLEVYGGARDEWFARNMSTNFHVFIRDQQTNQIISDYNDQTHNTPSWGTYWREGFHRFGVYWKSP
TDVTFYIDGQQTPDGSWAQVVMKDKDYSGATLNKNTHNMDQSAYIIIDTEDHDWRSEAGNIATDADLADD
SKNKRYVDWVRVYKPVNAANTSSVTSGAQIKAKHSQKCIDITNGAMNNGSTYQQWNCNSNNENQAFELVE
LTNNEYAISSQLTGLCMQIANSSTSNGAGVEQWVCDHTKANQRFTLNNTGDGYFELRSSLSNKCIDIAGK

LQTNGASVVQWQCYNGDNQRFQLIE

>BAF34350.1
MKGFTKHPLLLACGLGLSLSTYAADWDNIPIPAELDPGQSWELQESYSDSFNYSGKPSSFTSKWKDAYFH
NWTGPGLTYWSSDESWVGDGNLIISASRRQGTTQVNAGGCHLKTKVKYPIFLEANIKVSNLELSSNFWLL
SENDQREIDVLEVYGGARQDWFAKNMSTNFHVFFRNNDNSIISDFNDQTHNTPTWGNYWREGFHRFGVYW
KSPTEVTFYINGQKTTKGAWSQVVMKDKDYTGAILDKSRYNMDQEMFIILDTEDHSWRSEAGHIATDADL
ADGDKNKMYVDWIRVYKPTGGSTTPPTGGINPPSGYTNLQVAHSNLCLDVKSGALWNGSTYQQWSCNTGN
NNQRFQFTALGNDEYSISAKVSQLCMELASGSSADGAKVQQWVCNHANTSQVWSLVDKGSNTFEIRNKQS
GKCLEIANDSGANGADLRQWSCDGGTNQRFKFQ

>WP_064385033.1
MNKTTLFIGCLLTTTNLFANDWDSIPLPVTPGDGKVWQLQETYSDSFNYTGKPAAFTSKWNDTYFNSWTG
PGLTYWQQDESWVSDGNLIISASRRAGTDKVNAGVITSKTKVSFPIFLEANIKVSNLELSSNFWLLSDND
EREIDVLEVYGGARDDWFAKNMSTNFHVFIRDQQSNQIISDYNDQTHNTPSWGTYWREGFHRFGVYWKSP
TEVTFYIDGQQTPDGSWAQVVMKDKDYTGATLNKNTHNMDQSAYIIIDTEDHDWRSEAGNIATDADLADG
SKNKMYVDWVRVYKPVNASNTNSVSNGAQIKAKHSQKCIDITAGAMSNGSYYQQWGCGSDNANQQFNLVE
LSNNEYAISSQLSGLCMQIENASTSNGAKLEQWVCDHAKASQRFTLNSTGDGYFELKSSLSNKCVDIAGK
LQTDGADIVQWQCYNGDNQRFQFIE

>ANB23850.1
MKYLSILLAGIITSTVAHCEDWDNIPLPTSPGGGKVWQLQTQYSDSFNYIGKPSDFTDKWNDSYFNNWTG
PGLTYWSSNESWVANGNLIISASRRQGTNQVNAGVITSKTKVKFPIYLEARIKVSNLELSSNFWLLSQND
EREIDILEVYGGAADTWFAKNMSTNFHVFLRDEQTNQIISDFNDQTHNIPSTGTYWRDQFHRFGAYWKSP
TEVTFYIDGQQTPDGSWAQVVMKDKDYTGATLDKSQYNMDQEAFIIIDTEDHDWRSNQGIVASDAELADG
SKNKMYVDWIRVYKPVDGVVTGNTNLQAKHSGRCIDVAQGAMINGSQYQQWTCDTTNTNQSFKFISAGNN
EYLIQSTQSNLCVELKDNNSANGANIHQWVCNSANDNQKWTLHDKGDQHFEIRSKVTGKCIDVAGKATTN
GANIVQWSCYNGQNQRFKFLQ

>WP_057791544.1
MKYLSILLAGIITSTVAHCEDWDNIPLPTSPGGGKVWQLQTQYSDSFNYIGKPSDFTDKWNDSYFNNWTG
PGLTYWSSNESWVANGNLIISASRRQGTNQVNAGVITSKTKVKFPIYLEARIKVSNLELSSNFWLLSQND
EREIDILEVYGGAADTWFAKNMSTNFHVFLRDEQTNQIISDFNDQTHNIPSTGTYWRDQFHRFGAYWKSP
TEVTFYIDGQQTPDGSWAQVVMKDKDYTGATLDKSQYNMDQEAFIIIDTEDHDWRSNQGIVASDAELADG
SKNKMYVDWIRVYKPVDGVVTGNTNLQAKHSGRCIDVAQGAMINGSQYQQWTCDTTNTNQSFKFISAGNN
EYLIQSTQSNLCVELKDNNSANGANIHQWVCNSANDNQKWTLHDKGDQHFEIRSKVTGKCIDVAGKATTN
GANIVQWSCYNGQNQRFKFLQ

>KYL34728.1
MNKTTLFIGCLLTTTNLFANDWDSIPLPVTPGDGKVWQLQETYSDSFNYTGKPAAFTSKWNDTYFNSWTG
PGLTYWQQDESWVSDGNLIISASRRAGTDKVNAGVITSKTKVSFPIFLEANIKVSNLELSSNFWLLSDND
EREIDVLEVYGGARDDWFAKNMSTNFHVFIRDQQSNQIISDYNDQTHNTPSWGTYWREGFHRFGVYWKSP
TEVTFYIDGQQTPDGSWAQVVMKDKDYTGATLNKNTHNMDQSAYIIIDTEDHDWRSEAGNIATDADLADG
SKNKMYVDWVRVYKPVNASNTNSVSNGAQIKAKHSQKCIDITAGAMSNGSYYQQWGCGSDNANQQFNLVE
LSNNEYAISSQLSGLCMQIENASTSNGAKLEQWVCDHAKASQRFTLNSTGDGYFELKSSLSNKCVDIAGK
LQTDGADIVQWQCYNGDNQRFQFIE

>KXI28844.1
MLKLAFLPLSLLASLSLFAADWDDIPVPATLPSGQTWQLLPLSDDFNYSAAAEGKSDEFKQRWHEGYINA
WTGPSWTEWHPSQSAVSDGVLKLTAKRKPGSWDIYMGSITSKQSVHYPLFLEIRAKLSNSVLASDFWLLS
EDSTQEIDVLEAYGGDRPGHEWFAERLHLSHHVFVREPFQDYQPKSDDTWYHTGTKWRDDYHRIGVYWRD
PWHLEYYVDGKLVKTSSGKEVIDPLDYTKGTGLSKPMRVIINMEDQQWRTEQGLTPTDEELAEREKVTYS
IDWVRFYKPVDSK

>AMJ94778.1
MKYLSILLAGIITSTVAHCEDWDNIPLPTSPGGGKVWQLQTQYSDSFNYIGKPSDFTDKWNDSYFNNWTG
PGLTYWSSNESWVANGNLIISASRRQGTNQVNAGVITSKTKVKFPIYLEARIKVSNLELSSNFWLLSQND

EREIDILEVYGGAADTWFAKNMSTNFHVFLRDEQTNQIISDFNDQTHNIPSTGTYWRDQFHRFGAYWKSP
TEVTFYIDGQQTPDGSWAQVVMKDKDYTGATLDKSQYNMDQEAFIIIDTEDHDWRSNQGIVASDAELADG
SKNKMYVDWIRVYKPVDGVVTGNTNLQAKHSGRCIDVAQGAMINGSQYQQWTCDTTNTNQSFKFISAGNN
EYLIQSTQSNLCVELKDNNSANGANIHQWVCNSANDNQKWTLHDKGDQHFEIRSKVTGKCIDVAGKATTN
GANIVQWSCYNGQNQRFKFLQ

>AMJ90896.1
MKYLSILLAGIITSTVAHCEDWDNIPLPTSPGGGKVWQLQTQYSDSFNYIGKPSDFTDKWNDSYFNNWTG
PGLTYWSSNESWVANGNLIISASRRQGTNQVNAGVITSKTKVKFPIYLEARIKVSNLELSSNFWLLSQND
EREIDILEVYGGAADTWFAKNMSTNFHVFLRDEQTNQIISDFNDQTHNIPSTGTYWRDQFHRFGAYWKSP
TEVTFYIDGQQTPDGSWAQVVMKDKDYTGATLDKSQYNMDQEAFIIIDTEDHDWRSNQGIVASDAELADG
SKNKMYVDWIRVYKPVDGVVTGNTNLQAKHSGRCIDVAQGAMINGSQYQQWTCDTTNTNQSFKFISAGNN
EYLIQSTQSNLCVELKDNNSANGANIHQWVCNSANDNQKWTLHDKGDQHFEIRSKVTGKCIDVAGKATTN
GANIVQWSCYNGQNQRFKFLQ

>AMJ87035.1
MKYLSILLAGIITSTVAHCEDWDNIPLPTSPGGGKVWQLQTQYSDSFNYIGKPSDFTDKWNDSYFNNWTG
PGLTYWSSNESWVANGNLIISASRRQGTNQVNAGVITSKTKVKFPIYLEARIKVSNLELSSNFWLLSQND
EREIDILEVYGGAADTWFAKNMSTNFHVFLRDEQTNQIISDFNDQTHNIPSTGTYWRDQFHRFGAYWKSP
TEVTFYIDGQQTPDGSWAQVVMKDKDYTGATLDKSQYNMDQEAFIIIDTEDHDWRSNQGIVASDAELADG
SKNKMYVDWIRVYKPVDGVVTGNTNLQAKHSGRCIDVAQGAMINGSQYQQWTCDTTNTNQSFKFISAGNN
EYLIQSTQSNLCVELKDNNSANGANIHQWVCNSANDNQKWTLHDKGDQHFEIRSKVTGKCIDVAGKATTN
GANIVQWSCYNGQNQRFKFLQ

>AMJ74593.1
MKYLSILLAGIITSTVAHCEDWDNIPLPTSPGGGKVWQLQTQYSDSFNYIGKPSDFTDKWNDSYFNNWTG
PGLTYWSSNESWVANGNLIISASRRQGTNQVNAGVITSKTKVKFPIYLEARIKVSNLELSSNFWLLSQND
EREIDILEVYGGAADTWFAKNMSTNFHVFLRDEQTNQIISDFNDQTHNIPSTGTYWRDQFHRFGAYWKSP
TEVTFYIDGQQTPDGSWAQVVMKDKDYTGATLDKSQYNMDQEAFIIIDTEDHDWRSNQGIVASDAELADG
SKNKMYVDWIRVYKPVDGVVTGNTNLQAKHSGRCIDVAQGAMINGSQYQQWTCDTTNTNQSFKFISAGNN
EYLIQSTQSNLCVELKDNNSANGANIHQWVCNSANDNQKWTLHDKGDQHFEIRSKVTGKCIDVAGKATTN
GANIVQWSCYNGQNQRFKFLQ

>ALO78721.1
MRALLTAVLGLSCTHALAADWDNTPVPANAGNGKVWELQAVSDDFNYSSSLNNYHSEFTSRWHEGFINPW
TGPGLTEWTDGHAYVTGGNLGIAATRKLGTDKVRAGSITSHDTFTYPLYVETKAKISKLVLASDVWLLSA
DSTQEIDVLEAYGSDRAGQEWFAERIHLSHHVFIRDPFQDYQPTDAGSWYTDGQGTVWSDDFHRIGVHWK
DPWNLDYYIDGQLVRSVSGDNIIDPNGFTNGTGLSKPMHLIINTEDQDWRSDNGISPTDAELANTNKSIY
WVDWIRVYKPVDGGDNGENTDVPASATSIKGRQSGKCIDLASGSSANGANIQQWACGTNNANQEFTFVPV
DSGWYELRTKHNKCVGVGGNSSANGAVVIQWDCFNGQNLHVKPVDLGNGYVELRARHSNKCLDVADASTA
NGADIRQWQCNGNTNQQFSFN

>GAC10035.1
MAITSAQGADWSSFSIPAQAGAGKTWELQSVSDEFNYIAPTNNKPSAFTSRWNDSYINSWLGPGDTEFSS
EHSYTTGGALGLQATEKTGTNKVLSGIVSSKATFTYPLYLEAMVKPTNNTMANAVWMLSADSTREIDAME
SYGSDRPGQEWFDQRMHVSHHVFIREPFQDYQPKDGGSWIYNNGETYRNKFRRYGVHWKDAWNVDYYIDG
VLVRSVSGPNIIDLKNYTNGKGLNSPMHIILDMEYQPWRDVKPSSAELADSSKSIFWIDWIRVYKAQ

>GAC03896.1
MAITSAQGADWSSFSIPAQAGAGKTWELQSVSDEFNYIAPTNNKPSAFTSRWNDSYINSWLGPGDTEFSS
EHSYTTGGALGLQATEKAGTNKVLSGIVSSKATFTYPLYLEAMVKPTNNTMANAVWMLSADSTREIDAME
SYGSDRPGQEWFDQRMHVSHHVFIREPFQDYQPKDEGSWIYNNGETYRNKFRRYGVHWKDAWNVDYYIDG
VLVRSVSGPNIIDPKNYTNGKGLNSPMHIILDMEYQPWRDVKPSSAELADSSKSIFWIDWIRVYKAQ

>ALQ08013.1
MNILKLLSCSTCAILCTATHAADWDAYSIPASAGSGKTWQLQTVSDQFNYQAGTSNKPAAFTNRWNASYI
NAWLGPGDTEFSSGHSYTTGGALGLQATEKAGTNKVLAGIVSSKATFTYPLYLEAMVKPSNNTMANAVWM

LSSDSTQEIDAMESYGSDRVGQEWFDQRMHVSHHVFIREPFQDYQPKDAGAWVYNNGETYRNKFRRYGVH
WKDAWNLDYYIDGVLVRSVSGPNIIDPEGYTGGTGLNKPMHIILDMEHQPWRDVKPNSTELADSNKSIFW
IDWVRVYKAN

>WP_055733246.1
MLPRHYKNLLLLTQGLSLSFASLAANDWDTIPIPANPGTGYVWELQEAYSDSFNYSGKTNEFTSKWNDSY
FKSWTGPGLTHWDSSESWVADGNLIVSASRRQGTDKVNAGVITSKTKVKYPIFLEASIKVSNLELSSNFW
LLSENDEREIDILEVYGGAADTWYAKNMSTNFHVFIRDAASNQIISDFNDQTHNEPSWGTYWRDGFHRFA
AYWKSPTEVTFYIDGQQTPEGSWAQVVMKDKDYTGAVLDKSVYNLNQEAFIIIDTEDHSWRSEMGIIASD
TDLADNSKNKMYVDWVRVYKPVTDDSSGGENGNVSVPSTYTNLQLVHSDLCLDVASGATWNGSTYQQWIC
NTGNSNQRFQFSALGNGQYAISSEVSQLCLELDQASHADGATVHQWVCNHSDSKQTWTLFDKGSSTFEIR
NKVSGKCLEIANASSNNGAPLQQWSCDGGNNQRFKFL

>WP_055024616.1
MNVIYATTLMATSALTTVCIAADWDNIPLPADPGNGLVWQLNEQYSDSFNYQGKNATFNSKWNDTYFNNW
EGPGLTRWSQNESWVSDGNLIISASRKAPNLVNAGVITSKTKVKYPIFLEANIKVSNLELSSNFWLLSEN
DEREIDILEVYGGSRETWFTQNMSTNFHVFLRNSDNSIRSDFNDQTHNTPTWGNYWREGFHRFGAYWKSP
TDVTFYIDGQETPQGSWAEVVMKDKDYTGAIMDKYQYTMNEEMFIILDTEDHSWRSEAGNVATDADLADQ
SKNKMYVDWIRVYQPVTNGGSGGDNGGNDGSIEPNTQYNFVAKHSNKCIDVINSQTYNGSQYQQASCTNT
NAQKFTLNQLSNGFYTIRSNVSNLCLDLSAGSTSNGAKIQQWVCNSSNQNQMWNLADKGDGYYEIISKVS
NKCLDIAGKSTNDGASLTQWSCYNGTNQQFKVE

>WP_055014357.1
MKNNLLLIGCVLTSTNLMANDWDAIPLPVAPDNGKVWQLQEAYSDSFNYTGKPAAFTSKWNDTYFNSWTG
PGLTYWQRDESWVSDGNLIISASRRAGTDQVNAGVITSKTKVTFPIFLEASIKVSNLELSSNFWLLSDND
EREIDVLEVYGGARDKWFARNMSTNFHVFIRDQQTNQIISDYNDQTHNTPSWGTYWREGFHRFGVYWKSP
TDVTFYIDGQQTPDGSWAQVVMKDKDYSGATLNKNTHNMDQSAYIIIDTEDHDWRSEAGNIATDADLADD
SKNKMYVDWVRVYKPVNAANTSSVTSGAQIKAKHSQKCIDIKNGAMNNGSTYQQWNCNSNNENQAFELVE
LTNNEYAISSQLTGLCMQIANSSTSNGAGVEQWVCDHTKANQRFTLNSTGDGYFELRASLSNKCIDIAGK
LQTNGASVVQWQCYNGDNQRFQLIE

>GAA77741.1
MNILKLLSCSTCAILCTATHAADWEAYSIPASAGSGKTWQLQTVSDQFNYQAGTSNKPAAFTNRWNASYI
NAWLGPGDTEFSSGHSYTTGGALGLQATEKAGTNKVLAGIVSSKATFTYPLYLEAMVKPSNNTMANGVWM
LSSDSTQEIDAMEAYGSDRVGQEWFDQRMHVSHHVFIREPFQDYQPKDAGSWVYNNGETYRNKFRRYGVH
WKDAWNLDYYIDGVLVRSVSGPNIIDPEGYTGGTGLSKPMHILLDMEHQPWRDVKPNSAELADSNKSIFW
IDWIRVYKAN

>WP_045984435.1
MNILKLLSCSTCAILCTATHAADWDAYSIPASAGSGKTWQLQTVSDQFNYQAGTSNKPAAFTNRWNASYI
NAWLGPGDTEFSSGHSYTTGGALGLQATEKAGTNKVLAGIVSSKATFTYPLYLEAMVKPSNNTMANAVWM
LSSDSTQEIDAMESYGSDRVGQEWFDQRMHVSHHVFIREPFQDYQPKDAGAWVYNSGETYRNKFRRYGVH
WKDAWNLDYYIDGVLVRSVSGPNIIDPEGYTGGTGLNKPMHIILDMEHQPWRDVKPNSTELADSNKSIFW
IDWVRVYKAN

>WP_050482451.1
MNILKLLSCSTCAILCTATHAADWDAYSIPASAGSGKTWQLQTVSDQFNYQAGTSNKPAAFTNRWNASYI
NAWLGPGDTEFSSGHSYTTGGALGLQATEKAGTNKVLSGIVSSKATFTYPLYLEAMVKPSNNTMANAVWM
LSSDSTQEIDAMESYGSDRVGQEWFDQRMHVSHHVFIREPFQDYQPKDAGAWVYNSGETYRNKFRRYGVH
WKDAWNLDYYIDGVLVRSVSGPNIIDPEGYTGGTGLNKPMHIILDMEHQPWRDVKPNSTELADSNKSIFW
IDWVRVYKAN

>KDC50284.1
MNILKLLSCSTCAILCTATHAADWDAYSIPASAGSGKTWQLQTVSDQFNYQAGTSNKPAAFTNRWNASYI
NAWLGPGDTEFSSGHSYTTGGALGLQATEKAGTNKVLAGIVSSKATFTYPLYLEAMVKPSNNTMANAVWM
LSSDSTQEIDAMESYGSDRVGQEWFDQRMHVSHHVFIREPFQDYQPKDAGAWVYNSGETYRNKFRRYGVH
WKDAWNLDYYIDGVLVRSVSGPNIIDPEGYTGGTGLNKPMHIILDMEHQPWRDVKPNSTELADSNKSIFW

IDWVRVYKAN

>KDC53878.1
MNILKLLSCSTCAILCTATHAADWDAYSIPASAGSGKTWQLQTVSDQFNYQAGTSNKPAAFTNRWNASYI
NAWLGPGDTEFSSGHSYTTGGALGLQATEKAGTNKVLSGIVSSKATFTYPLYLEAMVKPSNNTMANAVWM
LSSDSTQEIDAMESYGSDRVGQEWFDQRMHVSHHVFIREPFQDYQPKDAGAWVYNSGETYRNKFRRYGVH
WKDAWNLDYYIDGVLVRSVSGPNIIDPEGYTGGTGLNKPMHIILDMEHQPWRDVKPNSTELADSNKSIFW
IDWVRVYKAN

>WP_026971821.1
MFNVQRVLISAVSLSVCSAVQANDWDDIPIPADPGSGYVWELQEAYSDSFNYTGKPSAFSSKWNDTYFHG
WTGPGLTYWSSDESWVSDGNLIISASRRAGTNQVNAGVVTSKTKVKYPIFLEASIKVSNLELSSNFWLLS
ENDQREIDILEVYGGAEDEWFAKNMSTNFHVFFRDGGNNIISDFNDQTHNTPSWGTYWRDGFHRFAAYWK
SPTDVTFYINGQPTPEGSWAQVVMKDKDYTGAILDKSRYNMDEEMFIILDTEDHSWRSEQGIVASDADLA
NPNKNKMYVDWIRVYKPVAEDGGGSGGDGSVDVPSGNTNLQLVHSNLCLDVANGATWNGSTYHQWVCNTG
NNNQRFTLSSLGNGEYALQSKVSQLCMELKDGSSSNGATVQQWVCNHSDSNQRWSLVDKGSNTFEIRNKA
SGKCLDIAGASMSNGGKLQQWACTGANNQRFRFVQ

>EWH10233.1
MLQKITLCASLALSSTTMYAADWDGVPIPAPAGQNKTWQILSISDDFNYAASPNNKPSAFTSRWNDSYIN
AWKGPGDTEFSSGHSYTNSGKLALQAAEKPGTDKVYAGIISSKQTFTYPLYIEARAKSTNNTMANAVWML
SADSTQELDAMEAYGSDRPGQEWFDRRMHVSHHVFIREPFQDYQPKDAGSWIYNNEEPWRVAYHNYGMHW
KDPWNVDYYIDGVLVRSVSGQQMIDPHNYTNGTGVNKPLHIIIDMEHQDWRDVKPTPAELADPARSIFYV
DWIRVYKPVDSSASAPTPPTSATSLKARHSNKCIDLAAGNSANGTNMQQWNCSATNTNQDITFVAKGGGY
YEMKTKHNKCIDVAGKATANGANLVQWNCYNGTNQQFKLLDKGNGWFQLQAKHSGKCLEIANSATTNGAN
LQQWGCGNGNNQQWKFQ

>AML39432.1
MPEGVLYVKRKQKLRWDGVQKLKSYLKIKRILAVLLCAVLLIVSCLAGPTTISYAVEASDELPENIRVDK
ADFDTIPDVAEHYKQLFGDNAVSYFKSINGESRIQRINFLQYPEGEYLEVNGQVTIDIPAMDARVWRLFG
DVRFEGSVAQTMELYVIDPNGVETQWTVFKNGGWKDGVTGLYTNYTKMGFQESNPTEGITHPWLIKDENG
DVIYDGYKMKIVGNGTLRSVYHWEEEGVPLEFDTSAWTVLGGDKEILDVSVNVDALTNLSMNGVNKLPEE
VFKRYHVNSGPIGVEQADGEITVLDEAYHKTTNDYGFIPGRGAFHYTLMTSWAGLKEDSERPGYADFTTT
KEIYAKSQPAIDKFESLYPSIGKDYVLTLDGWPKWMWENPNSGQSEHFGTPSYSNFDAAADAAAQLIKSI
DTRFDGLGPKYVEVKNESTIPQEWWFFQSDPQHAWSYLSEFHNKVAEAVKAENPDVLVGGPSSAFMYLEK
NDFDEARAQLKFMDETKDSLDWYSHHFYENSNLFIHDRENNSDGFLSGRMEAVLDLLNAHMVNTDNVKPI
YITEEGTYNTAGSDADYFQKLVAHNGYMLRFMNYSDTIGMLVPYLYPIINWRPNSNDTFYKYNETMNGLM
EEMTPLEAYLDMWKDYRGAFLPSEADQERVYTNAVRYNDKVYVAVHNLNTQRVNLDLNVFTGDANIASVT
RKHFFLEKGKLTYEEENVADLDNVYMRVQEMSVFEITLDSNPSFTKTWERQFAYAPEELVPTSPNAPAVF
KIESLPTTLAKATLRIGFGKTGSGFTTDMSVVVNPDNPANTQNFSKDLEYTNKPGDLLTFAEFELDASKL
LADNTIHIMIPEDGGYITSVQMIQYDEQEAPTGVATDALTASIADAKSKLSSTVVSSTGNEVEQGNKWVE
KHIHDTLNIEVMKAEVVAQDALATDSEINTALEDLTKAMGIFDQYTKTKETPTGNRGAKISFEDGEAVDY
TYNVDTVTTTTDSQGATDGSKALHTEFTSFTSYAWDTTGTYSGSLDFTAPEEGWSLGEDPFTFDVTNLRN
YKIQLRVEISDTSDIKGTYYYALGANASRSISIEDFGVAGGTWLADGNFPRNTAIDTENIKSIRFYVFSP
TEEPITNAALAFDRIIIGNVPNEHTEEQLAAEAADALSADSLIFAEGDTAQAVTNHISLPSVGLHNASIT
WISSHPSIVTNDGTVSRPEYGSGNQTVTLTATITIGAASTTKDIEVTVLQKAATPTSPGGNNSPINPMPS
IPQTTNVDVLVDGKKHDNIAKSVTTKEGDRTVTTITVDQQLLKNSLGNMDNDAIIAIVVANSSDVVYGKL
NGQSVKDLEQKNAVIELRTDTFSYKLPVQQINMNELVQELGSDVKLEDISIDMKISSADADAKKALESSA
VKGGFTVVSPPVDFHITYTYEEKTIEVRTFDTFVERSIVIPEDVNQTQITTGVVVEEDGTVKPIPTRFVK
QDGSYYAIMNSLTNGTYAVIWNPVNFQDMSGHWAENTVNDLGSRLIISGVDGTRFEPDRSITRAEFTAIV
IRAFGLNAEEGESSYSDIARDSWYEAYLNTATTYGLINGYANGQFGPNELITREQVMSIMERAMEITGYE
AVADMSEVDEILTKFKDRTEAAAYAKSGIAASIKAGLVMGRSADVLAPKAAITRAEVAEIVRRLLVNSDL
I

>WP_071352467.1
MDLYGGNEKRKFEGTGYFRLEKESDRWWLVDPEGSAFVTIGVNHADETNLKYDHNFEIWKEKYGSRENWI
KGLVKDLKDWGFNTIGWTGDYISGDWGQALDWFGDPINLGHSSSWAAADYKIADMPYCLQIRVAEIEDWN

GQPAYPDVYSREFDMYCEYLARSICANHAESKNLIGYFLVDIPGWLPHASGKDFKGLEGLDKEERKSKLF
DVASKYYETITKHIRKYDPNHLILGDRYNGNKGIPTEVLEAAKPYVDVLSVQYFPSNNEEGYQQMKEDLA
KWQALIDKPVINADIGNWCQTKMNPNRVSDLQTQADRARDYIDSISSLINEPWFIGWHWCAHVENKARGW
GIKDPYDQPYNDFINPVKGFNKEIYEKI

>WP_069937766.1
MSSFSSYGGDEKQKFHSTGYFRIEQTNERWWLVDPEGNAFVTIGVNHADETNLKYDHNFDIWKKKYGSRE
NWIKGLSKDLKDWGFNTIGWTGDYISGDWGVALDWFGDPINLGHSASWSAADYKHADMPYCLQIRVDAFE
DWNGQPTYPDVFSHEFDMYCEYLARSICADHSESKNLIGYFLVDIPAWIRHASGRDFKVLQDLNEEERAS
KLFEVANKYYETITKHIRKYDPNHLILGDRYNGNKGIPEEVLTAMKPYVDVLSVQYFTSPNQEGYQQMKE
DLGKWQKITGKPVILADIGNWCQTCMNPNRVSEIKDQSGRAEDYINSLNAVLNEPWFLGWHWCAHLENKA
RGWGIKDPYDEPYKDFIDPVEVYNKNVYKNLYQKA

>OES45769.1
MSSFSSYGGDEKQKFHSTGYFRIEQTNERWWLVDPEGNAFVTIGVNHADETNLKYDHNFDIWKKKYGSRE
NWIKGLSKDLKDWGFNTIGWTGDYISGDWGVALDWFGDPINLGHSASWSAADYKHADMPYCLQIRVDAFE
DWNGQPTYPDVFSHEFDMYCEYLARSICADHSESKNLIGYFLVDIPAWIRHASGRDFKVLQDLNEEERAS
KLFEVANKYYETITKHIRKYDPNHLILGDRYNGNKGIPEEVLTAMKPYVDVLSVQYFTSPNQEGYQQMKE
DLGKWQKITGKPVILADIGNWCQTCMNPNRVSEIKDQSGRAEDYINSLNAVLNEPWFLGWHWCAHLENKA
RGWGIKDPYDEPYKDFIDPVEVYNKNVYKNLYQKA

>GAV11364.1
MRYRRRYLISWTCNGNNGRDHWVMERNGKLSAADIKRLEKDLSNKWGLSRPVIITNYIQIN

>KSU86249.1
MSKFSSYGEDEKRKFQATGYFRLEQDHEKWCLVDPEGNAFVTIGVNHADESNLKYDHNFEIWKKKYGSRE
NWIKGLSKDLKDWGFNTIGWTGDYISGDWGIALDWFGDPINLGHSTSWSAADYKYADMPYCLQIRVAEFE
DWNGQPSYPDVFSHEFDMYCEYLARSICADHTESKNLIGYFLVDIPAWLPHASGRDFEVLKDLNEKERSS
KLFEVASKYYETITKYIRKYDPNHLILGDRYNGNKGIPEEVLSAMKPYVDVLSVQYFTSPNEEGYQKMKK
ELSSWQKFTGKPVILADIGNWCQTCMNPNRVSEIKDQSGRAADYIDSLSVILDEPWFLGWHWCAHLENKA
RGWGIKDPYDEPYHDFINPVKEFNKNVYKNIYQKV

>KSU82185.1
MNRTFEATGYFRLERENERWWLVDPKGNQFVTIGVNHADETNLKYDHNFDIWKTKYGSRENWIKGLSEDL
KSWGFNTIGWTGDYISGDWGVALDWFGDPINLGHSQSWSAADYKHADMPYCLQIRVSEFEDWNGHPVYPD
VFSHEFEMYCDYLARSICADHANSKNLIGYFLVDIPAWIRHASGSDFKQLEGLNEQERSSKLYEVAAKYY
ETITASIRKYDTNHLILGDRFNGNKGIPEEVLKAMKPYVDVLSVQYFTSPDEAGYQQMKEDLARWQAIAD
KPVVIADIGNWCKTHMNPNRVSDIHDQAGRADDYIASLNTVLHEPWFLGWHWCAHLENKARGWGIKDPYD
EPYADFVDPVKDFNKGVYQKIEDRVKF

>WP_041619012.1
MLCLWSLMFSTFTGWAPQGGVQAAEPAASVLNDEFQSWSLTHEHSPTLTLESAGTGGFDPTRVKRSAAYK
EFITYKTAEPLQSFAVYGYYHPQGKPYDHPLFYVSPNGTDYTKVTPGLIELGGYFPLIIYELEGLPAGSV
YLKIEYTGGLIVRSPLIGRVVLNGPSVVVPGMPPGEVSAGSKLTLSSTAGASIFYTTDGSDPRTSPAKKL
YTDGIGLSGISLLKAYSQFGTNTASASAVNTFRYIVKGTQDRVVPVTEDVYVDGTVPDTNRGTEPLLFMK
ALKNREAYLKFDLSKVDAGTQKVYLNLYGQTYDSSQAPARLKLYGVDSSWSEGTMTYNTKPLPTPAPAGA
EIGEAVWPYEPSGWMSIDITGYIKGQKALGKTTASVGIVNTTDGAAYVNAKEAGGNAPYLLLVQGQPGTG
PGEEPVNLPGLVDPLDTFEHVMARTNMRTEGADAQYLGGDLKRATRSTTALGSLTYKTAYDIQSFAIHSY
FFMGVAVDPLKVYASPNGTSFTEVVLQPYKSGAPVSNWQMFVYEAKGLPAGTRYLKIEVKGDVKAWTPQI
GKVILNKNVMSVLAVPDHGTVVGQPISVTLSTPTSGAQIFYKLNAETEARPYTGAISVQDSTTLHAFAIK
NGLEASAPRSYVYRSKADWVVDKYGQMNSADFPEKVKTDDELRADLAADKAYYDGLQAPNWDAYGGLVGS
KETYGLQAKGYYNVQTAADGRKILVTPLGTAFFSVGINGISPNDTYTMVTGREEIFEWLPDYANTEYKSA
FMGTKDYFSYYLANYIKKTGTPYTSEAFYQTSWARMKKWGFNSAGGWSPTPLSEQFKVPYFPFLPLGSLE
WAKIEGVKIFDIFADGAEAKLEAALEPVLTPNKNNPLIVGYFLGNENHYQKIAPEIPKLKGSVVAAKRRL
VQMLQEKYGTIEAFNTAWNGTYGSFAELNDAVLYVDSQQAQYDVDDFVKLYLDTYYGTIARVFRKYDPNH
LLLGDRWLTIPMNNAKLRGFMAEAAGRHMDVISMNHYAKNLNVTMLGEVHAKSGGKPLILTENGFGTMEQ
GLGSPLLVADQNERQLRYRTYVEGAASLGYVVGAHWFTYLDQAATGRWSEADDGEHYNFGLVNVADRPYK

TFLQGVMATNHDIYSVMLGRKEPFRYDFGDAPRQPGNNTMAIPYTPVPVPIDGEVNGFPAGAPKFVLDEA
QRVVGTDGAGMSGEYTLAWDNTNLYVTASITDPTPMRNQYTDMNVWRGDGVELFFGPGDLTTPGDPLFND
RQLIMSAQPTAEGQPFWYWFNTSRQKAVNMAVKARSGGYVIEAAIPWETVNMQASEGLQFLFDLGFDDSE
DGENRKRQWMWNGTNTNSTERSNWGKATLVKP

>WP_041618141.1
MNVRRLLSGGCAGVLLFSGIGVSNAAGTAGGSGGVSAPQLKTVTYSGAGAAGEAAVNGTAQGGAPASGEA
VPRLAADAGGSAGTAETETQALATAVQVDRFGQWVSATFPEKVTSESQLLEDVAADAAYYGALQAPDWDP
YGGLKGSREQYGLNATGFYSIQEAAGRKVMVTPEGNLFFSLGVNGIAPTDTYTVVTGREGIYEWIPPYES
EYKSAFLNTKDNFSYYLANRIRKTGQPYTSRSLYTEGIQRIRKWGFNTAAGWTPVNLSREFSVPHVPFLP
LSDMAWAKVNGLKFFDIFVDGAEAKLDAALGPLLTEHKNNPYIIGYFFGNESYYHKLAPDLPKMKGSEVA
AKRRLVQMLQEKYGTIEAFNAAWNGTYASFTALIDAPLYIDSQQAQYDVDDFVKLYLDTFFGTVSRVFRK
YDPNHLLIGDRWLTLPVNNVKVRGFLAEAAGRHLDVISINHYSKNLDTVMLNHIHQASGGKPIMLTEIGY
GSSEQGLGAPTQMLVADQNERMLRYRNYVEGAASLGYIVGVHWFAYLDQAATGRWAEGTTGERYNFGLLN
VADRPYKPFLEGVMASNKDIYPVLLGQRVPFKHEFGDVPRQPGTNKMEIPYTPAPIPIDGEVNGFPGDAA
AVTLGPGQLVNGTGGEGMQGEYTFAWDENRLYVTAVIKDPTPMKNNNPNINVWRGDGVELFFGPQDLTTP
GDPLFLDRQLIASAGLNNGQPFWYWYFTNRQKPVEMAVKLLPDGSGYVLEAALTWDSINLQGQADTRFLF
DFGFDDSEDGNTRKRQWVWNGTSANSTNRGHWGQATLVKRAAPAIRITGVEEGGDYTDEVLPVVEVEDAT
GVRSRTVTLDGAPWTDGTPVTAPGSHELTVEAVNTVGITAKKTVTFAVYGSTALELSPAAGQYSDEVQLE
AKLSGSSGGPVPGAELSFEVNGSAAGTAVTDAQGRAVLPYRISTGPESGQLSVKAAYTPAAGLYLRPAAS
EGALTVRPEDAALQYTGETYVKERRPAVLSAQVVQADDGSAGALSGLPVRFTVSEVRPDGTLRELAGVTD
SVYATGADGRAEAAAQLPPGLYEVRAELLAGPLYKAPAPVRSTLAVAAPVAGHSVTVNGTVPGGTFLGKP
AGTLVLNSMVSYDLAGGLTGALRVSGEPGGAELYVKSFDWLVIAGDRAYVQGTAVSGRQTYTVRLLLKEL
TTVSLYVWKGRDTSAEPVHKQLNAQLSGAVLTLP

>AFH59380.2
MKTVTYSGAGAAGEAAVNGTAQGGAPASGEAVPRLAADAGGSAGTAETETQALATAVQVDRFGQWVSATF
PEKVTSESQLLEDVAADAAYYGALQAPDWDPYGGLKGSREQYGLNATGFYSIQEAAGRKVMVTPEGNLFF
SLGVNGIAPTDTYTVVTGREGIYEWIPPYESEYKSAFLNTKDNFSYYLANRIRKTGQPYTSRSLYTEGIQ
RIRKWGFNTAAGWTPVNLSREFSVPHVPFLPLSDMAWAKVNGLKFFDIFVDGAEAKLDAALGPLLTEHKN
NPYIIGYFFGNESYYHKLAPDLPKMKGSEVAAKRRLVQMLQEKYGTIEAFNAAWNGTYASFTALIDAPLY
IDSQQAQYDVDDFVKLYLDTFFGTVSRVFRKYDPNHLLIGDRWLTLPVNNVKVRGFLAEAAGRHLDVISI
NHYSKNLDTVMLNHIHQASGGKPIMLTEIGYGSSEQGLGAPTQMLVADQNERMLRYRNYVEGAASLGYIV
GVHWFAYLDQAATGRWAEGTTGERYNFGLLNVADRPYKPFLEGVMASNKDIYPVLLGQRVPFKHEFGDVP
RQPGTNKMEIPYTPAPIPIDGEVNGFPGDAAAVTLGPGQLVNGTGGEGMQGEYTFAWDENRLYVTAVIKD
PTPMKNNNPNINVWRGDGVELFFGPQDLTTPGDPLFLDRQLIASAGLNNGQPFWYWYFTNRQKPVEMAVK
LLPDGSGYVLEAALTWDSINLQGQADTRFLFDFGFDDSEDGNTRKRQWVWNGTSANSTNRGHWGQATLVK
RAAPAIRITGVEEGGDYTDEVLPVVEVEDATGVRSRTVTLDGAPWTDGTPVTAPGSHELTVEAVNTVGIT
AKKTVTFAVYGSTALELSPAAGQYSDEVQLEAKLSGSSGGPVPGAELSFEVNGSAAGTAVTDAQGRAVLP
YRISTGPESGQLSVKAAYTPAAGLYLRPAASEGALTVRPEDAALQYTGETYVKERRPAVLSAQVVQADDG
SAGALSGLPVRFTVSEVRPDGTLRELAGVTDSVYATGADGRAEAAAQLPPGLYEVRAELLAGPLYKAPAP
VRSTLAVAAPVAGHSVTVNGTVPGGTFLGKPAGTLVLNSMVSYDLAGGLTGALRVSGEPGGAELYVKSFD
WLVIAGDRAYVQGTAVSGRQTYTVRLLLKELTTVSLYVWKGRDTSAEPVHKQLNAQLSGAVLTLP

>AFH61769.1
MILPFKFPGARFSTGIVVLAAITAAAAMLTSPPGFPGGLPGAGGPAGVPADEKQQPAKSESPAAGPLRVD
RFGQVASAKFQEKVAGEEALKKDREADEAYYGSLQAPDWDAYGGLKGSGETYGLKATGYYHVETLQGRKV
LVTPLGTLYFSLGVNGITVNDTYTQLKGQEDRFEWLPPYEGEYRSAFLNTKDTFSFYVANRIRKTGQTYT
SREFYQESISRIRKWGFNSAGGWSPANYATEFKVPHFPFLPLSEITEAKVDGLKIFDIFAEGAEARIDAL
FAKTLPQNKNNPYIVGYFLGNESDYHRIINDVPKLKASQAPSKRKLVEVLIAKYGTIDKFNTAWKAGYSS
FDRLYEEPLALSSSQAISDMDAFLELYLDTYYGTVERLFRKYDPNHLLLGDRWLTTPSNHPKVRGILAKT
AGKHMDVISINHYSPQLDRTMLQDVYDKSGGRPVLLTEFAFGTREQGLSAPLLASSEAERARLYRAYVEE
AASLGFVVGTHWFSYLDQPATGRWFEGLQGESYNFGLLNVADRPYKTFLSGVTETHKSIYDMVLGKKAPF
RTTEAAVRLK

>AFH59384.1
MLCLWSLIFSTFTGWAPQGGVQAAEPAASVLNDEFQSWSLTHEHSPALTLESAGTGGFDPTRVKRSAAYK

EFITYKTAEPLQSFAVYGYYHPQGKPYDHPLFYVSPNGTDYTKVTPGLIELGGYFPLIIYELEGLPAGSV
YLKIEYTGGLIVRSPLIGRVVLNGPSVVVPGMPPGEVPTGSKLTLSSTAGASIFYTTDGSDPRTSPAKKL
YTDGIGLSGISLLKAYSQFGTNTASASAVNTFRYIVKGTQDRVVPVTEDVYVDGTVPDTNRGTEPLLFMK
ALKNREAYLKFDLSKVDAGTQKVYLNLYGHTYDSSQAPARLKLYGVDSSWSEGTMTYNTKPLPTPAPAGA
EIGEAVWPYEPSGWMSIDITGYIKGQKALGKTTASVGIVNTTDGAAYVNAKEAGGNAPYLLLVQGQPGTG
PGEEPVNLPGLVDPLDTFEHVMARTNMRTEGADAQYLGGDLKRATRSTTALGSLTYKTAYDIQSFAIHSY
FFMGVAVDPLKVYASPNGTSFTEVVLQPYKSGASVSNWQMFVYEAKGLPAGTRYLKIEVKGDVKAWTPQI
GKVILNKNVMSVLAVPDHGTVVGQPISVTLSTPTSGAQIFYKLNAETEARPYTGAISVQDSTTLHTFAIK
NGLEASAPRSYVYRSKADWVVDKYGQMNSADFPEKVKTDDELRADLAADKAYYDGLQAPNWDAYGGLVGS
KETYGLQAKGYYNVQTAADGRKILVTPLGTAFFSVGINGISPNDTYTMVTGREEIFEWLPDYANTEYKSA
FMGTKDYFSYYLANYIKKTGTPYTSEAFYQTSWARMKKWGFNSAGGWSPTPLSEQFKVPYFPFLPLGSLE
WAKIEGVKIFDIFADGAEAKLEAALEPVLMPNKNNPLIVGYFLGNENHYQKIAPEIPKLKGSVVAAKRRL
VQMLQEKYGTIEAFNTAWNGTYGSFAELNDAVLYVDSQQAQYDVDDFVKLYLDTYYGTIARVFRKYDPNH
LLLGDRWLTIPMNNAKLRGFMAEAAGRHMDVISMNHYAKNLNVTMLGEVHAKSGGKPLILTENGFGTMEQ
GLGSPLLVADQNERQLRYRTYVEGAASLGYVVGAHWFTYLDQAATGRWSEADDGEHYNFGLVNVADRPYK
TFLQGVMATNHDIYSVMLGRKEPFRYDFGDAPRQPGNNTMAIPYTPVPVPIDGEVNGFPAGAPKFVLDEA
QRVVGTDGAGMSGEYTLAWDNTNLYVTASITDPTPMRNQYTDMNVWRGDGVELFFGPGDLTTPGDPLFND
RQLIMSAQPTAEGQPFWYWFNTSRQKAVNMAVKARSGGYVIEAAIPWETVNMQAAEGLQFLFDLGFDDSE
DGENRKRQWMWNGTNTNSTERSNWGKATLVKP

>AFC29596.1
MILPFKFPGARFSTGIVVLAAITAAAAMLTSPPGFPGGLPGAGGPAGVPADEKQQPAKSESPAAGPLRVD
RFGQVASAKFQEKVAGEEALKKDREADEAYYGSLQAPDWDAYGGLKGSGETYGLKATGYYHVETLQGRKV
LVTPLGTLYFSLGVNGITVNDTYTQLKGQEDRFEWLPPYEGEYRSAFLNTKDTFSFYVANRIRKTGQTYT
SREFYQESISRIRKWGFNSAGGWSPANYATEFKVPHFPFLPLSEITEAKVDGLKIFDIFAEGAEARIDAL
FAKTLPQNKNNPYIVGYFLGNESDYHRIINDVPKLKASQAPSKRKLVEVLIAKYGTIDKFNTAWKAGYSS
FDRLYEEPLALSSSQAISDMDAFLELYLDTYYGTVERLFRKYDPNHLLLGDRWLTTPSNHPKVRGILAKT
AGKHMDVISINHYSPQLDRTMLQDVYDKSGGRPVLLTEFAFGTREQGLSAPLLASSEAERARLYRAYVEE
AASLGFVVGTHWFSYLDQPATGRWFEGLQGESYNFGLLNVADRPYKTFLSGVTETHKSIYDMVLGKKAPF
RTTEAAVRLK

>AFC27244.1
MNLRRGLLMLCLWSLMFSTFTGWAPQGGVQAAEPAASVLNDEFQSWSLTHEHSPTLTLESAGTGGFDPTR
VKRSAAYKEFITYKTAEPLQSFAVYGYYHPQGKPYDHPLFYVSPNGTDYTKVTPGLIELGGYFPLIIYEL
EGLPAGSVYLKIEYTGGLIVRSPLIGRVVLNGPSVVVPGMPPGEVSAGSKLTLSSTAGASIFYTTDGSDP
RTSPAKKLYTDGIGLSGISLLKAYSQFGTNTASASAVNTFRYIVKGTQDRVVPVTEDVYVDGTVPDTNRG
TEPLLFMKALKNREAYLKFDLSKVDAGTQKVYLNLYGQTYDSSQAPARLKLYGVDSSWSEGTMTYNTKPL
PTPAPAGAEIGEAVWPYEPSGWMSIDITGYIKGQKALGKTTASVGIVNTTDGAAYVNAKEAGGNAPYLLL
VQGQPGTGPGEEPVNLPGLVDPLDTFEHVMARTNMRTEGADAQYLGGDLKRATRSTTALGSLTYKTAYDI
QSFAIHSYFFMGVAVDPLKVYASPNGTSFTEVVLQPYKSGAPVSNWQMFVYEAKGLPAGTRYLKIEVKGD
VKAWTPQIGKVILNKNVMSVLAVPDHGTVVGQPISVTLSTPTSGAQIFYKLNAETEARPYTGAISVQDST
TLHAFAIKNGLEASAPRSYVYRSKADWVVDKYGQMNSADFPEKVKTDDELRADLAADKAYYDGLQAPNWD
AYGGLVGSKETYGLQAKGYYNVQTAADGRKILVTPLGTAFFSVGINGISPNDTYTMVTGREEIFEWLPDY
ANTEYKSAFMGTKDYFSYYLANYIKKTGTPYTSEAFYQTSWARMKKWGFNSAGGWSPTPLSEQFKVPYFP
FLPLGSLEWAKIEGVKIFDIFADGAEAKLEAALEPVLTPNKNNPLIVGYFLGNENHYQKIAPEIPKLKGS
VVAAKRRLVQMLQEKYGTIEAFNTAWNGTYGSFAELNDAVLYVDSQQAQYDVDDFVKLYLDTYYGTIARV
FRKYDPNHLLLGDRWLTIPMNNAKLRGFMAEAAGRHMDVISMNHYAKNLNVTMLGEVHAKSGGKPLILTE
NGFGTMEQGLGSPLLVADQNERQLRYRTYVEGAASLGYVVGAHWFTYLDQAATGRWSEADDGEHYNFGLV
NVADRPYKTFLQGVMATNHDIYSVMLGRKEPFRYDFGDAPRQPGNNTMAIPYTPVPVPIDGEVNGFPAGA
PKFVLDEAQRVVGTDGAGMSGEYTLAWDNTNLYVTASITDPTPMRNQYTDMNVWRGDGVELFFGPGDLTT
PGDPLFNDRQLIMSAQPTAEGQPFWYWFNTSRQKAVNMAVKARSGGYVIEAAIPWETVNMQASEGLQFLF
DLGFDDSEDGENRKRQWMWNGTNTNSTERSNWGKATLVKP

>AFC27240.1
MNVRRLLSGGCAGVLLFSGIGVSNAAGTAGGSGGVSAPQLKTVTYSGAGAAGEAAVNGTAQGGAPASGEA
VPRLAADAGGSAGTAETETQALATAVQVDRFGQWVSATFPEKVTSESQLLEDVAADAAYYGALQAPDWDP
YGGLKGSREQYGLNATGFYSIQEAAGRKVMVTPEGNLFFSLGVNGIAPTDTYTVVTGREGIYEWIPPYES

EYKSAFLNTKDNFSYYLANRIRKTGQPYTSRSLYTEGIQRIRKWGFNTAAGWTPVNLSREFSVPHVPFLP
LSDMAWAKVNGLKFFDIFVDGAEAKLDAALGPLLTEHKNNPYIIGYFFGNESYYHKLAPDLPKMKGSEVA
AKRRLVQMLQEKYGTIEAFNAAWNGTYASFTALIDAPLYIDSQQAQYDVDDFVKLYLDTFFGTVSRVFRK
YDPNHLLIGDRWLTLPVNNVKVRGFLAEAAGRHLDVISINHYSKNLDTVMLNHIHQASGGKPIMLTEIGY
GSSEQGLGAPTQMLVADQNERMLRYRNYVEGAASLGYIVGVHWFAYLDQAATGRWAEGTTGERYNFGLLN
VADRPYKPFLEGVMASNKDIYPVLLGQRVPFKHEFGDVPRQPGTNKMEIPYTPAPIPIDGEVNGFPGDAA
AVTLGPGQLVNGTGGEGMQGEYTFAWDENRLYVTAVIKDPTPMKNNNPNINVWRGDGVELFFGPQDLTTP
GDPLFLDRQLIASAGLNNGQPFWYWYFTNRQKPVEMAVKLLPDGSGYVLEAALTWDSINLQGQADTRFLF
DFGFDDSEDGNTRKRQWVWNGTSANSTNRGHWGQATLVKRAAPAIRITGVEEGGDYTDEVLPVVEVEDAT
GVRSRTVTLDGAPWTDGTPVTAPGSHELTVEAVNTVGITAKKTVTFAVYGSTALELSPAAGQYSDEVQLE
AKLSGSSGGPVPGAELSFEVNGSAAGTAVTDAQGRAVLPYRISTGPESGQLSVKAAYTPAAGLYLRPAAS
EGALTVRPEDAALQYTGETYVKERRPAVLSAQVVQADDGSAGALSGLPVRFTVSEVRPDGTLRELAGVTD
SVYATGADGRAEAAAQLPPGLYEVRAELLAGPLYKAPAPVRSTLAVAAPVAGHSVTVNGTVPGGTFLGKP
AGTLVLNSMVSYDLAGGLTGALRVSGEPGGAELYVKSFDWLVIAGDRAYVQGTAVSGRQTYTVRLLLKEL
TTVSLYVWKGRDTSAEPVHKQLNAQLTGAVLTLP

>AEI41018.1
MILPFKFPGARFSTGIVVLAAITAAAAMLTSPPGFPGGLPGAGGPAGVPADEKQQPAKSESPAAGPLRVD
RFGQVASAKFQEKVAGEEALKKDREADEAYYGSLQAPDWDAYGGLKGSGETYGLKATGYYHVETLQGRKV
LVTPLGTLYFSLGVNGITVNDTYTQLKGQEDRFEWLPPYEGEYRSAFLNTKDTFSFYVANRIRKTGQTYT
SREFYQESISRIRKWGFNSAGGWSPANYSTEFKVPHFPFLPLSEITEAKVDGLKIFDIFAEGAEARINAL
FAKTLPQNKNNPYIVGYFLGNESDYHRIINDVPKLKASQAPSKRKLVEVLIAKYGTIDKFNTAWKAGYSS
FDRLYEEPLALSSSQAISDMDAFLELYLDTYYGTVERLFRKYDPNHLLLGDRWLTTPSNHPKVRGILAKT
AGKHMDVISINHYSPQLDRTMLQDVYEKSGGRPVLLTEFAFGTREQGLSAPLLASSEAERARLYRAYVEE
AASLGFVVGTHWFSYLDQPATGRWFEGLQGESYNFGLLNVADRPYKTFLSGVTETHKSIYDMVLGKKAPF
RTTEAAVRLK

>AEI38935.1
MLCLWSLMFSTFTGWAPQGGVQAAEPAASVLNDEFQSWSLTHEHSPTLTLESAGTGGFDPTRVKRSAAYK
EFITYKTAEPLQSFAVYGYYHPQGKPYDHPLFYVSPNGTDYTKVTPGLIELGGYFPLIIYELEGLPAGSV
YLKIEYTGGLIVRSPLIGRVVLNGPSVVVPGMPPGEVSAGSKLTLSSTAGASIFYTTDGSDPRTSPAKKL
YTDGIGLSGISLLKAYSQFGTNTASASAVNTFRYIVKGTQDRVVPVTEDVYVDGTVPDTNRGTEPLLFMK
ALKNREAYLKFDLSKVDAGTQKVYLNLYGQTYDSSQAPARLKLYGVDSSWSEGTMTYNTKPLPTPAPAGA
EIGEAVWPYEPSGWMSIDITGYIKGQKALGKTTASVGIVNTTDGAAYVNAKEAGGNAPYLLLVQGQPGTG
PGEEPVNLPGLVDPLDTFEHVMARTNMRTEGADAQYLGGDLKRATRSTTALGSLTYKTAYDIQSFAIHSY
FFMGVAVDPLKVYASPNGTSFTEVVLQPYKSGAPVSNWQMFVYEAKGLPAGTRYLKIEVKGDVKAWTPQI
GKVILNKNVMSVLAVPDHGTVVGQPISVTLSTPTSGAQIFYKLNAETEARPYTGAISVQDSTTLHAFAIK
NGLEASAPRSYVYRSKADWVVDKYGQMNSADFPEKVKTDDELRADLAADKAYYDGLQAPNWDAYGGLVGS
KETYGLQAKGYYNVQTAADGRKILVTPLGTAFFSVGINGISPNDTYTMVTGREEIFEWLPDYANTEYKSA
FMGTKDYFSYYLANYIKKTGTPYTSEAFYQTSWARMKKWGFNSAGGWSPTPLSEQFKVPYFPFLPLGSLE
WAKIEGVKIFDIFADGAEAKLEAALEPVLTPNKNNPLIVGYFLGNENHYQKIAPEIPKLKGSVVAAKRRL
VQMLQEKYGTIEAFNTAWNGTYGSFAELNDAVLYVDSQQAQYDVDDFVKLYLDTYYGTIARVFRKYDPNH
LLLGDRWLTIPMNNAKLRGFMAEAAGRHMDVISMNHYAKNLNVTMLGEVHAKSGGKPLILTENGFGTMEQ
GLGSPLLVADQNERQLRYRTYVEGAASLGYVVGAHWFTYLDQAATGRWSEADDGEHYNFGLVNVADRPYK
TFLQGVMATNHDIYSVMLGRKEPFRYDFGDAPRQPGNNTMAIPYTPVPVPIDGEVNGFPAGAPKFVLDEA
QRVVGTDGAGMSGEYTLAWDNANLYVTASITDPTPMRNQYTDMNVWRGDGVELFFGPGDLTTPGDPLFND
RQLIMSAQPTAEGQPFWYWFNTSRQKAVNMAVKARSGGYVIEAAIPWETVNMQAAEGLQFLFDLGFDDSE
DGENRKRQWMWNGTNTNSTERSNWGKATLVKP

>AEI38930.1
MNVRRLLSGGCAGVLLFSGIGVSNAAGTAGGSGGVSAPQLKTVTYSGAGAAGEAAVNGTAQGGAPASGEA
VPRLAADAGGSAGTAETQALATAVQVDRFGQWVSATFPEKVTSESQLLEDVAADAAYYGALQAPDWDPYG
GLKGSREQYGLNATGFYSIQEAAGRKVMVTPEGNLFFSLGVNGIAPTDTYTVVTGREGIYEWIPPYESEY
KSAFLNTKDNFSYYLANRIRKTGQPYTSRSLYTEGIQRIRKWGFNTAAGWTPVNLSREFSVPHVPFLPLS
DMAWAKVNGLKFFDIFVDGAEAKLDAALGPLLTEHKNNPYIIGYFFGNESYYHKLAPDLPKMKGSEVAAK
RRLVQMLQEKYGTIEAFNAAWNGTYASFTALIDAPLYIDSQQAQYDVDDFVKLYLDTFFGTVSRVFRKYD
PNHLLIGDRWLTLPVNNVKVRGFLAEAAGRHLDVISINHYSKNLDTVMLNHIHQASGGKPIMLTEIGYGS

SEQGLGAPTQMLVADQNERMLRYRNYVEGAASLGYIVGVHWFAYLDQAATGRWAEGTTGERYNFGLLNVA
DRPYKPFLEGVMASNKDIYPVLLGQRVPFKHEFGDVPRQPGTNKMEIPYTPAPIPIDGEVNGFPGDAAAV
TLGPGQLVNGTGGEGMQGEYTFAWDENRLYVTAVIKDPTPMKNNNPNINVWRGDGVELFFGPQDLTTPGD
PLFLDRQLIASAGLNNGQPFWYWYFTNRQKPVEMAVKLLPDGSGYVLEAALTWDSINLQGQADTRFLFDF
GFDDSEDGNTRKRQWVWNGTSANSTNRGHWGQATLVKRAAPAIRITGVEEGGDYTDEVLPVVEVEDATGV
RSRTVTLDGAPWTDGTPVTAPGSHELTVEAVNTVGITAKKTVTFAVYGSTALELSPAAGQYSDEVQLEAK
LSGSSGGPVPGAELSFEVNGSAAGTAVTDAQGRAVLPYRISTGPESGQLSVKAAYTPAAGLYLRPAASEG
ALTVRPEDAALQYTGETYVKERRPAVLSAQVVQADDGSAGALSGLPVRFTVSEVRPDGTLRELAGVTDSV
YATGADGRAEAAAQLPPGLYEVRAELLAGPLYTAPAPVRSTLAVAAPVAGHSVSVNGTVPGGTFLGKPAG
TLVLNSMVSYDLAGSLTGALRVSGEPSGAELYVKSFDWLVIAGDRAYVQGTAVSGRQTYTVRLLLKELTT
VSLYVWKGRDTSAEPVHKQLNAQLSGAVLTLP

>AFK65387.1
MILPFKFPGARFSTGIVVLAAITAAAAMLTSPPGFPGGLPGAGGPAGVPADEKQQPAKSESPAAGPLRVD
RFGQVASAKFQEKVAGEEALKKDREADEAYYGSLQAPDWDAYGGLKGSGETYGLKATGYYHVETLQGRKV
LVTPLGTLYFSLGVNGITVNDTYTQLKGQEDRFEWLPPYEGEYRSAFLNTKDTFSFYVANRIRKTGQTYT
SREFYQESISRIRKWGFNSAGGWSPANYATEFKVPHFPFLPLSEITEAKVDGLKIFDIFAEGAEARIDAL
FAKTLPQNKNNPYIVGYFLGNESDYHRIINDVPKLKASQAPSKRKLVEVLIAKYGTIDKFNTAWKAGYSS
FDRLYEEPLALSSSQAISDMDAFLELYLDTYYGTVERLFRKYDPNHLLLGDRWLTTPSNHPKVRGILAKT
AGKHMDVISINHYSPQLDRTMLQDVYDKSGGRPVLLTEFAFGTREQGLSAPLLASSEAERARLYRAYVEE
AASLGFVVGTHWFSYLDQPATGRWFEGLQGESYNFGLLNVADRPYKTFLSGVTETHKSIYDMVLGKKAPF
RTTEAAVRLK

>WP_010168305.1
MNFKKYFKVFGVMAMTVMTASTNAIELKPEATDAEVAAHSFELISFEAGEPELKITTSKGAAGNVSYEFS
DEYVTEGDVSLKVRYEEGSSANVAIARADGTTWDFSDDRMILAYDVTNPSDVSGRLVTTFKYNGGSISYQ
NYVPANATRTVYCVLNEDQYNIGADTLPSPVGDDGIVIAKGWGGSNFDPSSISSISLRYSLDDVGYYIFD
NFRVVKNPLLNPSITYANIVDEFGQYTRAEWDNKIHSEEELLAAAEAERIQNDIWIAESLARTDRSQYGG
YKNEDLRQEATGHFYTTKIDDKWTLIDPDGYPFFSTGFGIVRKNGMDTWVSGREYMYDLPEKTSKLGDHY
SRLNNTIQPPAGFKSGEGYNHYGANLERKFGDDWLKEWANDAVRRFEAWGITSIGAWAEPSLFFGKGSEH
KTPYTAFTWTTNSANGTHVRLYDTVPDAFDPEFAKSARKSIIDQAVKYGIDEDPYCFGLYVDNEYKWGNN
MSNNPLVNAIFDDDVANAKSYAKRHFVEVLEEKYGTISALNVAWGSKLASFEELGKPYKGKIAAEDAGMI
VGLLADKYYSVIDEILNELLPGTMYLGSRNTEFGTPIEVVKAATKYVDILSFNNYNPDVIRENFKTEEYD
MPMMIGEFNFSSQDAGVFGMNGTTVQTQEERAKSYIKYVESALTSGDFVGVHWFQYYDKPILGRSWDGEN
TSTGFVNGTDQPYEKLVEASRYLYDTMYETMFNHVPMTTIDILNDKINMSVGTTAQLDVKTSPANLQDDI
NYYTSNAYVAKVDDNGVVTAVTEGEATITARSASDLFVITSANITVGDDRNQRNIEFADVAKESTVAVGN
TINLAEYAEFTNVTAGEVEWKSSKKAIATVSSNGVVTAHQPGRVNIVIKDKNGYATDSINLVVK

>WP_010166088.1
MKLAMQLAILAVTAFTAIPTLAVSQRDIDNHSFGAVSFENDDKQTFKFSTSSPPWGFSEYSIEEFMPTDG
DNALKVRYEAGSSSLIRIAPSNGGTWDFSDNRMVLAFDVTNTNEYTQRLNTVFRYQGGTTAYVNVIPPNT
TKTMYCVLDQDKIQLGADALPSPIAEDGIIPGPGWGNVNFDKSKITGIDLSWSVGGDVGYYTFDNFRIVE
NPLLDVSTAYAGIVDKYGQYALKTWPNKIFSDAQLKEAQAAEEAQVAIWKAEQEARTDRTIYGGYKNEAL
RQEATGHFYTTKINDIWTLIDPEGYPFFSTGFGIVRKNGMDTWIDGRNYMFQELPAKNGEFADHYGKASG
MQAPFGQKYGTSFSFYSMNLEKKYGSDWLQDWGDMAIDRFEAWGLTSLGAWAEPDLFFGKGDTHKTPYAA
FTWTTVSSGKHVRLHDTIPDAFDPEFEKSTVAAIKDQAVRYGIHEDPYCFGVYVDNEYPWGTNMTNNNLV
KAIFDDDVASEKSYAKRHFVAVLEDKYGSISALNSAWGSNLSSFAELGKPYTGKIAAEDASMVVSQLSDQ
YYKVVDSVVTELLPGTMYLGSRNTEWGTPIEVIHSAIKYVDILSFNNYNPDVQRESFKFEEYDMPMIIGE
FCFSATDAGHFSPTTMAVSSQQDRADAYINYVESALKSGKFVGVHWFQYYDEPILGRSWDGENFNLGFVD
VTDQPYMELVDASRYVYDTMYDTMFTQIPMTNIQNEQTSIDLRVGQTATIETATTPANLNEDVSYYSTNL
YVATVDENGVVTAVGDGDATIVTKNANDLFVVTSTNVVVGDGTEPASIRFADNAKELTLAAGSTLDLKNQ
LKLDGVNAANITWKTSKKAIATIENGVVTAHAPGRINIVAEEKNGFATDSISIVVQ

>WP_014649103.1
MLCLWSLIFSTFTGWAPQGGVQAAEPAASVLNDEFQSWSLTHEHSPALTLESAGTGGFDPTRVKRSAAYK
EFITYKTAEPLQSFAVYGYYHPQGKPYDHPLFYVSPNGTDYTKVTPGLIELGGYFPLIIYELEGLPAGSV
YLKIEYTGGLIVRSPLIGRVVLNGPSVVVPGMPPGEVPTGSKLTLSSTAGASIFYTTDGSDPRTSPAKKL

YTDGIGLSGISLLKAYSQFGTNTASASAVNTFRYIVKGTQDRVVPVTEDVYVDGTVPDTNRGTEPLLFMK
ALKNREAYLKFDLSKVDAGTQKVYLNLYGHTYDSSQAPARLKLYGVDSSWSEGTMTYNTKPLPTPAPAGA
EIGEAVWPYEPSGWMSIDITGYIKGQKALGKTTASVGIVNTTDGAAYVNAKEAGGNAPYLLLVQGQPGTG
PGEEPVNLPGLVDPLDTFEHVMARTNMRTEGADAQYLGGDLKRATRSTTALGSLTYKTAYDIQSFAIHSY
FFMGVAVDPLKVYASPNGTSFTEVVLQPYKSGASVSNWQMFVYEAKGLPAGTRYLKIEVKGDVKAWTPQI
GKVILNKNVMSVLAVPDHGTVVGQPISVTLSTPTSGAQIFYKLNAETEARPYTGAISVQDSTTLHTFAIK
NGLEASAPRSYVYRSKADWVVDKYGQMNSADFPEKVKTDDELRADLAADKAYYDGLQAPNWDAYGGLVGS
KETYGLQAKGYYNVQTAADGRKILVTPLGTAFFSVGINGISPNDTYTMVTGREEIFEWLPDYANTEYKSA
FMGTKDYFSYYLANYIKKTGTPYTSEAFYQTSWARMKKWGFNSAGGWSPTPLSEQFKVPYFPFLPLGSLE
WAKIEGVKIFDIFADGAEAKLEAALEPVLMPNKNNPLIVGYFLGNENHYQKIAPEIPKLKGSVVAAKRRL
VQMLQEKYGTIEAFNTAWNGTYGSFAELNDAVLYVDSQQAQYDVDDFVKLYLDTYYGTIARVFRKYDPNH
LLLGDRWLTIPMNNAKLRGFMAEAAGRHMDVISMNHYAKNLNVTMLGEVHAKSGGKPLILTENGFGTMEQ
GLGSPLLVADQNERQLRYRTYVEGAASLGYVVGAHWFTYLDQAATGRWSEADDGEHYNFGLVNVADRPYK
TFLQGVMATNHDIYSVMLGRKEPFRYDFGDAPRQPGNNTMAIPYTPVPVPIDGEVNGFPAGAPKFVLDEA
QRVVGTDGAGMSGEYTLAWDNTNLYVTASITDPTPMRNQYTDMNVWRGDGVELFFGPGDLTTPGDPLFND
RQLIMSAQPTAEGQPFWYWFNTSRQKAVNMAVKARSGGYVIEAAIPWETVNMQAAEGLQFLFDLGFDDSE
DGENRKRQWMWNGTNTNSTERSNWGKATLVKP

>WP_014369879.1
MILPFKFPGARFSTGIVVLAAITAAAAMLTSPPGFPGGLPGAGGPAGVPADEKQQPAKSESPAAGPLRVD
RFGQVASAKFQEKVAGEEALKKDREADEAYYGSLQAPDWDAYGGLKGSGETYGLKATGYYHVETLQGRKV
LVTPLGTLYFSLGVNGITVNDTYTQLKGQEDRFEWLPPYEGEYRSAFLNTKDTFSFYVANRIRKTGQTYT
SREFYQESISRIRKWGFNSAGGWSPANYATEFKVPHFPFLPLSEITEAKVDGLKIFDIFAEGAEARIDAL
FAKTLPQNKNNPYIVGYFLGNESDYHRIINDVPKLKASQAPSKRKLVEVLIAKYGTIDKFNTAWKAGYSS
FDRLYEEPLALSSSQAISDMDAFLELYLDTYYGTVERLFRKYDPNHLLLGDRWLTTPSNHPKVRGILAKT
AGKHMDVISINHYSPQLDRTMLQDVYDKSGGRPVLLTEFAFGTREQGLSAPLLASSEAERARLYRAYVEE
AASLGFVVGTHWFSYLDQPATGRWFEGLQGESYNFGLLNVADRPYKTFLSGVTETHKSIYDMVLGKKAPF
RTTEAAVRLK

>WP_014368188.1
MNVRRLLSGGCAGVLLFSGIGVSNAAGTAGGSGGVSAPQLKTVTYSGAGAAGEAAVNGTAQGGAPASGEA
VPRLAADAGGSAGTAETETQALATAVQVDRFGQWVSATFPEKVTSESQLLEDVAADAAYYGALQAPDWDP
YGGLKGSREQYGLNATGFYSIQEAAGRKVMVTPEGNLFFSLGVNGIAPTDTYTVVTGREGIYEWIPPYES
EYKSAFLNTKDNFSYYLANRIRKTGQPYTSRSLYTEGIQRIRKWGFNTAAGWTPVNLSREFSVPHVPFLP
LSDMAWAKVNGLKFFDIFVDGAEAKLDAALGPLLTEHKNNPYIIGYFFGNESYYHKLAPDLPKMKGSEVA
AKRRLVQMLQEKYGTIEAFNAAWNGTYASFTALIDAPLYIDSQQAQYDVDDFVKLYLDTFFGTVSRVFRK
YDPNHLLIGDRWLTLPVNNVKVRGFLAEAAGRHLDVISINHYSKNLDTVMLNHIHQASGGKPIMLTEIGY
GSSEQGLGAPTQMLVADQNERMLRYRNYVEGAASLGYIVGVHWFAYLDQAATGRWAEGTTGERYNFGLLN
VADRPYKPFLEGVMASNKDIYPVLLGQRVPFKHEFGDVPRQPGTNKMEIPYTPAPIPIDGEVNGFPGDAA
AVTLGPGQLVNGTGGEGMQGEYTFAWDENRLYVTAVIKDPTPMKNNNPNINVWRGDGVELFFGPQDLTTP
GDPLFLDRQLIASAGLNNGQPFWYWYFTNRQKPVEMAVKLLPDGSGYVLEAALTWDSINLQGQADTRFLF
DFGFDDSEDGNTRKRQWVWNGTSANSTNRGHWGQATLVKRAAPAIRITGVEEGGDYTDEVLPVVEVEDAT
GVRSRTVTLDGAPWTDGTPVTAPGSHELTVEAVNTVGITAKKTVTFAVYGSTALELSPAAGQYSDEVQLE
AKLSGSSGGPVPGAELSFEVNGSAAGTAVTDAQGRAVLPYRISTGPESGQLSVKAAYTPAAGLYLRPAAS
EGALTVRPEDAALQYTGETYVKERRPAVLSAQVVQADDGSAGALSGLPVRFTVSEVRPDGTLRELAGVTD
SVYATGADGRAEAAAQLPPGLYEVRAELLAGPLYKAPAPVRSTLAVAAPVAGHSVTVNGTVPGGTFLGKP
AGTLVLNSMVSYDLAGGLTGALRVSGEPGGAELYVKSFDWLVIAGDRAYVQGTAVSGRQTYTVRLLLKEL
TTVSLYVWKGRDTSAEPVHKQLNAQLTGAVLTLP

>WP_013916179.1
MILPFKFPGARFSTGIVVLAAITAAAAMLTSPPGFPGGLPGAGGPAGVPADEKQQPAKSESPAAGPLRVD
RFGQVASAKFQEKVAGEEALKKDREADEAYYGSLQAPDWDAYGGLKGSGETYGLKATGYYHVETLQGRKV
LVTPLGTLYFSLGVNGITVNDTYTQLKGQEDRFEWLPPYEGEYRSAFLNTKDTFSFYVANRIRKTGQTYT
SREFYQESISRIRKWGFNSAGGWSPANYSTEFKVPHFPFLPLSEITEAKVDGLKIFDIFAEGAEARINAL
FAKTLPQNKNNPYIVGYFLGNESDYHRIINDVPKLKASQAPSKRKLVEVLIAKYGTIDKFNTAWKAGYSS
FDRLYEEPLALSSSQAISDMDAFLELYLDTYYGTVERLFRKYDPNHLLLGDRWLTTPSNHPKVRGILAKT
AGKHMDVISINHYSPQLDRTMLQDVYEKSGGRPVLLTEFAFGTREQGLSAPLLASSEAERARLYRAYVEE

AASLGFVVGTHWFSYLDQPATGRWFEGLQGESYNFGLLNVADRPYKTFLSGVTETHKSIYDMVLGKKAPF
RTTEAAVRLK

>WP_013914101.1
MLCLWSLMFSTFTGWAPQGGVQAAEPAASVLNDEFQSWSLTHEHSPTLTLESAGTGGFDPTRVKRSAAYK
EFITYKTAEPLQSFAVYGYYHPQGKPYDHPLFYVSPNGTDYTKVTPGLIELGGYFPLIIYELEGLPAGSV
YLKIEYTGGLIVRSPLIGRVVLNGPSVVVPGMPPGEVSAGSKLTLSSTAGASIFYTTDGSDPRTSPAKKL
YTDGIGLSGISLLKAYSQFGTNTASASAVNTFRYIVKGTQDRVVPVTEDVYVDGTVPDTNRGTEPLLFMK
ALKNREAYLKFDLSKVDAGTQKVYLNLYGQTYDSSQAPARLKLYGVDSSWSEGTMTYNTKPLPTPAPAGA
EIGEAVWPYEPSGWMSIDITGYIKGQKALGKTTASVGIVNTTDGAAYVNAKEAGGNAPYLLLVQGQPGTG
PGEEPVNLPGLVDPLDTFEHVMARTNMRTEGADAQYLGGDLKRATRSTTALGSLTYKTAYDIQSFAIHSY
FFMGVAVDPLKVYASPNGTSFTEVVLQPYKSGAPVSNWQMFVYEAKGLPAGTRYLKIEVKGDVKAWTPQI
GKVILNKNVMSVLAVPDHGTVVGQPISVTLSTPTSGAQIFYKLNAETEARPYTGAISVQDSTTLHAFAIK
NGLEASAPRSYVYRSKADWVVDKYGQMNSADFPEKVKTDDELRADLAADKAYYDGLQAPNWDAYGGLVGS
KETYGLQAKGYYNVQTAADGRKILVTPLGTAFFSVGINGISPNDTYTMVTGREEIFEWLPDYANTEYKSA
FMGTKDYFSYYLANYIKKTGTPYTSEAFYQTSWARMKKWGFNSAGGWSPTPLSEQFKVPYFPFLPLGSLE
WAKIEGVKIFDIFADGAEAKLEAALEPVLTPNKNNPLIVGYFLGNENHYQKIAPEIPKLKGSVVAAKRRL
VQMLQEKYGTIEAFNTAWNGTYGSFAELNDAVLYVDSQQAQYDVDDFVKLYLDTYYGTIARVFRKYDPNH
LLLGDRWLTIPMNNAKLRGFMAEAAGRHMDVISMNHYAKNLNVTMLGEVHAKSGGKPLILTENGFGTMEQ
GLGSPLLVADQNERQLRYRTYVEGAASLGYVVGAHWFTYLDQAATGRWSEADDGEHYNFGLVNVADRPYK
TFLQGVMATNHDIYSVMLGRKEPFRYDFGDAPRQPGNNTMAIPYTPVPVPIDGEVNGFPAGAPKFVLDEA
QRVVGTDGAGMSGEYTLAWDNANLYVTASITDPTPMRNQYTDMNVWRGDGVELFFGPGDLTTPGDPLFND
RQLIMSAQPTAEGQPFWYWFNTSRQKAVNMAVKARSGGYVIEAAIPWETVNMQAAEGLQFLFDLGFDDSE
DGENRKRQWMWNGTNTNSTERSNWGKATLVKP

>WP_013914096.1
MNVRRLLSGGCAGVLLFSGIGVSNAAGTAGGSGGVSAPQLKTVTYSGAGAAGEAAVNGTAQGGAPASGEA
VPRLAADAGGSAGTAETQALATAVQVDRFGQWVSATFPEKVTSESQLLEDVAADAAYYGALQAPDWDPYG
GLKGSREQYGLNATGFYSIQEAAGRKVMVTPEGNLFFSLGVNGIAPTDTYTVVTGREGIYEWIPPYESEY
KSAFLNTKDNFSYYLANRIRKTGQPYTSRSLYTEGIQRIRKWGFNTAAGWTPVNLSREFSVPHVPFLPLS
DMAWAKVNGLKFFDIFVDGAEAKLDAALGPLLTEHKNNPYIIGYFFGNESYYHKLAPDLPKMKGSEVAAK
RRLVQMLQEKYGTIEAFNAAWNGTYASFTALIDAPLYIDSQQAQYDVDDFVKLYLDTFFGTVSRVFRKYD
PNHLLIGDRWLTLPVNNVKVRGFLAEAAGRHLDVISINHYSKNLDTVMLNHIHQASGGKPIMLTEIGYGS
SEQGLGAPTQMLVADQNERMLRYRNYVEGAASLGYIVGVHWFAYLDQAATGRWAEGTTGERYNFGLLNVA
DRPYKPFLEGVMASNKDIYPVLLGQRVPFKHEFGDVPRQPGTNKMEIPYTPAPIPIDGEVNGFPGDAAAV
TLGPGQLVNGTGGEGMQGEYTFAWDENRLYVTAVIKDPTPMKNNNPNINVWRGDGVELFFGPQDLTTPGD
PLFLDRQLIASAGLNNGQPFWYWYFTNRQKPVEMAVKLLPDGSGYVLEAALTWDSINLQGQADTRFLFDF
GFDDSEDGNTRKRQWVWNGTSANSTNRGHWGQATLVKRAAPAIRITGVEEGGDYTDEVLPVVEVEDATGV
RSRTVTLDGAPWTDGTPVTAPGSHELTVEAVNTVGITAKKTVTFAVYGSTALELSPAAGQYSDEVQLEAK
LSGSSGGPVPGAELSFEVNGSAAGTAVTDAQGRAVLPYRISTGPESGQLSVKAAYTPAAGLYLRPAASEG
ALTVRPEDAALQYTGETYVKERRPAVLSAQVVQADDGSAGALSGLPVRFTVSEVRPDGTLRELAGVTDSV
YATGADGRAEAAAQLPPGLYEVRAELLAGPLYTAPAPVRSTLAVAAPVAGHSVSVNGTVPGGTFLGKPAG
TLVLNSMVSYDLAGSLTGALRVSGEPSGAELYVKSFDWLVIAGDRAYVQGTAVSGRQTYTVRLLLKELTT
VSLYVWKGRDTSAEPVHKQLNAQLSGAVLTLP

>BAT46645.1
MPEGVLYVKRKQKLRWDGVQKLKSYLKIKRILAVLLCAVLLIVSCLAGPTTISYAVEASDELPENIRVDK
ADFDTIPDVAEHYKQLFGDNAVSYFKSINGESRIQRINFLQYPEGEYLEVNGQVTIDIPAMDARVWRLFG
DVRFEGSVAQTMELYVIDPNGVETQWTVFKNGGWKDGVTGLYTNYTKMGFQESNPTEGITHPWLIKDENG
DVIYDGYKMKIVGNGTLRSVYHWEEEGVPLEFDTSAWTVLGGDKEILDVSVNVDALTNLSMNGVNKLPEE
VFKRYHVNSGPIGVEQADGEITVLDEAYHKTTNDYGFIPGRGAFHYTLMTSWAGLKEDSERPGYADFTTT
KEIYAKSQPAIDKFESLYPSIGKDYVLTLDGWPKWMWENPNSGQSEHFGTPSYSNFDAAADAAAQLIKSI
DTRFDGLGPKYVEVKNESTIPQEWWFFQSDPQHAWSYLSEFHNKVAEAVKAENPDVLVGGPSSAFMYLEK
NDFDEARAQLKFMDETKDSLDWYSHHFYENSNLFIHDRENNSDGFLSGRMEAVLDLLNAHMVNTDNVKPI
YITEEGTYNTAGSDADYFQKLVAHNGYMLRFMNYSDTIGMLVPYLYPIINWRPNSNDTFYKYNETMNGLM
EEMTPLEAYLDMWKDYRGAFLPSEADQERVYTNAVRYNDKVYVAVHNLNTQRVNLDLNVFTGDANIASVT
RKHFFLEKGKLTYEEENVADLDNVYMRVQEMSVFEITLDSNPSFTKTWERQFAYAPEELVPTSPNAPAVF

KIESLPTTLAKATLRIGFGKTGSGFTTDMSVVVNPDNPANTQNFSKDLEYTNKPGDLLTFAEFELDASKL
LADNTIHIMIPEDGGYITSVQMIQYDEQEAPTGVATDALTASIADAKSKLSSTVVSSTGNEVEQGNKWVE
KHIHDTLNIEVMKAEVVAQDALATDSEINTALEDLTKAMGIFDQYTKTKETPTGNRGAKISFEDGEAVDY
TYNVDTVTTTTDSQGATDGSKALHTEFTSFTSYAWDTTGTYSGSLDFTAPEEGWSLGEDPFTFDVTNLRN
YKIQLRVEISDTSDIKGTYYYALGANASRSISIEDFGVAGGTWLADGNFPRNTAIDTENIKSIRFYVFSP
TEEPITNAALAFDRIIIGNVPNEHTEEQLAAEAADALSADSLIFAEGDTAQAVTNHISLPSVGLHNASIT
WISSHPSIVTNDGTVSRPEYGSGNQTVTLTATITIGAASTTKDIEVTVLQKAATPTSPGGNNSPINPMPS
IPQTTNVDVLVDGKKHDNIAKSVTTKEGDRTVTTITVDQQLLKNSLGNMDNDAIIAIVVANSSDVVYGKL
NGQSVKDLEQKNAVIELRTDTFSYKLPVQQINMNELVQELGSDVKLEDISIDMKISSADADAKKALESSA
VKGGFTVVSPPVDFHITYTYEEKTIEVRTFDTFVERSIVIPEDVNQTQITTGVVVEEDGTVKPIPTRFVK
QDGSYYAIMNSLTNGTYAVIWNPVNFQDMSGHWAENTVNDLGSRLIISGVDGTRFEPDRSITRAEFTAIV
IRAFGLNAEEGESSYSDIARDSWYEAYLNTATTYGLINGYANGQFGPNELITREQVMSIMERAMEITGYE
AVADMSEVDEILTKFKDRTEAAAYAKSGIAASIKAGLVMGRSADVLAPKAAITRAEVAEIVRRLLVNSDL
I

>AEE96522.1
MVISDLTHFLAERCEVNISENGNSIVLDFDKEGGDLICEKDILEQWWRNEGYLILDVLNHEQWVMGVVIK
FWEINSTKPDLSVTMGVLPGIKARLSLPSNALNSQHMFLPRTPGKLKTVVAGQGVDPSKICKLSISSMEC
FTRQRLEIFDMHVAKEEPDYPLPDTVLVDKLGQLITREWYGKTHGEDELKVYLLNESEKSISAQGRCALD
EYGGWQNKRFKASGFFRTEYDGRRWWLVDPEGNAFYSLGMDCVWPGENAYINGIRKFFEWLPDEKGAFKE
AWTISTSNSDENYFNFTIADFIKTFGNVWWGQWARITRRRLLEWGINTIGNWSSPDFIKCARMPYVWPLA
DFPDTEYKVFRDFPDVFSEEYEKNAIKFARQLEPFKEDPYMIGYFLRNEPQWAFIQDLNLARILLEDDNP
SASKDALISFLAKRYDGDIQKLSKEWNIDLLSFDQLKNSLDTTALSPKALADLDDFSEVMIERYVAVPSK
EVKKIDPDHLNLGMRYGYISQDKLLAGHQNFDVFSINCYKMSPASDVDYVGKIIDMPVLVGEFHFGALDR
GLLATGLRAVTDQQQRGVAFKYYVETGAANKYCVGTHYFQLNDQPVLGRYDGENFQIGAVDVCCKPYNEF
TEGIKSASFDLYKVMAGEKSAYNIYPKEIERIGF

>AKV62624.1
MKRLFASLLNLAMIAGLAGQPGTANAATPFPTLNFDNGVIPSYVTGSNAALQIVTNTTGSKALKVNYAAT
DFPSVKFAPTTPWSVGSGNAIAFELTNASNKDITFYLRVDDSAQADGVKDSIVSQAVAKAGTTNQYFLSL
NSAVLDLGMRFLPPNPAGLQMGYAWGDKSIDPANVVSLQFFQMYPSTATALVIDNLRVIQDPNSNLSYLN
GIVDKYGQYSGASWSEKINSDQDLLNDKAEEALALNGSQPIATSQYGGWKNGPKLQATGRFRVAQYGGKW
SLVDPEGYLFFSTGVDVVRLDDMHTWISGRDAMFKDLPAKNSSLGEHFRYTTTVGSPPLGQTEGWLFNHY
SANLERKYGTDYINKWKDVSVARFKNWGFNSLGNWSEPTLFFGKGSQHKLAYVANGWTSWGTHTTIPSGE
WGGVADPYDPQFTVSVSDMVQNQIVAYGVAQDPWLIGIYVDNEIPWGSPATTQSKYLLISNILAMNAADS
KSYAKRAMIAHLKTKYNNNIATLNAQWGTSFASFTAMDAPFKPAQISNGMIPDYSTMLKLLARKYFSIVD
ATLTQALPNTLYLGSRFAEWGISKEVQEAAAEYVDVVSYNVYKESVNGHSWMDIAALNKPAIVGEFAFGS
NDRGMFGTGPNSESAASSQQERAAKFTNYMNAALQNPYFVGAHWFQYVDEPLLGRHWDGENYNLGFVDVA
DVPYASLVNAAKTVHAQAYATRFGSTGNTKISFEAAENLSLISAYNQATIQYVSEGATDGVRAMKVNVGT
LDTVYAGVELKPASPWNLGAAPSVTADVTNPTALPIQIRCNVLDNNGQLRTFYFTVNANASRTITMGSFG
ASPAQTSGADGYWGAVNGLSTTQIKSITFYLWEDAPQSGNSFIMDHLLISK
